# Genetic evidence for the causal effects of C–reactive protein on self-reported habitual sleep duration^☆^

**DOI:** 10.1016/j.bbih.2024.100754

**Published:** 2024-03-11

**Authors:** Olena Iakunchykova, Mengyu Pan, Inge K. Amlien, James M. Roe, Kristine B. Walhovd, Anders M. Fjell, Chi-Hua Chen, Michael E. Benros, Yunpeng Wang

**Affiliations:** a Lifespan Changes in Brain and Cognition (LCBC), Department of Psychology, University of Oslo, 0317, Oslo, Norway; b Division of Radiology and Nuclear Medicine, Oslo University Hospital, Rikshospitalet, POB 4950, Nydalen, 0424, Oslo, Norway; c Department of Radiology, University of California in San Diego, Gilman Drive 9500, 92093, La Jolla, CA, USA; d Copenhagen Research Centre for Mental Health, Mental Health Center Copenhagen, Copenhagen University Hospital, Gentofte Hospitalsvej 15, 2900, Hellerup, Denmark

## Abstract

Inflammatory responses to acute stimuli are proposed to regulate sleep, but the relationship between chronic inflammation and habitual sleep duration is elusive. Here, we study this relation using genetically predicted level of chronic inflammation, indexed by CRP and IL6 signaling, and self-reported sleep duration. By Mendelian randomization analysis, we show that elevated CRP level within <10 mg/L has a homeostatic effect that facilitates maintaining 7–8 h sleep duration per day — making short-sleepers sleep longer (p = 2.42 × 10^−2^) and long-sleepers sleep shorter (1.87 × 10^−7^); but it is not associated with the overall sleep duration (p = 0.17). This homeostatic effect replicated in an independent CRP dataset. We observed causal effects of the soluble interleukin 6 receptor and gp130 on overall sleep duration (p = 1.62 × 10^−8^, p = 2.61 × 10^−58^, respectively), but these effects disappeared when CRP effects were accounted for in the model. Using polygenic score analysis, we found that the homeostatic effect of CRP on sleep duration stems primarily from the genetic variants within the *CRP* gene region: when genetic variants outside of this region were used to predict CRP levels, the opposite direction of effect was observed. In conclusion, we show that elevated CRP level may causally facilitate maintaining an optimal sleep duration that is beneficial to health, thus updating our current knowledge of immune regulation on sleep.

## Highlights

• IL6 signaling and CRP levels are causally associated with self-reported habitual sleep.
• Elevated CRP levels show homeostatic effects on self-reported habitual sleep, independent of soluble IL6 receptor and gp130 levels.
• The homeostatic effects of CRP are mainly driven by cis-genetic variants of the *CRP* gene.

## 1 Introduction

Both immune system functioning and amount of sleep contribute to overall health and risk of disease, and sleep duration and immune function are highly entwined (Irwin, 2019). Alterations to habitual sleep patterns, such as increased sleep need, have been documented as a key component of sickness behavior in response to infections or other immune challenges (Opp and Krueger, 2015). These immunologically induced sleep alterations are believed to play an adaptive role and promote recovery from infection (Irwin and Cole, 2011; Krueger et al., 2016). Thus, acute immune responses and their effects on sleep are considered essential for maintaining homeostatic status and health. However, while both habitual sleep duration and chronic low-grade inflammation have been associated with increased risk of common chronic diseases (Besedovsky et al., 2019; Furman et al., 2019; Li et al., 2022; Sabia et al., 2022; Wang et al., 2019a; Yin et al., 2017), their interrelations have yet to be investigated.

Prior studies have primarily investigated associations between C-reactive protein (CRP) and interleukin-6 (IL6) signaling with sleep related traits and disorders, as these constitute the most studied mechanism of chronic inflammation (Bakour et al., 2017; Garbarino et al., 2021; Ghilotti et al., 2021; Irwin et al., 2016). A meta-analysis of 72 observational studies of self-reported sleep traits and experimental studies of sleep deprivation found that sleep disturbance and extremes of sleep duration were associated with higher levels of CRP and IL6 (Irwin et al., 2016). When used as a continuous variable, shorter sleep duration was associated with higher levels of CRP. However, when short vs. long sleepers were defined compared with an optimal sleep reference and analyzed separately, short sleep duration was not associated with CRP and IL6, but long sleep duration was associated with higher levels of CRP and IL6 (Irwin et al., 2016). Thus, it is justified to take approach of analyzing short vs optimal, long vs optimal sleep duration because the different effects in short or long that would not come to light in an analysis of continuously treated sleep duration.

**Fig. 1:**
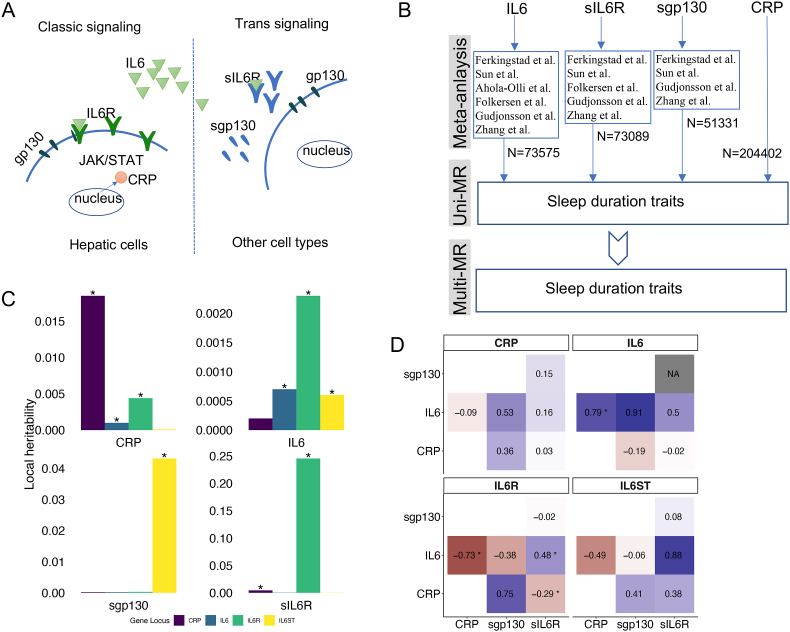
The diagram, analysis strategies, and genetic relations for the IL6 signaling pathway. **A.** A simplified view of the IL6 signaling pathway distinguishing classical and trans signaling. Binding of IL6 molecule to membrane bound IL6 receptor (IL6R) and gp130 initiates the classical pathway which induces the production of CRP in hepatic cells; Binding of IL6 to soluble IL6 receptors (sIL6R) and membrane bound gp130 stimulate the trans pathway on other cell types. **B.** Previously published GWAS summary statistics were meta-analyzed to improve statistical power in uni- and multivariable Mendelian randomization (MR) analysis. The final sample sizes are shown. **C.** The local heritability of IL6 signaling pathway components estimated from SNPs in the four genes which are indicated by colors. **D.** Genetic correlations between pairs of IL6 signaling pathway components (x and y axes) estimated from the four genes shown by four panels. “*” indicates statistically significant estimates (FDR <0.05).

IL6 signaling operates via two major pathways – classical and trans signaling (Fig. 1A) (Del Giudice and Gangestad, 2018). The classical IL6 signaling pathway functions through the membrane-bound IL6 receptor (IL6R), leading to the activation of the JAK/STAT cascade which in hepatocytes stimulates C-reactive protein (CRP) synthesis (Del Giudice and Gangestad, 2018; Ferreira et al., 2013; Philips et al., 2022). Conversely, the trans signaling pathway requires soluble IL6R (sIL6R) through which IL6 can exert its systemic effect on other tissues. Under this simplistic framework, increased sIL6R concentrations are considered to reduce classic signaling by buffering IL6 in circulation and decreasing the availability of the membrane-bound form (Ferreira et al., 2013; Garbers et al., 2014). Moreover, the membrane-bound glycoprotein 130 (gp130) can also be dissociated to soluble gp130 (sgp130), which can bind to circulating IL6-sIL6R complex, thereby reducing trans signaling efficiency.

Large-scale genome wide association studies (GWAS) conducted over the past two decades offer unprecedented opportunity to examine the relationship between chronic inflammation and sleep traits (Bycroft et al., 2018; Ligthart et al., 2018). Because genetic variations are largely stable from conception, such derived relationships by necessity reflect the average relations between traits across the lifespan. Therefore, it is conceivable that these life-course average relationships can differ from those derived from sleep manipulation and from immune stimulation studies, both of which are typically conducted in a short time and primarily reflect the immediate physiological response of one to the other. Mendelian randomization (MR) is a widely used approach to identify the potential causal relationship between risk factors and health outcomes (Sanderson et al., 2022). MR models use genetic variation between individuals as instrumental variables to obtain unconfounded estimates of the effect of the exposure on the outcome (Sanderson et al., 2022). To date, two studies have examined the causal effect of chronic inflammation on sleep problems (Kappelmann et al., 2021; Milaneschi et al., 2021). However, the focus of both studies was mainly depression, and sleep problems were analyzed as sub-symptoms and not directly examined in their own right. More importantly, the effect of low-grade chronic inflammation on *habitual sleep duration* rather than on sleep disorders has not been studied.

Here, we set out to systematically assess the causal effect of low-grade chronic inflammation, indexed by genetically predicted IL6 signaling and circulating CRP levels, on sleep duration traits. We distinguish between short-sleepers (≤6 h per day), those within the generally recommended 7–8 h range, and long sleepers (≥9 h). Because genetic variation is our main instrument, we first delineate the genetic architecture of the IL6 signaling pathway. Guided by this information, we then assess the genetic relationship between low-grade chronic inflammation and sleep duration.

## 2 Materials and methods

### 2.1 GWAS summary statistics data

Sample sizes and characteristics of GWAS data sources are listed in Supplementary Table S1. All studies were approved by an institutional review board at the time of data collection and analysis.

We used the association results from a meta-analytic GWAS of CRP including 204 402 individuals of European ancestry (Ligthart et al., 2018) to compute genetically predicted circulating CRP levels. An inverse-variant weighted meta-analysis using the METAL software (Willer et al., 2010) was performed to boost the statistical power of association statistics for GWAS of circulating levels of IL6 (6 sub-studies, N = 73 575), sIL6R (sIL6R; 5 sub-studies, N = 73 089), sgp130 (4 sub-studies, N = 51 331). GWAS datasets used in the meta-analyses were obtained from the DeCODE study (Ferkingstad et al., 2021), INTERVAL study (Sun et al., 2018), Young Finns study (Ahola-Olli et al., 2017), SCALLOP consortium (Folkersen et al., 2020), AGES-Reykjavik Study (Gudjonsson et al., 2022), and ARIC (Zhang et al., 2022). Genetic variants associated with sleep duration traits were obtained from published GWAS results based on UK biobank (UKBB) data. These traits were short sleep, where short sleepers (≤6 h in 24 h) were compared with normal sleepers (7 or 8 h in 24 h); long sleep, where long sleepers (≥9 h in 24 h) were compared to normal sleepers; and sleep duration (average sleep duration per night in hours) (Dashti et al., 2019).

All association statistics were preprocessed using a consistent protocol before subsequent analysis. Specifically, SNPs having a minor allele frequency (MAF) < 0.05, or imputation INFO <0.5, or ambiguous allelic coding (A/T, or C/G) were removed.

### 2.2 Local heritability and genetic correlations

The local heritability (loc-h^2^) for each inflammatory marker and local genetic correlations (loc-gr) between the markers were estimated using the program LAVA (Werme et al., 2022). This analysis started with selecting SNPs within 50 kb upstream and 50 kb downstream of the coding regions for the four inflammatory markers (*CRP*, *IL6*, *IL6R* and *IL6ST* for sgp130). Next, the preprocessed association statistics were used to estimate loc-h^2^ stemming from these four gene regions for the levels of the four corresponding markers. The 1000 genomes project phase 3 European subpopulation (1KGp3) was used to derive the linkage disequilibrium (LD) matrix required by LAVA. The estimated loc-h^2^ indicates the proportion of the variation in the marker levels explained by genetic variation in the gene region. F-tests were used to assess the significance of estimated loc-h^2^. The obtained p-values were corrected for multiple testing using the Benjamin-Hochberg false discovery rate (FDR) procedure (16 tests); FDR <0.05 was taken as the criterion for statistical significance. Local genetic correlations between pairs of inflammatory markers that arise from each of the four gene regions were also estimated using LAVA (Werme et al., 2022) with the same selected local SNPs and LD matrix.

Whole genome genetic correlations between inflammatory markers and sleep traits were estimated by the linkage disequilibrium score model (ldsc) (Bulik-Sullivan et al., 2015). As recommended by the authors of ldsc, only high-quality HapMap3 SNPs were used for estimation. The linkage disequilibrium score derived from 1KGp3 was used as input to ldsc. The FDR procedure was used to correct for multiple testing for sleep traits and inflammatory markers (9 tests), and FDR <0.05 was considered as criterion for statistical significance.

### 2.3 Two-sample mendelian randomization

To study the cause-effect relations between inflammatory markers and sleep traits a series of two-sample Mendelian randomization (MR) analyses were performed. The most powerful inverse-variance weighted model (IVW) (Bowden et al., 2017) was used in the primary analysis to estimate causal effects; models using Egger regression (Bowden et al., 2015) and MR-PRESSO (Verbanck et al., 2018) were used to guard against potential bias due to horizontal pleiotropy, where genetic variants affect both the exposure (inflammatory markers) and outcome (sleep duration) but via independent, parallel pathways. For MR analyses, only genome wide significant SNPs (p < 5 × 10^−8^) in the local regions (50 kb up-/down-stream of the coding genes) of CRP, sIL6R, and IL6ST were searched for appropriate instruments. The PLINK program (Chang et al., 2015) and the LD structure of 1KGp3 were used to select instruments with the following parameters, --*clump-kb 1 kb*, *--clump-p1 5x10*^*−8*^, and *--clump-r2 0.01*. The TwoSampleMR package v0.5.6 (Hemani et al., 2018) was used for data harmonization and causal inference for the IVW and Egger regression models. The harmonized datasets were also used as input to MR-PRESSO to remove outliers, *i.e.*, SNPs that show horizontal pleiotropy signals. Harmonized instrumental SNPs are shown in Supplementary Tables S4–S12.

A multivariable Mendelian randomization model (MVMR) (Sanderson et al., 2018) was used to estimate the direct causal effect of inflammatory markers on sleep traits. Selected instruments for the above univariable MR models were concatenated as instruments in MVMR models that included the full set of markers simultaneously. FDR <0.05 (9 tests) was used as the threshold for statistical significance.

In addition, to corroborate our results for IL6 signaling, the algorithm described in (Georgakis et al., 2020) was also implemented. Briefly, the SNPs associated with circulating CRP levels (p < 5 × 10^−8^) from the CRP GWAS and located in *IL6R* gene (50 kb up-/down-stream) were selected as potential IL6 signaling instruments. These selected SNPs were then clumped by PLINK using the parameters: -*clump-kb 1 kb*, *--clump-p1 5x10*^*−8*^, and *--clump-r2 0.01*. Lastly, the univariable IVW model was applied to estimate the causal effects of IL6 signaling upon sleep traits.

To provide further evidence for a causal relationship between CRP and sleep traits, we used the association results from a GWAS of circulating CRP levels measured 5–7 days after birth (Wang et al., 2020) as instruments in MR analysis. The genetic architecture of postnatal CRP level is largely similar to that found in adults (Wang et al., 2020), therefore we consider the GWAS of postnatal CRP an appropriate comparator dataset. The same protocols for instrumental SNP selection and univariable MR analysis as described above were used. Harmonized instrumental SNPs are shown in Supplementary Tables S13–S15.

Because insufficient sleep duration may partly reflect sleep disorder, MR analysis using GWAS results for insomnia (Lane et al., 2019), daytime sleepiness (Wang et al., 2019b) and accelerometry measured mean sleep duration (Jones et al., 2019) as outcomes and inflammatory markers as exposures was also conducted. The same protocols for GWAS summary statistics preprocessing, instrumental SNP selection and univariable/multivariable MR analysis as described above were implemented to estimate the causal effect of inflammatory markers on these disorders. Harmonized instrumental SNPs are shown in Supplementary Tables S16-S23 and S25-S27.

### 2.4 Phenotypic and polygenic score (PGS) analysis

The individual-level data from the UKBB were accessed (granted project no. 32048). For these datasets, ethical approval was obtained from the National Health Service and National Research Ethics Service (Ref 11/NW/0382).

Imputed genotypes derived from the UKBB Axiom Array from Affymetrix (about 90% of participants) and the UK BiLEVE Axiom Array (5% participants) were obtained for 487 409 participants. The following preprocessing procedure was performed prior to the analysis: samples with missing call-rate >0.02 or mismatched genetically inferred and self-reported sex were removed. Genotype imputation was performed by the UKBB team and for further details see Sugden et al. (2019). The serum CRP levels for these participants were measured by the UK Biobank biomarker panel (http://www.ukbiobank.ac.uk/uk-biobank-biomarker-panel/). To reduce the impact of recent infections on CRP levels, samples with CRP level >10 mg/L (Ridker, 2003; Sayed et al., 2021) were removed (N = 19 439) from subsequent analysis. Self-reproted habitual sleep duration for each participant during a 24-h period was obtained from answers to the UKBB questionnaire (for details, see (Dashti et al., 2019)). Individuals who reported sleep duration <4 or >14 h (N = 981) were excluded from the analysis. The precomputed genetic principal components, indicating subtle population stratification, were obtained from UKBB. After exclusion, in total, 438 456 inviduals were available for the subsequent analyses. For the accelerometry-based measures, the protocol used by Jones et al. (2019) was followed to compute mean sleep duration. Accelerometry-based measures were available for 103 711 who consented to wearing an activity monitor device (Axivity AX3). After removing inviduals who reported a CRP>10 mg/L, mean sleep duration <4 h or >14 h, or had low quality data (due to data corruption or to non-compliance of the participant with regard to study protocol), 81 465 individuals remained for analysis.

Two PGSs for circulating CRP levels were computed for UKBB participants, using effect size estimates from Ligthart et al. (2018). A trans-PGS was computed using SNPs outside of the CRP coding gene as defined in the MR analysis section; a cis-PGS was computed using the instrumental SNPs for CRP selected for the univariable MR analysis. For the trans-PGS, PLINK was used to select independent SNPs using the following parameters: *-clump-kb 250 --clump-p1 0.9 --clump-r2 0.1*. The *--score* function from PLINK was used to compute the two PGSs.

Linear regression models were used to estimate the associations between CRP, trans-PGS and cis-PGS CRP and sleep duration. These analyses were performed for self-reported overall sleep duration, for those who sleep <7 h, and for those who sleep ≥9 h, respectively. From the accelerometry measured data, three groups were defined as the following, short-sleeper: <6.5h, normal-sleeper: 6.5–8.5h, and long-sleeper (>8.5h). Age at assessment, sex, and the top ten genetic principal components were included as covariates.

### 2.5 Availability of data and materials

Genome-wide association study data sources are openly available as GWAS summary statistics (see references for the relevant publications in Supplementary Table S1). The results of meta-analyses conducted for this publication can be downloaded from figshare.

## 3 Results

### 3.1 Genetic architecture for IL6 signaling

Because previous GWAS studies for the components of the IL6 signaling pathway (IL6, sIL6R, sgp130) were based on relatively small samples, we first performed meta-analysis of these GWAS to increase statistical power to identify genetic associations (Fig. 1B; Methods and Materials; Supplementary Figs. 1–3). Based on the meta-analysis results, we estimated the heritability (h^2^) of the four inflammatory markers (Methods and Materials). Estimating global heritability using genome-wide SNPs, sIL6R had the highest estimated h^2^ (h^2^ = 0.18, p = 0.19), CRP and sgp130 had the intermediate h^2^ = 0.11 (p = 9.32 × 10^−7^) and h2 = 0.05 (p = 3.31 × 10^−2^), respectively, and IL6 had the lowest h^2^ = 0.03 (p = 3.97 × 10^−4^). For IL6, we identified only two independent loci based on data from more than 70 000 individuals: one is the *IL6R* gene region at chromosome 1 and the other is at chromosome 6. As in the previous studies, no associations around the *IL6* gene region (chromosome 7) were identified, (Ahola-Olli et al., 2017). We identified 21 genomic loci significantly associated with sIL6R levels, of which the most significant locus was in the *IL6R* gene region (rs11264233, p = 1.11 × 10^−300^, Supplementary Fig. S2 and Table S2). For sgp130, 13 loci were identified (Supplementary Fig. S3 and Table S3), including its coding gene region, *IL6ST*, which showed the strongest association signal (rs6873542, p = 1.67 × 10^−308^).

We investigated the genetic inter-relations among the four inflammatory markers by estimating local heritability (loc-h^2^) – estimated from SNPs near and within the coding regions (Fig. 1C; **Methods and Materials**). We obtained significant loc-h^2^ for all four markers from their respective coding genes: sIL6R (*IL6R*; loc-h^2^ = 0.25, p < 2 × 10^−300^, FDR<0.05), CRP (*CRP*; loc-h^2^ = 0.019, p < 2 × 10^−300^, FDR<0.05), sgp130 (*IL6ST*; loc-h^2^ = 0.043, p < 2 × 10^−300^, FDR<0.05), and IL6 (*IL6*; loc-h^2^ = 6.78 × 10^−4^, p = 4.21 × 10^−4^, FDR = 3.79 × 10^−3^). Local heritability estimated across-coding genes showed striking patterns that CRP and IL6 had significant estimates from two other genes (Fig. 1c; CRP: *IL6R* (loc-h^2^ = 4.39 × 10^−3^, p = 2.14 × 10^−167^, FDR = 2.78 × 10^−166^) and *IL6* (loc-h^2^ = 1.01 × 10^−3^, p = 6.76 × 10^−26^, FDR = 7.44 × 10^−25^); IL6: *IL6R* (loc-h^2^ = 2.28 × 10^−3^, p = 3.13 × 10^−19^, FDR = 3.13 × 10^−18^) and *IL6ST* (loc-h^2^ = 6.13 × 10^−4^, p = 1.96 × 10^−3^, FDR = 1.57 × 10^−2^)). sgp130 and sIL6R are essentially monogenic, *i.e.*, their coding genes contribute most to their estimated heritability. These results indicate that the genetic influences on CRP and IL6 levels are more complex than that on sgp130 and sIL6R.

We estimated the pair-wise genetic correlations that attribute to each of the four coding genes (local genetic correlation: loc-gr; Fig. 1D; **Methods and Materials**). In contrast to cross-gene loc-h^2^, loc-gr can reveal the direction of pairwise relations. The *IL6R* gene contributed to the negative genetic correlations between CRP and IL6 (loc-gr = −0.73, p = 4.11 × 10^−15^, FDR = 9.4 × 10^−14^), and between CRP and sIL6R (loc-gr = −0.28, p = 1.84 × 10^−17^, FDR = 4.22 × 10^−16^). It also contributed to the positive genetic correlation between IL6 and sIL6R (loc-gr = 0.48, p = 4.82 × 10^−9^, FDR = 1.01 × 10^−7^). The *IL6* gene contributed to the positive genetic correlation between IL6 and CRP (loc-gr = 0.79, p = 1.90 × 10^−5^, FDR = 3.81 × 10^−4^). In sum, these local genetic relations among the four markers are complicated. For example, while we would expect a positive overall correlation between CRP and IL6 levels (Fig. 1A) based on previous observational reports, the loc-gr can be both negative and positive depending on which genetic variants were used for the estimation.

### 3.2 Causal effects of inflammatory markers on sleep traits

We estimated the total and direct casual effects of inflammatory markers on sleep traits using univariable and the multivariable Mendelian randomization (MR) analysis, respectively (**Methods and Materials**). To reduce potential horizontal pleiotropy bias, we selected instrumental SNPs from the corresponding coding-gene regions of the four markers. Since no significant associations for IL6 levels were obtained in its coding region, we excluded IL6 from subsequent analyses.

**Fig. 2:**
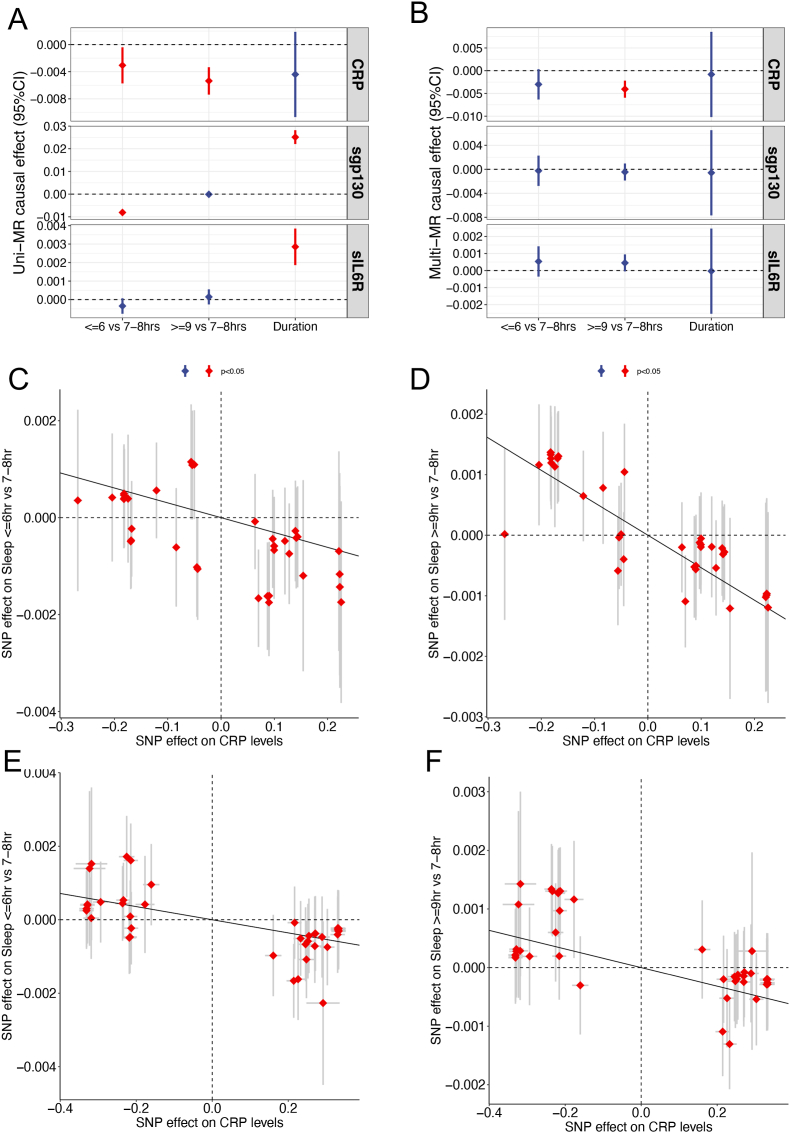
Estimated causal effects of IL6 signaling components on sleep duration traits. **A.** Total causal effects of CRP, sgp130 and sIL6R on sleep duration traits were estimated by univariable Mendelian randomization (MR). **B.** Direct causal effects of the three proteins on sleep duration traits were estimated by multivariable MR. **C.** The scatter plot for the effects of instrumental SNPs on CRP levels and habitual short sleeping. **D.** The scatter plot for the effects of instrumental SNPs on CRP levels and habitual long sleeping. **E.** The scatter plot for the effects of instrumental SNPs on CRP levels from an independent dataset and habitual short sleeping. **F.** The scatter plot for the effects of instrumental SNPs on CRP levels from an independent data and habitual long sleeping. The effects on short (≤6 h) and long (≥9 h) sleep is on the log odds ratio scale comparing to sleep 7–8 h per night. The effect on overall sleep duration is in hours. The slopes of the fitted lines in **C–F** indicate the total causal effects.

Our univariable MR analysis (IVW model) revealed that genetically predicted CRP levels were significantly associated with both the odds of being a habitual short-sleeper (sleep ≤6 h in 24 h) and a habitual long-sleeper (≥9 h) versus normal sleeper (7–8 h). A one unit increase in genetically predicted CRP reduced the log odds ratio (logOR) of being a short sleeper by 0.003 (p = 2.42 × 10^−2^, FDR = 0.12), and reduced the logOR of being a long-sleeper by 0.005 (p = 1.87 × 10^−7^, FDR = 1.12 × 10^−6^) (Fig. 2A–C and D; Table 1). In other words, increasing genetically predicted CRP levels could drive sleeping towards the normal range, *i.e.*, 7–8 h per day. For sgp130, a one unit increase in genetically predicted sgp130 reduced the logOR of being a short-sleeper by 0.008 (p = 2.48 × 10^−34^, FDR = 1.98 × 10^−33^) relative to the recommended range and increased overall sleep duration by 0.025 h (p = 2.61 × 10^−58^, FDR = 2.35 × 10^−57^) (Fig. 2A and Table 1). For sIL6R, a one unit increase in genetically predicted levels was associated with 0.0028 h more overall sleep duration (p = 1.62 × 10^−8^, FDR = 1.12 × 10^−7^; Table 1). Importantly, all these causal relations were neither driven by weak instrumental bias, measured by F-statistics (Supplementary Tables S4–S12), nor by horizontal pleiotropy bias, measured by the Egger regression intercept test and MR-PRESSO global test (Table 1).

**Table 1:** Univariate MR-IVW results for CRP, sIL6R and sgp130.

Marker	Sleep	NSNP	Effect	SE	P	FDR	Padj	Egger_P	PRESSO_P
CRP	Duration	37	−4.40e-3	3.21e-3	0.17	0.22	1.0	0.49	1.0
	Short	37	−3.06e-3	1.36e-3	2.42e-2	4.36e-2	0.22	0.24	1.0
	Long	37	−5.36e-3	1.03e-3	1.87e-7	4.21e-7	1.68e-6	0.53	1.0
sIL6R	Duration	83	2.85e-3	5.05e-4	1.62e-8	4.86e-8	1.46e-7	0.33	0.54
	Short	83	−3.46e-4	2.14e-4	0.11	0.17	0.99	1.46x10-4	0.97
	Long	83	1.46e-4	2.07e-4	0.48	0.54	1.0	0.23	<0.001
sgp130	Duration	54	2.51e-2	1.56e-3	2.61e-58	2.35e-57	2.35e-57	0.06	0.9
	Short	54	−8.09e-3	6.62e-4	2.48e-34	1.12e-33	2.23e-33	0.01	0.89
	Long	54	−1.04e-4	5.00e-4	0.84	0.84	1.0	0.24	1.0

NSNP, the number of SNPs used as instrument; Effect, IVW estimates for causal effect; SE, standard errors for the estimated causal effects; P, IVW p values; FDR, false discovery rate; Padj, adjusted by Bonferroni correction; Egger_P, p values for the test of horizontal pleiotropy by Egger regression; PRESSO_P, MRPRESSOR global test of horizontal pleiotropy.

Given the complex genetic relationships among components of the IL6 signaling pathway, only CRP showed significant direct effects on sleep traits (Table 2, Fig. 2B). A one unit increase in genetically predicted CRP levels reduced the logOR of being a long-sleeper by 0.004 (p = 2.73 × 10^−4^, FDR = 5.0 × 10^−4^) relative to the recommended range. Whereas the effect of CRP on the odds of being a short-sleeper numerically remained the same as in the univariable analysis, it became statistically non-significant (effect = −3.0 × 10^−3^; p = 0.08; Table 2, Fig. 2B). In these multivariable analyses, we did not observe weak instrument bias, as evidenced by the large conditional F-statistics in Table 2. Altogether, our data indicate that circulating CRP levels may have a homeostatic effect in maintaining a recommended optimal habitual sleep duration, *i.e.*, 7–8 h; And this effect is independent of other markers in the IL6 signaling pathway analyzed here.

**Table 2:** Multivariate MR results for CRP, sIL6Ra and sgp130.

Marker	Sleep	Effect	SE	F-stats	P	FDR
CRP	Duration	−8.19e-4	4.78e-3	484.32	0.86	0.98
	Short	−3.00e-3	1.70e-3	484.32	8.09e-2	0.24
	Long	−4.06e-3	9.53e-4	484.32	5.56e-5	5.0e-4
sIL6R	Duration	−3.29e-5	1.28e-3	5376.15	0.98	0.98
	Short	5.35e-4	4.54e-4	5376.15	0.24	0.54
	Long	4.50e-4	2.54e-4	5376.15	8.10e-2	0.24
sgp130	Duration	−5.79e-4	3.63e-3	218.75	0.87	0.98
	Short	−2.47e-4	1.29e-3	218.75	0.85	0.98
	Long	−4.51e-4	7.22e-4	218.75	0.53	0.95

Effect, estimates for direct causal effect; SE, standard errors for the estimated causal effects; F-stats, conditional F statistics; P, IVW p values; FDR, false discovery rate.

As there are no other large-scale sleep duration data available, we attempted replication of the CRP effect on sleep duration using an independent CRP GWAS (Wang et al., 2020) based on circulating CRP levels measured 5–7 days after birth. With these independent genetic results, we again observed significant effects of genetically predicted CRP on short- and long-sleepers in UKBB, but not on overall sleep duration (short-sleeper: effect = −1.59 × 10^−3^, p = 4.18 × 10^−3^; long-sleeper: effect = −1.79 × 10^−3^, p = 7.79 × 10^−4^) (Fig. 2E and F; Supplementary Fig. S6). Thus, these results corroborate our primary findings.

### 3.3 Phenotypic and genetic relations between CRP and sleep duration

**Fig. 3:**
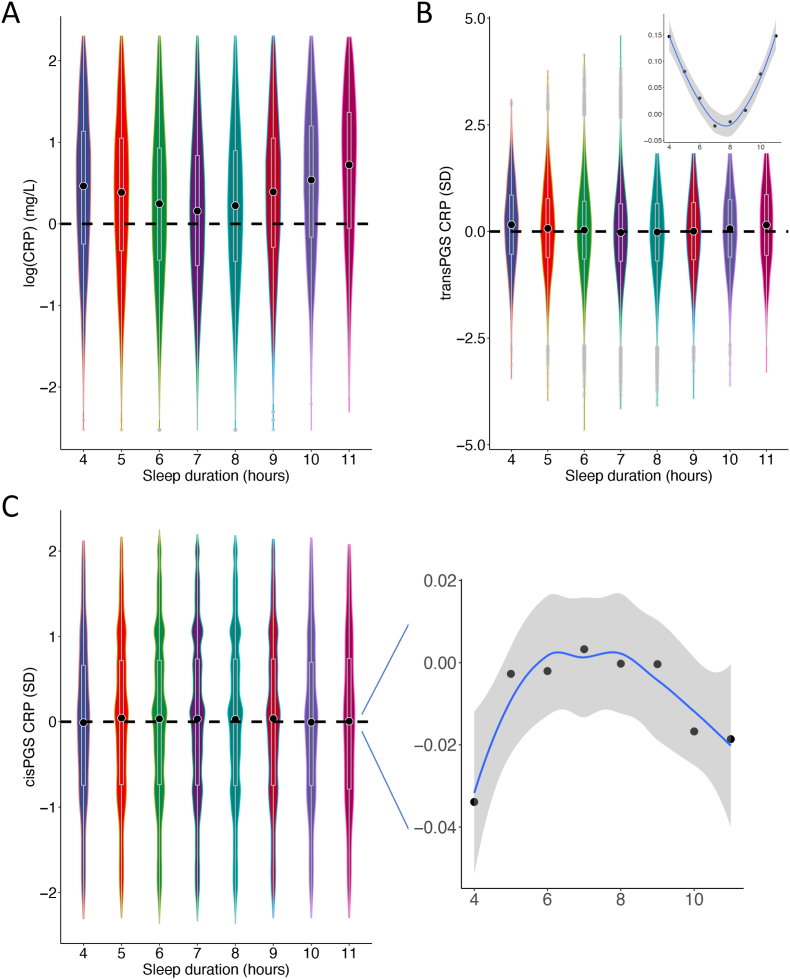
Phenotypic and polygenic relations between CRP and habitual sleep duration. **A.** The relation between logarithm of measured CRP levels (y axis) and habitual sleep duration are shown by box and violin plots. **B.** The box and violin plots for the relation between transPGS of CRP and habitual sleep duration. C. The box and violin plots for the relation between cisPGS of CRP and habitual sleep duration. Sleep durations were divided into eight group (x axis). Circled black dots indicate the median values of CRP value in each group. The relation between the mean CRP levels and sleep durations in each group are zoomed in **B** and **C**, and fitted by LOESS smooth curves in blue.

To reconcile the homeostatic effects of CRP on habitual sleep duration with previously reported deleterious effects of elevated CRP levels (Irwin et al., 2016), we investigated these relations using individual-level data from the UKBB. Fig. 3 shows that, indeed, elevated CRP levels were associated with shorter sleep for short-sleepers and longer sleep for long-sleepers (Fig. 3A, Supplementary Fig. S10, and Supplementary Fig. S7); And importantly, these relations also held for the genetically predicted CRP levels using SNPs outside of the *CRP* gene (transPGS; Fig. 3B). These apparent deleterious effects of CRP on sleep traits are also consistent with genome-wide genetic correlation analysis (Supplementary Fig. S9). However, when using SNPs within the *CRP* gene region (cisPGS), *i.e.*, the instrumental SNPs in MR analysis, the opposite relations appeared (Fig. 3C) – increased cisPGS CRP level showed homeostatic effects on sleep durations.

We then performed a series of regression analyses to statistically test the pattern of results in Fig. 3 (**Methods and Materials**). For short-sleepers, a one standard deviation increase in CRP (1.82 mg/L) was associated with 0.038 h less sleep (p < 2e^−16^); for long-sleepers, a one standard deviation increase in CRP (2.02 mg/L) was associated with 0.05 h more sleep (p < 2e^−16^); for overall sleep duration, a one standard deviation increase in CRP (1.84 mg/L) was associated with 0.006 h more sleep (p = 3.26x10-3). Both cisPGS and transPGS were significantly associated with measured CRP levels (cisPGS: beta = 0.11, p < 2 × 10^−16^; transPGS: beta = 0.15, p < 2 × 10^−16^). Using transPGS as the predictor, a one unit increase in transPGS was associated with 0.01 h less sleep for short-sleepers (p = 2.46 × 10^−14^) and 0.013 h more sleep for long-sleepers (p = 1.25 × 10^−3^), but no association with overall sleep duration (p = 0.66). Interestingly, when cisPGS was used, a one unit increase in cisPGS was associated with 0.003 h more sleep for short-sleepers (p = 2.59 × 10^−2^), 0.007 h less sleep for long-sleepers (p = 0.07), but again, no association with overall sleep duration (p = 0.92). To test if including subjects whose baseline CRP>10 mg/L affects the results, we performed additional analysis based on all full CRP rang in UKBB (Supplementary Additional analysis and results). Again, we observed the similar pattern of CRP effects on sleep duration traits. Thus, these data suggest that the origin of CRP variation matters for self-reported sleep duration: those stemming from genetic variations in the CRP gene could have opposite effects on sleep duration compared to those originating from other genomic regions.

## 4 Discussion

**Fig. 4:**
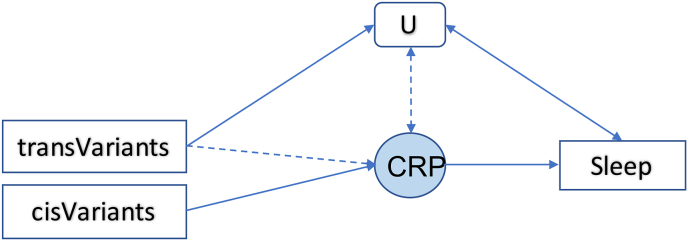
A tentative model explains the results of the present study. Genetic variations in the cis-region contribute to the inter-individual variation in CRP conditioned on all other stimulating factors to CRP expression equal. TransVariants contribute to the variation of CRP levels potentially mediated through their effect on unmeasured/unknown factors (U). These factors could increase CRP levels and at the same time affect sleep traits. When the CRP-sleep effect is in the opposite direction than the U-sleep effect (e.g., increased CRP reduces the risk of sleep disorders but increased U levels increase such risk), then the U-Sleep relation could obscure the true CRP-Sleep relation. This case is highly probable given contemporary knowledge that CRP is highly polygenic and sleep is associated with many health conditions. Whether transVariants have direct effects on CRP levels and whether CRP levels reversely affect U are unclear.

Here we present a thorough genetic analysis on the effect of the IL6 signaling pathway on habitual sleep duration. Rather than analyzing each component of this pathway separately, we accounted for the complex genetically determined relations between them. Based on the results, we postulate that elevated CRP levels within <10 mg/L could potentially facilitate the generally recommended optimal sleep duration, 7–8 h per day. This effect was independent of two other key components of the IL6 signaling pathway: IL6R and gp130. Our findings suggest a testable model for future research (Fig. 4): Genetic variants outside of the *CRP* gene region could influence CRP levels through unmeasured variables (*U* in Fig. 4). These variables may also affect self-reported habitual sleep traits, but in the opposite direction to their effects on CRP. Testing this model could open a new avenue for research to gain understanding on the biological mechanisms of sleep regulation.

Elevated CRP level within <10 mg/L has been previously interpreted as a marker for chronic systemic inflammation and considered to be a cause or consequence of suboptimal health status (Hegazy et al., 2022; Huang et al., 2021; Markozannes et al., 2021; Walker et al., 2019). This low-grade elevation could also be determined at conception by germline genetic variants. Genetic variations in *IL6* and *IL6R* genes that increase the risk of some chronic disorders could potentially reduce the circulating CRP levels, a possibility supported by a recent large-scale association study (Said et al., 2022). Our analysis using cis and trans PGS for CRP and measured CRP levels supports a health promoting effect of CRP. This effect may be in line with recent data suggesting that low-grade CRP elevation may protect against negative health outcomes, including schizophrenia and prostate cancer (Prins et al., 2016; Reay et al.; Said et al., 2022). The valuable insight from our study is demonstration of importance of selecting biologically instruments for causal inference distinguishing between cis and trans genetic variants (Burgess and Cronjé, 2024). While cis variants are biologically motivated and are more likely to reflect the causal relationships, genome-wide approach to instrument selection has produced contradictory answers, especially for CRP (Burgess and Cronjé, 2024).

Evidence provided by animal model studies suggests sleep regulation is linked to the immune system and is dependent on circadian changes of inflammatory cytokine release. Thus, sleep and immune response functions are intertwined since both are responsible for supporting the homeostasis. However, in response to chronic stressors and diseases with elevated levels of inflammatory cytokines, normal sleep can also become dysregulated and loose it adaptive function, and indeed there is evidence for this in humans (Irwin, 2019). Recent research has suggested that immune function related proteins, like cytokines, can influence behavior even outside the context of infection (Salvador et al., 2021). It was hypothesized that certain baseline constitute cytokine levels are required for homeostatic brain function, suggesting co-evolution of the immune and nervous systems to coordinate the behavior (Salvador et al., 2021).

From physiological and biological perspectives, the homeostatic effects of CRP are plausible. CRP exists in two isoforms – soluble pentamer and insoluble monomer. In the face of acute infection/inflammation, the pentameric form of CRP, produced mainly in the liver, dissociates irreversibly into the monomeric form that acts locally as pro-inflammatory factors, e.g., activating the complement system (Du Clos and Mold, 2004). Still, CRP-induced complement activation does not lead to the C5–C9 activation (Du Clos and Mold, 2004) that amplifies pro-inflammatory signals. Thus, the pro-inflammatory effect of CRP is highly regulated and plays a beneficial role for an organism's local defense. CRP has anti-inflammatory functions as well. In vivo and in vitro work has shown that CRP can opsonize endogenous and exogeneous antigens to facilitate cellular clearance by other immune cells (Black et al., 2004). These beneficial effects of CRP on the organism have also gained support from evolutionary studies (Pathak and Agrawal, 2019; Torzewski, 2022). Therefore, both the data presented here and in previous studies may call for a revision of binary interpretations of low-grade CRP elevation in medical research (Del Giudice and Gangestad, 2018).

Previous studies have primarily interpreted elevated CRP levels in the low range as an indicator of chronic inflammation. After the activation of the classic signaling pathway in Fig. 1A, IL6 binds to the membrane IL6R on hepatocytes, leading to elevated CRP synthesis (Ferreira et al., 2013). As such, elevated CRP level has been frequently used as an indicator for on-going infection/inflammation. Recently, genetically predicted elevated CRP level has also been used as an indicator of low activity of the trans IL6 signaling pathway through which IL6 may function on a systemic level (Georgakis et al., 2020). Two studies have employed this strategy and performed univariable MR analysis examining the effect of IL6 trans signaling on depression-related sleep-problems/disorders (Kappelmann et al., 2021; Milaneschi et al., 2021). Both studies implied a weak causal effect of IL6 signaling on sleep problems, whereas, Milaneschi et al. also found a protective effect of CRP on the risk of insomnia (Milaneschi et al., 2021). We found, instead, a protective effect of IL6 trans signaling on the risk of insomnia using both direct instruments for sIL6R and the indirect Georgakis et al. model (Georgakis et al., 2020) (Supplementary Fig. S4). Importantly, however, we argue that the effect of IL6 trans signaling interpretation can be problematic, since our multivariable MR clearly showed that the effect of sIL6R on insomnia is confounded or mediated by CRP (Supplementary Fig. S5). Therefore, future studies should take caution when interpreting univariable analyses, especially in biological pathways where extensive inter-component interactions have been established, such as the IL6 signaling pathway.

Several strengths of our study allowed us to overcome some of the limitations in previous studies. Before looking into the effect of IL6 signaling pathway on sleep traits, we thoroughly delineated the genetic relations between components in this pathway. While our efforts were inevitably limited by the current availability of data, we leveraged all publicly accessible GWAS by conducting a meta-analysis. As a novel finding, we have shown that the local genetic correlations between CRP and IL6 can be positive or negative depending on the location of the genetic variants in the genome used. Delineating the genetic architecture of the IL6 signaling pathway made it possible to properly design the Mendelian randomization study. The uncovered genetic architecture strongly suggested multivariable analysis which revealed CRP as the sole culprit for the effect of IL6-signaling on sleep duration. We reconcile this finding with previous work by analyzing large-scale individual-level data — which leads us to the tentative explanation of the apparent discrepancies (Fig. 4). While a significant amount of previous research has indicated a role of the immune system in sleep regulation, epidemiological studies on this topic are often affected by limitations due to confounding of exposure-outcome associations through environmental factors; among others, chronic inflammation is observed in persons exposed to stress, certain types of diet, smoking, and air pollution. By contrast, MR studies employ instrumental variables based on genotype thereby taking advantage of the random allocation of parental alleles to zygotes at meiosis which is independent of environmental factors and reducing the risk of confounding (Swerdlow et al., 2016). Nevertheless, there were also limitations. Even though we collected all publicly available data to our knowledge, the sample sizes for IL6, sIL6R and sgp130 GWAS were smaller than for CRP. Therefore, the disparity in statistical power achieved by GWAS of the different components of the IL6 signaling pathway likely influences the results. Besides, due to the complexity of the IL6 signaling pathway (Paludan et al., 2021), we could not analyze all its components even in our simplistic depiction. Another limitation of the present study is its generalizability. UK biobank is not fully representative of the UK population; so it is not possible to estimate generalizable disease prevalence and incidence rates (Fry et al., 2017). The previous analysis comparing with non-participants that has shown that participants of UK biobank have higher education, less chronic diseases, lower risk factor prevalence, and lower mortality compared to the whole UK population (Fry et al., 2017; Littlejohns et al., 2019). Another study to directly compare risk factor associations in UK Biobank with nationally representative cohort studies has shown similar magnitude for associations between risk factors and outcomes (Batty et al., 2020). Therefore, it is argued that UK Biobank sample is suitable to detect generalizable associations between most baseline characteristics and outcomes (Batty et al., 2020; Littlejohns et al., 2019).

Further, the GWAS results used in this study were obtained from people of White ethnicity and may not be generalizable to other ethnicities. In addition, while our findings are robust with self-reported sleep traits they are not with accelerometry-measured sleep duration (Supplementary Table S24).

Although the focus of this study is the effect of chronic inflammation on habitual sleep duration, a plethora of studies of sleep deprivation and immune stimulation unequivocally showed the bidirectional relations between sleep and immune response (Irwin and Opp, 2017). We chose to focus only on one direction — inflammation markers to sleep traits — for the following reasons. First, the components of the IL6 signaling pathway have well defined coding genes that allowed us to perform cis MR which greatly reduces the risk of horizontal pleiotropy. Thus, we were able to obtain robust findings. Second, the largest sleep duration GWAS showed the genetic complexity of this trait: genome-wide SNPs could barely account for less than 10% of trait variance (Dashti et al., 2019) and genome-wide significantly associated SNPs account for <1% of the variance. These facts would make MR analysis using sleep traits as exposures challenging due to weak instrument and horizontal pleiotropy bias. Last, we underscore that although MR is a cost-effective study design to provide causal inference, carefully controlled experiments that aim to manipulate the biomarker levels of the IL6 signaling pathway and conduct long-term follow-up of sleep duration are necessary to confirm cause-effect relations.

## 5 Conclusions and perspectives

The immune-sleep relationship is complex, and the components of the immune system can affect sleep duration by contributing to sleep regulation. Given the long co-evolution of immune and nervous systems (Salvador et al., 2021), this interplay may have left genetic footprints in the genome. This study, as well our previous research (Fjell et al., 2023), suggest that habitual sleep duration in the optimal range may be partly under genetic regulation via immune system related genes.

## Funding

This study is supported by the 10.13039/501100005416Norwegian Research Council to YW (No.302854) and the 10.13039/501100009566UiO:Life Science Convergence environment (4MENT), 10.13039/501100005366University of Oslo, Norway.

## CRediT authorship contribution statement

**Olena Iakunchykova:** Writing – review & editing, Writing – original draft, Formal analysis, Conceptualization. **Mengyu Pan:** Writing – review & editing, Formal analysis, Data curation. **Inge K. Amlien:** Writing – review & editing, Formal analysis, Data curation. **James M. Roe:** Writing – review & editing. **Kristine B. Walhovd:** Writing – review & editing, Investigation. **Anders M. Fjell:** Writing – review & editing, Investigation. **Chi-Hua Chen:** Writing – review & editing, Investigation. **Michael E. Benros:** Writing – review & editing, Investigation. **Yunpeng Wang:** Writing – review & editing, Writing – original draft, Formal analysis, Conceptualization.

## Declaration of competing interest

The authors declare that they have no known competing financial interests or personal relationships that could have appeared to influence the work reported in this paper.

## Data Availability

Data will be made available on request.
