# Supplementary material for: Genetic evidence for the causal effects of C–reactive protein on self-reported habitual sleep duration
Source: Brain Behav Immun Health. 2024 Mar 11;37:100754. doi: 10.1016/j.bbih.2024.100754 (PMC10950822; doi:10.1016/j.bbih.2024.100754)
Supplement: Multimedia component 1 [file mmc1.docx]

**Supporting Information**

**Genetic evidence for the causal effects of C–reactive protein on self-reported habitual sleep duration**

Iakunchykova et al.

Table of Contents

[Additional analysis and results. 3](#_Toc158718853)

[Figure S1. QQ and Manhattan plots for meta-analyzed IL6 GWAS. 4](#_Toc158718854)

[Figure S2. QQ and Manhattan plots for meta-analyzed sIL6R GWAS. 5](#_Toc158718855)

[Figure S3. QQ and Manhattan plots for meta-analyzed sgp130 GWAS. 6](#_Toc158718856)

[Figure S4. Univariate MR of inflammatory markers and sleep related traits and disorders. 7](#_Toc158718857)

[Figure S5. multivariate MR of inflammatory markers and sleep related traits and disorders. 8](#_Toc158718858)

[Figure S6. Univariate MR of CRP measure around birth and sleep related traits and disorders. 9](#_Toc158718859)

[Figure S7. Relation between CRP levels and accelerometry measured sleep duration. 10](#_Toc158718860)

[Figure S8. Relation between transPGS for CRP and accelerometry measured sleep duration. 11](#_Toc158718861)

[Figure S9. Genome wide genetic correlation between inflammatory markers and sleep traits. 12](#_Toc158718862)

[Table S1. GWAS data sources. 13](#_Toc158718863)

[Table S2. Genomic loci associated with sIL6R. 16](#_Toc158718864)

[Table S3. Genomic loci associated with sgp130. 17](#_Toc158718865)

[Table S4. Harmonized instrumental cisSNPs (p<5x10^-8^) for CRP to Short Sleep MR. 18](#_Toc158718866)

[Table S5. Harmonized instrumental cisSNPs (p<5x10^-8^) for CRP to Long Sleep MR. 20](#_Toc158718867)

[Table S6. Harmonized instrumental cisSNPs (p<5x10^-8^) for CRP to Sleep Duration MR. 22](#_Toc158718868)

[Table S7. Harmonized instrumental cisSNPs (p<5x10^-8^) for sIL6R to Short Sleep MR. 24](#_Toc158718869)

[Table S8. Harmonized instrumental cisSNPs (p<5x10^-8^) for sIL6R to Long Sleep MR. 27](#_Toc158718870)

[Table S9. Harmonized instrumental cisSNPs (p<5x10^-8^) for sIL6R to Sleep Duration MR. 30](#_Toc158718871)

[Table S10. Harmonized instrumental cisSNPs (p<5x10^-8^) for sgp130 to Short Sleep MR. 33](#_Toc158718872)

[Table S11. Harmonized instrumental cisSNPs (p<5x10^-8^) for sgp130 to Long Sleep MR. 35](#_Toc158718873)

[Table S12. Harmonized instrumental cisSNPs (p<5x10^-8^) for sgp130 to Sleep Duration MR. 37](#_Toc158718874)

[Table S13. Harmonized instrumental cisSNPs (p<5x10^-8^) for CRP in iPsych to Short Sleep MR. 39](#_Toc158718875)

[Table S14. Harmonized instrumental cisSNPs (p<5x10^-8^) for CRP in iPsych to Long Sleep MR. 41](#_Toc158718876)

[Table S15. Harmonized instrumental cisSNPs (p<5x10^-8^) for CRP in iPsych to Sleep Duration MR. 43](#_Toc158718877)

[Table S16. Harmonized instrumental cisSNPs (p<5x10^-8^) for CRP to Insomnia MR. 45](#_Toc158718878)

[Table S17. Harmonized instrumental cisSNPs (p<5x10^-8^) for CRP to Sleepiness MR. 46](#_Toc158718879)

[Table S18. Harmonized instrumental cisSNPs (p<5x10^-8^) for sIL6R to Insomnia MR. 48](#_Toc158718880)

[Table S19. Harmonized instrumental cisSNPs (p<5x10^-8^) for sIL6R to Sleepiness MR. 51](#_Toc158718881)

[Table S20. Harmonized instrumental cis SNPs (p<5x10^-8^) for sgp130 to Insomnia MR. 54](#_Toc158718882)

[Table S21. Harmonized instrumental cisSNPs (p<5x10^-8^) for sgp130 to Sleepiness MR. 56](#_Toc158718883)

[Table S22. Harmonized instrumental cisSNPs (p<5x10^-8^) for CRP in iPsych to Insomnia MR. 58](#_Toc158718884)

[Table S23. Harmonized instrumental cisSNPs (p<5x10^-8^) for CRP in iPsych to Sleepiness MR. 60](#_Toc158718885)

[Table S24. Causal effects of inflammatory markers on accelerometry measured sleep duration. 62](#_Toc158718886)

[Table S25. Harmonized instrumental cisSNPs (p<5x10^-8^) for CRP to accelerometry measure sleep duration MR. 63](#_Toc158718887)

[Table S26. Harmonized instrumental cisSNPs (p<5x10^-8^) for sgp130 to accelerometry measure sleep duration MR. 65](#_Toc158718888)

[Table S27. Harmonized instrumental cisSNPs (p<5x10^-8^) for sIL6R to accelerometry measure sleep duration MR. 67](#_Toc158718889)

# Additional analysis and results.

We included the 19439 individuals whose CRP levels were measured above 10mg/L at baseline, range from 0.08 mg/L to 79.96 mg/L. For short-sleepers, a one standard deviation increase in CRP (4.23 mg/L) was associated with 0.032 hours less sleep (p<2x10^-16^); for long-sleepers, a one standard deviation increase in CRP (5.16 mg/L) was associated with 0.06 hours more sleep (p<2x10^-16^); for overall sleep duration, a one standard deviation increase in CRP (4.33 mg/L) was associated with 0.007 hours more sleep (p=5.53x10^-5^). Both cisPGS and transPGS were significantly associated with measured CRP levels (cisPGS: beta=0.13, p<2x10^-16^; transPGS: beta=0.17, p<2x10^-16^). Using transPGS as the predictor, a one unit increase in transPGS was associated with 0.011 hours less sleep for short-sleepers (p<2x10^-16^) and 0.014 hours more sleep for long-sleepers (p=2.70x10^-4^), but no association with overall sleep duration (p=0.88). Interestingly, when cisPGS was used, a one unit increase in cisPGS was associated with 0.002 hours more sleep for short-sleepers (p=0.11), 0.005 hours less sleep for long-sleepers (p=0.20), but again, no association with overall sleep duration (p=0.95).

# Figure S1. QQ and Manhattan plots for meta-analyzed IL6 GWAS.

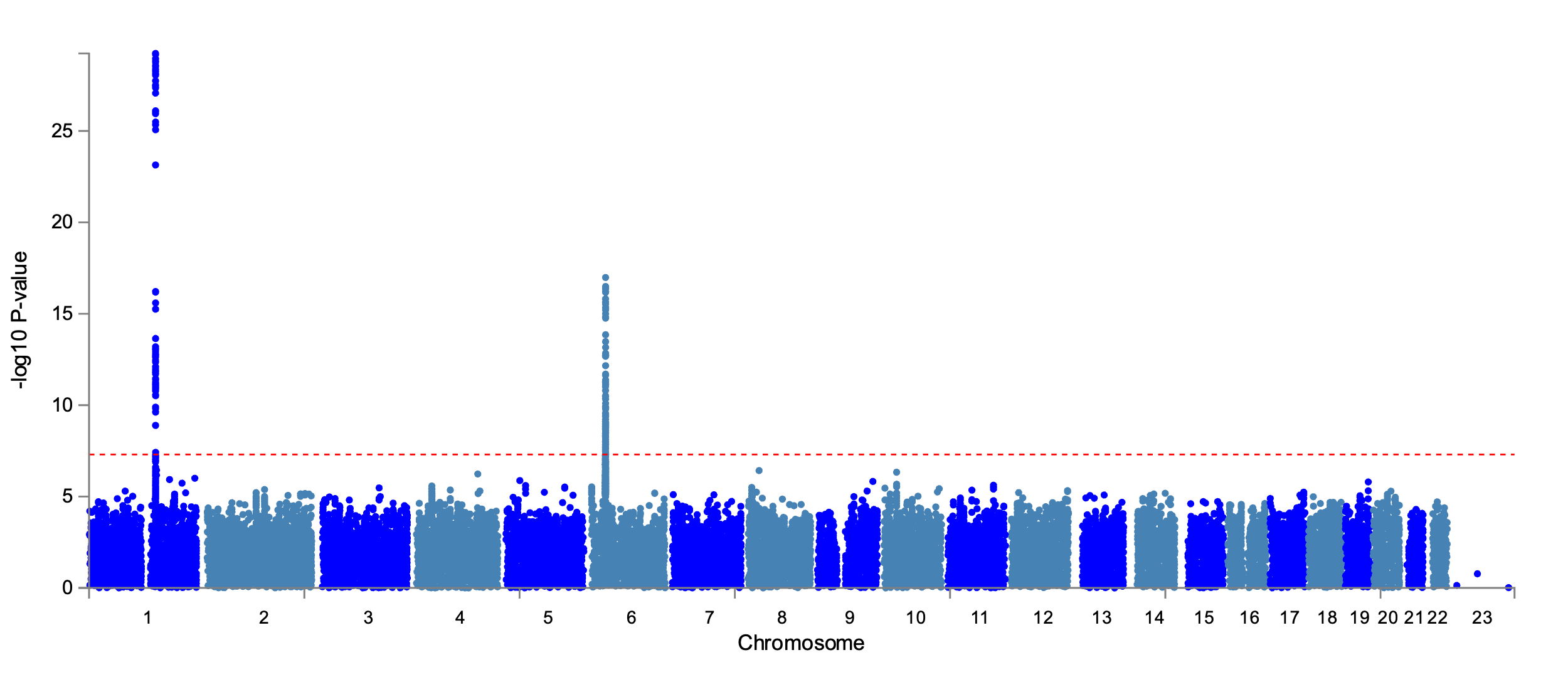


Genome wide association statistics for IL6 from sub-studies and meta-analysis result are presented by QQ plot in the top panel; colors indicate different studies. Bottom panel: the Manhattan plot of meta-analysis results. Sub1: DeCODE, N=35449; Sub2: SCALLOP consortium, N=14244; Sub3: INTERVAL study, N=3301; Sub4: AGES-Reykjavik study, N=5368; Sub5: ARIC, N=7213; Sub6: Young Finns Study, FINRISK2002, N=8000. For details on studies see Table S1.

# Figure S2. QQ and Manhattan plots for meta-analyzed sIL6R GWAS.


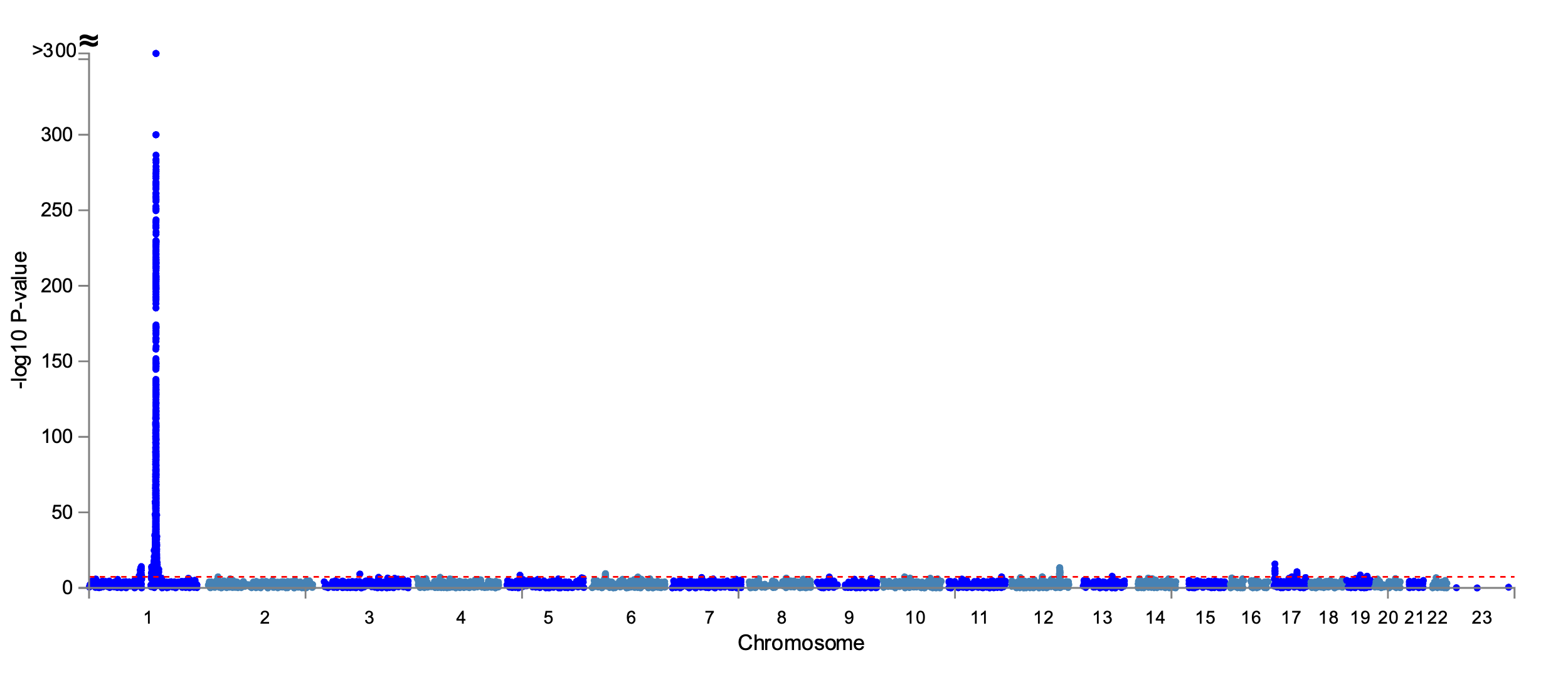

Genome wide association statistics for sIL6R from sub-studies and meta-analysis result are presented by QQ plot in the top panel; colors indicate different studies. Bottom panel: the Manhattan plot of meta-analysis results. Sub1: DeCODE, N=35449; Sub2: SCALLOP consortium, N=21758; Sub3: INTERVAL study, N=3301; Sub4: AGES-Reykjavik study, N=5368; Sub5: ARIC, N=7213; For details on studies see Table S1.

# Figure S3. QQ and Manhattan plots for meta-analyzed sgp130 GWAS.

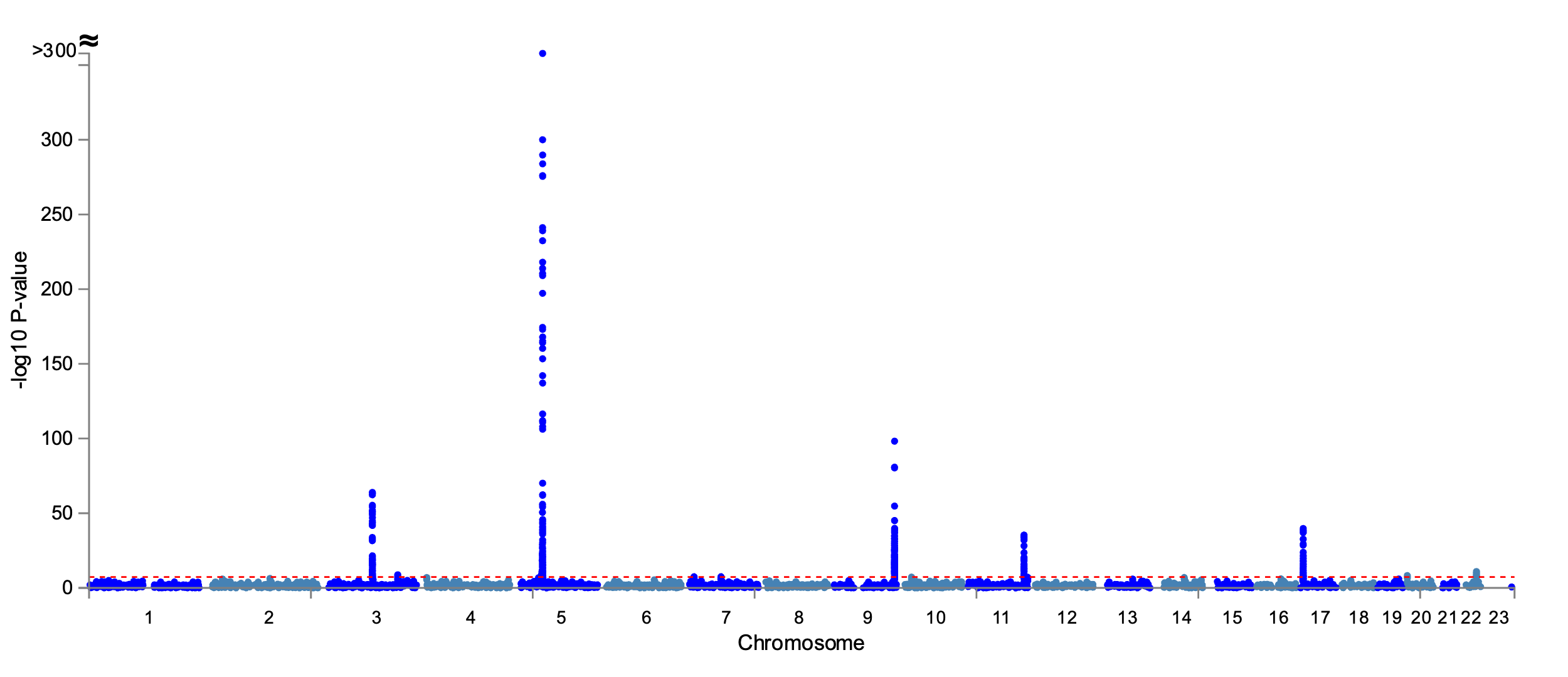


Genome wide association statistics for sgp130 from sub-studies and meta-analysis result are presented by QQ plot in the top panel; colors indicate different studies. Bottom panel: the Manhattan plot of meta-analysis results. Sub1: DeCODE, N=35449; Sub2: INTERVAL study, N=3301; Sub3: AGES-Reykjavik study, N=5368; Sub4: ARIC, N=7213; For details on studies see Table S1.

# Figure S4. Univariate MR of inflammatory markers and sleep related traits and disorders.

Total causal effects estimated by univariable Mendelian randomization (Uni-MR) between IL6 trans signaling and sleep traits. IL6sig used the Georgakis *et al* model to construct instruments; sIL6R directly using SNPs from the sIL6R GWAS meta-analysis. Significance level was set at FDR <0.05, which are highlighted in red.

# Figure S5. multivariate MR of inflammatory markers and sleep related traits and disorders.

Direct causal effects estimated by mutivariable Mendelian randomization (Multi-MR) between inflammatory markers and sleep traits. Significance level was set at FDR <0.05, which are highlighted in red.

# Figure S6. Univariate MR of CRP measure around birth and sleep related traits and disorders.

Total causal effects estimated by univariable Mendelian randomization (Uni-MR) between CRP levels measure around birth and sleep traits. Significance level was set at FDR <0.05, which are highlighted in red.

# Figure S7. Relation between CRP levels and accelerometry measured sleep duration.

# Figure S8. Relation between transPGS for CRP and accelerometry measured sleep duration.

# Figure S9. Genome wide genetic correlation between inflammatory markers and sleep traits.

short sleep: <= 6 hours compared to normal 7-8 hours sleep per day; Long sleep: >= 9 hours compared to normal 7-8 hours sleep per day; Overall sleep duration (Duration). “*” indicates FDR <0.05.

# **Table S1. GWAS data sources.**

| Phenotype | GWAS data source | Sample size | Type of biomarker assay | Covariates and exclusions |
| --- | --- | --- | --- | --- |
| ***Inflammatory markers*** |  |  |  |  |
| CRP levels | Meta-analysis of 88 studies of European individuals  (Ligthart et al., 2018) | 204402 | High sensitivity CRP laboratory assays | Exclusions: >4 SD above the mean, autoimmune disease, immunotherapy. Log-transformed protein values; covariates: age, sex, population substructure, relatedness. |
| Downregulated IL6 signaling | Meta-analysis of 88 studies of European individuals  (Ligthart *et al.*, 2018) | 204402 | High sensitivity CRP laboratory assays | Exclusions: >4 SD above the mean, autoimmune disease, immunotherapy. Log-transformed protein values; covariates: age, sex, population substructure, relatedness. |
| IL6 | Meta-analysis of GWAS | 73575 |  |  |
|  | DeCODE, Iceland  (Ferkingstad et al., 2021) | 35449 | SomaScan multiplex aptamer assay (version 4) , SOMAmer 4673.13 | Rank-inverse normal transformed levels adjusted for age, sex and sample age. LD score regression to account for inflation in test statistics due to cryptic relatedness and stratification |
|  | Healthy blood donors in INTERVAL study  (Sun et al., 2018) | 3301 | SomaScan multiplex aptamer assay, SOMAmer 4673.13 | Log-transformed protein levels were adjusted in a linear regression for age, sex, duration between blood draw and processing (binary, ≤1 day/>1day) and the first three principal components of ancestry from multi-dimensional scaling. The rank-inverse normalized from linear regression were used as phenotypes for association testing. |
|  | Young Finns Study (YFS), FINRISK2002  (Ahola-Olli et al., 2017) | 8000 | Bio-Rad’s premixed Bio-Plex Pro Human Cytokine 27-plex Assay and 21-plex Assay, and Bio-Plex 200 reader with Bio-Plex 6.0 software. | Exclusions: below or above laboratory analysis detection limits. The inverse-normal transformed protein levels were adjusted for age, sex, body mass index, and the first ten genetic principal components by calculating residuals of linear regression model. Subsequently, another inverse transformation was performed for model residuals to ensure normally distributed phenotypes. |
|  | SCALLOP consortium (13 studies)  (Folkersen et al., 2020) | 14244 | Olink proximity extension assay (PEA) cardiovascular (CVD) I panel | Before running the genetic analyses, NPX values of proteins (on the log2 scale) were rank-based, inverse-normal transformed and/or standardized to unit variance, thus avoiding potential Olink batch differences between cohorts. Genetic analyses were conducted using additive model regressions adjusted for population structure and study-specific parameters. |
|  | AGES-Reykjavik Study  (Gudjonsson et al., 2022) | 5368 | SomaScan multiplex aptamer assay | Box-Cox transformation was applied on the protein data and extreme outlier values were excluded, defined as values above the 99.5th percentile of the distribution of 99th percentile cutoffs across all proteins after scaling. Linear regression model was used to control for age, sex, 5 genetic principal components, and genotyping platform. |
|  | ARIC, community-based cohort,  European ancestry  (Zhang et al., 2022) | 7213 | SomaLogic Inc. using the V4 platform by an aptamer (SOMAmer)-based approach | The log-transformed relative abundances of SOMAmers were adjusted in a linear regression model including PEER factors and sex, age, study site and ten genetic PCs. The residuals from this linear regression were then rank-inverse normalized. |
| sIL-6R | Meta-analysis of GWAS | 73089 |  |  |
|  | DeCODE, Iceland  (Ferkingstad et al., 2021) | 35449 | SomaScan multiplex aptamer assay (version 4), SOMAmer 15602.43 | Rank-inverse normal transformed levels adjusted for age, sex and sample age. LD score regression to account for inflation in test statistics due to cryptic relatedness and stratification |
|  | Healthy blood donors in INTERVAL study  (Sun et al., 2018) | 3301 | SomaScan multiplex aptamer assay, SOMAmer 4139.71 | Log-transformed protein levels were adjusted in a linear regression for age, sex, duration between blood draw and processing (binary, ≤1 day/>1day) and the first three principal components of ancestry from multi-dimensional scaling. The rank-inverse normalized from linear regression were used as phenotypes for association testing. |
|  | SCALLOP consortium (13 studies)  (Folkersen et al., 2020) | 21758 | Olink proximity extension assay (PEA) cardiovascular (CVD) I panel | Before running the genetic analyses, NPX values of proteins (on the log2 scale) were rank-based, inverse-normal transformed and/or standardized to unit variance, thus avoiding potential Olink batch differences between cohorts. Genetic analyses were conducted using additive model regressions adjusted for population structure and study-specific parameters. |
|  | AGES-Reykjavik Study  (Gudjonsson et al., 2022) | 5368 | SomaScan multiplex aptamer assay | Box-Cox transformation was applied on the protein data and extreme outlier values were excluded, defined as values above the 99.5th percentile of the distribution of 99th percentile cutoffs across all proteins after scaling. Linear regression model was used to control for age, sex, 5 genetic principal components, and genotyping platform. |
|  | ARIC, community-based cohort,  European ancestry  (Zhang et al., 2022) | 7213 | SomaLogic Inc. using the V4 platform by an aptamer (SOMAmer)-based approach, SOMAmer 15602.43 | The log-transformed relative abundances of SOMAmers were adjusted in a linear regression model including PEER factors and sex, age, study site and ten genetic PCs. The residuals from this linear regression were then rank-inverse normalized. |
| sIL6ST (sgp130) | Meta-analysis of GWAS | 51331 |  |  |
|  | DeCODE, Iceland  (Ferkingstad et al., 2021) | 35449 | SomaScan multiplex aptamer assay (version 4), SOMAmer 2620.4 | Rank-inverse normal transformed levels adjusted for age, sex and sample age. LD score regression to account for inflation in test statistics due to cryptic relatedness and stratification |
|  | Healthy blood donors in INTERVAL study  (Sun et al., 2018) | 3301 | SomaScan multiplex aptamer assay, SOMAmer 2620.4 | Log-transformed protein levels were adjusted in a linear regression for age, sex, duration between blood draw and processing (binary, ≤1 day/>1day) and the first three principal components of ancestry from multi-dimensional scaling. The rank-inverse normalized from linear regression were used as phenotypes for association testing. |
|  | AGES-Reykjavik Study  (Gudjonsson et al., 2022) | 5368 | SomaScan multiplex aptamer assay | Box-Cox transformation was applied on the protein data and extreme outlier values were excluded, defined as values above the 99.5th percentile of the distribution of 99th percentile cutoffs across all proteins after scaling. Linear regression model was used to control for age, sex, 5 genetic principal components, and genotyping platform. |
|  | ARIC, community-based cohort,  European ancestry  (Zhang et al., 2022) | 7213 | SomaLogic Inc. using the V4 platform by an aptamer (SOMAmer)-based approach, SOMAmer 2620.4 | The log-transformed relative abundances of SOMAmers were adjusted in a linear regression model including PEER factors and sex, age, study site and ten genetic PCs. The residuals from this linear regression were then rank-inverse normalized. |
| CRP levels in newborns | iPsych  (Pedersen et al., 2018) |  |  |  |
| ***Sleep traits*** |  |  |  |  |
| Sleep duration | UK Biobank  (Dashti et al., 2019) | 446 118 | About how many hours sleep do you get in every 24 h? (please include naps), with responses in hour increments. Sleep duration was treated as a continuous variable. | Adjusted for age, sex, 10 principal components of ancestry, genotyping array, and genetic correlation matrix |
| Short sleep (vs 7-8 hours) | UK Biobank  (Dashti *et al.*, 2019) | 106 192 cases and 305 742 controls | About how many hours sleep do you get in every 24 h? (please include naps), with responses in hour increments. Sleep duration was categorized as either short (6 h or less), normal (7 or 8 h) | Adjusted for age, sex, 10 principal components of ancestry, genotyping array, and genetic correlation matrix |
| Long sleep (vs 7-8 hours) | UK Biobank  (Dashti *et al.*, 2019) | 34 184 and 305 742 controls | About how many hours sleep do you get in every 24 h? (please include naps), with responses in hour increments. Sleep duration normal (7 or 8 h), or long (9 h or more) sleep duration. | Adjusted for age, sex, 10 principal components of ancestry, genotyping array, and genetic correlation matrix |
| Frequent insomnia symptoms | UK Biobank  (Lane et al., 2019) | 453 379 | Two parallel GWAS: (i) frequent insomnia symptoms (“never/rarely” versus “usually”; n = 129,270 cases and 108,357 controls) and (ii) any insomnia symptoms (“never/rarely” versus “sometimes”/”usually”; n = 345,022 cases and 108,357 controls) | Adjusted for age, sex, ten principal components of ancestry, and genotyping array using |
| Excessive daytime sleepiness | UK Biobank  (Wang et al., 2019) | 452 071 | “How likely are you to doze off or fall asleep during the daytime when you don’t mean to? (e.g.: when working, reading or driving)”, with the answer categories “never” (N = 347,285), “sometimes” (N = 92,794), “often” (N = 11,963), or “all of the time” (N = 29). | Adjusting for age, sex, genotyping array, 10 PCs, and genetic relatedness matrix |

# **Table S2. Genomic loci associated with sIL6R.**

| Locus | chr | uniqID | rsID | pos | start | end | p |
| --- | --- | --- | --- | --- | --- | --- | --- |
| 1 | 1 | 1:115878242:G:T | rs6673867 | 115878242 | 115876618 | 115879821 | 8.85e-09 |
| 2 | 1 | 1:120596340:A:G | rs587633342 | 120596340 | 117723631 | 120663982 | 6.94e-15 |
| 3 | 1 | 1:121477012:A:G | rs587774109 | 121477012 | 121477012 | 121477012 | 1.77e-08 |
| 4 | 1 | 1:145089772:A:G | rs587771308 | 145089772 | 144521971 | 145749076 | 6.55e-13 |
| 5 | 1 | 1:147041130:C:G | rs76915123 | 147041130 | 146246117 | 147824088 | 1.17e-13 |
| 6 | 1 | 1:154625632:A:G | rs11264233 | 154625632 | 148234791 | 159067841 | 1.11e-308 |
| 7 | 1 | 1:159929850:A:T | rs113204365 | 159929850 | 159801111 | 159953521 | 9.57e-10 |
| 8 | 1 | 1:160824430:C:G | rs181431636 | 160824430 | 160238758 | 161023133 | 4.99e-13 |
| 9 | 2 | 2:23661805:G:T | rs188051165 | 23661805 | 23640014 | 23678042 | 4.87e-08 |
| 10 | 3 | 3:84962340:A:T | rs550294817 | 84962340 | 84962340 | 84962340 | 6.56e-10 |
| 11 | 5 | 5:30548772:A:T | rs112123437 | 30548772 | 30548772 | 30548772 | 3.93e-09 |
| 12 | 10 | 10:50641270:C:G | rs1924491 | 50641270 | 50641270 | 50653161 | 4.83e-08 |
| 13 | 11 | 11:126209383:A:G | rs240541 | 126209383 | 126203911 | 126213395 | 4.49e-08 |
| 14 | 12 | 12:71759224:C:T | rs111994982 | 71759224 | 71748427 | 71830684 | 4.06e-08 |
| 15 | 12 | 12:111884608:C:T | rs3184504 | 111884608 | 111373404 | 113218868 | 3.69e-14 |
| 16 | 13 | 13:86910581:A:T | rs28501150 | 86910581 | 86910581 | 86935878 | 1.84e-08 |
| 17 | 17 | 17:6955298:A:G | rs192495138 | 6955298 | 6955298 | 7080316 | 1.45e-16 |
| 18 | 17 | 17:46132937:A:T | rs34750858 | 46132937 | 46132937 | 46283794 | 4.63e-08 |
| 19 | 17 | 17:57955071:A:G | rs1292048 | 57955071 | 57796677 | 58046076 | 1.86e-11 |
| 20 | 19 | 19:32332849:G:T | rs1654833 | 32332849 | 32332849 | 32332849 | 2.79e-09 |
| 21 | 19 | 19:48170435:C:T | rs140068336 | 48170435 | 48099998 | 48203098 | 1.83e-08 |

# **Table S3. Genomic loci associated with sgp130.**

| Locus | uniqID | rsID | chr | pos | start | end | p |
| --- | --- | --- | --- | --- | --- | --- | --- |
| 1 | 3:98402969:C:T | rs11927405 | 3 | 98402969 | 98250862 | 99023224 | 1.09e-64 |
| 2 | 3:155560978:C:T | rs407258 | 3 | 155560978 | 155456723 | 155732590 | 1.64e-09 |
| 3 | 5:52648879:A:G | rs62357767 | 5 | 52648879 | 52538652 | 52648879 | 1.38e-09 |
| 4 | 5:53728808:C:T | rs75607564 | 5 | 53728808 | 53728808 | 53728808 | 3.31e-11 |
| 5 | 5:55282618:C:T | rs6873542 | 5 | 55282618 | 54256812 | 55603036 | 1.67e-308 |
| 6 | 7:12268668:A:C | rs5011432 | 7 | 12268668 | 12233848 | 12285140 | 3.21e-08 |
| 7 | 7:73026378:C:T | rs17145750 | 7 | 73026378 | 72856269 | 73058017 | 2.89e-08 |
| 8 | 9:136155000:C:T | rs635634 | 9 | 136155000 | 136041865 | 136400566 | 6.01e-99 |
| 9 | 11:126234820:C:G | rs78689694 | 11 | 126234820 | 126217870 | 126291021 | 3.86e-36 |
| 10 | 17:7080316:C:T | rs55714927 | 17 | 7080316 | 6809629 | 7264365 | 1.68e-40 |
| 11 | 17:7632621:G:T | rs2642162 | 17 | 7632621 | 7631671 | 7634859 | 2.35e-08 |
| 12 | 20:3681399:C:G | rs4813634 | 20 | 3681399 | 3675333 | 3689420 | 5.56e-09 |
| 13 | 22:39860130:C:T | rs2008174 | 22 | 39860130 | 39738425 | 39860868 | 1.11e-11 |

# **Table S4. Harmonized instrumental cisSNPs (p<5x10^-8^) for CRP to Short Sleep MR.**

| SNP | CHR | POS | A1 | A2 | A1_exp_frq | Beta_exp | SE_exp | N_exp | P_exp | A1_out_frq | Beta_out | SE_out | N_out | P_out | Fstats |
| --- | --- | --- | --- | --- | --- | --- | --- | --- | --- | --- | --- | --- | --- | --- | --- |
| rs10908741 | 1 | 159726123 | T | G | 0.25 | -5.02E-02 | 4.16E-03 | 204402 | 1.64E-33 | 0.24 | 1.09E-03 | 1.12E-03 | 326224 | 3.30E-01 | 145.68 |
| rs10908742 | 1 | 159732811 | G | T | 0.24 | -5.62E-02 | 4.92E-03 | 204402 | 3.50E-30 | 0.24 | 1.15E-03 | 1.19E-03 | 326224 | 3.30E-01 | 130.47 |
| rs11265257 | 1 | 159668984 | T | C | 0.39 | -1.69E-01 | 3.64E-03 | 204402 | 1.00E-300 | 0.39 | -4.69E-04 | 9.85E-04 | 326224 | 6.20E-01 | 2150.79 |
| rs11265260 | 1 | 159700039 | G | A | 0.06 | 2.21E-01 | 7.29E-03 | 204402 | 2.12E-202 | 0.06 | -6.91E-04 | 2.06E-03 | 326224 | 7.50E-01 | 921.76 |
| rs11265263 | 1 | 159710517 | A | C | 0.08 | -2.69E-01 | 8.04E-03 | 204402 | 7.52E-245 | 0.07 | 3.57E-04 | 1.87E-03 | 326224 | 8.90E-01 | 1117.07 |
| rs11588887 | 1 | 159717162 | A | G | 0.14 | -2.04E-01 | 5.81E-03 | 204402 | 5.79E-271 | 0.16 | 4.16E-04 | 1.32E-03 | 326224 | 7.70E-01 | 1237.07 |
| rs11589667 | 1 | 159641029 | T | C | 0.33 | -4.53E-02 | 4.70E-03 | 204402 | 5.18E-22 | 0.34 | -1.02E-03 | 1.03E-03 | 326224 | 3.20E-01 | 93.13 |
| rs11811420 | 1 | 159719872 | C | G | 0.36 | 9.98E-02 | 3.68E-03 | 204402 | 6.49E-162 | 0.35 | -5.86E-04 | 1.01E-03 | 326224 | 5.80E-01 | 735.50 |
| rs12029953 | 1 | 159727739 | A | C | 0.25 | -5.41E-02 | 4.44E-03 | 204402 | 5.18E-34 | 0.24 | 1.09E-03 | 1.12E-03 | 326224 | 3.40E-01 | 147.98 |
| rs12049404 | 1 | 159713844 | T | C | 0.16 | -1.75E-01 | 4.97E-03 | 204402 | 2.55E-270 | 0.16 | 3.93E-04 | 1.32E-03 | 326224 | 7.90E-01 | 1234.47 |
| rs1205 | 1 | 159682233 | T | C | 0.33 | -1.83E-01 | 3.73E-03 | 204402 | 1.00E-300 | 0.33 | 3.88E-04 | 1.02E-03 | 326224 | 7.20E-01 | 2400.50 |
| rs12081252 | 1 | 159706513 | C | T | 0.06 | 2.23E-01 | 7.57E-03 | 204402 | 5.40E-190 | 0.06 | -1.17E-03 | 2.09E-03 | 326224 | 5.80E-01 | 864.74 |
| rs12093699 | 1 | 159647988 | A | G | 0.31 | 1.28E-01 | 3.84E-03 | 204402 | 5.13E-243 | 0.31 | -7.44E-04 | 1.04E-03 | 326224 | 4.90E-01 | 1108.82 |
| rs12094103 | 1 | 159723619 | A | G | 0.36 | 9.96E-02 | 3.71E-03 | 204402 | 5.19E-159 | 0.35 | -6.68E-04 | 1.01E-03 | 326224 | 5.30E-01 | 722.30 |
| rs12567054 | 1 | 159644968 | G | T | 0.07 | 1.54E-01 | 8.26E-03 | 204402 | 1.08E-77 | 0.06 | -1.20E-03 | 1.97E-03 | 326224 | 5.30E-01 | 348.39 |
| rs12727021 | 1 | 159702487 | A | G | 0.31 | 1.40E-01 | 3.80E-03 | 204402 | 1.09E-294 | 0.31 | -2.74E-04 | 1.04E-03 | 326224 | 8.00E-01 | 1346.52 |
| rs12739022 | 1 | 159659431 | C | T | 0.32 | 1.41E-01 | 3.96E-03 | 204402 | 3.72E-278 | 0.31 | -4.17E-04 | 1.04E-03 | 326224 | 7.00E-01 | 1270.61 |
| rs12754915 | 1 | 159660869 | C | T | 0.31 | 1.44E-01 | 3.87E-03 | 204402 | 1.00E-300 | 0.31 | -3.95E-04 | 1.04E-03 | 326224 | 7.20E-01 | 1377.50 |
| rs1341665 | 1 | 159691559 | A | G | 0.33 | -1.81E-01 | 3.85E-03 | 204402 | 1.00E-300 | 0.34 | 4.07E-04 | 1.01E-03 | 326224 | 7.10E-01 | 2221.42 |
| rs1470515 | 1 | 159653599 | T | C | 0.39 | -1.68E-01 | 3.72E-03 | 204402 | 1.00E-300 | 0.39 | -2.29E-04 | 9.87E-04 | 326224 | 8.00E-01 | 2033.30 |
| rs1572970 | 1 | 159673585 | A | G | 0.70 | -4.39E-02 | 3.97E-03 | 204402 | 2.06E-28 | 0.70 | -1.06E-03 | 1.05E-03 | 326224 | 3.10E-01 | 122.39 |
| rs16842484 | 1 | 159646924 | C | T | 0.26 | 1.20E-01 | 4.18E-03 | 204402 | 6.84E-181 | 0.26 | -4.81E-04 | 1.10E-03 | 326224 | 6.90E-01 | 822.83 |
| rs16842599 | 1 | 159697475 | C | T | 0.06 | 2.23E-01 | 7.54E-03 | 204402 | 2.82E-192 | 0.06 | -1.43E-03 | 2.08E-03 | 326224 | 5.00E-01 | 875.17 |
| rs1811472 | 1 | 159642349 | C | G | 0.41 | -1.21E-01 | 3.89E-03 | 204402 | 2.11E-213 | 0.40 | 5.63E-04 | 9.89E-04 | 326224 | 5.70E-01 | 972.20 |
| rs1971863 | 1 | 159638931 | C | T | 0.26 | 9.03E-02 | 4.15E-03 | 204402 | 7.94E-105 | 0.25 | -1.61E-03 | 1.11E-03 | 326224 | 1.50E-01 | 473.01 |
| rs2027469 | 1 | 159667190 | A | G | 0.18 | -8.41E-02 | 4.89E-03 | 204402 | 3.21E-66 | 0.19 | -6.12E-04 | 1.22E-03 | 326224 | 6.10E-01 | 295.73 |
| rs2794498 | 1 | 159636116 | T | G | 0.26 | 8.71E-02 | 4.05E-03 | 204402 | 8.34E-103 | 0.25 | -1.62E-03 | 1.11E-03 | 326224 | 1.50E-01 | 463.87 |
| rs2794500 | 1 | 159635021 | T | C | 0.36 | 7.03E-02 | 3.69E-03 | 204402 | 9.98E-81 | 0.35 | -1.66E-03 | 1.01E-03 | 326224 | 9.30E-02 | 362.33 |
| rs2794520 | 1 | 159678816 | T | C | 0.33 | -1.82E-01 | 3.71E-03 | 204402 | 1.00E-300 | 0.33 | 4.97E-04 | 1.02E-03 | 326224 | 6.50E-01 | 2408.87 |
| rs2808628 | 1 | 159676011 | A | G | 0.33 | -1.82E-01 | 3.86E-03 | 204402 | 1.00E-300 | 0.33 | 4.68E-04 | 1.02E-03 | 326224 | 6.70E-01 | 2238.23 |
| rs3093075 | 1 | 159679913 | T | G | 0.06 | 2.26E-01 | 7.39E-03 | 204402 | 2.02E-204 | 0.06 | -1.74E-03 | 2.08E-03 | 326224 | 4.10E-01 | 931.16 |
| rs4131568 | 1 | 159722056 | T | C | 0.36 | 9.70E-02 | 3.82E-03 | 204402 | 4.89E-142 | 0.34 | -4.38E-04 | 1.01E-03 | 326224 | 6.90E-01 | 644.29 |
| rs4255379 | 1 | 159718312 | A | G | 0.63 | 6.39E-02 | 3.82E-03 | 204402 | 1.35E-62 | 0.61 | -7.98E-05 | 9.83E-04 | 326224 | 9.50E-01 | 279.05 |
| rs4512645 | 1 | 159729047 | A | G | 0.35 | 9.95E-02 | 3.93E-03 | 204402 | 8.22E-142 | 0.34 | -5.84E-04 | 1.02E-03 | 326224 | 5.80E-01 | 643.16 |
| rs7553007 | 1 | 159698549 | A | G | 0.32 | -1.82E-01 | 3.86E-03 | 204402 | 1.00E-300 | 0.33 | 3.89E-04 | 1.02E-03 | 326224 | 7.30E-01 | 2220.29 |
| rs876537 | 1 | 159674933 | T | C | 0.38 | -1.70E-01 | 3.67E-03 | 204402 | 1.00E-300 | 0.39 | -4.90E-04 | 9.83E-04 | 326224 | 6.00E-01 | 2137.45 |
| rs895582 | 1 | 159637919 | A | G | 0.26 | 8.99E-02 | 4.09E-03 | 204402 | 3.79E-107 | 0.25 | -1.75E-03 | 1.11E-03 | 326224 | 1.20E-01 | 483.62 |

Instrumental cis SNPs (50K) select for CRP to Sleep classical Mendelian randomization analysis. CHR: chromosome; POS: hg19 genomic position; A1: effective allele; A2: the other allele; A1_exp_frq: A1 frequency in the exposure dataset; Beta_exp, effect of A1 for the exposure; SE_exp; standard error for Beta_exp; N_exp: sample size for exposure; P_exp, association p value for exposure; A1_out_frq: A1 frequency in the outcome dataset; Beta_out: effect size of A1 on outcome; SE_out: standard error of Beta_out; N_out: sample size for outcome; P_out: association p value for outcome; F-stats: F-statistics.

# **Table S5. Harmonized instrumental cisSNPs (p<5x10^-8^) for CRP to Long Sleep MR.**

| SNP | CHR | POS | A1 | A2 | A1_exp_frq | Beta_exp | SE_exp | N_exp | P_exp | A1_out_frq | Beta_out | SE_out | N_out | P_out | Fstats |
| --- | --- | --- | --- | --- | --- | --- | --- | --- | --- | --- | --- | --- | --- | --- | --- |
| rs10908741 | 1 | 159726123 | T | G | 0.25 | -5.02E-02 | 4.16E-03 | 204402 | 1.64E-33 | 0.24 | 1.56E-05 | 8.51E-04 | 326224 | 9.70E-01 | 145.68 |
| rs10908742 | 1 | 159732811 | G | T | 0.24 | -5.62E-02 | 4.92E-03 | 204402 | 3.50E-30 | 0.24 | -5.87E-04 | 9.00E-04 | 326224 | 5.20E-01 | 130.47 |
| rs11265257 | 1 | 159668984 | T | C | 0.39 | -1.69E-01 | 3.64E-03 | 204402 | 1.00E-300 | 0.39 | 1.30E-03 | 7.46E-04 | 326224 | 7.80E-02 | 2150.79 |
| rs11265260 | 1 | 159700039 | G | A | 0.06 | 2.21E-01 | 7.29E-03 | 204402 | 2.12E-202 | 0.06 | -1.02E-03 | 1.56E-03 | 326224 | 5.00E-01 | 921.76 |
| rs11265263 | 1 | 159710517 | A | C | 0.08 | -2.69E-01 | 8.04E-03 | 204402 | 7.52E-245 | 0.07 | 1.75E-05 | 1.42E-03 | 326224 | 9.80E-01 | 1117.07 |
| rs11588887 | 1 | 159717162 | A | G | 0.14 | -2.04E-01 | 5.81E-03 | 204402 | 5.79E-271 | 0.16 | 1.16E-03 | 1.00E-03 | 326224 | 2.40E-01 | 1237.07 |
| rs11589667 | 1 | 159641029 | T | C | 0.33 | -4.53E-02 | 4.70E-03 | 204402 | 5.18E-22 | 0.34 | -3.96E-04 | 7.76E-04 | 326224 | 6.00E-01 | 93.13 |
| rs11811420 | 1 | 159719872 | C | G | 0.36 | 9.98E-02 | 3.68E-03 | 204402 | 6.49E-162 | 0.35 | -5.42E-05 | 7.62E-04 | 326224 | 9.20E-01 | 735.50 |
| rs12029953 | 1 | 159727739 | A | C | 0.25 | -5.41E-02 | 4.44E-03 | 204402 | 5.18E-34 | 0.24 | -3.69E-05 | 8.52E-04 | 326224 | 9.80E-01 | 147.98 |
| rs12049404 | 1 | 159713844 | T | C | 0.16 | -1.75E-01 | 4.97E-03 | 204402 | 2.55E-270 | 0.16 | 1.13E-03 | 9.99E-04 | 326224 | 2.50E-01 | 1234.47 |
| rs1205 | 1 | 159682233 | T | C | 0.33 | -1.83E-01 | 3.73E-03 | 204402 | 1.00E-300 | 0.33 | 1.37E-03 | 7.71E-04 | 326224 | 7.10E-02 | 2400.50 |
| rs12081252 | 1 | 159706513 | C | T | 0.06 | 2.23E-01 | 7.57E-03 | 204402 | 5.40E-190 | 0.06 | -9.91E-04 | 1.58E-03 | 326224 | 5.20E-01 | 864.74 |
| rs12093699 | 1 | 159647988 | A | G | 0.31 | 1.28E-01 | 3.84E-03 | 204402 | 5.13E-243 | 0.31 | -5.40E-04 | 7.87E-04 | 326224 | 4.80E-01 | 1108.82 |
| rs12094103 | 1 | 159723619 | A | G | 0.36 | 9.96E-02 | 3.71E-03 | 204402 | 5.19E-159 | 0.35 | -1.55E-04 | 7.62E-04 | 326224 | 8.20E-01 | 722.30 |
| rs12567054 | 1 | 159644968 | G | T | 0.07 | 1.54E-01 | 8.26E-03 | 204402 | 1.08E-77 | 0.06 | -1.21E-03 | 1.50E-03 | 326224 | 4.10E-01 | 348.39 |
| rs12727021 | 1 | 159702487 | A | G | 0.31 | 1.40E-01 | 3.80E-03 | 204402 | 1.09E-294 | 0.31 | -2.14E-04 | 7.87E-04 | 326224 | 7.60E-01 | 1346.52 |
| rs12739022 | 1 | 159659431 | C | T | 0.32 | 1.41E-01 | 3.96E-03 | 204402 | 3.72E-278 | 0.31 | -3.14E-04 | 7.89E-04 | 326224 | 6.70E-01 | 1270.61 |
| rs12754915 | 1 | 159660869 | C | T | 0.31 | 1.44E-01 | 3.87E-03 | 204402 | 1.00E-300 | 0.31 | -2.77E-04 | 7.89E-04 | 326224 | 7.00E-01 | 1377.50 |
| rs1341665 | 1 | 159691559 | A | G | 0.33 | -1.81E-01 | 3.85E-03 | 204402 | 1.00E-300 | 0.34 | 1.20E-03 | 7.66E-04 | 326224 | 1.10E-01 | 2221.42 |
| rs1470515 | 1 | 159653599 | T | C | 0.39 | -1.68E-01 | 3.72E-03 | 204402 | 1.00E-300 | 0.39 | 1.30E-03 | 7.47E-04 | 326224 | 7.70E-02 | 2033.30 |
| rs1572970 | 1 | 159673585 | A | G | 0.70 | -4.39E-02 | 3.97E-03 | 204402 | 2.06E-28 | 0.70 | 1.05E-03 | 7.97E-04 | 326224 | 1.90E-01 | 122.39 |
| rs16842484 | 1 | 159646924 | C | T | 0.26 | 1.20E-01 | 4.18E-03 | 204402 | 6.84E-181 | 0.26 | -1.92E-04 | 8.34E-04 | 326224 | 8.00E-01 | 822.83 |
| rs16842599 | 1 | 159697475 | C | T | 0.06 | 2.23E-01 | 7.54E-03 | 204402 | 2.82E-192 | 0.06 | -9.65E-04 | 1.58E-03 | 326224 | 5.30E-01 | 875.17 |
| rs1811472 | 1 | 159642349 | C | G | 0.41 | -1.21E-01 | 3.89E-03 | 204402 | 2.11E-213 | 0.40 | 6.48E-04 | 7.49E-04 | 326224 | 3.80E-01 | 972.20 |
| rs1971863 | 1 | 159638931 | C | T | 0.26 | 9.03E-02 | 4.15E-03 | 204402 | 7.94E-105 | 0.25 | -5.04E-04 | 8.42E-04 | 326224 | 5.40E-01 | 473.01 |
| rs2027469 | 1 | 159667190 | A | G | 0.18 | -8.41E-02 | 4.89E-03 | 204402 | 3.21E-66 | 0.19 | 7.83E-04 | 9.27E-04 | 326224 | 4.00E-01 | 295.73 |
| rs2794498 | 1 | 159636116 | T | G | 0.26 | 8.71E-02 | 4.05E-03 | 204402 | 8.34E-103 | 0.25 | -5.23E-04 | 8.39E-04 | 326224 | 5.30E-01 | 463.87 |
| rs2794500 | 1 | 159635021 | T | C | 0.36 | 7.03E-02 | 3.69E-03 | 204402 | 9.98E-81 | 0.35 | -1.09E-03 | 7.61E-04 | 326224 | 1.50E-01 | 362.33 |
| rs2794520 | 1 | 159678816 | T | C | 0.33 | -1.82E-01 | 3.71E-03 | 204402 | 1.00E-300 | 0.33 | 1.36E-03 | 7.70E-04 | 326224 | 7.10E-02 | 2408.87 |
| rs2808628 | 1 | 159676011 | A | G | 0.33 | -1.82E-01 | 3.86E-03 | 204402 | 1.00E-300 | 0.33 | 1.33E-03 | 7.71E-04 | 326224 | 7.90E-02 | 2238.23 |
| rs3093075 | 1 | 159679913 | T | G | 0.06 | 2.26E-01 | 7.39E-03 | 204402 | 2.02E-204 | 0.06 | -1.20E-03 | 1.58E-03 | 326224 | 4.40E-01 | 931.16 |
| rs4131568 | 1 | 159722056 | T | C | 0.36 | 9.70E-02 | 3.82E-03 | 204402 | 4.89E-142 | 0.34 | -1.22E-04 | 7.68E-04 | 326224 | 8.50E-01 | 644.29 |
| rs4255379 | 1 | 159718312 | A | G | 0.63 | 6.39E-02 | 3.82E-03 | 204402 | 1.35E-62 | 0.61 | -1.99E-04 | 7.45E-04 | 326224 | 7.80E-01 | 279.05 |
| rs4512645 | 1 | 159729047 | A | G | 0.35 | 9.95E-02 | 3.93E-03 | 204402 | 8.22E-142 | 0.34 | -1.95E-04 | 7.69E-04 | 326224 | 7.80E-01 | 643.16 |
| rs7553007 | 1 | 159698549 | A | G | 0.32 | -1.82E-01 | 3.86E-03 | 204402 | 1.00E-300 | 0.33 | 1.27E-03 | 7.71E-04 | 326224 | 9.40E-02 | 2220.29 |
| rs876537 | 1 | 159674933 | T | C | 0.38 | -1.70E-01 | 3.67E-03 | 204402 | 1.00E-300 | 0.39 | 1.27E-03 | 7.45E-04 | 326224 | 8.40E-02 | 2137.45 |
| rs895582 | 1 | 159637919 | A | G | 0.26 | 8.99E-02 | 4.09E-03 | 204402 | 3.79E-107 | 0.25 | -5.64E-04 | 8.39E-04 | 326224 | 5.00E-01 | 483.62 |

Instrumental cis SNPs (50K) select for CRP to Sleep classical Mendelian randomization analysis. CHR: chromosome; POS: hg19 genomic position; A1: effective allele; A2: the other allele; A1_exp_frq: A1 frequency in the exposure dataset; Beta_exp, effect of A1 for the exposure; SE_exp; standard error for Beta_exp; N_exp: sample size for exposure; P_exp, association p value for exposure; A1_out_frq: A1 frequency in the outcome dataset; Beta_out: effect size of A1 on outcome; SE_out: standard error of Beta_out; N_out: sample size for outcome; P_out: association p value for outcome; F-stats: F-statistics.

# **Table S6. Harmonized instrumental cisSNPs (p<5x10^-8^) for CRP to Sleep Duration MR.**

| SNP | CHR | POS | A1 | A2 | A1_exp_frq | Beta_exp | SE_exp | N_exp | P_exp | A1_out_frq | Beta_out | SE_out | N_out | P_out | Fstats |
| --- | --- | --- | --- | --- | --- | --- | --- | --- | --- | --- | --- | --- | --- | --- | --- |
| rs10908741 | 1 | 1.6E+08 | T | G | 0.25 | -5.02E-02 | 4.16E-03 | 204402 | 1.64E-33 | 0.24 | -6.98E-04 | 2.65E-03 | 326224 | 7.80E-01 | 145.68 |
| rs10908742 | 1 | 1.6E+08 | G | T | 0.24 | -5.62E-02 | 4.92E-03 | 204402 | 3.50E-30 | 0.24 | -2.87E-03 | 2.80E-03 | 326224 | 2.90E-01 | 130.47 |
| rs11265257 | 1 | 1.6E+08 | T | C | 0.39 | -1.69E-01 | 3.64E-03 | 204402 | 1.00E-300 | 0.39 | 2.94E-03 | 2.32E-03 | 326224 | 2.10E-01 | 2150.79 |
| rs11265260 | 1 | 1.6E+08 | G | A | 0.06 | 2.21E-01 | 7.29E-03 | 204402 | 2.12E-202 | 0.06 | 1.84E-03 | 4.87E-03 | 326224 | 7.60E-01 | 921.76 |
| rs11265263 | 1 | 1.6E+08 | A | C | 0.08 | -2.69E-01 | 8.04E-03 | 204402 | 7.52E-245 | 0.07 | -1.99E-03 | 4.42E-03 | 326224 | 7.00E-01 | 1117.07 |
| rs11588887 | 1 | 1.6E+08 | A | G | 0.14 | -2.04E-01 | 5.81E-03 | 204402 | 5.79E-271 | 0.16 | 1.98E-03 | 3.12E-03 | 326224 | 5.20E-01 | 1237.07 |
| rs11589667 | 1 | 1.6E+08 | T | C | 0.33 | -4.53E-02 | 4.70E-03 | 204402 | 5.18E-22 | 0.34 | 1.30E-03 | 2.42E-03 | 326224 | 6.20E-01 | 93.13 |
| rs11811420 | 1 | 1.6E+08 | C | G | 0.36 | 9.98E-02 | 3.68E-03 | 204402 | 6.49E-162 | 0.35 | -3.93E-04 | 2.38E-03 | 326224 | 8.70E-01 | 735.5 |
| rs12029953 | 1 | 1.6E+08 | A | C | 0.25 | -5.41E-02 | 4.44E-03 | 204402 | 5.18E-34 | 0.24 | -7.15E-04 | 2.65E-03 | 326224 | 7.70E-01 | 147.98 |
| rs12049404 | 1 | 1.6E+08 | T | C | 0.16 | -1.75E-01 | 4.97E-03 | 204402 | 2.55E-270 | 0.16 | 1.97E-03 | 3.12E-03 | 326224 | 5.20E-01 | 1234.47 |
| rs1205 | 1 | 1.6E+08 | T | C | 0.33 | -1.83E-01 | 3.73E-03 | 204402 | 1.00E-300 | 0.33 | 8.86E-04 | 2.40E-03 | 326224 | 6.90E-01 | 2400.5 |
| rs12081252 | 1 | 1.6E+08 | C | T | 0.06 | 2.23E-01 | 7.57E-03 | 204402 | 5.40E-190 | 0.06 | 2.92E-03 | 4.93E-03 | 326224 | 6.00E-01 | 864.74 |
| rs12093699 | 1 | 1.6E+08 | A | G | 0.31 | 1.28E-01 | 3.84E-03 | 204402 | 5.13E-243 | 0.31 | -3.46E-05 | 2.45E-03 | 326224 | 9.70E-01 | 1108.82 |
| rs12094103 | 1 | 1.6E+08 | A | G | 0.36 | 9.96E-02 | 3.71E-03 | 204402 | 5.19E-159 | 0.35 | -4.32E-04 | 2.38E-03 | 326224 | 8.60E-01 | 722.3 |
| rs12567054 | 1 | 1.6E+08 | G | T | 0.07 | 1.54E-01 | 8.26E-03 | 204402 | 1.08E-77 | 0.06 | 8.44E-04 | 4.66E-03 | 326224 | 8.80E-01 | 348.39 |
| rs12727021 | 1 | 1.6E+08 | A | G | 0.31 | 1.40E-01 | 3.80E-03 | 204402 | 1.09E-294 | 0.31 | 6.54E-05 | 2.45E-03 | 326224 | 9.80E-01 | 1346.52 |
| rs12739022 | 1 | 1.6E+08 | C | T | 0.32 | 1.41E-01 | 3.96E-03 | 204402 | 3.72E-278 | 0.31 | 1.92E-04 | 2.46E-03 | 326224 | 9.40E-01 | 1270.61 |
| rs12754915 | 1 | 1.6E+08 | C | T | 0.31 | 1.44E-01 | 3.87E-03 | 204402 | 1.00E-300 | 0.31 | 2.31E-04 | 2.46E-03 | 326224 | 9.30E-01 | 1377.5 |
| rs1341665 | 1 | 1.6E+08 | A | G | 0.33 | -1.81E-01 | 3.85E-03 | 204402 | 1.00E-300 | 0.34 | 2.48E-04 | 2.39E-03 | 326224 | 8.90E-01 | 2221.42 |
| rs1470515 | 1 | 1.6E+08 | T | C | 0.39 | -1.68E-01 | 3.72E-03 | 204402 | 1.00E-300 | 0.39 | 2.66E-03 | 2.33E-03 | 326224 | 2.60E-01 | 2033.3 |
| rs1572970 | 1 | 1.6E+08 | A | G | 0.7 | -4.39E-02 | 3.97E-03 | 204402 | 2.06E-28 | 0.7 | 3.45E-03 | 2.48E-03 | 326224 | 1.70E-01 | 122.39 |
| rs16842484 | 1 | 1.6E+08 | C | T | 0.26 | 1.20E-01 | 4.18E-03 | 204402 | 6.84E-181 | 0.26 | 2.93E-04 | 2.60E-03 | 326224 | 9.50E-01 | 822.83 |
| rs16842599 | 1 | 1.6E+08 | C | T | 0.06 | 2.23E-01 | 7.54E-03 | 204402 | 2.82E-192 | 0.06 | 3.16E-03 | 4.92E-03 | 326224 | 5.70E-01 | 875.17 |
| rs1811472 | 1 | 1.6E+08 | C | G | 0.41 | -1.21E-01 | 3.89E-03 | 204402 | 2.11E-213 | 0.4 | 4.14E-04 | 2.33E-03 | 326224 | 8.60E-01 | 972.2 |
| rs1971863 | 1 | 1.6E+08 | C | T | 0.26 | 9.03E-02 | 4.15E-03 | 204402 | 7.94E-105 | 0.25 | -9.76E-04 | 2.63E-03 | 326224 | 7.10E-01 | 473.01 |
| rs2027469 | 1 | 1.6E+08 | A | G | 0.18 | -8.41E-02 | 4.89E-03 | 204402 | 3.21E-66 | 0.19 | 3.26E-03 | 2.89E-03 | 326224 | 2.70E-01 | 295.73 |
| rs2794498 | 1 | 1.6E+08 | T | G | 0.26 | 8.71E-02 | 4.05E-03 | 204402 | 8.34E-103 | 0.25 | -9.67E-04 | 2.62E-03 | 326224 | 7.10E-01 | 463.87 |
| rs2794500 | 1 | 1.6E+08 | T | C | 0.36 | 7.03E-02 | 3.69E-03 | 204402 | 9.98E-81 | 0.35 | -1.19E-03 | 2.37E-03 | 326224 | 6.30E-01 | 362.33 |
| rs2794520 | 1 | 1.6E+08 | T | C | 0.33 | -1.82E-01 | 3.71E-03 | 204402 | 1.00E-300 | 0.33 | 6.60E-04 | 2.40E-03 | 326224 | 7.60E-01 | 2408.87 |
| rs2808628 | 1 | 1.6E+08 | A | G | 0.33 | -1.82E-01 | 3.86E-03 | 204402 | 1.00E-300 | 0.33 | 6.72E-04 | 2.40E-03 | 326224 | 7.60E-01 | 2238.23 |
| rs3093075 | 1 | 1.6E+08 | T | G | 0.06 | 2.26E-01 | 7.39E-03 | 204402 | 2.02E-204 | 0.06 | 2.57E-03 | 4.92E-03 | 326224 | 6.40E-01 | 931.16 |
| rs4131568 | 1 | 1.6E+08 | T | C | 0.36 | 9.70E-02 | 3.82E-03 | 204402 | 4.89E-142 | 0.34 | -7.59E-04 | 2.39E-03 | 326224 | 7.50E-01 | 644.29 |
| rs4255379 | 1 | 1.6E+08 | A | G | 0.63 | 6.39E-02 | 3.82E-03 | 204402 | 1.35E-62 | 0.61 | -1.13E-04 | 2.32E-03 | 326224 | 9.60E-01 | 279.05 |
| rs4512645 | 1 | 1.6E+08 | A | G | 0.35 | 9.95E-02 | 3.93E-03 | 204402 | 8.22E-142 | 0.34 | -5.75E-04 | 2.40E-03 | 326224 | 8.10E-01 | 643.16 |
| rs7553007 | 1 | 1.6E+08 | A | G | 0.32 | -1.82E-01 | 3.86E-03 | 204402 | 1.00E-300 | 0.33 | 5.46E-04 | 2.40E-03 | 326224 | 7.90E-01 | 2220.29 |
| rs876537 | 1 | 1.6E+08 | T | C | 0.38 | -1.70E-01 | 3.67E-03 | 204402 | 1.00E-300 | 0.39 | 2.95E-03 | 2.32E-03 | 326224 | 2.10E-01 | 2137.45 |
| rs895582 | 1 | 1.6E+08 | A | G | 0.26 | 8.99E-02 | 4.09E-03 | 204402 | 3.79E-107 | 0.25 | -8.36E-04 | 2.62E-03 | 326224 | 7.50E-01 | 483.62 |

Instrumental cis SNPs (50K) select for CRP to Sleep classical Mendelian randomization analysis. CHR: chromosome; POS: hg19 genomic position; A1: effective allele; A2: the other allele; A1_exp_frq: A1 frequency in the exposure dataset; Beta_exp, effect of A1 for the exposure; SE_exp; standard error for Beta_exp; N_exp: sample size for exposure; P_exp, association p value for exposure; A1_out_frq: A1 frequency in the outcome dataset; Beta_out: effect size of A1 on outcome; SE_out: standard error of Beta_out; N_out: sample size for outcome; P_out: association p value for outcome; F-stats: F-statistics.

# **Table S7. Harmonized instrumental cisSNPs (p<5x10^-8^) for sIL6R to Short Sleep MR.**

| SNP | CHR | POS | A1 | A2 | A1_exp_frq | Beta_exp | SE_exp | N_exp | P_exp | A1_out_frq | Beta_out | SE_out | N_out | P_out | Fstats |
| --- | --- | --- | --- | --- | --- | --- | --- | --- | --- | --- | --- | --- | --- | --- | --- |
| rs10047079 | 1 | 154468135 | T | C | 0.77 | 5.88E-01 | 5.40E-03 | 69970 | 1.00E-300 | 0.81 | -2.22E-03 | 1.21E-03 | 326224 | 7.20E-02 | 11836.63 |
| rs1073907 | 1 | 154332520 | T | C | 0.30 | 1.53E-01 | 8.10E-03 | 35571 | 3.20E-79 | 0.30 | 8.54E-04 | 1.04E-03 | 326224 | 4.30E-01 | 357.26 |
| rs10908839 | 1 | 154430798 | C | G | 0.58 | -6.11E-01 | 5.20E-03 | 70935 | 1.00E-300 | 0.78 | 1.67E-04 | 1.16E-03 | 326224 | 8.70E-01 | 13801.73 |
| rs10908840 | 1 | 154459477 | T | C | 0.44 | 1.21E-01 | 5.70E-03 | 70930 | 2.23E-101 | 0.48 | 2.01E-04 | 9.60E-04 | 326224 | 8.70E-01 | 449.89 |
| rs111885536 | 1 | 154461260 | A | G | 0.19 | -2.11E-01 | 7.00E-03 | 70935 | 1.26E-202 | 0.16 | -3.00E-03 | 1.30E-03 | 326224 | 2.10E-02 | 910.32 |
| rs11265606 | 1 | 154356459 | T | C | 0.28 | 1.80E-01 | 6.20E-03 | 70935 | 4.84E-186 | 0.29 | 9.90E-04 | 1.06E-03 | 326224 | 3.60E-01 | 844.75 |
| rs11265607 | 1 | 154357678 | A | G | 0.68 | -1.83E-01 | 6.20E-03 | 70935 | 3.48E-191 | 0.71 | -9.23E-04 | 1.06E-03 | 326224 | 3.90E-01 | 869.30 |
| rs11265621 | 1 | 154442960 | A | G | 0.46 | 5.41E-01 | 4.30E-03 | 70935 | 1.00E-300 | 0.64 | 1.30E-04 | 1.00E-03 | 326224 | 8.70E-01 | 15811.60 |
| rs11265622 | 1 | 154451420 | A | G | 0.53 | -5.37E-01 | 4.30E-03 | 69970 | 1.00E-300 | 0.36 | -2.06E-04 | 1.00E-03 | 326224 | 8.10E-01 | 15578.52 |
| rs113624284 | 1 | 154339299 | A | G | 0.79 | -2.60E-01 | 7.80E-03 | 70935 | 1.49E-242 | 0.85 | -7.18E-04 | 1.35E-03 | 326224 | 6.00E-01 | 1108.55 |
| rs11576181 | 1 | 154330659 | T | G | 0.67 | -1.23E-01 | 6.10E-03 | 70935 | 1.70E-89 | 0.70 | -9.47E-04 | 1.04E-03 | 326224 | 3.80E-01 | 405.26 |
| rs11580535 | 1 | 154364317 | T | G | 0.13 | 3.55E-01 | 8.00E-03 | 70935 | 1.00E-300 | 0.14 | 6.32E-04 | 1.39E-03 | 326224 | 6.40E-01 | 1965.81 |
| rs11582433 | 1 | 154349605 | T | C | 0.14 | 2.70E-01 | 7.70E-03 | 70935 | 4.44E-269 | 0.15 | 9.61E-04 | 1.33E-03 | 326224 | 4.70E-01 | 1233.20 |
| rs11590203 | 1 | 154333569 | T | G | 0.14 | 2.50E-01 | 7.90E-03 | 69970 | 2.44E-222 | 0.15 | 6.96E-04 | 1.35E-03 | 326224 | 6.00E-01 | 1002.24 |
| rs116088025 | 1 | 154354350 | T | C | 0.14 | 2.73E-01 | 7.70E-03 | 70935 | 3.37E-273 | 0.15 | 1.02E-03 | 1.33E-03 | 326224 | 4.50E-01 | 1259.79 |
| rs12023772 | 1 | 154483868 | A | G | 0.17 | 7.12E-01 | 5.50E-03 | 70935 | 1.00E-300 | 0.16 | 1.64E-04 | 1.31E-03 | 326224 | 8.30E-01 | 16749.07 |
| rs12025518 | 1 | 154340789 | A | C | 0.69 | 1.92E-01 | 6.30E-03 | 69969 | 1.00E-200 | 0.74 | -4.73E-04 | 1.09E-03 | 326224 | 7.10E-01 | 926.86 |
| rs12033701 | 1 | 154365886 | T | C | 0.11 | -1.66E-01 | 8.70E-03 | 70934 | 1.23E-81 | 0.10 | -1.36E-03 | 1.57E-03 | 326224 | 3.70E-01 | 364.06 |
| rs12044132 | 1 | 154462360 | T | C | 0.15 | 6.85E-01 | 9.60E-03 | 34606 | 1.00E-300 | 0.16 | 1.15E-05 | 1.31E-03 | 326224 | 9.20E-01 | 5086.96 |
| rs12061599 | 1 | 154344135 | T | G | 0.28 | 1.29E-01 | 6.20E-03 | 70935 | 5.38E-98 | 0.30 | 9.62E-04 | 1.04E-03 | 326224 | 3.70E-01 | 434.92 |
| rs12075836 | 1 | 154371487 | T | C | 0.14 | -2.62E-01 | 7.70E-03 | 70935 | 8.28E-252 | 0.15 | 1.81E-03 | 1.36E-03 | 326224 | 1.90E-01 | 1158.65 |
| rs12118018 | 1 | 154477440 | A | G | 0.46 | 5.36E-01 | 4.30E-03 | 70935 | 1.00E-300 | 0.64 | 1.82E-04 | 1.00E-03 | 326224 | 8.30E-01 | 15555.31 |
| rs12118721 | 1 | 154397416 | T | C | 0.55 | -8.09E-01 | 3.90E-03 | 70934 | 1.00E-300 | 0.43 | 9.98E-04 | 9.76E-04 | 326224 | 3.40E-01 | 42976.48 |
| rs12119111 | 1 | 154478600 | A | G | 0.46 | 5.37E-01 | 4.30E-03 | 70935 | 1.00E-300 | 0.64 | 2.03E-04 | 1.00E-03 | 326224 | 8.20E-01 | 15578.52 |
| rs12129500 | 1 | 154423764 | T | C | 0.56 | -8.43E-01 | 3.90E-03 | 70935 | 1.00E-300 | 0.42 | 9.05E-04 | 9.70E-04 | 326224 | 3.90E-01 | 46711.40 |
| rs12133641 | 1 | 154428283 | A | G | 0.57 | -1.01E+00 | 3.40E-03 | 70935 | 1.00E-300 | 0.59 | -2.17E-04 | 9.73E-04 | 326224 | 7.60E-01 | 88401.28 |
| rs12563459 | 1 | 154362686 | A | G | 0.15 | -1.85E-01 | 7.60E-03 | 70935 | 1.95E-130 | 0.15 | 1.42E-03 | 1.33E-03 | 326224 | 3.10E-01 | 594.46 |
| rs12568083 | 1 | 154455949 | T | C | 0.39 | -5.02E-01 | 7.20E-03 | 34606 | 1.00E-300 | 0.36 | -1.89E-04 | 1.00E-03 | 326224 | 8.30E-01 | 4853.44 |
| rs12739228 | 1 | 154426190 | A | G | 0.04 | -6.32E-01 | 1.59E-02 | 68557 | 1.00E-300 | 0.04 | 4.69E-04 | 2.54E-03 | 326224 | 8.80E-01 | 1578.44 |
| rs12753254 | 1 | 154416935 | A | G | 0.41 | 9.76E-01 | 3.50E-03 | 70935 | 1.00E-300 | 0.42 | 4.00E-04 | 9.72E-04 | 326224 | 6.30E-01 | 77745.37 |
| rs12753666 | 1 | 154474875 | A | G | 0.46 | 5.36E-01 | 4.30E-03 | 70935 | 1.00E-300 | 0.64 | 1.97E-04 | 1.00E-03 | 326224 | 8.20E-01 | 15537.91 |
| rs138765671 | 1 | 154345962 | A | C | 0.14 | 2.61E-01 | 7.80E-03 | 70935 | 2.75E-243 | 0.15 | 7.87E-04 | 1.35E-03 | 326224 | 5.60E-01 | 1123.11 |
| rs147763778 | 1 | 154368223 | A | G | 0.04 | 3.99E-01 | 1.60E-02 | 68557 | 3.27E-138 | 0.04 | -1.57E-03 | 2.47E-03 | 326224 | 5.30E-01 | 623.13 |
| rs1889313 | 1 | 154351717 | A | C | 0.14 | 2.70E-01 | 7.70E-03 | 70935 | 6.23E-269 | 0.15 | 9.55E-04 | 1.33E-03 | 326224 | 4.70E-01 | 1233.20 |
| rs2229238 | 1 | 154437896 | T | C | 0.58 | -5.86E-01 | 5.50E-03 | 69970 | 1.00E-300 | 0.18 | 2.13E-03 | 1.23E-03 | 326224 | 8.60E-02 | 11336.44 |
| rs34094138 | 1 | 154366911 | T | G | 0.78 | 1.86E-01 | 7.60E-03 | 70935 | 1.20E-131 | 0.85 | -1.50E-03 | 1.33E-03 | 326224 | 2.80E-01 | 597.03 |
| rs35717427 | 1 | 154391882 | A | G | 0.12 | 4.99E-01 | 8.30E-03 | 69969 | 1.00E-300 | 0.12 | -4.11E-05 | 1.46E-03 | 326224 | 9.90E-01 | 3607.23 |
| rs4240872 | 1 | 154436195 | T | C | 0.39 | 6.19E-01 | 5.00E-03 | 70935 | 1.00E-300 | 0.77 | -1.86E-04 | 1.15E-03 | 326224 | 8.90E-01 | 15331.39 |
| rs4393147 | 1 | 154414037 | T | C | 0.41 | 9.75E-01 | 3.50E-03 | 70935 | 1.00E-300 | 0.42 | 4.52E-04 | 9.73E-04 | 326224 | 5.90E-01 | 77554.29 |
| rs4474240 | 1 | 154457855 | A | C | 0.47 | -2.14E-01 | 7.00E-03 | 69970 | 5.07E-206 | 0.16 | -2.90E-03 | 1.30E-03 | 326224 | 2.50E-02 | 933.74 |
| rs4478801 | 1 | 154464572 | A | G | 0.58 | 5.01E-01 | 7.20E-03 | 34598 | 1.00E-300 | 0.64 | 1.96E-04 | 1.00E-03 | 326224 | 8.20E-01 | 4843.77 |
| rs4521987 | 1 | 154388668 | T | C | 0.45 | -2.73E-01 | 7.90E-03 | 69969 | 8.69E-265 | 0.15 | 1.67E-03 | 1.37E-03 | 326224 | 2.40E-01 | 1196.81 |
| rs45478197 | 1 | 154422733 | T | C | 0.10 | -2.03E-01 | 9.00E-03 | 70935 | 2.60E-113 | 0.08 | -1.52E-03 | 1.76E-03 | 326224 | 3.70E-01 | 508.75 |
| rs4553185 | 1 | 154410955 | T | C | 0.44 | 8.30E-01 | 3.90E-03 | 69970 | 1.00E-300 | 0.58 | -1.01E-03 | 9.71E-04 | 326224 | 3.40E-01 | 45281.66 |
| rs4845372 | 1 | 154415396 | A | C | 0.42 | 9.40E-01 | 3.60E-03 | 70935 | 1.00E-300 | 0.43 | 7.73E-04 | 9.70E-04 | 326224 | 3.90E-01 | 68164.51 |
| rs4845617 | 1 | 154377898 | A | G | 0.39 | 1.25E-01 | 5.70E-03 | 69966 | 1.71E-105 | 0.40 | 2.90E-04 | 9.84E-04 | 326224 | 8.00E-01 | 480.92 |
| rs4845618 | 1 | 154400015 | T | G | 0.44 | 8.09E-01 | 3.90E-03 | 70934 | 1.00E-300 | 0.57 | -9.68E-04 | 9.73E-04 | 326224 | 3.50E-01 | 42997.74 |
| rs4845637 | 1 | 154490178 | A | G | 0.53 | -5.36E-01 | 4.30E-03 | 70935 | 1.00E-300 | 0.36 | -1.36E-04 | 1.00E-03 | 326224 | 8.70E-01 | 15526.32 |
| rs57569414 | 1 | 154380419 | A | C | 0.12 | 4.79E-01 | 8.40E-03 | 69970 | 1.00E-300 | 0.12 | 2.42E-04 | 1.48E-03 | 326224 | 8.70E-01 | 3250.36 |
| rs59632925 | 1 | 154406540 | T | G | 0.56 | -8.16E-01 | 3.90E-03 | 70935 | 1.00E-300 | 0.41 | 7.21E-04 | 9.73E-04 | 326224 | 5.00E-01 | 43788.25 |
| rs6427631 | 1 | 154370020 | T | C | 0.68 | -1.84E-01 | 6.20E-03 | 70935 | 2.06E-193 | 0.71 | -1.01E-03 | 1.06E-03 | 326224 | 3.50E-01 | 878.84 |
| rs6657938 | 1 | 154336126 | A | G | 0.67 | -1.30E-01 | 6.10E-03 | 70935 | 4.00E-100 | 0.70 | -8.22E-04 | 1.04E-03 | 326224 | 4.50E-01 | 455.58 |
| rs6664039 | 1 | 154337238 | A | G | 0.79 | -2.58E-01 | 7.80E-03 | 70935 | 8.02E-240 | 0.85 | -6.74E-04 | 1.35E-03 | 326224 | 6.20E-01 | 1096.63 |
| rs6664608 | 1 | 154479670 | T | C | 0.46 | 5.36E-01 | 4.30E-03 | 70935 | 1.00E-300 | 0.64 | 1.89E-04 | 1.00E-03 | 326224 | 8.30E-01 | 15549.51 |
| rs66654715 | 1 | 154376820 | C | G | 0.03 | -5.54E-01 | 1.76E-02 | 68557 | 9.70E-218 | 0.03 | 1.80E-04 | 2.80E-03 | 326224 | 9.70E-01 | 991.53 |
| rs6667434 | 1 | 154409100 | A | G | 0.56 | -8.17E-01 | 3.90E-03 | 70935 | 1.00E-300 | 0.41 | 7.07E-04 | 9.74E-04 | 326224 | 5.10E-01 | 43863.40 |
| rs6674171 | 1 | 154491683 | A | G | 0.77 | 5.88E-01 | 5.40E-03 | 69970 | 1.00E-300 | 0.81 | -2.24E-03 | 1.21E-03 | 326224 | 6.90E-02 | 11836.63 |
| rs6675472 | 1 | 154445503 | T | C | 0.58 | -5.88E-01 | 5.30E-03 | 70935 | 1.00E-300 | 0.19 | 2.29E-03 | 1.22E-03 | 326224 | 6.40E-02 | 12287.51 |
| rs6683206 | 1 | 154418088 | T | C | 0.56 | -8.25E-01 | 3.90E-03 | 70935 | 1.00E-300 | 0.41 | 7.29E-04 | 9.75E-04 | 326224 | 5.00E-01 | 44759.37 |
| rs6684439 | 1 | 154395839 | T | C | 0.40 | 9.64E-01 | 3.80E-03 | 62756 | 1.00E-300 | 0.41 | 4.92E-04 | 9.90E-04 | 326224 | 5.70E-01 | 64355.68 |
| rs6686467 | 1 | 154329095 | A | G | 0.14 | 2.52E-01 | 7.80E-03 | 70935 | 5.53E-228 | 0.15 | 8.08E-04 | 1.35E-03 | 326224 | 5.50E-01 | 1041.30 |
| rs6686750 | 1 | 154419843 | A | G | 0.55 | -8.42E-01 | 3.90E-03 | 70935 | 1.00E-300 | 0.42 | 8.86E-04 | 9.70E-04 | 326224 | 4.00E-01 | 46633.85 |
| rs6687597 | 1 | 154434936 | A | G | 0.39 | 6.20E-01 | 5.00E-03 | 70935 | 1.00E-300 | 0.77 | -1.80E-04 | 1.15E-03 | 326224 | 8.90E-01 | 15351.21 |
| rs6694817 | 1 | 154401972 | T | C | 0.56 | -7.97E-01 | 4.00E-03 | 70933 | 1.00E-300 | 0.42 | 6.15E-04 | 9.74E-04 | 326224 | 5.70E-01 | 39740.42 |
| rs6700296 | 1 | 154473660 | T | C | 0.53 | -5.36E-01 | 4.30E-03 | 70935 | 1.00E-300 | 0.36 | -1.90E-04 | 1.00E-03 | 326224 | 8.30E-01 | 15537.91 |
| rs72633646 | 1 | 154334683 | A | G | 0.14 | 2.53E-01 | 7.80E-03 | 70935 | 1.75E-230 | 0.15 | 6.94E-04 | 1.35E-03 | 326224 | 6.10E-01 | 1053.75 |
| rs72633650 | 1 | 154360838 | T | C | 0.79 | -3.54E-01 | 8.00E-03 | 70934 | 1.00E-300 | 0.86 | -6.29E-04 | 1.39E-03 | 326224 | 6.40E-01 | 1955.85 |
| rs72698115 | 1 | 154379369 | A | C | 0.82 | 1.79E-01 | 8.80E-03 | 70934 | 1.14E-92 | 0.90 | 1.58E-03 | 1.59E-03 | 326224 | 3.00E-01 | 412.37 |
| rs72698169 | 1 | 154486799 | A | C | 0.78 | -7.11E-01 | 5.50E-03 | 70935 | 1.00E-300 | 0.84 | -7.67E-05 | 1.31E-03 | 326224 | 8.80E-01 | 16697.34 |
| rs73018293 | 1 | 154465577 | T | C | 0.20 | -5.86E-01 | 5.30E-03 | 70935 | 1.00E-300 | 0.19 | 2.20E-03 | 1.21E-03 | 326224 | 7.40E-02 | 12233.19 |
| rs73020232 | 1 | 154482669 | T | C | 0.20 | -5.86E-01 | 5.30E-03 | 70935 | 1.00E-300 | 0.19 | 2.18E-03 | 1.21E-03 | 326224 | 7.70E-02 | 12237.37 |
| rs73020246 | 1 | 154485640 | A | G | 0.77 | 5.87E-01 | 5.30E-03 | 70935 | 1.00E-300 | 0.81 | -2.19E-03 | 1.21E-03 | 326224 | 7.60E-02 | 12258.25 |
| rs7513603 | 1 | 154481158 | T | C | 0.46 | -2.13E-01 | 6.90E-03 | 70935 | 5.71E-206 | 0.16 | -2.85E-03 | 1.30E-03 | 326224 | 2.80E-02 | 951.14 |
| rs7519499 | 1 | 154487926 | A | G | 0.46 | 5.36E-01 | 4.30E-03 | 70935 | 1.00E-300 | 0.64 | 1.73E-04 | 1.00E-03 | 326224 | 8.40E-01 | 15555.31 |
| rs7521458 | 1 | 154407713 | T | C | 0.57 | -9.71E-01 | 3.50E-03 | 70935 | 1.00E-300 | 0.58 | -4.57E-04 | 9.73E-04 | 326224 | 5.80E-01 | 76966.61 |
| rs7525477 | 1 | 154394297 | A | G | 0.42 | -3.30E-01 | 5.80E-03 | 69969 | 1.00E-300 | 0.44 | -4.62E-04 | 1.01E-03 | 326224 | 6.90E-01 | 3245.07 |
| rs7526131 | 1 | 154425135 | A | G | 0.44 | 8.44E-01 | 3.90E-03 | 70935 | 1.00E-300 | 0.58 | -9.45E-04 | 9.70E-04 | 326224 | 3.70E-01 | 46811.21 |
| rs7537291 | 1 | 154433407 | A | G | 0.39 | 6.21E-01 | 5.00E-03 | 70935 | 1.00E-300 | 0.77 | -1.81E-04 | 1.15E-03 | 326224 | 8.90E-01 | 15410.74 |
| rs7549250 | 1 | 154404336 | T | C | 0.44 | 8.26E-01 | 3.90E-03 | 70933 | 1.00E-300 | 0.58 | -1.01E-03 | 9.69E-04 | 326224 | 3.40E-01 | 44878.79 |
| rs7553271 | 1 | 154373231 | T | C | 0.49 | 1.26E-01 | 5.70E-03 | 70934 | 1.13E-108 | 0.40 | 9.21E-05 | 9.82E-04 | 326224 | 9.70E-01 | 487.87 |
| rs79438587 | 1 | 154342517 | T | C | 0.16 | 3.48E-01 | 7.70E-03 | 69970 | 1.00E-300 | 0.17 | -1.20E-04 | 1.31E-03 | 326224 | 9.50E-01 | 2036.71 |
| rs79794939 | 1 | 154390932 | T | C | 0.07 | -3.75E-01 | 1.06E-02 | 70930 | 5.13E-272 | 0.08 | -2.00E-04 | 1.80E-03 | 326224 | 9.00E-01 | 1248.89 |
| rs9651053 | 1 | 154359411 | A | G | 0.11 | -1.66E-01 | 8.70E-03 | 70934 | 4.13E-82 | 0.10 | -1.39E-03 | 1.57E-03 | 326224 | 3.60E-01 | 365.82 |
| rs9803896 | 1 | 154347450 | A | G | 0.14 | 2.74E-01 | 7.70E-03 | 70935 | 7.89E-275 | 0.15 | 1.08E-03 | 1.34E-03 | 326224 | 4.20E-01 | 1269.95 |

Instrumental cis SNPs (50K) select for CRP to Sleep classical Mendelian randomization analysis. CHR: chromosome; POS: hg19 genomic position; A1: effective allele; A2: the other allele; A1_exp_frq: A1 frequency in the exposure dataset; Beta_exp, effect of A1 for the exposure; SE_exp; standard error for Beta_exp; N_exp: sample size for exposure; P_exp, association p value for exposure; A1_out_frq: A1 frequency in the outcome dataset; Beta_out: effect size of A1 on outcome; SE_out: standard error of Beta_out; N_out: sample size for outcome; P_out: association p value for outcome; F-stats: F-statistics.

# **Table S8. Harmonized instrumental cisSNPs (p<5x10^-8^) for sIL6R to Long Sleep MR.**

| SNP | CHR | POS | A1 | A2 | A1_exp_frq | Beta_exp | SE_exp | N_exp | P_exp | A1_out_frq | Beta_out | SE_out | N_out | P_out | Fstats |
| --- | --- | --- | --- | --- | --- | --- | --- | --- | --- | --- | --- | --- | --- | --- | --- |
| rs10047079 | 1 | 154468135 | T | C | 0.77 | 5.88E-01 | 5.40E-03 | 1.00E-300 | 69970 | 0.81 | 1.62E-04 | 9.21E-04 | 326224 | 8.40E-01 | 11836.63 |
| rs1073907 | 1 | 154332520 | T | C | 0.30 | 1.53E-01 | 8.10E-03 | 3.20E-79 | 35571 | 0.30 | -8.23E-04 | 7.91E-04 | 326224 | 3.10E-01 | 357.26 |
| rs10908839 | 1 | 154430798 | C | G | 0.58 | -6.11E-01 | 5.20E-03 | 1.00E-300 | 70935 | 0.78 | 7.16E-04 | 8.81E-04 | 326224 | 4.00E-01 | 13801.73 |
| rs10908840 | 1 | 154459477 | T | C | 0.44 | 1.21E-01 | 5.70E-03 | 2.23E-101 | 70930 | 0.48 | 1.02E-03 | 7.28E-04 | 326224 | 1.60E-01 | 449.89 |
| rs111885536 | 1 | 154461260 | A | G | 0.19 | -2.11E-01 | 7.00E-03 | 1.26E-202 | 70935 | 0.16 | -2.23E-03 | 9.87E-04 | 326224 | 2.50E-02 | 910.32 |
| rs11265606 | 1 | 154356459 | T | C | 0.28 | 1.80E-01 | 6.20E-03 | 4.84E-186 | 70935 | 0.29 | -1.19E-03 | 8.01E-04 | 326224 | 1.40E-01 | 844.75 |
| rs11265607 | 1 | 154357678 | A | G | 0.68 | -1.83E-01 | 6.20E-03 | 3.48E-191 | 70935 | 0.71 | 1.27E-03 | 8.01E-04 | 326224 | 1.20E-01 | 869.30 |
| rs11265621 | 1 | 154442960 | A | G | 0.46 | 5.41E-01 | 4.30E-03 | 1.00E-300 | 70935 | 0.65 | 1.53E-03 | 7.60E-04 | 326224 | 4.40E-02 | 15811.60 |
| rs11265622 | 1 | 154451420 | A | G | 0.53 | -5.37E-01 | 4.30E-03 | 1.00E-300 | 69970 | 0.36 | -1.43E-03 | 7.60E-04 | 326224 | 5.80E-02 | 15578.52 |
| rs113624284 | 1 | 154339299 | A | G | 0.79 | -2.60E-01 | 7.80E-03 | 1.49E-242 | 70935 | 0.85 | -1.28E-03 | 1.02E-03 | 326224 | 2.10E-01 | 1108.55 |
| rs11576181 | 1 | 154330659 | T | G | 0.67 | -1.23E-01 | 6.10E-03 | 1.70E-89 | 70935 | 0.70 | 8.29E-04 | 7.91E-04 | 326224 | 3.10E-01 | 405.26 |
| rs11580535 | 1 | 154364317 | T | G | 0.13 | 3.55E-01 | 8.00E-03 | 1.00E-300 | 70935 | 0.14 | 1.68E-04 | 1.06E-03 | 326224 | 8.70E-01 | 1965.81 |
| rs11582433 | 1 | 154349605 | T | C | 0.14 | 2.70E-01 | 7.70E-03 | 4.44E-269 | 70935 | 0.15 | 9.98E-04 | 1.01E-03 | 326224 | 3.20E-01 | 1233.20 |
| rs11590203 | 1 | 154333569 | T | G | 0.14 | 2.50E-01 | 7.90E-03 | 2.44E-222 | 69970 | 0.15 | 1.08E-03 | 1.02E-03 | 326224 | 2.90E-01 | 1002.24 |
| rs116088025 | 1 | 154354350 | T | C | 0.14 | 2.73E-01 | 7.70E-03 | 3.37E-273 | 70935 | 0.15 | 9.84E-04 | 1.01E-03 | 326224 | 3.30E-01 | 1259.79 |
| rs12023772 | 1 | 154483868 | A | G | 0.17 | 7.12E-01 | 5.50E-03 | 1.00E-300 | 70935 | 0.16 | 5.83E-04 | 9.90E-04 | 326224 | 5.60E-01 | 16749.07 |
| rs12025518 | 1 | 154340789 | A | C | 0.69 | 1.92E-01 | 6.30E-03 | 1.00E-200 | 69969 | 0.74 | 8.49E-05 | 8.27E-04 | 326224 | 9.00E-01 | 926.86 |
| rs12033701 | 1 | 154365886 | T | C | 0.11 | -1.66E-01 | 8.70E-03 | 1.23E-81 | 70934 | 0.10 | -1.47E-03 | 1.19E-03 | 326224 | 2.10E-01 | 364.06 |
| rs12044132 | 1 | 154462360 | T | C | 0.15 | 6.85E-01 | 9.60E-03 | 1.00E-300 | 34606 | 0.16 | 5.85E-04 | 9.90E-04 | 326224 | 5.60E-01 | 5086.96 |
| rs12061599 | 1 | 154344135 | T | G | 0.28 | 1.29E-01 | 6.20E-03 | 5.38E-98 | 70935 | 0.30 | -6.75E-04 | 7.92E-04 | 326224 | 4.10E-01 | 434.92 |
| rs12075836 | 1 | 154371487 | T | C | 0.14 | -2.62E-01 | 7.70E-03 | 8.28E-252 | 70935 | 0.15 | 1.51E-03 | 1.03E-03 | 326224 | 1.50E-01 | 1158.65 |
| rs12118018 | 1 | 154477440 | A | G | 0.46 | 5.36E-01 | 4.30E-03 | 1.00E-300 | 70935 | 0.64 | 1.44E-03 | 7.59E-04 | 326224 | 5.80E-02 | 15555.31 |
| rs12118721 | 1 | 154397416 | T | C | 0.55 | -8.09E-01 | 3.90E-03 | 1.00E-300 | 70934 | 0.43 | 1.08E-03 | 7.39E-04 | 326224 | 1.50E-01 | 42976.48 |
| rs12119111 | 1 | 154478600 | A | G | 0.46 | 5.37E-01 | 4.30E-03 | 1.00E-300 | 70935 | 0.64 | 1.45E-03 | 7.59E-04 | 326224 | 5.60E-02 | 15578.52 |
| rs12129500 | 1 | 154423764 | T | C | 0.56 | -8.43E-01 | 3.90E-03 | 1.00E-300 | 70935 | 0.42 | 8.34E-04 | 7.34E-04 | 326224 | 2.70E-01 | 46711.40 |
| rs12133641 | 1 | 154428283 | A | G | 0.57 | -1.01E+00 | 3.40E-03 | 1.00E-300 | 70935 | 0.59 | -3.38E-04 | 7.37E-04 | 326224 | 6.40E-01 | 88401.28 |
| rs12563459 | 1 | 154362686 | A | G | 0.15 | -1.85E-01 | 7.60E-03 | 1.95E-130 | 70935 | 0.15 | 1.15E-03 | 1.01E-03 | 326224 | 2.70E-01 | 594.46 |
| rs12568083 | 1 | 154455949 | T | C | 0.39 | -5.02E-01 | 7.20E-03 | 1.00E-300 | 34606 | 0.36 | -1.43E-03 | 7.60E-04 | 326224 | 5.90E-02 | 4853.44 |
| rs12739228 | 1 | 154426190 | A | G | 0.04 | -6.32E-01 | 1.59E-02 | 1.00E-300 | 68557 | 0.04 | 5.13E-04 | 1.92E-03 | 326224 | 7.70E-01 | 1578.44 |
| rs12753254 | 1 | 154416935 | A | G | 0.41 | 9.76E-01 | 3.50E-03 | 1.00E-300 | 70935 | 0.42 | 1.97E-04 | 7.36E-04 | 326224 | 7.80E-01 | 77745.37 |
| rs12753666 | 1 | 154474875 | A | G | 0.46 | 5.36E-01 | 4.30E-03 | 1.00E-300 | 70935 | 0.64 | 1.45E-03 | 7.59E-04 | 326224 | 5.50E-02 | 15537.91 |
| rs138765671 | 1 | 154345962 | A | C | 0.14 | 2.61E-01 | 7.80E-03 | 2.75E-243 | 70935 | 0.15 | 1.28E-03 | 1.02E-03 | 326224 | 2.10E-01 | 1123.11 |
| rs147763778 | 1 | 154368223 | A | G | 0.04 | 3.99E-01 | 1.60E-02 | 3.27E-138 | 68557 | 0.04 | -1.82E-03 | 1.87E-03 | 326224 | 3.30E-01 | 623.13 |
| rs1889313 | 1 | 154351717 | A | C | 0.14 | 2.70E-01 | 7.70E-03 | 6.23E-269 | 70935 | 0.15 | 9.80E-04 | 1.01E-03 | 326224 | 3.30E-01 | 1233.20 |
| rs2229238 | 1 | 154437896 | T | C | 0.58 | -5.86E-01 | 5.50E-03 | 1.00E-300 | 69970 | 0.18 | -6.39E-04 | 9.36E-04 | 326224 | 4.80E-01 | 11336.44 |
| rs34094138 | 1 | 154366911 | T | G | 0.78 | 1.86E-01 | 7.60E-03 | 1.20E-131 | 70935 | 0.85 | -1.18E-03 | 1.01E-03 | 326224 | 2.50E-01 | 597.03 |
| rs35717427 | 1 | 154391882 | A | G | 0.12 | 4.99E-01 | 8.30E-03 | 1.00E-300 | 69969 | 0.12 | 2.36E-04 | 1.11E-03 | 326224 | 8.30E-01 | 3607.23 |
| rs4240872 | 1 | 154436195 | T | C | 0.39 | 6.19E-01 | 5.00E-03 | 1.00E-300 | 70935 | 0.77 | 4.97E-04 | 8.68E-04 | 326224 | 5.40E-01 | 15331.39 |
| rs4393147 | 1 | 154414037 | T | C | 0.41 | 9.75E-01 | 3.50E-03 | 1.00E-300 | 70935 | 0.42 | 1.72E-04 | 7.37E-04 | 326224 | 8.00E-01 | 77554.29 |
| rs4474240 | 1 | 154457855 | A | C | 0.47 | -2.14E-01 | 7.00E-03 | 5.07E-206 | 69970 | 0.16 | -2.25E-03 | 9.87E-04 | 326224 | 2.30E-02 | 933.74 |
| rs4478801 | 1 | 154464572 | A | G | 0.58 | 5.01E-01 | 7.20E-03 | 1.00E-300 | 34598 | 0.64 | 1.43E-03 | 7.59E-04 | 326224 | 5.80E-02 | 4843.77 |
| rs4521987 | 1 | 154388668 | T | C | 0.45 | -2.73E-01 | 7.90E-03 | 8.69E-265 | 69969 | 0.15 | 1.81E-03 | 1.04E-03 | 326224 | 8.50E-02 | 1196.81 |
| rs45478197 | 1 | 154422733 | T | C | 0.10 | -2.03E-01 | 9.00E-03 | 2.60E-113 | 70935 | 0.08 | -1.19E-03 | 1.33E-03 | 326224 | 3.80E-01 | 508.75 |
| rs4553185 | 1 | 154410955 | T | C | 0.44 | 8.30E-01 | 3.90E-03 | 1.00E-300 | 69970 | 0.58 | -1.10E-03 | 7.35E-04 | 326224 | 1.40E-01 | 45281.66 |
| rs4845372 | 1 | 154415396 | A | C | 0.42 | 9.40E-01 | 3.60E-03 | 1.00E-300 | 70935 | 0.43 | 3.57E-04 | 7.34E-04 | 326224 | 6.20E-01 | 68164.51 |
| rs4845617 | 1 | 154377898 | A | G | 0.39 | 1.25E-01 | 5.70E-03 | 1.71E-105 | 69966 | 0.40 | -1.68E-03 | 7.45E-04 | 326224 | 2.50E-02 | 480.92 |
| rs4845618 | 1 | 154400015 | T | G | 0.44 | 8.09E-01 | 3.90E-03 | 1.00E-300 | 70934 | 0.57 | -1.09E-03 | 7.37E-04 | 326224 | 1.50E-01 | 42997.74 |
| rs4845637 | 1 | 154490178 | A | G | 0.53 | -5.36E-01 | 4.30E-03 | 1.00E-300 | 70935 | 0.36 | -1.46E-03 | 7.59E-04 | 326224 | 5.30E-02 | 15526.32 |
| rs57569414 | 1 | 154380419 | A | C | 0.12 | 4.79E-01 | 8.40E-03 | 1.00E-300 | 69970 | 0.12 | 1.49E-05 | 1.12E-03 | 326224 | 9.80E-01 | 3250.36 |
| rs59632925 | 1 | 154406540 | T | G | 0.56 | -8.16E-01 | 3.90E-03 | 1.00E-300 | 70935 | 0.41 | 9.28E-04 | 7.37E-04 | 326224 | 2.20E-01 | 43788.25 |
| rs6427631 | 1 | 154370020 | T | C | 0.68 | -1.84E-01 | 6.20E-03 | 2.06E-193 | 70935 | 0.71 | 1.23E-03 | 8.02E-04 | 326224 | 1.30E-01 | 878.84 |
| rs6657938 | 1 | 154336126 | A | G | 0.67 | -1.30E-01 | 6.10E-03 | 4.00E-100 | 70935 | 0.70 | 7.35E-04 | 7.91E-04 | 326224 | 3.70E-01 | 455.58 |
| rs6664039 | 1 | 154337238 | A | G | 0.79 | -2.58E-01 | 7.80E-03 | 8.02E-240 | 70935 | 0.85 | -1.23E-03 | 1.02E-03 | 326224 | 2.30E-01 | 1096.63 |
| rs6664608 | 1 | 154479670 | T | C | 0.46 | 5.36E-01 | 4.30E-03 | 1.00E-300 | 70935 | 0.64 | 1.44E-03 | 7.59E-04 | 326224 | 5.80E-02 | 15549.51 |
| rs66654715 | 1 | 154376820 | C | G | 0.03 | -5.54E-01 | 1.76E-02 | 9.70E-218 | 68557 | 0.03 | 1.55E-04 | 2.12E-03 | 326224 | 9.30E-01 | 991.53 |
| rs6667434 | 1 | 154409100 | A | G | 0.56 | -8.17E-01 | 3.90E-03 | 1.00E-300 | 70935 | 0.41 | 9.50E-04 | 7.37E-04 | 326224 | 2.10E-01 | 43863.40 |
| rs6674171 | 1 | 154491683 | A | G | 0.77 | 5.88E-01 | 5.40E-03 | 1.00E-300 | 69970 | 0.81 | 1.65E-04 | 9.21E-04 | 326224 | 8.40E-01 | 11836.63 |
| rs6675472 | 1 | 154445503 | T | C | 0.58 | -5.88E-01 | 5.30E-03 | 1.00E-300 | 70935 | 0.19 | -2.90E-04 | 9.23E-04 | 326224 | 7.30E-01 | 12287.51 |
| rs6683206 | 1 | 154418088 | T | C | 0.56 | -8.25E-01 | 3.90E-03 | 1.00E-300 | 70935 | 0.41 | 9.10E-04 | 7.38E-04 | 326224 | 2.30E-01 | 44759.37 |
| rs6684439 | 1 | 154395839 | T | C | 0.40 | 9.64E-01 | 3.80E-03 | 1.00E-300 | 62756 | 0.41 | 1.48E-04 | 7.50E-04 | 326224 | 8.30E-01 | 64355.68 |
| rs6686467 | 1 | 154329095 | A | G | 0.14 | 2.52E-01 | 7.80E-03 | 5.53E-228 | 70935 | 0.15 | 1.06E-03 | 1.02E-03 | 326224 | 3.00E-01 | 1041.30 |
| rs6686750 | 1 | 154419843 | A | G | 0.55 | -8.42E-01 | 3.90E-03 | 1.00E-300 | 70935 | 0.42 | 8.33E-04 | 7.35E-04 | 326224 | 2.70E-01 | 46633.85 |
| rs6687597 | 1 | 154434936 | A | G | 0.39 | 6.20E-01 | 5.00E-03 | 1.00E-300 | 70935 | 0.77 | 5.15E-04 | 8.68E-04 | 326224 | 5.30E-01 | 15351.21 |
| rs6694817 | 1 | 154401972 | T | C | 0.56 | -7.97E-01 | 4.00E-03 | 1.00E-300 | 70933 | 0.42 | 9.24E-04 | 7.37E-04 | 326224 | 2.20E-01 | 39740.42 |
| rs6700296 | 1 | 154473660 | T | C | 0.53 | -5.36E-01 | 4.30E-03 | 1.00E-300 | 70935 | 0.36 | -1.44E-03 | 7.59E-04 | 326224 | 5.70E-02 | 15537.91 |
| rs72633646 | 1 | 154334683 | A | G | 0.14 | 2.53E-01 | 7.80E-03 | 1.75E-230 | 70935 | 0.15 | 1.10E-03 | 1.02E-03 | 326224 | 2.80E-01 | 1053.75 |
| rs72633650 | 1 | 154360838 | T | C | 0.79 | -3.54E-01 | 8.00E-03 | 1.00E-300 | 70934 | 0.86 | -1.30E-04 | 1.05E-03 | 326224 | 9.00E-01 | 1955.85 |
| rs72698115 | 1 | 154379369 | A | C | 0.82 | 1.79E-01 | 8.80E-03 | 1.14E-92 | 70934 | 0.90 | 1.44E-03 | 1.20E-03 | 326224 | 2.30E-01 | 412.37 |
| rs72698169 | 1 | 154486799 | A | C | 0.78 | -7.11E-01 | 5.50E-03 | 1.00E-300 | 70935 | 0.84 | -6.47E-04 | 9.90E-04 | 326224 | 5.20E-01 | 16697.34 |
| rs73018293 | 1 | 154465577 | T | C | 0.20 | -5.86E-01 | 5.30E-03 | 1.00E-300 | 70935 | 0.19 | -1.79E-04 | 9.21E-04 | 326224 | 8.30E-01 | 12233.19 |
| rs73020232 | 1 | 154482669 | T | C | 0.20 | -5.86E-01 | 5.30E-03 | 1.00E-300 | 70935 | 0.19 | -1.43E-04 | 9.21E-04 | 326224 | 8.60E-01 | 12237.37 |
| rs73020246 | 1 | 154485640 | A | G | 0.77 | 5.87E-01 | 5.30E-03 | 1.00E-300 | 70935 | 0.81 | 1.32E-04 | 9.21E-04 | 326224 | 8.70E-01 | 12258.25 |
| rs7513603 | 1 | 154481158 | T | C | 0.46 | -2.13E-01 | 6.90E-03 | 5.71E-206 | 70935 | 0.16 | -2.26E-03 | 9.86E-04 | 326224 | 2.30E-02 | 951.14 |
| rs7519499 | 1 | 154487926 | A | G | 0.46 | 5.36E-01 | 4.30E-03 | 1.00E-300 | 70935 | 0.64 | 1.47E-03 | 7.59E-04 | 326224 | 5.20E-02 | 15555.31 |
| rs7521458 | 1 | 154407713 | T | C | 0.57 | -9.71E-01 | 3.50E-03 | 1.00E-300 | 70935 | 0.58 | -1.76E-04 | 7.37E-04 | 326224 | 8.00E-01 | 76966.61 |
| rs7525477 | 1 | 154394297 | A | G | 0.42 | -3.30E-01 | 5.80E-03 | 1.00E-300 | 69969 | 0.44 | 3.56E-04 | 7.66E-04 | 326224 | 6.40E-01 | 3245.07 |
| rs7526131 | 1 | 154425135 | A | G | 0.44 | 8.44E-01 | 3.90E-03 | 1.00E-300 | 70935 | 0.58 | -8.46E-04 | 7.34E-04 | 326224 | 2.60E-01 | 46811.21 |
| rs7537291 | 1 | 154433407 | A | G | 0.39 | 6.21E-01 | 5.00E-03 | 1.00E-300 | 70935 | 0.77 | 5.37E-04 | 8.68E-04 | 326224 | 5.10E-01 | 15410.74 |
| rs7549250 | 1 | 154404336 | T | C | 0.44 | 8.26E-01 | 3.90E-03 | 1.00E-300 | 70933 | 0.58 | -1.09E-03 | 7.34E-04 | 326224 | 1.40E-01 | 44878.79 |
| rs7553271 | 1 | 154373231 | T | C | 0.49 | 1.26E-01 | 5.70E-03 | 1.13E-108 | 70934 | 0.40 | -1.72E-03 | 7.44E-04 | 326224 | 2.20E-02 | 487.87 |
| rs79438587 | 1 | 154342517 | T | C | 0.16 | 3.48E-01 | 7.70E-03 | 1.00E-300 | 69970 | 0.17 | 8.21E-04 | 9.92E-04 | 326224 | 4.00E-01 | 2036.71 |
| rs79794939 | 1 | 154390932 | T | C | 0.07 | -3.75E-01 | 1.06E-02 | 5.13E-272 | 70930 | 0.08 | 3.51E-03 | 1.36E-03 | 326224 | 1.00E-02 | 1248.89 |
| rs9651053 | 1 | 154359411 | A | G | 0.11 | -1.66E-01 | 8.70E-03 | 4.13E-82 | 70934 | 0.10 | -1.45E-03 | 1.19E-03 | 326224 | 2.20E-01 | 365.82 |
| rs9803896 | 1 | 154347450 | A | G | 0.14 | 2.74E-01 | 7.70E-03 | 7.89E-275 | 70935 | 0.15 | 1.14E-03 | 1.01E-03 | 326224 | 2.60E-01 | 1269.95 |

Instrumental cis SNPs (50K) select for CRP to Sleep classical Mendelian randomization analysis. CHR: chromosome; POS: hg19 genomic position; A1: effective allele; A2: the other allele; A1_exp_frq: A1 frequency in the exposure dataset; Beta_exp, effect of A1 for the exposure; SE_exp; standard error for Beta_exp; N_exp: sample size for exposure; P_exp, association p value for exposure; A1_out_frq: A1 frequency in the outcome dataset; Beta_out: effect size of A1 on outcome; SE_out: standard error of Beta_out; N_out: sample size for outcome; P_out: association p value for outcome; F-stats: F-statistics.

# **Table S9. Harmonized instrumental cisSNPs (p<5x10^-8^) for sIL6R to Sleep Duration MR.**

| SNP | CHR | POS | A1 | A2 | A1_exp_frq | Beta_exp | SE_exp | N_exp | P_exp | A1_out_frq | Beta_out | SE_out | N_out | P_out | Fstats |
| --- | --- | --- | --- | --- | --- | --- | --- | --- | --- | --- | --- | --- | --- | --- | --- |
| rs10047079 | 1 | 154468135 | T | C | 0.77 | 5.88E-01 | 5.40E-03 | 69970 | 1.00E-300 | 0.81 | 7.32E-03 | 2.87E-03 | 326224 | 1.20E-02 | 11836.63 |
| rs1073907 | 1 | 154332520 | T | C | 0.30 | 1.53E-01 | 8.10E-03 | 35570.9 | 3.20E-79 | 0.30 | -2.39E-03 | 2.46E-03 | 326224 | 3.50E-01 | 357.26 |
| rs10908839 | 1 | 154430798 | C | G | 0.58 | -6.11E-01 | 5.20E-03 | 70935 | 1.00E-300 | 0.78 | 2.70E-03 | 2.74E-03 | 326224 | 3.50E-01 | 13801.73 |
| rs10908840 | 1 | 154459477 | T | C | 0.44 | 1.21E-01 | 5.70E-03 | 70929.9 | 2.23E-101 | 0.48 | 1.90E-03 | 2.27E-03 | 326224 | 4.10E-01 | 449.89 |
| rs111885536 | 1 | 154461260 | A | G | 0.19 | -2.11E-01 | 7.00E-03 | 70935 | 1.26E-202 | 0.16 | 2.10E-03 | 3.08E-03 | 326224 | 4.80E-01 | 910.32 |
| rs11265606 | 1 | 154356459 | T | C | 0.28 | 1.80E-01 | 6.20E-03 | 70934.9 | 4.84E-186 | 0.29 | -3.25E-03 | 2.49E-03 | 326224 | 2.00E-01 | 844.75 |
| rs11265607 | 1 | 154357678 | A | G | 0.68 | -1.83E-01 | 6.20E-03 | 70934.9 | 3.48E-191 | 0.71 | 3.35E-03 | 2.49E-03 | 326224 | 1.80E-01 | 869.30 |
| rs11265621 | 1 | 154442960 | A | G | 0.46 | 5.41E-01 | 4.30E-03 | 70934.9 | 1.00E-300 | 0.65 | 4.27E-03 | 2.37E-03 | 326224 | 8.10E-02 | 15811.60 |
| rs11265622 | 1 | 154451420 | A | G | 0.53 | -5.37E-01 | 4.30E-03 | 69970 | 1.00E-300 | 0.36 | -3.98E-03 | 2.37E-03 | 326224 | 1.00E-01 | 15578.52 |
| rs113624284 | 1 | 154339299 | A | G | 0.79 | -2.60E-01 | 7.80E-03 | 70935 | 1.49E-242 | 0.85 | -1.46E-03 | 3.18E-03 | 326224 | 6.90E-01 | 1108.55 |
| rs11576181 | 1 | 154330659 | T | G | 0.67 | -1.23E-01 | 6.10E-03 | 70934.9 | 1.70E-89 | 0.70 | 2.54E-03 | 2.46E-03 | 326224 | 3.20E-01 | 405.26 |
| rs11580535 | 1 | 154364317 | T | G | 0.13 | 3.55E-01 | 8.00E-03 | 70935 | 1.00E-300 | 0.14 | -1.83E-04 | 3.29E-03 | 326224 | 8.90E-01 | 1965.81 |
| rs11582433 | 1 | 154349605 | T | C | 0.14 | 2.70E-01 | 7.70E-03 | 70935 | 4.44E-269 | 0.15 | 8.80E-04 | 3.15E-03 | 326224 | 8.40E-01 | 1233.20 |
| rs11590203 | 1 | 154333569 | T | G | 0.14 | 2.50E-01 | 7.90E-03 | 69970 | 2.44E-222 | 0.15 | 1.41E-03 | 3.19E-03 | 326224 | 7.10E-01 | 1002.24 |
| rs116088025 | 1 | 154354350 | T | C | 0.14 | 2.73E-01 | 7.70E-03 | 70935 | 3.37E-273 | 0.15 | 7.19E-04 | 3.15E-03 | 326224 | 8.90E-01 | 1259.79 |
| rs12023772 | 1 | 154483868 | A | G | 0.17 | 7.12E-01 | 5.50E-03 | 70934.9 | 1.00E-300 | 0.16 | 3.00E-03 | 3.09E-03 | 326224 | 3.50E-01 | 16749.07 |
| rs12025518 | 1 | 154340789 | A | C | 0.69 | 1.92E-01 | 6.30E-03 | 69969 | 1.00E-200 | 0.74 | 1.62E-05 | 2.58E-03 | 326224 | 9.70E-01 | 926.86 |
| rs12033701 | 1 | 154365886 | T | C | 0.11 | -1.66E-01 | 8.70E-03 | 70934 | 1.23E-81 | 0.10 | 9.26E-05 | 3.72E-03 | 326224 | 9.30E-01 | 364.06 |
| rs12044132 | 1 | 154462360 | T | C | 0.15 | 6.85E-01 | 9.60E-03 | 34606 | 1.00E-300 | 0.16 | 3.27E-03 | 3.09E-03 | 326224 | 3.10E-01 | 5086.96 |
| rs12061599 | 1 | 154344135 | T | G | 0.28 | 1.29E-01 | 6.20E-03 | 70935 | 5.38E-98 | 0.30 | -2.37E-03 | 2.46E-03 | 326224 | 3.50E-01 | 434.92 |
| rs12075836 | 1 | 154371487 | T | C | 0.14 | -2.62E-01 | 7.70E-03 | 70934.9 | 8.28E-252 | 0.15 | 1.21E-03 | 3.20E-03 | 326224 | 7.10E-01 | 1158.65 |
| rs12118018 | 1 | 154477440 | A | G | 0.46 | 5.36E-01 | 4.30E-03 | 70934.9 | 1.00E-300 | 0.64 | 3.95E-03 | 2.37E-03 | 326224 | 1.00E-01 | 15555.31 |
| rs12118721 | 1 | 154397416 | T | C | 0.55 | -8.09E-01 | 3.90E-03 | 70933.9 | 1.00E-300 | 0.43 | -1.23E-03 | 2.30E-03 | 326224 | 6.10E-01 | 42976.48 |
| rs12119111 | 1 | 154478600 | A | G | 0.46 | 5.37E-01 | 4.30E-03 | 70934.9 | 1.00E-300 | 0.64 | 3.86E-03 | 2.37E-03 | 326224 | 1.10E-01 | 15578.52 |
| rs12129500 | 1 | 154423764 | T | C | 0.56 | -8.43E-01 | 3.90E-03 | 70934.9 | 1.00E-300 | 0.42 | -1.31E-03 | 2.29E-03 | 326224 | 5.90E-01 | 46711.40 |
| rs12133641 | 1 | 154428283 | A | G | 0.57 | -1.01E+00 | 3.40E-03 | 70935 | 1.00E-300 | 0.59 | -1.41E-03 | 2.30E-03 | 326224 | 5.80E-01 | 88401.28 |
| rs12563459 | 1 | 154362686 | A | G | 0.15 | -1.85E-01 | 7.60E-03 | 70934.9 | 1.95E-130 | 0.15 | 2.81E-04 | 3.14E-03 | 326224 | 9.20E-01 | 594.46 |
| rs12568083 | 1 | 154455949 | T | C | 0.39 | -5.02E-01 | 7.20E-03 | 34606 | 1.00E-300 | 0.36 | -3.97E-03 | 2.37E-03 | 326224 | 1.00E-01 | 4853.44 |
| rs12739228 | 1 | 154426190 | A | G | 0.04 | -6.32E-01 | 1.59E-02 | 68557 | 1.00E-300 | 0.04 | -2.61E-03 | 5.98E-03 | 326224 | 7.00E-01 | 1578.44 |
| rs12753254 | 1 | 154416935 | A | G | 0.41 | 9.76E-01 | 3.50E-03 | 70934.9 | 1.00E-300 | 0.42 | 7.58E-04 | 2.29E-03 | 326224 | 7.80E-01 | 77745.37 |
| rs12753666 | 1 | 154474875 | A | G | 0.46 | 5.36E-01 | 4.30E-03 | 70934.9 | 1.00E-300 | 0.64 | 3.98E-03 | 2.37E-03 | 326224 | 1.00E-01 | 15537.91 |
| rs138765671 | 1 | 154345962 | A | C | 0.14 | 2.61E-01 | 7.80E-03 | 70935 | 2.75E-243 | 0.15 | 1.47E-03 | 3.19E-03 | 326224 | 7.00E-01 | 1123.11 |
| rs147763778 | 1 | 154368223 | A | G | 0.04 | 3.99E-01 | 1.60E-02 | 68557 | 3.27E-138 | 0.04 | -2.24E-03 | 5.82E-03 | 326224 | 6.80E-01 | 623.13 |
| rs1889313 | 1 | 154351717 | A | C | 0.14 | 2.70E-01 | 7.70E-03 | 70935 | 6.23E-269 | 0.15 | 8.47E-04 | 3.15E-03 | 326224 | 8.50E-01 | 1233.20 |
| rs2229238 | 1 | 154437896 | T | C | 0.58 | -5.86E-01 | 5.50E-03 | 69970 | 1.00E-300 | 0.18 | -7.74E-03 | 2.91E-03 | 326224 | 8.70E-03 | 11336.44 |
| rs34094138 | 1 | 154366911 | T | G | 0.78 | 1.86E-01 | 7.60E-03 | 70935 | 1.20E-131 | 0.85 | -2.46E-04 | 3.14E-03 | 326224 | 9.30E-01 | 597.03 |
| rs35717427 | 1 | 154391882 | A | G | 0.12 | 4.99E-01 | 8.30E-03 | 69968.9 | 1.00E-300 | 0.12 | 1.87E-03 | 3.45E-03 | 326224 | 6.20E-01 | 3607.23 |
| rs4240872 | 1 | 154436195 | T | C | 0.39 | 6.19E-01 | 5.00E-03 | 70935 | 1.00E-300 | 0.77 | 3.45E-03 | 2.70E-03 | 326224 | 2.20E-01 | 15331.39 |
| rs4393147 | 1 | 154414037 | T | C | 0.41 | 9.75E-01 | 3.50E-03 | 70934.9 | 1.00E-300 | 0.42 | 6.28E-04 | 2.30E-03 | 326224 | 8.20E-01 | 77554.29 |
| rs4474240 | 1 | 154457855 | A | C | 0.47 | -2.14E-01 | 7.00E-03 | 69970 | 5.07E-206 | 0.16 | 1.87E-03 | 3.08E-03 | 326224 | 5.30E-01 | 933.74 |
| rs4478801 | 1 | 154464572 | A | G | 0.58 | 5.01E-01 | 7.20E-03 | 34597.9 | 1.00E-300 | 0.64 | 3.97E-03 | 2.37E-03 | 326224 | 1.00E-01 | 4843.77 |
| rs4521987 | 1 | 154388668 | T | C | 0.45 | -2.73E-01 | 7.90E-03 | 69968.9 | 8.69E-265 | 0.15 | 1.64E-03 | 3.23E-03 | 326224 | 6.10E-01 | 1196.81 |
| rs45478197 | 1 | 154422733 | T | C | 0.10 | -2.03E-01 | 9.00E-03 | 70935 | 2.60E-113 | 0.08 | 1.47E-03 | 4.16E-03 | 326224 | 6.70E-01 | 508.75 |
| rs4553185 | 1 | 154410955 | T | C | 0.44 | 8.30E-01 | 3.90E-03 | 69969.9 | 1.00E-300 | 0.58 | 1.08E-03 | 2.29E-03 | 326224 | 6.60E-01 | 45281.66 |
| rs4845372 | 1 | 154415396 | A | C | 0.42 | 9.40E-01 | 3.60E-03 | 70934.9 | 1.00E-300 | 0.43 | -1.28E-04 | 2.29E-03 | 326224 | 9.20E-01 | 68164.51 |
| rs4845617 | 1 | 154377898 | A | G | 0.39 | 1.25E-01 | 5.70E-03 | 69965.8 | 1.71E-105 | 0.40 | -3.33E-03 | 2.32E-03 | 326224 | 1.70E-01 | 480.92 |
| rs4845618 | 1 | 154400015 | T | G | 0.44 | 8.09E-01 | 3.90E-03 | 70934 | 1.00E-300 | 0.57 | 1.12E-03 | 2.30E-03 | 326224 | 6.40E-01 | 42997.74 |
| rs4845637 | 1 | 154490178 | A | G | 0.53 | -5.36E-01 | 4.30E-03 | 70934.9 | 1.00E-300 | 0.36 | -4.15E-03 | 2.37E-03 | 326224 | 8.70E-02 | 15526.32 |
| rs57569414 | 1 | 154380419 | A | C | 0.12 | 4.79E-01 | 8.40E-03 | 69969.9 | 1.00E-300 | 0.12 | 6.57E-04 | 3.50E-03 | 326224 | 8.90E-01 | 3250.36 |
| rs59632925 | 1 | 154406540 | T | G | 0.56 | -8.16E-01 | 3.90E-03 | 70934.9 | 1.00E-300 | 0.41 | -3.86E-04 | 2.30E-03 | 326224 | 8.80E-01 | 43788.25 |
| rs6427631 | 1 | 154370020 | T | C | 0.68 | -1.84E-01 | 6.20E-03 | 70934.9 | 2.06E-193 | 0.71 | 3.43E-03 | 2.50E-03 | 326224 | 1.70E-01 | 878.84 |
| rs6657938 | 1 | 154336126 | A | G | 0.67 | -1.30E-01 | 6.10E-03 | 70935 | 4.00E-100 | 0.70 | 2.20E-03 | 2.46E-03 | 326224 | 3.90E-01 | 455.58 |
| rs6664039 | 1 | 154337238 | A | G | 0.79 | -2.58E-01 | 7.80E-03 | 70935 | 8.02E-240 | 0.85 | -1.50E-03 | 3.18E-03 | 326224 | 6.80E-01 | 1096.63 |
| rs6664608 | 1 | 154479670 | T | C | 0.46 | 5.36E-01 | 4.30E-03 | 70934.9 | 1.00E-300 | 0.64 | 3.94E-03 | 2.36E-03 | 326224 | 1.00E-01 | 15549.51 |
| rs66654715 | 1 | 154376820 | C | G | 0.03 | -5.54E-01 | 1.76E-02 | 68557 | 9.70E-218 | 0.03 | -1.99E-04 | 6.60E-03 | 326224 | 9.80E-01 | 991.53 |
| rs6667434 | 1 | 154409100 | A | G | 0.56 | -8.17E-01 | 3.90E-03 | 70934.9 | 1.00E-300 | 0.41 | -3.65E-04 | 2.30E-03 | 326224 | 8.90E-01 | 43863.40 |
| rs6674171 | 1 | 154491683 | A | G | 0.77 | 5.88E-01 | 5.40E-03 | 69970 | 1.00E-300 | 0.81 | 7.48E-03 | 2.87E-03 | 326224 | 1.00E-02 | 11836.63 |
| rs6675472 | 1 | 154445503 | T | C | 0.58 | -5.88E-01 | 5.30E-03 | 70935 | 1.00E-300 | 0.19 | -7.68E-03 | 2.87E-03 | 326224 | 8.40E-03 | 12287.51 |
| rs6683206 | 1 | 154418088 | T | C | 0.56 | -8.25E-01 | 3.90E-03 | 70935 | 1.00E-300 | 0.41 | -6.10E-04 | 2.30E-03 | 326224 | 8.10E-01 | 44759.37 |
| rs6684439 | 1 | 154395839 | T | C | 0.40 | 9.64E-01 | 3.80E-03 | 62755.9 | 1.00E-300 | 0.41 | 5.59E-04 | 2.34E-03 | 326224 | 8.50E-01 | 64355.68 |
| rs6686467 | 1 | 154329095 | A | G | 0.14 | 2.52E-01 | 7.80E-03 | 70935 | 5.53E-228 | 0.15 | 1.19E-03 | 3.19E-03 | 326224 | 7.60E-01 | 1041.30 |
| rs6686750 | 1 | 154419843 | A | G | 0.55 | -8.42E-01 | 3.90E-03 | 70934.9 | 1.00E-300 | 0.42 | -1.25E-03 | 2.29E-03 | 326224 | 6.00E-01 | 46633.85 |
| rs6687597 | 1 | 154434936 | A | G | 0.39 | 6.20E-01 | 5.00E-03 | 70935 | 1.00E-300 | 0.77 | 3.46E-03 | 2.71E-03 | 326224 | 2.20E-01 | 15351.21 |
| rs6694817 | 1 | 154401972 | T | C | 0.56 | -7.97E-01 | 4.00E-03 | 70932.9 | 1.00E-300 | 0.42 | -2.87E-04 | 2.30E-03 | 326224 | 9.20E-01 | 39740.42 |
| rs6700296 | 1 | 154473660 | T | C | 0.53 | -5.36E-01 | 4.30E-03 | 70934.9 | 1.00E-300 | 0.36 | -3.95E-03 | 2.37E-03 | 326224 | 1.00E-01 | 15537.91 |
| rs72633646 | 1 | 154334683 | A | G | 0.14 | 2.53E-01 | 7.80E-03 | 70935 | 1.75E-230 | 0.15 | 1.48E-03 | 3.19E-03 | 326224 | 6.90E-01 | 1053.75 |
| rs72633650 | 1 | 154360838 | T | C | 0.79 | -3.54E-01 | 8.00E-03 | 70934 | 1.00E-300 | 0.86 | 2.20E-04 | 3.29E-03 | 326224 | 8.80E-01 | 1955.85 |
| rs72698115 | 1 | 154379369 | A | C | 0.82 | 1.79E-01 | 8.80E-03 | 70934 | 1.14E-92 | 0.90 | -1.07E-03 | 3.75E-03 | 326224 | 7.20E-01 | 412.37 |
| rs72698169 | 1 | 154486799 | A | C | 0.78 | -7.11E-01 | 5.50E-03 | 70934.9 | 1.00E-300 | 0.84 | -3.13E-03 | 3.08E-03 | 326224 | 3.30E-01 | 16697.34 |
| rs73018293 | 1 | 154465577 | T | C | 0.20 | -5.86E-01 | 5.30E-03 | 70934.9 | 1.00E-300 | 0.19 | -7.33E-03 | 2.87E-03 | 326224 | 1.20E-02 | 12233.19 |
| rs73020232 | 1 | 154482669 | T | C | 0.20 | -5.86E-01 | 5.30E-03 | 70934.9 | 1.00E-300 | 0.19 | -7.28E-03 | 2.87E-03 | 326224 | 1.20E-02 | 12237.37 |
| rs73020246 | 1 | 154485640 | A | G | 0.77 | 5.87E-01 | 5.30E-03 | 70934.9 | 1.00E-300 | 0.81 | 7.27E-03 | 2.87E-03 | 326224 | 1.20E-02 | 12258.25 |
| rs7513603 | 1 | 154481158 | T | C | 0.46 | -2.13E-01 | 6.90E-03 | 70934.9 | 5.71E-206 | 0.16 | 1.82E-03 | 3.08E-03 | 326224 | 5.40E-01 | 951.14 |
| rs7519499 | 1 | 154487926 | A | G | 0.46 | 5.36E-01 | 4.30E-03 | 70934.9 | 1.00E-300 | 0.64 | 4.02E-03 | 2.37E-03 | 326224 | 9.70E-02 | 15555.31 |
| rs7521458 | 1 | 154407713 | T | C | 0.57 | -9.71E-01 | 3.50E-03 | 70934.9 | 1.00E-300 | 0.58 | -5.64E-04 | 2.30E-03 | 326224 | 8.50E-01 | 76966.61 |
| rs7525477 | 1 | 154394297 | A | G | 0.42 | -3.30E-01 | 5.80E-03 | 69968.8 | 1.00E-300 | 0.44 | -3.23E-04 | 2.39E-03 | 326224 | 8.50E-01 | 3245.07 |
| rs7526131 | 1 | 154425135 | A | G | 0.44 | 8.44E-01 | 3.90E-03 | 70935 | 1.00E-300 | 0.58 | 1.35E-03 | 2.29E-03 | 326224 | 5.70E-01 | 46811.21 |
| rs7537291 | 1 | 154433407 | A | G | 0.39 | 6.21E-01 | 5.00E-03 | 70935 | 1.00E-300 | 0.77 | 3.50E-03 | 2.71E-03 | 326224 | 2.10E-01 | 15410.74 |
| rs7549250 | 1 | 154404336 | T | C | 0.44 | 8.26E-01 | 3.90E-03 | 70933 | 1.00E-300 | 0.58 | 1.06E-03 | 2.29E-03 | 326224 | 6.60E-01 | 44878.79 |
| rs7553271 | 1 | 154373231 | T | C | 0.49 | 1.26E-01 | 5.70E-03 | 70933.9 | 1.13E-108 | 0.40 | -3.10E-03 | 2.32E-03 | 326224 | 2.00E-01 | 487.87 |
| rs79438587 | 1 | 154342517 | T | C | 0.16 | 3.48E-01 | 7.70E-03 | 69969.9 | 1.00E-300 | 0.17 | 1.14E-03 | 3.09E-03 | 326224 | 7.10E-01 | 2036.71 |
| rs79794939 | 1 | 154390932 | T | C | 0.07 | -3.75E-01 | 1.06E-02 | 70930 | 5.13E-272 | 0.08 | 7.93E-03 | 4.23E-03 | 326224 | 5.90E-02 | 1248.89 |
| rs9651053 | 1 | 154359411 | A | G | 0.11 | -1.66E-01 | 8.70E-03 | 70934 | 4.13E-82 | 0.10 | 2.32E-04 | 3.72E-03 | 326224 | 9.00E-01 | 365.82 |
| rs9803896 | 1 | 154347450 | A | G | 0.14 | 2.74E-01 | 7.70E-03 | 70935 | 7.89E-275 | 0.15 | 8.93E-04 | 3.16E-03 | 326224 | 8.30E-01 | 1269.95 |

Instrumental cis SNPs (50K) select for CRP to Sleep classical Mendelian randomization analysis. CHR: chromosome; POS: hg19 genomic position; A1: effective allele; A2: the other allele; A1_exp_frq: A1 frequency in the exposure dataset; Beta_exp, effect of A1 for the exposure; SE_exp; standard error for Beta_exp; N_exp: sample size for exposure; P_exp, association p value for exposure; A1_out_frq: A1 frequency in the outcome dataset; Beta_out: effect size of A1 on outcome; SE_out: standard error of Beta_out; N_out: sample size for outcome; P_out: association p value for outcome; F-stats: F-statistics.

# **Table S10. Harmonized instrumental cisSNPs (p<5x10^-8^) for sgp130 to Short Sleep MR.**

| SNP | CHR | POS | A1 | A2 | A1_exp_frq | Beta_exp | SE_exp | N_exp | P_exp | A1_out_frq | Beta_out | SE_out | N_out | P_out | Fstats |
| --- | --- | --- | --- | --- | --- | --- | --- | --- | --- | --- | --- | --- | --- | --- | --- |
| rs10043068 | 5 | 55201091 | A | G | 0.18 | 8.37E-02 | 9.20E-03 | 45876 | 9.03E-20 | 0.17 | -2.54E-03 | 1.28E-03 | 326224 | 5.90E-02 | 82.77 |
| rs10045084 | 5 | 55333925 | A | G | 0.42 | 7.71E-02 | 7.40E-03 | 45875 | 2.96E-25 | 0.66 | -9.38E-04 | 1.01E-03 | 326224 | 3.60E-01 | 108.55 |
| rs10056283 | 5 | 55330467 | T | C | 0.45 | 1.03E-01 | 7.20E-03 | 45875 | 2.16E-46 | 0.47 | -2.15E-04 | 9.66E-04 | 326224 | 8.00E-01 | 203.06 |
| rs10075152 | 5 | 55284949 | T | C | 0.28 | -7.86E-02 | 7.90E-03 | 45875 | 3.81E-23 | 0.28 | -1.06E-03 | 1.06E-03 | 326224 | 3.10E-01 | 98.99 |
| rs10214033 | 5 | 55327306 | A | G | 0.43 | 9.48E-02 | 7.20E-03 | 45875 | 1.68E-39 | 0.45 | 1.03E-04 | 9.69E-04 | 326224 | 9.40E-01 | 173.36 |
| rs10471419 | 5 | 55251223 | T | C | 0.81 | -3.95E-01 | 1.04E-02 | 45875 | 1.00E-300 | 0.86 | 2.85E-03 | 1.40E-03 | 326224 | 4.40E-02 | 1444.73 |
| rs10805486 | 5 | 55183662 | A | G | 0.47 | 5.00E-02 | 7.20E-03 | 45877 | 2.85E-12 | 0.50 | -2.98E-03 | 9.62E-04 | 326224 | 2.80E-03 | 48.23 |
| rs10940492 | 5 | 55235425 | T | C | 0.82 | -4.16E-01 | 1.12E-02 | 45876 | 1.00E-300 | 0.88 | 3.53E-03 | 1.50E-03 | 326224 | 2.00E-02 | 1381.58 |
| rs1144496 | 5 | 55210985 | T | C | 0.45 | -4.75E-02 | 7.20E-03 | 45876 | 5.04E-11 | 0.56 | 5.09E-04 | 9.66E-04 | 326224 | 6.00E-01 | 43.52 |
| rs11574762 | 5 | 55293271 | T | C | 0.85 | 7.83E-02 | 1.32E-02 | 45875 | 3.01E-09 | 0.93 | -7.19E-04 | 1.87E-03 | 326224 | 6.90E-01 | 35.19 |
| rs11574765 | 5 | 55278967 | A | G | 0.82 | -4.24E-01 | 1.12E-02 | 45875 | 1.00E-300 | 0.88 | 3.44E-03 | 1.50E-03 | 326224 | 2.30E-02 | 1431.81 |
| rs11574769 | 5 | 55258221 | T | C | 0.82 | -4.23E-01 | 1.12E-02 | 45875 | 1.00E-300 | 0.88 | 3.45E-03 | 1.50E-03 | 326224 | 2.30E-02 | 1426.41 |
| rs11574777 | 5 | 55248943 | A | C | 0.11 | 4.22E-01 | 1.12E-02 | 45875 | 1.00E-300 | 0.12 | -3.44E-03 | 1.50E-03 | 326224 | 2.30E-02 | 1419.67 |
| rs11739048 | 5 | 55263495 | T | C | 0.13 | 4.00E-01 | 1.04E-02 | 45875 | 1.00E-300 | 0.13 | -2.91E-03 | 1.41E-03 | 326224 | 4.10E-02 | 1476.33 |
| rs11740906 | 5 | 55319195 | A | G | 0.12 | 3.78E-01 | 1.06E-02 | 45875 | 3.21E-276 | 0.14 | -2.91E-03 | 1.41E-03 | 326224 | 4.30E-02 | 1271.66 |
| rs11741161 | 5 | 55314248 | C | G | 0.12 | 4.00E-01 | 1.10E-02 | 45875 | 1.50E-290 | 0.12 | -3.04E-03 | 1.47E-03 | 326224 | 4.40E-02 | 1322.98 |
| rs11741953 | 5 | 55267814 | T | C | 0.82 | -4.23E-01 | 1.08E-02 | 45875 | 1.00E-300 | 0.88 | 3.27E-03 | 1.48E-03 | 326224 | 3.00E-02 | 1534.75 |
| rs11742754 | 5 | 55298493 | T | C | 0.83 | -4.16E-01 | 1.15E-02 | 45875 | 1.29E-284 | 0.89 | 3.26E-03 | 1.54E-03 | 326224 | 3.60E-02 | 1309.81 |
| rs11747625 | 5 | 55276273 | T | G | 0.11 | 4.23E-01 | 1.12E-02 | 45875 | 1.00E-300 | 0.12 | -3.46E-03 | 1.50E-03 | 326224 | 2.30E-02 | 1429.11 |
| rs11953360 | 5 | 55324944 | A | C | 0.74 | -1.96E-01 | 8.70E-03 | 45875 | 1.04E-112 | 0.78 | 1.22E-03 | 1.16E-03 | 326224 | 3.00E-01 | 505.99 |
| rs12514537 | 5 | 55339462 | T | C | 0.44 | 8.58E-02 | 7.20E-03 | 45875 | 9.63E-33 | 0.45 | -1.76E-04 | 9.73E-04 | 326224 | 8.40E-01 | 142.01 |
| rs13170520 | 5 | 55273842 | T | C | 0.82 | -4.24E-01 | 1.12E-02 | 45875 | 1.00E-300 | 0.88 | 3.45E-03 | 1.50E-03 | 326224 | 2.30E-02 | 1430.46 |
| rs13179290 | 5 | 55302841 | T | C | 0.13 | 4.00E-01 | 1.05E-02 | 45875 | 1.00E-300 | 0.13 | -2.99E-03 | 1.41E-03 | 326224 | 3.60E-02 | 1449.07 |
| rs13182872 | 5 | 55228754 | T | C | 0.82 | -4.12E-01 | 1.11E-02 | 45875 | 1.00E-300 | 0.88 | 3.33E-03 | 1.50E-03 | 326224 | 2.80E-02 | 1379.02 |
| rs13183319 | 5 | 55208557 | A | G | 0.26 | -6.30E-02 | 8.20E-03 | 45876 | 1.16E-14 | 0.26 | 1.10E-03 | 1.09E-03 | 326224 | 3.20E-01 | 59.03 |
| rs13354596 | 5 | 55213858 | T | G | 0.74 | -8.23E-02 | 8.70E-03 | 45876 | 2.73E-21 | 0.79 | 1.35E-03 | 1.17E-03 | 326224 | 2.80E-01 | 89.49 |
| rs1373998 | 5 | 55255565 | A | G | 0.13 | 4.00E-01 | 1.04E-02 | 45875 | 1.00E-300 | 0.13 | -2.91E-03 | 1.41E-03 | 326224 | 4.10E-02 | 1476.33 |
| rs149027249 | 5 | 55227430 | A | G | 0.07 | -7.99E-02 | 1.38E-02 | 45875 | 6.56E-09 | 0.07 | 5.16E-04 | 1.90E-03 | 326224 | 7.70E-01 | 33.52 |
| rs161649 | 5 | 55190030 | T | C | 0.47 | 4.95E-02 | 7.20E-03 | 45877 | 4.83E-12 | 0.50 | -3.02E-03 | 9.62E-04 | 326224 | 2.40E-03 | 47.27 |
| rs166233 | 5 | 55186121 | T | C | 0.47 | 4.99E-02 | 7.20E-03 | 45877 | 3.31E-12 | 0.50 | -3.02E-03 | 9.62E-04 | 326224 | 2.50E-03 | 48.03 |
| rs2112979 | 5 | 55292036 | A | G | 0.68 | 7.81E-02 | 7.90E-03 | 45875 | 6.99E-23 | 0.72 | 1.12E-03 | 1.06E-03 | 326224 | 2.80E-01 | 97.73 |
| rs254987 | 5 | 55193329 | A | G | 0.54 | -6.34E-02 | 7.20E-03 | 45876 | 1.38E-18 | 0.52 | 2.14E-03 | 9.82E-04 | 326224 | 3.70E-02 | 77.54 |
| rs324995 | 5 | 55225328 | T | G | 0.49 | 4.36E-02 | 7.20E-03 | 45875 | 1.17E-09 | 0.44 | -1.02E-03 | 9.73E-04 | 326224 | 3.00E-01 | 36.67 |
| rs34954805 | 5 | 55212356 | A | G | 0.26 | -6.24E-02 | 8.20E-03 | 45876 | 2.19E-14 | 0.26 | 1.01E-03 | 1.10E-03 | 326224 | 3.60E-01 | 57.91 |
| rs62361958 | 5 | 55215989 | A | G | 0.14 | 1.59E-01 | 1.02E-02 | 45876 | 6.41E-55 | 0.14 | -2.77E-03 | 1.40E-03 | 326224 | 6.10E-02 | 241.77 |
| rs62363861 | 5 | 55223755 | A | G | 0.08 | 3.94E-01 | 1.31E-02 | 45875 | 5.96E-198 | 0.06 | -4.10E-03 | 1.99E-03 | 326224 | 4.30E-02 | 903.21 |
| rs62363863 | 5 | 55226939 | T | C | 0.10 | 3.94E-01 | 1.21E-02 | 45875 | 3.90E-233 | 0.07 | -4.45E-03 | 1.90E-03 | 326224 | 2.10E-02 | 1057.59 |
| rs62363895 | 5 | 55254536 | A | G | 0.81 | -3.95E-01 | 1.04E-02 | 45875 | 1.00E-300 | 0.86 | 2.81E-03 | 1.40E-03 | 326224 | 4.80E-02 | 1444.00 |
| rs6450357 | 5 | 55269996 | T | C | 0.82 | -4.23E-01 | 1.08E-02 | 45875 | 1.00E-300 | 0.88 | 3.27E-03 | 1.48E-03 | 326224 | 2.90E-02 | 1535.48 |
| rs6450361 | 5 | 55300526 | T | C | 0.13 | 4.00E-01 | 1.05E-02 | 45875 | 1.00E-300 | 0.13 | -2.91E-03 | 1.41E-03 | 326224 | 4.20E-02 | 1449.07 |
| rs6861772 | 5 | 55271621 | A | G | 0.81 | -3.99E-01 | 1.04E-02 | 45875 | 1.00E-300 | 0.87 | 2.87E-03 | 1.41E-03 | 326224 | 4.40E-02 | 1472.64 |
| rs6863337 | 5 | 55289146 | T | G | 0.81 | -4.00E-01 | 1.05E-02 | 45875 | 1.00E-300 | 0.87 | 2.93E-03 | 1.41E-03 | 326224 | 4.00E-02 | 1451.25 |
| rs6873542 | 5 | 55282618 | T | C | 0.13 | 4.00E-01 | 1.04E-02 | 45875 | 1.00E-300 | 0.13 | -2.92E-03 | 1.41E-03 | 326224 | 4.00E-02 | 1477.81 |
| rs6891628 | 5 | 55296538 | C | G | 0.13 | 4.00E-01 | 1.04E-02 | 45875 | 1.00E-300 | 0.13 | -2.98E-03 | 1.41E-03 | 326224 | 3.60E-02 | 1475.59 |
| rs7713750 | 5 | 55306073 | A | G | 0.13 | 3.99E-01 | 1.05E-02 | 45875 | 1.00E-300 | 0.13 | -2.99E-03 | 1.41E-03 | 326224 | 3.60E-02 | 1442.55 |
| rs7714146 | 5 | 55217758 | T | C | 0.08 | -7.60E-02 | 1.32E-02 | 45876 | 8.93E-09 | 0.08 | 1.14E-03 | 1.76E-03 | 326224 | 5.10E-01 | 33.15 |
| rs7726239 | 5 | 55295306 | A | C | 0.12 | 4.23E-01 | 1.08E-02 | 45875 | 1.00E-300 | 0.12 | -3.33E-03 | 1.48E-03 | 326224 | 2.60E-02 | 1533.30 |
| rs7728232 | 5 | 55220344 | A | G | 0.35 | 8.63E-02 | 7.50E-03 | 45876 | 1.09E-30 | 0.34 | -9.65E-04 | 1.04E-03 | 326224 | 3.60E-01 | 132.40 |
| rs7730934 | 5 | 55266512 | A | G | 0.12 | 4.23E-01 | 1.07E-02 | 45875 | 1.00E-300 | 0.12 | -3.27E-03 | 1.48E-03 | 326224 | 2.90E-02 | 1559.88 |
| rs77847765 | 5 | 55220580 | A | G | 0.16 | 1.34E-01 | 9.60E-03 | 45876 | 4.44E-44 | 0.14 | -1.82E-03 | 1.39E-03 | 326224 | 2.20E-01 | 193.67 |
| rs78443884 | 5 | 55221681 | C | G | 0.07 | 3.93E-01 | 1.40E-02 | 45876 | 7.59E-174 | 0.06 | -4.51E-03 | 2.03E-03 | 326224 | 2.90E-02 | 786.00 |
| rs78842467 | 5 | 55323891 | A | G | 0.74 | -1.95E-01 | 8.70E-03 | 45875 | 6.22E-112 | 0.78 | 1.11E-03 | 1.16E-03 | 326224 | 3.40E-01 | 500.83 |
| rs9292108 | 5 | 55328578 | A | G | 0.55 | -1.03E-01 | 7.20E-03 | 45875 | 1.99E-46 | 0.53 | 2.02E-04 | 9.66E-04 | 326224 | 8.10E-01 | 203.46 |
| rs9632389 | 5 | 55204187 | T | G | 0.76 | -8.30E-02 | 9.20E-03 | 45877 | 1.56E-19 | 0.83 | 2.69E-03 | 1.27E-03 | 326224 | 4.40E-02 | 81.39 |

Instrumental cis SNPs (50K) select for CRP to Sleep classical Mendelian randomization analysis. CHR: chromosome; POS: hg19 genomic position; A1: effective allele; A2: the other allele; A1_exp_frq: A1 frequency in the exposure dataset; Beta_exp, effect of A1 for the exposure; SE_exp; standard error for Beta_exp; N_exp: sample size for exposure; P_exp, association p value for exposure; A1_out_frq: A1 frequency in the outcome dataset; Beta_out: effect size of A1 on outcome; SE_out: standard error of Beta_out; N_out: sample size for outcome; P_out: association p value for outcome; F-stats: F-statistics.

# **Table S11. Harmonized instrumental cisSNPs (p<5x10^-8^) for sgp130 to Long Sleep MR.**

| SNP | CHR | POS | A1 | A2 | A1_exp_frq | Beta_exp | SE_exp | N_exp | P_exp | A1_out_frq | Beta_out | SE_out | N_out | P_out | Fstats |
| --- | --- | --- | --- | --- | --- | --- | --- | --- | --- | --- | --- | --- | --- | --- | --- |
| rs10043068 | 5 | 55201091 | A | G | 0.18 | 8.37E-02 | 9.20E-03 | 45876 | 9.03E-20 | 0.17 | -5.62E-04 | 9.65E-04 | 326224 | 5.80E-01 | 82.77044 |
| rs10045084 | 5 | 55333925 | A | G | 0.42 | 7.71E-02 | 7.40E-03 | 45875 | 2.96E-25 | 0.66 | -9.29E-05 | 7.66E-04 | 326224 | 9.00E-01 | 108.5539 |
| rs10056283 | 5 | 55330467 | T | C | 0.45 | 1.03E-01 | 7.20E-03 | 45875 | 2.16E-46 | 0.47 | -5.17E-04 | 7.32E-04 | 326224 | 4.90E-01 | 203.0625 |
| rs10075152 | 5 | 55284949 | T | C | 0.28 | -7.86E-02 | 7.90E-03 | 45875 | 3.81E-23 | 0.28 | -3.15E-04 | 8.06E-04 | 326224 | 6.80E-01 | 98.98991 |
| rs10214033 | 5 | 55327306 | A | G | 0.43 | 9.48E-02 | 7.20E-03 | 45875 | 1.68E-39 | 0.45 | 2.23E-05 | 7.35E-04 | 326224 | 9.60E-01 | 173.3611 |
| rs10471419 | 5 | 55251223 | T | C | 0.81 | -3.95E-01 | 1.04E-02 | 45875 | 1.00E-300 | 0.86 | 2.35E-04 | 1.06E-03 | 326224 | 8.40E-01 | 1444.731 |
| rs10805486 | 5 | 55183662 | A | G | 0.47 | 5.00E-02 | 7.20E-03 | 45877 | 2.85E-12 | 0.51 | -5.35E-04 | 7.29E-04 | 326224 | 4.80E-01 | 48.22531 |
| rs10940492 | 5 | 55235425 | T | C | 0.82 | -4.16E-01 | 1.12E-02 | 45876 | 1.00E-300 | 0.88 | -1.01E-04 | 1.13E-03 | 326224 | 9.00E-01 | 1381.582 |
| rs1144496 | 5 | 55210985 | T | C | 0.45 | -4.75E-02 | 7.20E-03 | 45876 | 5.04E-11 | 0.56 | -1.38E-04 | 7.31E-04 | 326224 | 8.40E-01 | 43.52334 |
| rs11574762 | 5 | 55293271 | T | C | 0.85 | 7.83E-02 | 1.32E-02 | 45875 | 3.01E-09 | 0.93 | -1.73E-03 | 1.42E-03 | 326224 | 2.10E-01 | 35.18647 |
| rs11574765 | 5 | 55278967 | A | G | 0.82 | -4.24E-01 | 1.12E-02 | 45875 | 1.00E-300 | 0.88 | -1.45E-04 | 1.13E-03 | 326224 | 8.70E-01 | 1431.812 |
| rs11574769 | 5 | 55258221 | T | C | 0.82 | -4.23E-01 | 1.12E-02 | 45875 | 1.00E-300 | 0.88 | -1.77E-04 | 1.13E-03 | 326224 | 8.50E-01 | 1426.411 |
| rs11574777 | 5 | 55248943 | A | C | 0.11 | 4.22E-01 | 1.12E-02 | 45875 | 1.00E-300 | 0.12 | 1.81E-04 | 1.13E-03 | 326224 | 8.50E-01 | 1419.675 |
| rs11739048 | 5 | 55263495 | T | C | 0.13 | 4.00E-01 | 1.04E-02 | 45875 | 1.00E-300 | 0.13 | -1.46E-04 | 1.06E-03 | 326224 | 9.00E-01 | 1476.333 |
| rs11740906 | 5 | 55319195 | A | G | 0.12 | 3.78E-01 | 1.06E-02 | 45875 | 3.21E-276 | 0.14 | -2.78E-04 | 1.06E-03 | 326224 | 8.10E-01 | 1271.663 |
| rs11741161 | 5 | 55314248 | C | G | 0.12 | 4.00E-01 | 1.10E-02 | 45875 | 1.50E-290 | 0.12 | 3.06E-04 | 1.11E-03 | 326224 | 7.60E-01 | 1322.975 |
| rs11741953 | 5 | 55267814 | T | C | 0.82 | -4.23E-01 | 1.08E-02 | 45875 | 1.00E-300 | 0.88 | -3.69E-04 | 1.12E-03 | 326224 | 7.20E-01 | 1534.753 |
| rs11742754 | 5 | 55298493 | T | C | 0.83 | -4.16E-01 | 1.15E-02 | 45875 | 1.29E-284 | 0.89 | 5.32E-05 | 1.17E-03 | 326224 | 9.90E-01 | 1309.811 |
| rs11747625 | 5 | 55276273 | T | G | 0.11 | 4.23E-01 | 1.12E-02 | 45875 | 1.00E-300 | 0.12 | 1.46E-04 | 1.13E-03 | 326224 | 8.70E-01 | 1429.11 |
| rs11953360 | 5 | 55324944 | A | C | 0.74 | -1.96E-01 | 8.70E-03 | 45875 | 1.04E-112 | 0.78 | -9.99E-05 | 8.77E-04 | 326224 | 8.90E-01 | 505.9914 |
| rs12514537 | 5 | 55339462 | T | C | 0.44 | 8.58E-02 | 7.20E-03 | 45875 | 9.63E-33 | 0.45 | -2.09E-04 | 7.37E-04 | 326224 | 8.00E-01 | 142.0069 |
| rs13170520 | 5 | 55273842 | T | C | 0.82 | -4.24E-01 | 1.12E-02 | 45875 | 1.00E-300 | 0.88 | -1.61E-04 | 1.13E-03 | 326224 | 8.60E-01 | 1430.46 |
| rs13179290 | 5 | 55302841 | T | C | 0.13 | 4.00E-01 | 1.05E-02 | 45875 | 1.00E-300 | 0.13 | -2.15E-04 | 1.07E-03 | 326224 | 8.50E-01 | 1449.071 |
| rs13182872 | 5 | 55228754 | T | C | 0.82 | -4.12E-01 | 1.11E-02 | 45875 | 1.00E-300 | 0.88 | -1.44E-04 | 1.13E-03 | 326224 | 8.80E-01 | 1379.018 |
| rs13183319 | 5 | 55208557 | A | G | 0.26 | -6.30E-02 | 8.20E-03 | 45876 | 1.16E-14 | 0.26 | -2.46E-04 | 8.29E-04 | 326224 | 7.60E-01 | 59.02736 |
| rs13354596 | 5 | 55213858 | T | G | 0.74 | -8.23E-02 | 8.70E-03 | 45876 | 2.73E-21 | 0.79 | -4.08E-04 | 8.88E-04 | 326224 | 6.30E-01 | 89.48725 |
| rs1373998 | 5 | 55255565 | A | G | 0.13 | 4.00E-01 | 1.04E-02 | 45875 | 1.00E-300 | 0.13 | -1.27E-04 | 1.06E-03 | 326224 | 9.20E-01 | 1476.333 |
| rs149027249 | 5 | 55227430 | A | G | 0.07 | -7.99E-02 | 1.38E-02 | 45875 | 6.56E-09 | 0.07 | 2.24E-03 | 1.44E-03 | 326224 | 1.20E-01 | 33.52242 |
| rs161649 | 5 | 55190030 | T | C | 0.47 | 4.95E-02 | 7.20E-03 | 45877 | 4.83E-12 | 0.51 | -5.12E-04 | 7.29E-04 | 326224 | 5.00E-01 | 47.26563 |
| rs166233 | 5 | 55186121 | T | C | 0.47 | 4.99E-02 | 7.20E-03 | 45877 | 3.31E-12 | 0.51 | -5.56E-04 | 7.29E-04 | 326224 | 4.60E-01 | 48.0326 |
| rs2112979 | 5 | 55292036 | A | G | 0.68 | 7.81E-02 | 7.90E-03 | 45875 | 6.99E-23 | 0.72 | 3.70E-04 | 8.06E-04 | 326224 | 6.30E-01 | 97.7345 |
| rs254987 | 5 | 55193329 | A | G | 0.54 | -6.34E-02 | 7.20E-03 | 45876 | 1.38E-18 | 0.52 | 2.92E-04 | 7.44E-04 | 326224 | 7.20E-01 | 77.53781 |
| rs324995 | 5 | 55225328 | T | G | 0.49 | 4.36E-02 | 7.20E-03 | 45875 | 1.17E-09 | 0.44 | 1.43E-04 | 7.36E-04 | 326224 | 8.30E-01 | 36.66975 |
| rs34954805 | 5 | 55212356 | A | G | 0.26 | -6.24E-02 | 8.20E-03 | 45876 | 2.19E-14 | 0.26 | -2.09E-04 | 8.33E-04 | 326224 | 7.90E-01 | 57.90839 |
| rs62361958 | 5 | 55215989 | A | G | 0.14 | 1.59E-01 | 1.02E-02 | 45876 | 6.41E-55 | 0.14 | -3.15E-05 | 1.06E-03 | 326224 | 1.00E+00 | 241.772 |
| rs62363861 | 5 | 55223755 | A | G | 0.08 | 3.94E-01 | 1.31E-02 | 45875 | 5.96E-198 | 0.06 | 7.33E-05 | 1.50E-03 | 326224 | 9.40E-01 | 903.209 |
| rs62363863 | 5 | 55226939 | T | C | 0.10 | 3.94E-01 | 1.21E-02 | 45875 | 3.90E-233 | 0.07 | -2.33E-04 | 1.44E-03 | 326224 | 9.00E-01 | 1057.593 |
| rs62363895 | 5 | 55254536 | A | G | 0.81 | -3.95E-01 | 1.04E-02 | 45875 | 1.00E-300 | 0.86 | 1.96E-04 | 1.06E-03 | 326224 | 8.70E-01 | 1444 |
| rs6450357 | 5 | 55269996 | T | C | 0.82 | -4.23E-01 | 1.08E-02 | 45875 | 1.00E-300 | 0.88 | -3.67E-04 | 1.12E-03 | 326224 | 7.20E-01 | 1535.479 |
| rs6450361 | 5 | 55300526 | T | C | 0.13 | 4.00E-01 | 1.05E-02 | 45875 | 1.00E-300 | 0.13 | -1.90E-04 | 1.07E-03 | 326224 | 8.70E-01 | 1449.071 |
| rs6861772 | 5 | 55271621 | A | G | 0.81 | -3.99E-01 | 1.04E-02 | 45875 | 1.00E-300 | 0.87 | 1.78E-04 | 1.06E-03 | 326224 | 8.80E-01 | 1472.641 |
| rs6863337 | 5 | 55289146 | T | G | 0.81 | -4.00E-01 | 1.05E-02 | 45875 | 1.00E-300 | 0.87 | 1.81E-04 | 1.07E-03 | 326224 | 8.80E-01 | 1451.247 |
| rs6873542 | 5 | 55282618 | T | C | 0.13 | 4.00E-01 | 1.04E-02 | 45875 | 1.00E-300 | 0.13 | -1.68E-04 | 1.07E-03 | 326224 | 8.90E-01 | 1477.811 |
| rs6891628 | 5 | 55296538 | C | G | 0.13 | 4.00E-01 | 1.04E-02 | 45875 | 1.00E-300 | 0.13 | -1.99E-04 | 1.06E-03 | 326224 | 8.60E-01 | 1475.594 |
| rs7713750 | 5 | 55306073 | A | G | 0.13 | 3.99E-01 | 1.05E-02 | 45875 | 1.00E-300 | 0.13 | -2.08E-04 | 1.07E-03 | 326224 | 8.60E-01 | 1442.553 |
| rs7714146 | 5 | 55217758 | T | C | 0.08 | -7.60E-02 | 1.32E-02 | 45876 | 8.93E-09 | 0.08 | 2.11E-03 | 1.33E-03 | 326224 | 1.10E-01 | 33.14968 |
| rs7726239 | 5 | 55295306 | A | C | 0.12 | 4.23E-01 | 1.08E-02 | 45875 | 1.00E-300 | 0.12 | 3.37E-04 | 1.12E-03 | 326224 | 7.40E-01 | 1533.303 |
| rs7728232 | 5 | 55220344 | A | G | 0.35 | 8.63E-02 | 7.50E-03 | 45876 | 1.09E-30 | 0.34 | -3.14E-04 | 7.86E-04 | 326224 | 6.90E-01 | 132.4034 |
| rs7730934 | 5 | 55266512 | A | G | 0.12 | 4.23E-01 | 1.07E-02 | 45875 | 1.00E-300 | 0.12 | 3.79E-04 | 1.12E-03 | 326224 | 7.10E-01 | 1559.881 |
| rs77847765 | 5 | 55220580 | A | G | 0.16 | 1.34E-01 | 9.60E-03 | 45876 | 4.44E-44 | 0.14 | 9.97E-04 | 1.05E-03 | 326224 | 3.30E-01 | 193.6736 |
| rs78443884 | 5 | 55221681 | C | G | 0.07 | 3.93E-01 | 1.40E-02 | 45876 | 7.59E-174 | 0.06 | -3.81E-04 | 1.53E-03 | 326224 | 8.30E-01 | 786.0013 |
| rs78842467 | 5 | 55323891 | A | G | 0.74 | -1.95E-01 | 8.70E-03 | 45875 | 6.22E-112 | 0.78 | -2.09E-04 | 8.75E-04 | 326224 | 7.90E-01 | 500.8335 |
| rs9292108 | 5 | 55328578 | A | G | 0.55 | -1.03E-01 | 7.20E-03 | 45875 | 1.99E-46 | 0.53 | 5.09E-04 | 7.32E-04 | 326224 | 5.00E-01 | 203.4585 |
| rs9632389 | 5 | 55204187 | T | G | 0.76 | -8.30E-02 | 9.20E-03 | 45877 | 1.56E-19 | 0.83 | 5.94E-04 | 9.64E-04 | 326224 | 5.60E-01 | 81.39178 |

Instrumental cis SNPs (50K) select for CRP to Sleep classical Mendelian randomization analysis. CHR: chromosome; POS: hg19 genomic position; A1: effective allele; A2: the other allele; A1_exp_frq: A1 frequency in the exposure dataset; Beta_exp, effect of A1 for the exposure; SE_exp; standard error for Beta_exp; N_exp: sample size for exposure; P_exp, association p value for exposure; A1_out_frq: A1 frequency in the outcome dataset; Beta_out: effect size of A1 on outcome; SE_out: standard error of Beta_out; N_out: sample size for outcome; P_out: association p value for outcome; F-stats: F-statistics.

# **Table S12. Harmonized instrumental cisSNPs (p<5x10^-8^) for sgp130 to Sleep Duration MR.**

| SNP | CHR | POS | A1 | A2 | A1_exp_frq | Beta_exp | SE_exp | N_exp | P_exp | A1_out_frq | Beta_out | SE_out | N_out | P_out | Fstats |
| --- | --- | --- | --- | --- | --- | --- | --- | --- | --- | --- | --- | --- | --- | --- | --- |
| rs10043068 | 5 | 55201091 | A | G | 0.18 | 8.37E-02 | 9.20E-03 | 45876 | 9.03E-20 | 0.17 | 6.19E-03 | 3.01E-03 | 326224 | 3.60E-02 | 82.77 |
| rs10045084 | 5 | 55333925 | A | G | 0.42 | 7.71E-02 | 7.40E-03 | 45875 | 2.96E-25 | 0.66 | 2.80E-03 | 2.39E-03 | 326224 | 2.70E-01 | 108.55 |
| rs10056283 | 5 | 55330467 | T | C | 0.45 | 1.03E-01 | 7.20E-03 | 45875 | 2.16E-46 | 0.47 | -6.18E-04 | 2.28E-03 | 326224 | 7.80E-01 | 203.06 |
| rs10075152 | 5 | 55284949 | T | C | 0.28 | -7.86E-02 | 7.90E-03 | 45875 | 3.81E-23 | 0.28 | 9.24E-04 | 2.51E-03 | 326224 | 7.00E-01 | 98.99 |
| rs10214033 | 5 | 55327306 | A | G | 0.43 | 9.48E-02 | 7.20E-03 | 45875 | 1.68E-39 | 0.45 | 1.93E-04 | 2.29E-03 | 326224 | 9.20E-01 | 173.36 |
| rs10471419 | 5 | 55251223 | T | C | 0.81 | -3.95E-01 | 1.04E-02 | 45875 | 1.00E-300 | 0.86 | -8.52E-03 | 3.31E-03 | 326224 | 9.20E-03 | 1444.73 |
| rs10805486 | 5 | 55183662 | A | G | 0.47 | 5.00E-02 | 7.20E-03 | 45877 | 2.85E-12 | 0.50 | 6.08E-03 | 2.27E-03 | 326224 | 6.70E-03 | 48.23 |
| rs10940492 | 5 | 55235425 | T | C | 0.82 | -4.16E-01 | 1.12E-02 | 45876 | 1.00E-300 | 0.88 | -1.11E-02 | 3.53E-03 | 326224 | 1.50E-03 | 1381.58 |
| rs1144496 | 5 | 55210985 | T | C | 0.45 | -4.75E-02 | 7.20E-03 | 45876 | 5.04E-11 | 0.56 | -2.03E-03 | 2.28E-03 | 326224 | 3.80E-01 | 43.52 |
| rs11574762 | 5 | 55293271 | T | C | 0.85 | 7.83E-02 | 1.32E-02 | 45875 | 3.01E-09 | 0.93 | -1.32E-03 | 4.41E-03 | 326224 | 7.80E-01 | 35.19 |
| rs11574765 | 5 | 55278967 | A | G | 0.82 | -4.24E-01 | 1.12E-02 | 45875 | 1.00E-300 | 0.88 | -1.12E-02 | 3.54E-03 | 326224 | 1.30E-03 | 1431.81 |
| rs11574769 | 5 | 55258221 | T | C | 0.82 | -4.23E-01 | 1.12E-02 | 45875 | 1.00E-300 | 0.88 | -1.12E-02 | 3.54E-03 | 326224 | 1.40E-03 | 1426.41 |
| rs11574777 | 5 | 55248943 | A | C | 0.11 | 4.22E-01 | 1.12E-02 | 45875 | 1.00E-300 | 0.12 | 1.11E-02 | 3.54E-03 | 326224 | 1.40E-03 | 1419.67 |
| rs11739048 | 5 | 55263495 | T | C | 0.13 | 4.00E-01 | 1.04E-02 | 45875 | 1.00E-300 | 0.13 | 8.86E-03 | 3.32E-03 | 326224 | 7.20E-03 | 1476.33 |
| rs11740906 | 5 | 55319195 | A | G | 0.12 | 3.78E-01 | 1.06E-02 | 45875 | 3.21E-276 | 0.14 | 8.13E-03 | 3.32E-03 | 326224 | 1.40E-02 | 1271.66 |
| rs11741161 | 5 | 55314248 | C | G | 0.12 | 4.00E-01 | 1.10E-02 | 45875 | 1.50E-290 | 0.12 | 1.02E-02 | 3.48E-03 | 326224 | 3.00E-03 | 1322.98 |
| rs11741953 | 5 | 55267814 | T | C | 0.82 | -4.23E-01 | 1.08E-02 | 45875 | 1.00E-300 | 0.88 | -1.12E-02 | 3.50E-03 | 326224 | 1.20E-03 | 1534.75 |
| rs11742754 | 5 | 55298493 | T | C | 0.83 | -4.16E-01 | 1.15E-02 | 45875 | 1.29E-284 | 0.89 | -1.06E-02 | 3.64E-03 | 326224 | 3.20E-03 | 1309.81 |
| rs11747625 | 5 | 55276273 | T | G | 0.11 | 4.23E-01 | 1.12E-02 | 45875 | 1.00E-300 | 0.12 | 1.12E-02 | 3.54E-03 | 326224 | 1.30E-03 | 1429.11 |
| rs11953360 | 5 | 55324944 | A | C | 0.74 | -1.96E-01 | 8.70E-03 | 45875 | 1.04E-112 | 0.78 | -5.09E-03 | 2.73E-03 | 326224 | 5.70E-02 | 505.99 |
| rs12514537 | 5 | 55339462 | T | C | 0.44 | 8.58E-02 | 7.20E-03 | 45875 | 9.63E-33 | 0.45 | 2.98E-04 | 2.30E-03 | 326224 | 8.90E-01 | 142.01 |
| rs13170520 | 5 | 55273842 | T | C | 0.82 | -4.24E-01 | 1.12E-02 | 45875 | 1.00E-300 | 0.88 | -1.12E-02 | 3.54E-03 | 326224 | 1.30E-03 | 1430.46 |
| rs13179290 | 5 | 55302841 | T | C | 0.13 | 4.00E-01 | 1.05E-02 | 45875 | 1.00E-300 | 0.13 | 8.93E-03 | 3.32E-03 | 326224 | 6.80E-03 | 1449.07 |
| rs13182872 | 5 | 55228754 | T | C | 0.82 | -4.12E-01 | 1.11E-02 | 45875 | 1.00E-300 | 0.88 | -1.08E-02 | 3.53E-03 | 326224 | 1.90E-03 | 1379.02 |
| rs13183319 | 5 | 55208557 | A | G | 0.26 | -6.30E-02 | 8.20E-03 | 45876 | 1.16E-14 | 0.26 | -3.14E-03 | 2.58E-03 | 326224 | 2.10E-01 | 59.03 |
| rs13354596 | 5 | 55213858 | T | G | 0.74 | -8.23E-02 | 8.70E-03 | 45876 | 2.73E-21 | 0.79 | -5.78E-03 | 2.77E-03 | 326224 | 3.60E-02 | 89.49 |
| rs1373998 | 5 | 55255565 | A | G | 0.13 | 4.00E-01 | 1.04E-02 | 45875 | 1.00E-300 | 0.13 | 8.84E-03 | 3.32E-03 | 326224 | 7.30E-03 | 1476.33 |
| rs149027249 | 5 | 55227430 | A | G | 0.07 | -7.99E-02 | 1.38E-02 | 45875 | 6.56E-09 | 0.07 | 2.58E-03 | 4.49E-03 | 326224 | 5.90E-01 | 33.52 |
| rs161649 | 5 | 55190030 | T | C | 0.47 | 4.95E-02 | 7.20E-03 | 45877 | 4.83E-12 | 0.50 | 6.23E-03 | 2.27E-03 | 326224 | 5.60E-03 | 47.27 |
| rs166233 | 5 | 55186121 | T | C | 0.47 | 4.99E-02 | 7.20E-03 | 45877 | 3.31E-12 | 0.50 | 6.10E-03 | 2.27E-03 | 326224 | 6.60E-03 | 48.03 |
| rs2112979 | 5 | 55292036 | A | G | 0.68 | 7.81E-02 | 7.90E-03 | 45875 | 6.99E-23 | 0.72 | -1.03E-03 | 2.51E-03 | 326224 | 6.70E-01 | 97.73 |
| rs254987 | 5 | 55193329 | A | G | 0.54 | -6.34E-02 | 7.20E-03 | 45876 | 1.38E-18 | 0.52 | -4.96E-03 | 2.32E-03 | 326224 | 2.90E-02 | 77.54 |
| rs324995 | 5 | 55225328 | T | G | 0.49 | 4.36E-02 | 7.20E-03 | 45875 | 1.17E-09 | 0.44 | 3.23E-03 | 2.30E-03 | 326224 | 1.50E-01 | 36.67 |
| rs34954805 | 5 | 55212356 | A | G | 0.26 | -6.24E-02 | 8.20E-03 | 45876 | 2.19E-14 | 0.26 | -2.96E-03 | 2.60E-03 | 326224 | 2.40E-01 | 57.91 |
| rs62361958 | 5 | 55215989 | A | G | 0.14 | 1.59E-01 | 1.02E-02 | 45876 | 6.41E-55 | 0.14 | 8.10E-03 | 3.31E-03 | 326224 | 1.30E-02 | 241.77 |
| rs62363861 | 5 | 55223755 | A | G | 0.08 | 3.94E-01 | 1.31E-02 | 45875 | 5.96E-198 | 0.06 | 1.24E-02 | 4.69E-03 | 326224 | 6.60E-03 | 903.21 |
| rs62363863 | 5 | 55226939 | T | C | 0.10 | 3.94E-01 | 1.21E-02 | 45875 | 3.90E-233 | 0.07 | 1.25E-02 | 4.49E-03 | 326224 | 4.40E-03 | 1057.59 |
| rs62363895 | 5 | 55254536 | A | G | 0.81 | -3.95E-01 | 1.04E-02 | 45875 | 1.00E-300 | 0.86 | -8.53E-03 | 3.31E-03 | 326224 | 9.10E-03 | 1444.00 |
| rs6450357 | 5 | 55269996 | T | C | 0.82 | -4.23E-01 | 1.08E-02 | 45875 | 1.00E-300 | 0.88 | -1.12E-02 | 3.50E-03 | 326224 | 1.20E-03 | 1535.48 |
| rs6450361 | 5 | 55300526 | T | C | 0.13 | 4.00E-01 | 1.05E-02 | 45875 | 1.00E-300 | 0.13 | 8.87E-03 | 3.33E-03 | 326224 | 7.20E-03 | 1449.07 |
| rs6861772 | 5 | 55271621 | A | G | 0.81 | -3.99E-01 | 1.04E-02 | 45875 | 1.00E-300 | 0.87 | -8.77E-03 | 3.32E-03 | 326224 | 7.80E-03 | 1472.64 |
| rs6863337 | 5 | 55289146 | T | G | 0.81 | -4.00E-01 | 1.05E-02 | 45875 | 1.00E-300 | 0.87 | -8.89E-03 | 3.32E-03 | 326224 | 7.10E-03 | 1451.25 |
| rs6873542 | 5 | 55282618 | T | C | 0.13 | 4.00E-01 | 1.04E-02 | 45875 | 1.00E-300 | 0.13 | 8.88E-03 | 3.32E-03 | 326224 | 7.10E-03 | 1477.81 |
| rs6891628 | 5 | 55296538 | C | G | 0.13 | 4.00E-01 | 1.04E-02 | 45875 | 1.00E-300 | 0.13 | 8.92E-03 | 3.32E-03 | 326224 | 6.70E-03 | 1475.59 |
| rs7713750 | 5 | 55306073 | A | G | 0.13 | 3.99E-01 | 1.05E-02 | 45875 | 1.00E-300 | 0.13 | 8.95E-03 | 3.33E-03 | 326224 | 6.70E-03 | 1442.55 |
| rs7714146 | 5 | 55217758 | T | C | 0.08 | -7.60E-02 | 1.32E-02 | 45876 | 8.93E-09 | 0.08 | 3.99E-03 | 4.16E-03 | 326224 | 3.70E-01 | 33.15 |
| rs7726239 | 5 | 55295306 | A | C | 0.12 | 4.23E-01 | 1.08E-02 | 45875 | 1.00E-300 | 0.12 | 1.12E-02 | 3.50E-03 | 326224 | 1.10E-03 | 1533.30 |
| rs7728232 | 5 | 55220344 | A | G | 0.35 | 8.63E-02 | 7.50E-03 | 45876 | 1.09E-30 | 0.34 | 2.91E-03 | 2.45E-03 | 326224 | 2.50E-01 | 132.40 |
| rs7730934 | 5 | 55266512 | A | G | 0.12 | 4.23E-01 | 1.07E-02 | 45875 | 1.00E-300 | 0.12 | 1.12E-02 | 3.49E-03 | 326224 | 1.20E-03 | 1559.88 |
| rs77847765 | 5 | 55220580 | A | G | 0.16 | 1.34E-01 | 9.60E-03 | 45876 | 4.44E-44 | 0.14 | 8.75E-03 | 3.28E-03 | 326224 | 7.50E-03 | 193.67 |
| rs78443884 | 5 | 55221681 | C | G | 0.07 | 3.93E-01 | 1.40E-02 | 45876 | 7.59E-174 | 0.06 | 1.21E-02 | 4.79E-03 | 326224 | 9.50E-03 | 786.00 |
| rs78842467 | 5 | 55323891 | A | G | 0.74 | -1.95E-01 | 8.70E-03 | 45875 | 6.22E-112 | 0.78 | -5.10E-03 | 2.73E-03 | 326224 | 5.50E-02 | 500.83 |
| rs9292108 | 5 | 55328578 | A | G | 0.55 | -1.03E-01 | 7.20E-03 | 45875 | 1.99E-46 | 0.53 | 6.42E-04 | 2.28E-03 | 326224 | 7.70E-01 | 203.46 |
| rs9632389 | 5 | 55204187 | T | G | 0.76 | -8.30E-02 | 9.20E-03 | 45877 | 1.56E-19 | 0.83 | -6.28E-03 | 3.01E-03 | 326224 | 3.30E-02 | 81.39 |

Instrumental cis SNPs (50K) select for CRP to Sleep classical Mendelian randomization analysis. CHR: chromosome; POS: hg19 genomic position; A1: effective allele; A2: the other allele; A1_exp_frq: A1 frequency in the exposure dataset; Beta_exp, effect of A1 for the exposure; SE_exp; standard error for Beta_exp; N_exp: sample size for exposure; P_exp, association p value for exposure; A1_out_frq: A1 frequency in the outcome dataset; Beta_out: effect size of A1 on outcome; SE_out: standard error of Beta_out; N_out: sample size for outcome; P_out: association p value for outcome; F-stats: F-statistics.

# **Table S13. Harmonized instrumental cisSNPs (p<5x10^-8^) for CRP in iPsych to Short Sleep MR.**

| SNP | CHR | POS | A1 | A2 | A1_exp_frq | Beta_exp | SE_exp | N_exp | P_exp | A1_out_frq | Beta_out | SE_out | N_out | P_out | Fstats |
| --- | --- | --- | --- | --- | --- | --- | --- | --- | --- | --- | --- | --- | --- | --- | --- |
| rs11265257 | 1 | 159668984 | T | C | 0.39 | -2.16E-01 | 1.82E-02 | 8318 | 3.62E-32 | 0.39 | -4.69E-04 | 9.85E-04 | 326224 | 6.20E-01 |  |
| rs11265264 | 1 | 159712842 | A | G | 0.36 | 2.70E-01 | 1.85E-02 | 8318 | 1.35E-47 | 0.36 | -7.17E-04 | 1.00E-03 | 326224 | 4.90E-01 |  |
| rs1130864 | 1 | 159683091 | A | G | 0.32 | 3.33E-01 | 1.89E-02 | 8318 | 9.41E-68 | 0.31 | -3.14E-04 | 1.04E-03 | 326224 | 7.70E-01 |  |
| rs11588887 | 1 | 159717162 | A | G | 0.16 | -1.77E-01 | 2.50E-02 | 8318 | 1.78E-12 | 0.16 | 4.16E-04 | 1.32E-03 | 326224 | 7.70E-01 |  |
| rs12093699 | 1 | 159647988 | A | G | 0.32 | 3.03E-01 | 1.88E-02 | 8318 | 1.69E-57 | 0.31 | -7.44E-04 | 1.04E-03 | 326224 | 4.90E-01 |  |
| rs12094103 | 1 | 159723619 | A | G | 0.36 | 2.46E-01 | 1.85E-02 | 8318 | 7.20E-40 | 0.35 | -6.68E-04 | 1.01E-03 | 326224 | 5.30E-01 |  |
| rs12726900 | 1 | 159732523 | A | G | 0.37 | 2.48E-01 | 2.00E-02 | 8318 | 5.23E-35 | 0.35 | -1.08E-03 | 1.05E-03 | 326224 | 3.20E-01 |  |
| rs12727021 | 1 | 159702487 | A | G | 0.32 | 3.31E-01 | 1.89E-02 | 8318 | 2.93E-67 | 0.31 | -2.74E-04 | 1.04E-03 | 326224 | 8.00E-01 |  |
| rs12739022 | 1 | 159659431 | T | C | 0.68 | -3.28E-01 | 1.90E-02 | 8318 | 7.59E-66 | 0.69 | 4.17E-04 | 1.04E-03 | 326224 | 7.00E-01 |  |
| rs12754745 | 1 | 159670483 | A | G | 0.32 | 3.30E-01 | 1.90E-02 | 8318 | 1.19E-66 | 0.31 | -4.03E-04 | 1.04E-03 | 326224 | 7.10E-01 |  |
| rs12754915 | 1 | 159660869 | T | C | 0.68 | -3.28E-01 | 1.90E-02 | 8318 | 7.18E-66 | 0.69 | 3.95E-04 | 1.04E-03 | 326224 | 7.20E-01 |  |
| rs12759988 | 1 | 159708751 | A | G | 0.35 | 2.72E-01 | 1.87E-02 | 8318 | 3.36E-47 | 0.33 | -3.75E-04 | 1.02E-03 | 326224 | 7.30E-01 |  |
| rs12760041 | 1 | 159714035 | T | C | 0.35 | 2.70E-01 | 1.87E-02 | 8318 | 1.03E-46 | 0.33 | -3.85E-04 | 1.02E-03 | 326224 | 7.20E-01 |  |
| rs137918962 | 1 | 159652311 | A | G | 0.05 | 2.92E-01 | 4.37E-02 | 8318 | 2.60E-11 | 0.05 | -2.27E-03 | 2.23E-03 | 326224 | 3.10E-01 |  |
| rs1470515 | 1 | 159653599 | T | C | 0.39 | -2.13E-01 | 1.83E-02 | 8318 | 4.19E-31 | 0.39 | -2.29E-04 | 9.87E-04 | 326224 | 8.00E-01 |  |
| rs16842484 | 1 | 159646924 | T | C | 0.72 | -2.94E-01 | 2.02E-02 | 8318 | 4.37E-47 | 0.74 | 4.81E-04 | 1.10E-03 | 326224 | 6.90E-01 |  |
| rs1811471 | 1 | 159642599 | A | G | 0.28 | 2.89E-01 | 2.03E-02 | 8318 | 1.60E-45 | 0.26 | -4.70E-04 | 1.10E-03 | 326224 | 7.00E-01 |  |
| rs1900394 | 1 | 159632498 | T | G | 0.64 | -2.14E-01 | 1.89E-02 | 8318 | 1.20E-29 | 0.65 | 1.61E-03 | 1.01E-03 | 326224 | 1.00E-01 |  |
| rs2211320 | 1 | 159693605 | A | G | 0.33 | -2.34E-01 | 1.88E-02 | 8318 | 4.97E-35 | 0.33 | 5.34E-04 | 1.02E-03 | 326224 | 6.20E-01 |  |
| rs2592892 | 1 | 159645586 | C | G | 0.26 | -1.60E-01 | 2.15E-02 | 8318 | 1.02E-13 | 0.26 | 9.55E-04 | 1.11E-03 | 326224 | 4.00E-01 |  |
| rs2592893 | 1 | 159644292 | T | C | 0.74 | 1.61E-01 | 2.15E-02 | 8318 | 8.38E-14 | 0.74 | -9.74E-04 | 1.11E-03 | 326224 | 3.90E-01 |  |
| rs2794498 | 1 | 159636116 | T | G | 0.27 | 2.26E-01 | 2.04E-02 | 8318 | 2.29E-28 | 0.25 | -1.62E-03 | 1.11E-03 | 326224 | 1.50E-01 |  |
| rs2794500 | 1 | 159635021 | T | C | 0.36 | 2.15E-01 | 1.89E-02 | 8318 | 1.25E-29 | 0.35 | -1.66E-03 | 1.01E-03 | 326224 | 9.30E-02 |  |
| rs2808629 | 1 | 159676796 | A | G | 0.34 | -2.36E-01 | 1.88E-02 | 8318 | 8.22E-36 | 0.33 | 4.44E-04 | 1.02E-03 | 326224 | 6.90E-01 |  |
| rs3093059 | 1 | 159685136 | A | G | 0.95 | -3.18E-01 | 4.15E-02 | 8318 | 2.26E-14 | 0.94 | 1.52E-03 | 2.08E-03 | 326224 | 4.70E-01 |  |
| rs3116636 | 1 | 159686483 | A | G | 0.32 | 3.33E-01 | 1.89E-02 | 8318 | 9.63E-68 | 0.31 | -2.88E-04 | 1.04E-03 | 326224 | 7.90E-01 |  |
| rs3116651 | 1 | 159698485 | T | C | 0.32 | 3.31E-01 | 1.89E-02 | 8318 | 2.54E-67 | 0.31 | -2.28E-04 | 1.04E-03 | 326224 | 8.40E-01 |  |
| rs3116653 | 1 | 159696910 | C | G | 0.32 | 3.33E-01 | 1.89E-02 | 8318 | 3.41E-68 | 0.31 | -2.37E-04 | 1.04E-03 | 326224 | 8.30E-01 |  |
| rs3116656 | 1 | 159692372 | A | G | 0.68 | -3.31E-01 | 1.89E-02 | 8318 | 2.56E-67 | 0.69 | 2.99E-04 | 1.04E-03 | 326224 | 7.90E-01 |  |
| rs3122012 | 1 | 159689323 | T | C | 0.68 | -3.30E-01 | 1.89E-02 | 8318 | 3.73E-67 | 0.69 | 3.03E-04 | 1.04E-03 | 326224 | 7.80E-01 |  |
| rs35198997 | 1 | 159710368 | T | C | 0.74 | -3.18E-01 | 2.06E-02 | 8318 | 4.85E-53 | 0.74 | 4.45E-05 | 1.11E-03 | 326224 | 9.90E-01 |  |
| rs4131568 | 1 | 159722056 | T | C | 0.36 | 2.54E-01 | 1.86E-02 | 8318 | 3.22E-42 | 0.34 | -4.38E-04 | 1.01E-03 | 326224 | 6.90E-01 |  |
| rs4255379 | 1 | 159718312 | A | G | 0.61 | 2.17E-01 | 1.84E-02 | 8318 | 1.00E-31 | 0.61 | -7.98E-05 | 9.83E-04 | 326224 | 9.50E-01 |  |
| rs4261114 | 1 | 159700989 | A | C | 0.68 | -3.31E-01 | 1.89E-02 | 8318 | 2.73E-67 | 0.69 | 2.48E-04 | 1.04E-03 | 326224 | 8.20E-01 |  |
| rs4420078 | 1 | 159720716 | A | G | 0.39 | -2.16E-01 | 1.84E-02 | 8318 | 1.94E-31 | 0.39 | 8.88E-05 | 9.83E-04 | 326224 | 9.50E-01 |  |
| rs4512645 | 1 | 159729047 | A | G | 0.36 | 2.52E-01 | 1.86E-02 | 8318 | 2.31E-41 | 0.34 | -5.84E-04 | 1.02E-03 | 326224 | 5.80E-01 |  |
| rs7551731 | 1 | 159694779 | T | C | 0.67 | 2.33E-01 | 1.88E-02 | 8318 | 8.61E-35 | 0.67 | -5.09E-04 | 1.02E-03 | 326224 | 6.40E-01 |  |
| rs77289344 | 1 | 159691075 | T | G | 0.95 | -3.23E-01 | 4.17E-02 | 8318 | 1.11E-14 | 0.95 | 1.40E-03 | 2.10E-03 | 326224 | 5.10E-01 |  |
| rs876537 | 1 | 159674933 | T | C | 0.39 | -2.18E-01 | 1.82E-02 | 8318 | 6.62E-33 | 0.39 | -4.90E-04 | 9.83E-04 | 326224 | 6.00E-01 |  |
| rs895581 | 1 | 159637987 | A | G | 0.73 | -2.25E-01 | 2.04E-02 | 8318 | 3.91E-28 | 0.75 | 1.72E-03 | 1.11E-03 | 326224 | 1.30E-01 |  |

Instrumental cis SNPs (50K) select for CRP to Sleep classical Mendelian randomization analysis. CHR: chromosome; POS: hg19 genomic position; A1: effective allele; A2: the other allele; A1_exp_frq: A1 frequency in the exposure dataset; Beta_exp, effect of A1 for the exposure; SE_exp; standard error for Beta_exp; N_exp: sample size for exposure; P_exp, association p value for exposure; A1_out_frq: A1 frequency in the outcome dataset; Beta_out: effect size of A1 on outcome; SE_out: standard error of Beta_out; N_out: sample size for outcome; P_out: association p value for outcome; F-stats: F-statistics.

# **Table S14. Harmonized instrumental cisSNPs (p<5x10^-8^) for CRP in iPsych to Long Sleep MR.**

| SNP | CHR | POS | A1 | A2 | A1_exp_frq | Beta_exp | SE_exp | N_exp | P_exp | A1_out_frq | Beta_out | SE_out | N_out | P_out | Fstats |
| --- | --- | --- | --- | --- | --- | --- | --- | --- | --- | --- | --- | --- | --- | --- | --- |
| rs11265257 | 1 | 159668984 | T | C | 0.39 | -2.16E-01 | 1.82E-02 | 8318 | 3.62E-32 | 0.39 | 1.30E-03 | 7.46E-04 | 326224 | 7.80E-02 | 141.37 |
| rs11265264 | 1 | 159712842 | A | G | 0.36 | 2.70E-01 | 1.85E-02 | 8318 | 1.35E-47 | 0.36 | -2.46E-04 | 7.61E-04 | 326224 | 7.30E-01 | 213.00 |
| rs1130864 | 1 | 159683091 | A | G | 0.32 | 3.33E-01 | 1.89E-02 | 8318 | 9.41E-68 | 0.31 | -2.86E-04 | 7.87E-04 | 326224 | 6.90E-01 | 309.50 |
| rs11588887 | 1 | 159717162 | A | G | 0.16 | -1.77E-01 | 2.50E-02 | 8318 | 1.78E-12 | 0.16 | 1.16E-03 | 1.00E-03 | 326224 | 2.40E-01 | 50.01 |
| rs12093699 | 1 | 159647988 | A | G | 0.32 | 3.03E-01 | 1.88E-02 | 8318 | 1.69E-57 | 0.31 | -5.40E-04 | 7.87E-04 | 326224 | 4.80E-01 | 259.93 |
| rs12094103 | 1 | 159723619 | A | G | 0.36 | 2.46E-01 | 1.85E-02 | 8318 | 7.20E-40 | 0.35 | -1.55E-04 | 7.62E-04 | 326224 | 8.20E-01 | 176.24 |
| rs12726900 | 1 | 159732523 | A | G | 0.37 | 2.48E-01 | 2.00E-02 | 8318 | 5.23E-35 | 0.35 | -2.32E-04 | 7.98E-04 | 326224 | 7.50E-01 | 153.64 |
| rs12727021 | 1 | 159702487 | A | G | 0.32 | 3.31E-01 | 1.89E-02 | 8318 | 2.93E-67 | 0.31 | -2.14E-04 | 7.87E-04 | 326224 | 7.60E-01 | 306.16 |
| rs12739022 | 1 | 159659431 | T | C | 0.68 | -3.28E-01 | 1.90E-02 | 8318 | 7.59E-66 | 0.69 | 3.14E-04 | 7.89E-04 | 326224 | 6.70E-01 | 298.56 |
| rs12754745 | 1 | 159670483 | A | G | 0.32 | 3.30E-01 | 1.90E-02 | 8318 | 1.19E-66 | 0.31 | -2.88E-04 | 7.89E-04 | 326224 | 6.90E-01 | 302.03 |
| rs12754915 | 1 | 159660869 | T | C | 0.68 | -3.28E-01 | 1.90E-02 | 8318 | 7.18E-66 | 0.69 | 2.77E-04 | 7.89E-04 | 326224 | 7.00E-01 | 298.56 |
| rs12759988 | 1 | 159708751 | A | G | 0.35 | 2.72E-01 | 1.87E-02 | 8318 | 3.36E-47 | 0.33 | -7.79E-05 | 7.71E-04 | 326224 | 9.00E-01 | 210.79 |
| rs12760041 | 1 | 159714035 | T | C | 0.35 | 2.70E-01 | 1.87E-02 | 8318 | 1.03E-46 | 0.33 | -1.45E-04 | 7.71E-04 | 326224 | 8.30E-01 | 208.16 |
| rs137918962 | 1 | 159652311 | A | G | 0.05 | 2.92E-01 | 4.37E-02 | 8318 | 2.60E-11 | 0.05 | 2.82E-04 | 1.69E-03 | 326224 | 8.80E-01 | 44.56 |
| rs1470515 | 1 | 159653599 | T | C | 0.39 | -2.13E-01 | 1.83E-02 | 8318 | 4.19E-31 | 0.39 | 1.30E-03 | 7.47E-04 | 326224 | 7.70E-02 | 135.60 |
| rs16842484 | 1 | 159646924 | T | C | 0.72 | -2.94E-01 | 2.02E-02 | 8318 | 4.37E-47 | 0.74 | 1.92E-04 | 8.34E-04 | 326224 | 8.00E-01 | 211.26 |
| rs1811471 | 1 | 159642599 | A | G | 0.28 | 2.89E-01 | 2.03E-02 | 8318 | 1.60E-45 | 0.26 | -9.93E-05 | 8.36E-04 | 326224 | 8.90E-01 | 202.82 |
| rs1900394 | 1 | 159632498 | T | G | 0.64 | -2.14E-01 | 1.89E-02 | 8318 | 1.20E-29 | 0.65 | 9.71E-04 | 7.61E-04 | 326224 | 2.00E-01 | 128.68 |
| rs2211320 | 1 | 159693605 | A | G | 0.33 | -2.34E-01 | 1.88E-02 | 8318 | 4.97E-35 | 0.33 | 1.30E-03 | 7.71E-04 | 326224 | 8.50E-02 | 154.53 |
| rs2592892 | 1 | 159645586 | C | G | 0.26 | -1.60E-01 | 2.15E-02 | 8318 | 1.02E-13 | 0.26 | -3.03E-04 | 8.39E-04 | 326224 | 7.30E-01 | 55.59 |
| rs2592893 | 1 | 159644292 | T | C | 0.74 | 1.61E-01 | 2.15E-02 | 8318 | 8.38E-14 | 0.74 | 3.09E-04 | 8.39E-04 | 326224 | 7.30E-01 | 55.87 |
| rs2794498 | 1 | 159636116 | T | G | 0.27 | 2.26E-01 | 2.04E-02 | 8318 | 2.29E-28 | 0.25 | -5.23E-04 | 8.39E-04 | 326224 | 5.30E-01 | 122.73 |
| rs2794500 | 1 | 159635021 | T | C | 0.36 | 2.15E-01 | 1.89E-02 | 8318 | 1.25E-29 | 0.35 | -1.09E-03 | 7.61E-04 | 326224 | 1.50E-01 | 128.80 |
| rs2808629 | 1 | 159676796 | A | G | 0.34 | -2.36E-01 | 1.88E-02 | 8318 | 8.22E-36 | 0.33 | 1.34E-03 | 7.70E-04 | 326224 | 7.70E-02 | 157.58 |
| rs3093059 | 1 | 159685136 | A | G | 0.95 | -3.18E-01 | 4.15E-02 | 8318 | 2.26E-14 | 0.94 | 1.43E-03 | 1.57E-03 | 326224 | 3.60E-01 | 58.61 |
| rs3116636 | 1 | 159686483 | A | G | 0.32 | 3.33E-01 | 1.89E-02 | 8318 | 9.63E-68 | 0.31 | -2.59E-04 | 7.88E-04 | 326224 | 7.20E-01 | 309.50 |
| rs3116651 | 1 | 159698485 | T | C | 0.32 | 3.31E-01 | 1.89E-02 | 8318 | 2.54E-67 | 0.31 | -1.98E-04 | 7.87E-04 | 326224 | 7.80E-01 | 306.16 |
| rs3116653 | 1 | 159696910 | C | G | 0.32 | 3.33E-01 | 1.89E-02 | 8318 | 3.41E-68 | 0.31 | -2.00E-04 | 7.87E-04 | 326224 | 7.70E-01 | 309.87 |
| rs3116656 | 1 | 159692372 | A | G | 0.68 | -3.31E-01 | 1.89E-02 | 8318 | 2.56E-67 | 0.69 | 1.70E-04 | 7.86E-04 | 326224 | 8.00E-01 | 306.16 |
| rs3122012 | 1 | 159689323 | T | C | 0.68 | -3.30E-01 | 1.89E-02 | 8318 | 3.73E-67 | 0.69 | 2.01E-04 | 7.87E-04 | 326224 | 7.70E-01 | 305.42 |
| rs35198997 | 1 | 159710368 | T | C | 0.74 | -3.18E-01 | 2.06E-02 | 8318 | 4.85E-53 | 0.74 | 2.86E-04 | 8.40E-04 | 326224 | 7.10E-01 | 238.90 |
| rs4131568 | 1 | 159722056 | T | C | 0.36 | 2.54E-01 | 1.86E-02 | 8318 | 3.22E-42 | 0.34 | -1.22E-04 | 7.68E-04 | 326224 | 8.50E-01 | 187.07 |
| rs4255379 | 1 | 159718312 | A | G | 0.61 | 2.17E-01 | 1.84E-02 | 8318 | 1.00E-31 | 0.61 | -1.99E-04 | 7.45E-04 | 326224 | 7.80E-01 | 138.83 |
| rs4261114 | 1 | 159700989 | A | C | 0.68 | -3.31E-01 | 1.89E-02 | 8318 | 2.73E-67 | 0.69 | 2.17E-04 | 7.88E-04 | 326224 | 7.60E-01 | 306.34 |
| rs4420078 | 1 | 159720716 | A | G | 0.39 | -2.16E-01 | 1.84E-02 | 8318 | 1.94E-31 | 0.39 | 1.96E-04 | 7.45E-04 | 326224 | 7.90E-01 | 137.30 |
| rs4512645 | 1 | 159729047 | A | G | 0.36 | 2.52E-01 | 1.86E-02 | 8318 | 2.31E-41 | 0.34 | -1.95E-04 | 7.69E-04 | 326224 | 7.80E-01 | 183.41 |
| rs7551731 | 1 | 159694779 | T | C | 0.67 | 2.33E-01 | 1.88E-02 | 8318 | 8.61E-35 | 0.67 | -1.31E-03 | 7.71E-04 | 326224 | 8.50E-02 | 153.07 |
| rs77289344 | 1 | 159691075 | T | G | 0.95 | -3.23E-01 | 4.17E-02 | 8318 | 1.11E-14 | 0.95 | 1.08E-03 | 1.59E-03 | 326224 | 4.90E-01 | 59.92 |
| rs876537 | 1 | 159674933 | T | C | 0.39 | -2.18E-01 | 1.82E-02 | 8318 | 6.62E-33 | 0.39 | 1.27E-03 | 7.45E-04 | 326224 | 8.40E-02 | 143.87 |
| rs895581 | 1 | 159637987 | A | G | 0.73 | -2.25E-01 | 2.04E-02 | 8318 | 3.91E-28 | 0.75 | 5.99E-04 | 8.38E-04 | 326224 | 4.70E-01 | 121.76 |

Instrumental cis SNPs (50K) select for CRP to Sleep classical Mendelian randomization analysis. CHR: chromosome; POS: hg19 genomic position; A1: effective allele; A2: the other allele; A1_exp_frq: A1 frequency in the exposure dataset; Beta_exp, effect of A1 for the exposure; SE_exp; standard error for Beta_exp; N_exp: sample size for exposure; P_exp, association p value for exposure; A1_out_frq: A1 frequency in the outcome dataset; Beta_out: effect size of A1 on outcome; SE_out: standard error of Beta_out; N_out: sample size for outcome; P_out: association p value for outcome; F-stats: F-statistics.

# **Table S15. Harmonized instrumental cisSNPs (p<5x10^-8^) for CRP in iPsych to Sleep Duration MR.**

| SNP | CHR | POS | A1 | A2 | A1_exp_frq | Beta_exp | SE_exp | N_exp | P_exp | A1_out_frq | Beta_out | SE_out | N_out | P_out | Fstats |
| --- | --- | --- | --- | --- | --- | --- | --- | --- | --- | --- | --- | --- | --- | --- | --- |
| rs11265257 | 1 | 159668984 | T | C | 0.39 | -2.16E-01 | 1.82E-02 | 8318 | 3.62E-32 | 0.39 | 2.94E-03 | 2.32E-03 | 326224 | 2.10E-01 | 141.37 |
| rs11265264 | 1 | 159712842 | A | G | 0.36 | 2.70E-01 | 1.85E-02 | 8318 | 1.35E-47 | 0.36 | -5.19E-04 | 2.37E-03 | 326224 | 8.40E-01 | 213.00 |
| rs1130864 | 1 | 159683091 | A | G | 0.32 | 3.33E-01 | 1.89E-02 | 8318 | 9.41E-68 | 0.31 | -4.41E-05 | 2.45E-03 | 326224 | 9.80E-01 | 309.50 |
| rs11588887 | 1 | 159717162 | A | G | 0.16 | -1.77E-01 | 2.50E-02 | 8318 | 1.78E-12 | 0.16 | 1.98E-03 | 3.12E-03 | 326224 | 5.20E-01 | 50.01 |
| rs12093699 | 1 | 159647988 | A | G | 0.32 | 3.03E-01 | 1.88E-02 | 8318 | 1.69E-57 | 0.31 | -3.46E-05 | 2.45E-03 | 326224 | 9.70E-01 | 259.93 |
| rs12094103 | 1 | 159723619 | A | G | 0.36 | 2.46E-01 | 1.85E-02 | 8318 | 7.20E-40 | 0.35 | -4.32E-04 | 2.38E-03 | 326224 | 8.60E-01 | 176.24 |
| rs12726900 | 1 | 159732523 | A | G | 0.37 | 2.48E-01 | 2.00E-02 | 8318 | 5.23E-35 | 0.35 | 8.25E-05 | 2.49E-03 | 326224 | 9.70E-01 | 153.64 |
| rs12727021 | 1 | 159702487 | A | G | 0.32 | 3.31E-01 | 1.89E-02 | 8318 | 2.93E-67 | 0.31 | 6.54E-05 | 2.45E-03 | 326224 | 9.80E-01 | 306.16 |
| rs12739022 | 1 | 159659431 | T | C | 0.68 | -3.28E-01 | 1.90E-02 | 8318 | 7.59E-66 | 0.69 | -1.92E-04 | 2.46E-03 | 326224 | 9.40E-01 | 298.56 |
| rs12754745 | 1 | 159670483 | A | G | 0.32 | 3.30E-01 | 1.90E-02 | 8318 | 1.19E-66 | 0.31 | 2.28E-04 | 2.46E-03 | 326224 | 9.30E-01 | 302.03 |
| rs12754915 | 1 | 159660869 | T | C | 0.68 | -3.28E-01 | 1.90E-02 | 8318 | 7.18E-66 | 0.69 | -2.31E-04 | 2.46E-03 | 326224 | 9.30E-01 | 298.56 |
| rs12759988 | 1 | 159708751 | A | G | 0.35 | 2.72E-01 | 1.87E-02 | 8318 | 3.36E-47 | 0.33 | -4.59E-04 | 2.40E-03 | 326224 | 8.60E-01 | 210.79 |
| rs12760041 | 1 | 159714035 | T | C | 0.35 | 2.70E-01 | 1.87E-02 | 8318 | 1.03E-46 | 0.33 | -6.98E-04 | 2.40E-03 | 326224 | 7.80E-01 | 208.16 |
| rs137918962 | 1 | 159652311 | A | G | 0.05 | 2.92E-01 | 4.37E-02 | 8318 | 2.60E-11 | 0.05 | 7.07E-03 | 5.26E-03 | 326224 | 2.00E-01 | 44.56 |
| rs1470515 | 1 | 159653599 | T | C | 0.39 | -2.13E-01 | 1.83E-02 | 8318 | 4.19E-31 | 0.39 | 2.66E-03 | 2.33E-03 | 326224 | 2.60E-01 | 135.60 |
| rs16842484 | 1 | 159646924 | T | C | 0.72 | -2.94E-01 | 2.02E-02 | 8318 | 4.37E-47 | 0.74 | -2.93E-04 | 2.60E-03 | 326224 | 9.50E-01 | 211.26 |
| rs1811471 | 1 | 159642599 | A | G | 0.28 | 2.89E-01 | 2.03E-02 | 8318 | 1.60E-45 | 0.26 | 8.04E-04 | 2.61E-03 | 326224 | 7.90E-01 | 202.82 |
| rs1900394 | 1 | 159632498 | T | G | 0.64 | -2.14E-01 | 1.89E-02 | 8318 | 1.20E-29 | 0.65 | 1.11E-03 | 2.37E-03 | 326224 | 6.50E-01 | 128.68 |
| rs2211320 | 1 | 159693605 | A | G | 0.33 | -2.34E-01 | 1.88E-02 | 8318 | 4.97E-35 | 0.33 | 3.66E-04 | 2.40E-03 | 326224 | 8.50E-01 | 154.53 |
| rs2592892 | 1 | 159645586 | C | G | 0.26 | -1.60E-01 | 2.15E-02 | 8318 | 1.02E-13 | 0.26 | -2.62E-03 | 2.61E-03 | 326224 | 3.40E-01 | 55.59 |
| rs2592893 | 1 | 159644292 | T | C | 0.74 | 1.61E-01 | 2.15E-02 | 8318 | 8.38E-14 | 0.74 | 2.79E-03 | 2.61E-03 | 326224 | 3.10E-01 | 55.87 |
| rs2794498 | 1 | 159636116 | T | G | 0.27 | 2.26E-01 | 2.04E-02 | 8318 | 2.29E-28 | 0.25 | -9.67E-04 | 2.62E-03 | 326224 | 7.10E-01 | 122.73 |
| rs2794500 | 1 | 159635021 | T | C | 0.36 | 2.15E-01 | 1.89E-02 | 8318 | 1.25E-29 | 0.35 | -1.19E-03 | 2.37E-03 | 326224 | 6.30E-01 | 128.80 |
| rs2808629 | 1 | 159676796 | A | G | 0.34 | -2.36E-01 | 1.88E-02 | 8318 | 8.22E-36 | 0.33 | 7.09E-04 | 2.40E-03 | 326224 | 7.40E-01 | 157.58 |
| rs3093059 | 1 | 159685136 | A | G | 0.95 | -3.18E-01 | 4.15E-02 | 8318 | 2.26E-14 | 0.94 | -2.23E-03 | 4.91E-03 | 326224 | 7.00E-01 | 58.61 |
| rs3116636 | 1 | 159686483 | A | G | 0.32 | 3.33E-01 | 1.89E-02 | 8318 | 9.63E-68 | 0.31 | -6.75E-05 | 2.46E-03 | 326224 | 9.80E-01 | 309.50 |
| rs3116651 | 1 | 159698485 | T | C | 0.32 | 3.31E-01 | 1.89E-02 | 8318 | 2.54E-67 | 0.31 | 5.63E-05 | 2.45E-03 | 326224 | 9.80E-01 | 306.16 |
| rs3116653 | 1 | 159696910 | C | G | 0.32 | 3.33E-01 | 1.89E-02 | 8318 | 3.41E-68 | 0.31 | 6.86E-05 | 2.45E-03 | 326224 | 9.80E-01 | 309.87 |
| rs3116656 | 1 | 159692372 | A | G | 0.68 | -3.31E-01 | 1.89E-02 | 8318 | 2.56E-67 | 0.69 | -2.28E-04 | 2.45E-03 | 326224 | 9.30E-01 | 306.16 |
| rs3122012 | 1 | 159689323 | T | C | 0.68 | -3.30E-01 | 1.89E-02 | 8318 | 3.73E-67 | 0.69 | -1.40E-04 | 2.45E-03 | 326224 | 9.60E-01 | 305.42 |
| rs35198997 | 1 | 159710368 | T | C | 0.74 | -3.18E-01 | 2.06E-02 | 8318 | 4.85E-53 | 0.74 | 1.31E-03 | 2.62E-03 | 326224 | 5.90E-01 | 238.90 |
| rs4131568 | 1 | 159722056 | T | C | 0.36 | 2.54E-01 | 1.86E-02 | 8318 | 3.22E-42 | 0.34 | -7.59E-04 | 2.39E-03 | 326224 | 7.50E-01 | 187.07 |
| rs4255379 | 1 | 159718312 | A | G | 0.61 | 2.17E-01 | 1.84E-02 | 8318 | 1.00E-31 | 0.61 | -1.13E-04 | 2.32E-03 | 326224 | 9.60E-01 | 138.83 |
| rs4261114 | 1 | 159700989 | A | C | 0.68 | -3.31E-01 | 1.89E-02 | 8318 | 2.73E-67 | 0.69 | -8.46E-05 | 2.46E-03 | 326224 | 9.70E-01 | 306.34 |
| rs4420078 | 1 | 159720716 | A | G | 0.39 | -2.16E-01 | 1.84E-02 | 8318 | 1.94E-31 | 0.39 | 1.04E-04 | 2.32E-03 | 326224 | 9.60E-01 | 137.30 |
| rs4512645 | 1 | 159729047 | A | G | 0.36 | 2.52E-01 | 1.86E-02 | 8318 | 2.31E-41 | 0.34 | -5.75E-04 | 2.40E-03 | 326224 | 8.10E-01 | 183.41 |
| rs7551731 | 1 | 159694779 | T | C | 0.67 | 2.33E-01 | 1.88E-02 | 8318 | 8.61E-35 | 0.67 | -3.81E-04 | 2.40E-03 | 326224 | 8.50E-01 | 153.07 |
| rs77289344 | 1 | 159691075 | T | G | 0.95 | -3.23E-01 | 4.17E-02 | 8318 | 1.11E-14 | 0.95 | -2.98E-03 | 4.97E-03 | 326224 | 5.90E-01 | 59.92 |
| rs876537 | 1 | 159674933 | T | C | 0.39 | -2.18E-01 | 1.82E-02 | 8318 | 6.62E-33 | 0.39 | 2.95E-03 | 2.32E-03 | 326224 | 2.10E-01 | 143.87 |
| rs895581 | 1 | 159637987 | A | G | 0.73 | -2.25E-01 | 2.04E-02 | 8318 | 3.91E-28 | 0.75 | 9.45E-04 | 2.62E-03 | 326224 | 7.20E-01 | 121.76 |

Instrumental cis SNPs (50K) select for CRP to Sleep classical Mendelian randomization analysis. CHR: chromosome; POS: hg19 genomic position; A1: effective allele; A2: the other allele; A1_exp_frq: A1 frequency in the exposure dataset; Beta_exp, effect of A1 for the exposure; SE_exp; standard error for Beta_exp; N_exp: sample size for exposure; P_exp, association p value for exposure; A1_out_frq: A1 frequency in the outcome dataset; Beta_out: effect size of A1 on outcome; SE_out: standard error of Beta_out; N_out: sample size for outcome; P_out: association p value for outcome; F-stats: F-statistics.

# **Table S16. Harmonized instrumental cisSNPs (p<5x10^-8^) for CRP to Insomnia MR.**

| SNP | CHR | POS | A1 | A2 | A1_exp_frq | Beta_exp | SE_exp | N_exp | P_exp | A1_out_frq | Beta_out | SE_out | N_out | P_out | Fstats |
| --- | --- | --- | --- | --- | --- | --- | --- | --- | --- | --- | --- | --- | --- | --- | --- |
| rs10908741 | 1 | 159726123 | T | G | 0.25 | -5.02E-02 | 4.16E-03 | 204402 | 1.64E-33 | 0.24 | 0.00E+00 | 5.98E-03 | 382581 | 9.85E-01 | 145.68 |
| rs11265257 | 1 | 159668984 | T | C | 0.39 | -1.69E-01 | 3.64E-03 | 204402 | 1.00E-300 | 0.39 | 2.00E-03 | 5.24E-03 | 384026 | 7.46E-01 | 2150.79 |
| rs11265260 | 1 | 159700039 | G | A | 0.06 | 2.21E-01 | 7.29E-03 | 204402 | 2.12E-202 | 0.06 | 9.95E-03 | 1.09E-02 | 386533 | 3.66E-01 | 921.76 |
| rs11265263 | 1 | 159710517 | A | C | 0.08 | -2.69E-01 | 8.04E-03 | 204402 | 7.52E-245 | 0.07 | -7.43E-03 | 9.93E-03 | 386533 | 4.52E-01 | 1117.07 |
| rs11588887 | 1 | 159717162 | A | G | 0.14 | -2.04E-01 | 5.81E-03 | 204402 | 5.79E-271 | 0.16 | -1.30E-03 | 7.05E-03 | 381493 | 8.52E-01 | 1237.07 |
| rs11811420 | 1 | 159719872 | C | G | 0.36 | 9.98E-02 | 3.68E-03 | 204402 | 6.49E-162 | 0.35 | -3.21E-03 | 5.36E-03 | 383360 | 5.47E-01 | 735.50 |
| rs12029953 | 1 | 159727739 | A | C | 0.25 | -5.41E-02 | 4.44E-03 | 204402 | 5.18E-34 | 0.24 | -2.00E-04 | 5.98E-03 | 382493 | 9.73E-01 | 147.98 |
| rs12049404 | 1 | 159713844 | T | C | 0.16 | -1.75E-01 | 4.97E-03 | 204402 | 2.55E-270 | 0.16 | -1.30E-03 | 7.04E-03 | 381757 | 8.59E-01 | 1234.47 |
| rs1205 | 1 | 159682233 | T | C | 0.33 | -1.83E-01 | 3.73E-03 | 204402 | 1.00E-300 | 0.33 | 3.00E-03 | 5.40E-03 | 386533 | 5.58E-01 | 2400.50 |
| rs12081252 | 1 | 159706513 | C | T | 0.06 | 2.23E-01 | 7.57E-03 | 204402 | 5.40E-190 | 0.06 | 5.98E-03 | 1.10E-02 | 385762 | 5.61E-01 | 864.74 |
| rs12093699 | 1 | 159647988 | A | G | 0.31 | 1.28E-01 | 3.84E-03 | 204402 | 5.13E-243 | 0.31 | -7.02E-03 | 5.53E-03 | 386533 | 2.07E-01 | 1108.82 |
| rs12094103 | 1 | 159723619 | A | G | 0.36 | 9.96E-02 | 3.71E-03 | 204402 | 5.19E-159 | 0.35 | -2.50E-03 | 5.36E-03 | 383822 | 6.37E-01 | 722.30 |
| rs12567054 | 1 | 159644968 | G | T | 0.07 | 1.54E-01 | 8.26E-03 | 204402 | 1.08E-77 | 0.06 | 1.39E-02 | 1.06E-02 | 381247 | 1.99E-01 | 348.39 |
| rs12727021 | 1 | 159702487 | A | G | 0.31 | 1.40E-01 | 3.80E-03 | 204402 | 1.09E-294 | 0.31 | -4.11E-03 | 5.53E-03 | 386233 | 4.60E-01 | 1346.52 |
| rs12739022 | 1 | 159659431 | C | T | 0.32 | 1.41E-01 | 3.96E-03 | 204402 | 3.72E-278 | 0.31 | -4.91E-03 | 5.57E-03 | 380707 | 3.82E-01 | 1270.61 |
| rs12754915 | 1 | 159660869 | C | T | 0.31 | 1.44E-01 | 3.87E-03 | 204402 | 1.00E-300 | 0.31 | -5.11E-03 | 5.57E-03 | 380709 | 3.60E-01 | 1377.50 |
| rs1341665 | 1 | 159691559 | A | G | 0.33 | -1.81E-01 | 3.85E-03 | 204402 | 1.00E-300 | 0.34 | 2.00E-03 | 5.37E-03 | 386533 | 7.14E-01 | 2221.42 |
| rs1470515 | 1 | 159653599 | T | C | 0.39 | -1.68E-01 | 3.72E-03 | 204402 | 1.00E-300 | 0.39 | 2.00E-03 | 5.25E-03 | 381417 | 6.93E-01 | 2033.30 |
| rs1572970 | 1 | 159673585 | A | G | 0.70 | -4.39E-02 | 3.97E-03 | 204402 | 2.06E-28 | 0.70 | -3.99E-03 | 5.60E-03 | 383211 | 4.40E-01 | 122.39 |
| rs16842484 | 1 | 159646924 | C | T | 0.26 | 1.20E-01 | 4.18E-03 | 204402 | 6.84E-181 | 0.25 | -9.14E-03 | 5.91E-03 | 378816 | 1.21E-01 | 822.83 |
| rs16842599 | 1 | 159697475 | C | T | 0.06 | 2.23E-01 | 7.54E-03 | 204402 | 2.82E-192 | 0.06 | 6.98E-03 | 1.10E-02 | 386422 | 5.32E-01 | 875.17 |
| rs1971863 | 1 | 159638931 | C | T | 0.26 | 9.03E-02 | 4.15E-03 | 204402 | 7.94E-105 | 0.25 | -6.52E-03 | 5.96E-03 | 379800 | 2.75E-01 | 473.01 |
| rs2027469 | 1 | 159667190 | A | G | 0.18 | -8.41E-02 | 4.89E-03 | 204402 | 3.21E-66 | 0.19 | 0.00E+00 | 6.53E-03 | 383844 | 9.86E-01 | 295.73 |
| rs2794498 | 1 | 159636116 | T | G | 0.26 | 8.71E-02 | 4.05E-03 | 204402 | 8.34E-103 | 0.25 | -6.82E-03 | 5.90E-03 | 385571 | 2.46E-01 | 463.87 |
| rs2794500 | 1 | 159635021 | T | C | 0.36 | 7.03E-02 | 3.69E-03 | 204402 | 9.98E-81 | 0.35 | -4.81E-03 | 5.34E-03 | 386358 | 3.68E-01 | 362.33 |
| rs2794520 | 1 | 159678816 | T | C | 0.33 | -1.82E-01 | 3.71E-03 | 204402 | 1.00E-300 | 0.33 | 3.99E-03 | 5.40E-03 | 386533 | 4.86E-01 | 2408.87 |
| rs2808628 | 1 | 159676011 | A | G | 0.33 | -1.82E-01 | 3.86E-03 | 204402 | 1.00E-300 | 0.33 | 3.99E-03 | 5.40E-03 | 385861 | 4.91E-01 | 2238.23 |
| rs3093075 | 1 | 159679913 | T | G | 0.06 | 2.26E-01 | 7.39E-03 | 204402 | 2.02E-204 | 0.06 | 8.96E-03 | 1.10E-02 | 386120 | 4.04E-01 | 931.16 |
| rs4131568 | 1 | 159722056 | T | C | 0.36 | 9.70E-02 | 3.82E-03 | 204402 | 4.89E-142 | 0.34 | -1.00E-03 | 5.39E-03 | 386533 | 8.48E-01 | 644.29 |
| rs4255379 | 1 | 159718312 | A | G | 0.63 | 6.39E-02 | 3.82E-03 | 204402 | 1.35E-62 | 0.61 | -3.99E-03 | 5.24E-03 | 383610 | 4.09E-01 | 279.05 |
| rs4512645 | 1 | 159729047 | A | G | 0.35 | 9.95E-02 | 3.93E-03 | 204402 | 8.22E-142 | 0.34 | -1.60E-03 | 5.40E-03 | 385080 | 7.68E-01 | 643.16 |
| rs7553007 | 1 | 159698549 | A | G | 0.32 | -1.82E-01 | 3.86E-03 | 204402 | 1.00E-300 | 0.33 | 2.00E-03 | 5.40E-03 | 386533 | 6.91E-01 | 2220.29 |
| rs876537 | 1 | 159674933 | T | C | 0.38 | -1.70E-01 | 3.67E-03 | 204402 | 1.00E-300 | 0.39 | 1.00E-03 | 5.22E-03 | 386533 | 7.79E-01 | 2137.45 |
| rs895582 | 1 | 159637919 | A | G | 0.26 | 8.99E-02 | 4.09E-03 | 204402 | 3.79E-107 | 0.25 | -7.23E-03 | 5.90E-03 | 385378 | 2.23E-01 | 483.62 |

Instrumental cis SNPs (50K) select for CRP to Sleep classical Mendelian randomization analysis. CHR: chromosome; POS: hg19 genomic position; A1: effective allele; A2: the other allele; A1_exp_frq: A1 frequency in the exposure dataset; Beta_exp, effect of A1 for the exposure; SE_exp; standard error for Beta_exp; N_exp: sample size for exposure; P_exp, association p value for exposure; A1_out_frq: A1 frequency in the outcome dataset; Beta_out: effect size of A1 on outcome; SE_out: standard error of Beta_out; N_out: sample size for outcome; P_out: association p value for outcome; F-stats: F-statistics.

# **Table S17. Harmonized instrumental cisSNPs (p<5x10^-8^) for CRP to Sleepiness MR.**

| SNP | CHR | POS | A1 | A2 | A1_exp_frq | Beta_exp | SE_exp | N_exp | P_exp | A1_out_frq | Beta_out | SE_out | N_out | P_out | Fstats |
| --- | --- | --- | --- | --- | --- | --- | --- | --- | --- | --- | --- | --- | --- | --- | --- |
| rs10908741 | 1 | 159726123 | T | G | 0.25 | -5.02E-02 | 4.16E-03 | 204402 | 1.64E-33 | 0.24 | 1.09E-03 | 1.12E-03 | 326224 | 3.30E-01 | 145.68 |
| rs10908742 | 1 | 159732811 | G | T | 0.24 | -5.62E-02 | 4.92E-03 | 204402 | 3.50E-30 | 0.24 | 1.15E-03 | 1.19E-03 | 326224 | 3.30E-01 | 130.47 |
| rs11265257 | 1 | 159668984 | T | C | 0.39 | -1.69E-01 | 3.64E-03 | 204402 | 1.00E-300 | 0.39 | -4.69E-04 | 9.85E-04 | 326224 | 6.20E-01 | 2150.79 |
| rs11265260 | 1 | 159700039 | G | A | 0.06 | 2.21E-01 | 7.29E-03 | 204402 | 2.12E-202 | 0.06 | -6.91E-04 | 2.06E-03 | 326224 | 7.50E-01 | 921.76 |
| rs11265263 | 1 | 159710517 | A | C | 0.08 | -2.69E-01 | 8.04E-03 | 204402 | 7.52E-245 | 0.07 | 3.57E-04 | 1.87E-03 | 326224 | 8.90E-01 | 1117.07 |
| rs11588887 | 1 | 159717162 | A | G | 0.14 | -2.04E-01 | 5.81E-03 | 204402 | 5.79E-271 | 0.16 | 4.16E-04 | 1.32E-03 | 326224 | 7.70E-01 | 1237.07 |
| rs11589667 | 1 | 159641029 | T | C | 0.33 | -4.53E-02 | 4.70E-03 | 204402 | 5.18E-22 | 0.34 | -1.02E-03 | 1.03E-03 | 326224 | 3.20E-01 | 93.13 |
| rs11811420 | 1 | 159719872 | C | G | 0.36 | 9.98E-02 | 3.68E-03 | 204402 | 6.49E-162 | 0.35 | -5.86E-04 | 1.01E-03 | 326224 | 5.80E-01 | 735.50 |
| rs12029953 | 1 | 159727739 | A | C | 0.25 | -5.41E-02 | 4.44E-03 | 204402 | 5.18E-34 | 0.24 | 1.09E-03 | 1.12E-03 | 326224 | 3.40E-01 | 147.98 |
| rs12049404 | 1 | 159713844 | T | C | 0.16 | -1.75E-01 | 4.97E-03 | 204402 | 2.55E-270 | 0.16 | 3.93E-04 | 1.32E-03 | 326224 | 7.90E-01 | 1234.47 |
| rs1205 | 1 | 159682233 | T | C | 0.33 | -1.83E-01 | 3.73E-03 | 204402 | 1.00E-300 | 0.33 | 3.88E-04 | 1.02E-03 | 326224 | 7.20E-01 | 2400.50 |
| rs12081252 | 1 | 159706513 | C | T | 0.06 | 2.23E-01 | 7.57E-03 | 204402 | 5.40E-190 | 0.06 | -1.17E-03 | 2.09E-03 | 326224 | 5.80E-01 | 864.74 |
| rs12093699 | 1 | 159647988 | A | G | 0.31 | 1.28E-01 | 3.84E-03 | 204402 | 5.13E-243 | 0.31 | -7.44E-04 | 1.04E-03 | 326224 | 4.90E-01 | 1108.82 |
| rs12094103 | 1 | 159723619 | A | G | 0.36 | 9.96E-02 | 3.71E-03 | 204402 | 5.19E-159 | 0.35 | -6.68E-04 | 1.01E-03 | 326224 | 5.30E-01 | 722.30 |
| rs12567054 | 1 | 159644968 | G | T | 0.07 | 1.54E-01 | 8.26E-03 | 204402 | 1.08E-77 | 0.06 | -1.20E-03 | 1.97E-03 | 326224 | 5.30E-01 | 348.39 |
| rs12727021 | 1 | 159702487 | A | G | 0.31 | 1.40E-01 | 3.80E-03 | 204402 | 1.09E-294 | 0.31 | -2.74E-04 | 1.04E-03 | 326224 | 8.00E-01 | 1346.52 |
| rs12739022 | 1 | 159659431 | C | T | 0.32 | 1.41E-01 | 3.96E-03 | 204402 | 3.72E-278 | 0.31 | -4.17E-04 | 1.04E-03 | 326224 | 7.00E-01 | 1270.61 |
| rs12754915 | 1 | 159660869 | C | T | 0.31 | 1.44E-01 | 3.87E-03 | 204402 | 1.00E-300 | 0.31 | -3.95E-04 | 1.04E-03 | 326224 | 7.20E-01 | 1377.50 |
| rs1341665 | 1 | 159691559 | A | G | 0.33 | -1.81E-01 | 3.85E-03 | 204402 | 1.00E-300 | 0.34 | 4.07E-04 | 1.01E-03 | 326224 | 7.10E-01 | 2221.42 |
| rs1470515 | 1 | 159653599 | T | C | 0.39 | -1.68E-01 | 3.72E-03 | 204402 | 1.00E-300 | 0.39 | -2.29E-04 | 9.87E-04 | 326224 | 8.00E-01 | 2033.30 |
| rs1572970 | 1 | 159673585 | A | G | 0.70 | -4.39E-02 | 3.97E-03 | 204402 | 2.06E-28 | 0.70 | -1.06E-03 | 1.05E-03 | 326224 | 3.10E-01 | 122.39 |
| rs16842484 | 1 | 159646924 | C | T | 0.26 | 1.20E-01 | 4.18E-03 | 204402 | 6.84E-181 | 0.26 | -4.81E-04 | 1.10E-03 | 326224 | 6.90E-01 | 822.83 |
| rs16842599 | 1 | 159697475 | C | T | 0.06 | 2.23E-01 | 7.54E-03 | 204402 | 2.82E-192 | 0.06 | -1.43E-03 | 2.08E-03 | 326224 | 5.00E-01 | 875.17 |
| rs1811472 | 1 | 159642349 | C | G | 0.41 | -1.21E-01 | 3.89E-03 | 204402 | 2.11E-213 | 0.40 | 5.63E-04 | 9.89E-04 | 326224 | 5.70E-01 | 972.20 |
| rs1971863 | 1 | 159638931 | C | T | 0.26 | 9.03E-02 | 4.15E-03 | 204402 | 7.94E-105 | 0.25 | -1.61E-03 | 1.11E-03 | 326224 | 1.50E-01 | 473.01 |
| rs2027469 | 1 | 159667190 | A | G | 0.18 | -8.41E-02 | 4.89E-03 | 204402 | 3.21E-66 | 0.19 | -6.12E-04 | 1.22E-03 | 326224 | 6.10E-01 | 295.73 |
| rs2794498 | 1 | 159636116 | T | G | 0.26 | 8.71E-02 | 4.05E-03 | 204402 | 8.34E-103 | 0.25 | -1.62E-03 | 1.11E-03 | 326224 | 1.50E-01 | 463.87 |
| rs2794500 | 1 | 159635021 | T | C | 0.36 | 7.03E-02 | 3.69E-03 | 204402 | 9.98E-81 | 0.35 | -1.66E-03 | 1.01E-03 | 326224 | 9.30E-02 | 362.33 |
| rs2794520 | 1 | 159678816 | T | C | 0.33 | -1.82E-01 | 3.71E-03 | 204402 | 1.00E-300 | 0.33 | 4.97E-04 | 1.02E-03 | 326224 | 6.50E-01 | 2408.87 |
| rs2808628 | 1 | 159676011 | A | G | 0.33 | -1.82E-01 | 3.86E-03 | 204402 | 1.00E-300 | 0.33 | 4.68E-04 | 1.02E-03 | 326224 | 6.70E-01 | 2238.23 |
| rs3093075 | 1 | 159679913 | T | G | 0.06 | 2.26E-01 | 7.39E-03 | 204402 | 2.02E-204 | 0.06 | -1.74E-03 | 2.08E-03 | 326224 | 4.10E-01 | 931.16 |
| rs4131568 | 1 | 159722056 | T | C | 0.36 | 9.70E-02 | 3.82E-03 | 204402 | 4.89E-142 | 0.34 | -4.38E-04 | 1.01E-03 | 326224 | 6.90E-01 | 644.29 |
| rs4255379 | 1 | 159718312 | A | G | 0.63 | 6.39E-02 | 3.82E-03 | 204402 | 1.35E-62 | 0.61 | -7.98E-05 | 9.83E-04 | 326224 | 9.50E-01 | 279.05 |
| rs4512645 | 1 | 159729047 | A | G | 0.35 | 9.95E-02 | 3.93E-03 | 204402 | 8.22E-142 | 0.34 | -5.84E-04 | 1.02E-03 | 326224 | 5.80E-01 | 643.16 |
| rs7553007 | 1 | 159698549 | A | G | 0.32 | -1.82E-01 | 3.86E-03 | 204402 | 1.00E-300 | 0.33 | 3.89E-04 | 1.02E-03 | 326224 | 7.30E-01 | 2220.29 |
| rs876537 | 1 | 159674933 | T | C | 0.38 | -1.70E-01 | 3.67E-03 | 204402 | 1.00E-300 | 0.39 | -4.90E-04 | 9.83E-04 | 326224 | 6.00E-01 | 2137.45 |
| rs895582 | 1 | 159637919 | A | G | 0.26 | 8.99E-02 | 4.09E-03 | 204402 | 3.79E-107 | 0.25 | -1.75E-03 | 1.11E-03 | 326224 | 1.20E-01 | 483.62 |

Instrumental cis SNPs (50K) select for CRP to Sleep classical Mendelian randomization analysis. CHR: chromosome; POS: hg19 genomic position; A1: effective allele; A2: the other allele; A1_exp_frq: A1 frequency in the exposure dataset; Beta_exp, effect of A1 for the exposure; SE_exp; standard error for Beta_exp; N_exp: sample size for exposure; P_exp, association p value for exposure; A1_out_frq: A1 frequency in the outcome dataset; Beta_out: effect size of A1 on outcome; SE_out: standard error of Beta_out; N_out: sample size for outcome; P_out: association p value for outcome; F-stats: F-statistics.

# **Table S18. Harmonized instrumental cisSNPs (p<5x10^-8^) for sIL6R to Insomnia MR.**

| SNP | CHR | POS | A1 | A2 | A1_exp_frq | Beta_exp | SE_exp | N_exp | P_exp | A1_out_frq | Beta_out | SE_out | N_out | P_out | Fstats |
| --- | --- | --- | --- | --- | --- | --- | --- | --- | --- | --- | --- | --- | --- | --- | --- |
| rs10047079 | 1 | 154468135 | T | C | 0.77 | 5.88E-01 | 5.40E-03 | 69970 | 1.00E-300 | 0.81 | -3.99E-03 | 6.46E-03 | 384198 | 5.21E-01 | 11836.63 |
| rs1073907 | 1 | 154332520 | T | C | 0.30 | 1.53E-01 | 8.10E-03 | 35570.9 | 3.20E-79 | 0.30 | 5.98E-03 | 5.54E-03 | 386264 | 3.11E-01 | 357.26 |
| rs10908839 | 1 | 154430798 | C | G | 0.58 | -6.11E-01 | 5.20E-03 | 70935 | 1.00E-300 | 0.78 | -1.00E-03 | 6.18E-03 | 385821 | 8.54E-01 | 13801.73 |
| rs10908840 | 1 | 154459477 | T | C | 0.44 | 1.21E-01 | 5.70E-03 | 70929.9 | 2.23E-101 | 0.48 | -9.00E-04 | 5.11E-03 | 383459 | 8.66E-01 | 449.89 |
| rs111885536 | 1 | 154461260 | A | G | 0.19 | -2.11E-01 | 7.00E-03 | 70935 | 1.26E-202 | 0.16 | 3.00E-03 | 6.95E-03 | 384756 | 6.74E-01 | 910.32 |
| rs11265606 | 1 | 154356459 | T | C | 0.28 | 1.80E-01 | 6.20E-03 | 70934.9 | 4.84E-186 | 0.29 | 3.99E-03 | 5.62E-03 | 383176 | 4.97E-01 | 844.75 |
| rs11265607 | 1 | 154357678 | A | G | 0.68 | -1.83E-01 | 6.20E-03 | 70934.9 | 3.48E-191 | 0.71 | -3.99E-03 | 5.62E-03 | 384297 | 4.75E-01 | 869.30 |
| rs11265621 | 1 | 154442960 | A | G | 0.46 | 5.41E-01 | 4.30E-03 | 70934.9 | 1.00E-300 | 0.65 | -4.99E-03 | 5.35E-03 | 382027 | 3.40E-01 | 15811.60 |
| rs11265622 | 1 | 154451420 | A | G | 0.53 | -5.37E-01 | 4.30E-03 | 69970 | 1.00E-300 | 0.35 | 4.99E-03 | 5.34E-03 | 383410 | 3.63E-01 | 15578.52 |
| rs113624284 | 1 | 154339299 | A | G | 0.79 | -2.60E-01 | 7.80E-03 | 70935 | 1.49E-242 | 0.85 | -3.00E-03 | 7.16E-03 | 385183 | 7.00E-01 | 1108.55 |
| rs11576181 | 1 | 154330659 | T | G | 0.67 | -1.23E-01 | 6.10E-03 | 70934.9 | 1.70E-89 | 0.70 | -5.98E-03 | 5.56E-03 | 384582 | 3.02E-01 | 405.26 |
| rs11580535 | 1 | 154364317 | T | G | 0.13 | 3.55E-01 | 8.00E-03 | 70935 | 1.00E-300 | 0.14 | -2.70E-03 | 7.41E-03 | 384656 | 7.13E-01 | 1965.81 |
| rs11582433 | 1 | 154349605 | T | C | 0.14 | 2.70E-01 | 7.70E-03 | 70935 | 4.44E-269 | 0.15 | 2.00E-03 | 7.08E-03 | 386470 | 7.73E-01 | 1233.20 |
| rs11590203 | 1 | 154333569 | T | G | 0.14 | 2.50E-01 | 7.90E-03 | 69970 | 2.44E-222 | 0.15 | 2.00E-03 | 7.17E-03 | 386130 | 8.09E-01 | 1002.24 |
| rs116088025 | 1 | 154354350 | T | C | 0.14 | 2.73E-01 | 7.70E-03 | 70935 | 3.37E-273 | 0.15 | 2.00E-03 | 7.08E-03 | 386307 | 7.91E-01 | 1259.79 |
| rs12023772 | 1 | 154483868 | A | G | 0.17 | 7.12E-01 | 5.50E-03 | 70934.9 | 1.00E-300 | 0.16 | -6.52E-03 | 6.94E-03 | 385943 | 3.45E-01 | 16749.07 |
| rs12025518 | 1 | 154340789 | A | C | 0.69 | 1.92E-01 | 6.30E-03 | 69969 | 1.00E-200 | 0.74 | 8.94E-03 | 5.83E-03 | 383456 | 1.24E-01 | 926.86 |
| rs12033701 | 1 | 154365886 | T | C | 0.11 | -1.66E-01 | 8.70E-03 | 70934 | 1.23E-81 | 0.10 | -3.21E-03 | 8.39E-03 | 386463 | 7.07E-01 | 364.06 |
| rs12044132 | 1 | 154462360 | T | C | 0.15 | 6.85E-01 | 9.60E-03 | 34606 | 1.00E-300 | 0.16 | -6.52E-03 | 6.93E-03 | 386533 | 3.50E-01 | 5086.96 |
| rs12061599 | 1 | 154344135 | T | G | 0.28 | 1.29E-01 | 6.20E-03 | 70935 | 5.38E-98 | 0.30 | 5.98E-03 | 5.55E-03 | 385786 | 2.75E-01 | 434.92 |
| rs12075836 | 1 | 154371487 | T | C | 0.14 | -2.62E-01 | 7.70E-03 | 70934.9 | 8.28E-252 | 0.15 | -1.04E-02 | 7.26E-03 | 382126 | 1.53E-01 | 1158.65 |
| rs12118018 | 1 | 154477440 | A | G | 0.46 | 5.36E-01 | 4.30E-03 | 70934.9 | 1.00E-300 | 0.64 | -3.99E-03 | 5.33E-03 | 383854 | 4.03E-01 | 15555.31 |
| rs12118721 | 1 | 154397416 | T | C | 0.55 | -8.09E-01 | 3.90E-03 | 70933.9 | 1.00E-300 | 0.43 | 5.98E-03 | 5.20E-03 | 378538 | 2.77E-01 | 42976.48 |
| rs12119111 | 1 | 154478600 | A | G | 0.46 | 5.37E-01 | 4.30E-03 | 70934.9 | 1.00E-300 | 0.65 | -3.99E-03 | 5.33E-03 | 384147 | 4.38E-01 | 15578.52 |
| rs12129500 | 1 | 154423764 | T | C | 0.56 | -8.43E-01 | 3.90E-03 | 70934.9 | 1.00E-300 | 0.42 | 4.99E-03 | 5.15E-03 | 386374 | 3.73E-01 | 46711.40 |
| rs12133641 | 1 | 154428283 | A | G | 0.57 | -1.01E+00 | 3.40E-03 | 70935 | 1.00E-300 | 0.59 | 7.53E-03 | 5.17E-03 | 386179 | 1.43E-01 | 88401.28 |
| rs12563459 | 1 | 154362686 | A | G | 0.15 | -1.85E-01 | 7.60E-03 | 70934.9 | 1.95E-130 | 0.15 | -8.84E-03 | 7.10E-03 | 384453 | 2.14E-01 | 594.46 |
| rs12568083 | 1 | 154455949 | T | C | 0.39 | -5.02E-01 | 7.20E-03 | 34606 | 1.00E-300 | 0.36 | 4.99E-03 | 5.34E-03 | 383550 | 3.73E-01 | 4853.44 |
| rs12739228 | 1 | 154426190 | A | G | 0.04 | -6.32E-01 | 1.59E-02 | 68557 | 1.00E-300 | 0.04 | 1.69E-02 | 1.36E-02 | 383755 | 2.27E-01 | 1578.44 |
| rs12753254 | 1 | 154416935 | A | G | 0.41 | 9.76E-01 | 3.50E-03 | 70934.9 | 1.00E-300 | 0.42 | -7.83E-03 | 5.18E-03 | 383552 | 1.31E-01 | 77745.37 |
| rs12753666 | 1 | 154474875 | A | G | 0.46 | 5.36E-01 | 4.30E-03 | 70934.9 | 1.00E-300 | 0.64 | -3.99E-03 | 5.34E-03 | 383667 | 4.07E-01 | 15537.91 |
| rs138765671 | 1 | 154345962 | A | C | 0.14 | 2.61E-01 | 7.80E-03 | 70935 | 2.75E-243 | 0.15 | 3.00E-03 | 7.18E-03 | 385869 | 6.79E-01 | 1123.11 |
| rs147763778 | 1 | 154368223 | A | G | 0.04 | 3.99E-01 | 1.60E-02 | 68557 | 3.27E-138 | 0.04 | -2.06E-02 | 1.37E-02 | 378573 | 1.32E-01 | 623.13 |
| rs1889313 | 1 | 154351717 | A | C | 0.14 | 2.70E-01 | 7.70E-03 | 70935 | 6.23E-269 | 0.15 | 2.00E-03 | 7.07E-03 | 386533 | 7.60E-01 | 1233.20 |
| rs2229238 | 1 | 154437896 | T | C | 0.58 | -5.86E-01 | 5.50E-03 | 69970 | 1.00E-300 | 0.18 | 4.99E-03 | 6.55E-03 | 386533 | 4.18E-01 | 11336.44 |
| rs34094138 | 1 | 154366911 | T | G | 0.78 | 1.86E-01 | 7.60E-03 | 70935 | 1.20E-131 | 0.85 | 8.44E-03 | 7.10E-03 | 384246 | 2.35E-01 | 597.03 |
| rs35717427 | 1 | 154391882 | A | G | 0.12 | 4.99E-01 | 8.30E-03 | 69968.9 | 1.00E-300 | 0.12 | -4.31E-03 | 7.74E-03 | 386533 | 5.80E-01 | 3607.23 |
| rs4240872 | 1 | 154436195 | T | C | 0.39 | 6.19E-01 | 5.00E-03 | 70935 | 1.00E-300 | 0.77 | -2.00E-03 | 6.08E-03 | 386533 | 7.44E-01 | 15331.39 |
| rs4393147 | 1 | 154414037 | T | C | 0.41 | 9.75E-01 | 3.50E-03 | 70934.9 | 1.00E-300 | 0.42 | -8.13E-03 | 5.18E-03 | 383486 | 1.18E-01 | 77554.29 |
| rs4474240 | 1 | 154457855 | A | C | 0.47 | -2.14E-01 | 7.00E-03 | 69970 | 5.07E-206 | 0.16 | 3.00E-03 | 6.94E-03 | 384727 | 6.80E-01 | 933.74 |
| rs4478801 | 1 | 154464572 | A | G | 0.58 | 5.01E-01 | 7.20E-03 | 34597.9 | 1.00E-300 | 0.64 | -4.99E-03 | 5.34E-03 | 383565 | 3.67E-01 | 4843.77 |
| rs4521987 | 1 | 154388668 | T | C | 0.45 | -2.73E-01 | 7.90E-03 | 69968.9 | 8.69E-265 | 0.14 | -9.04E-03 | 7.43E-03 | 373340 | 2.23E-01 | 1196.81 |
| rs45478197 | 1 | 154422733 | T | C | 0.10 | -2.03E-01 | 9.00E-03 | 70935 | 2.60E-113 | 0.08 | 8.96E-03 | 9.46E-03 | 383510 | 3.48E-01 | 508.75 |
| rs4553185 | 1 | 154410955 | T | C | 0.44 | 8.30E-01 | 3.90E-03 | 69969.9 | 1.00E-300 | 0.58 | -6.98E-03 | 5.17E-03 | 383931 | 2.01E-01 | 45281.66 |
| rs4845372 | 1 | 154415396 | A | C | 0.42 | 9.40E-01 | 3.60E-03 | 70934.9 | 1.00E-300 | 0.43 | -7.33E-03 | 5.16E-03 | 383571 | 1.58E-01 | 68164.51 |
| rs4845617 | 1 | 154377898 | A | G | 0.39 | 1.25E-01 | 5.70E-03 | 69965.8 | 1.71E-105 | 0.40 | 3.99E-03 | 5.27E-03 | 374979 | 5.05E-01 | 480.92 |
| rs4845618 | 1 | 154400015 | T | G | 0.44 | 8.09E-01 | 3.90E-03 | 70934 | 1.00E-300 | 0.57 | -5.98E-03 | 5.16E-03 | 384012 | 2.46E-01 | 42997.74 |
| rs4845637 | 1 | 154490178 | A | G | 0.53 | -5.36E-01 | 4.30E-03 | 70934.9 | 1.00E-300 | 0.36 | 3.99E-03 | 5.33E-03 | 384686 | 4.04E-01 | 15526.32 |
| rs57569414 | 1 | 154380419 | A | C | 0.12 | 4.79E-01 | 8.40E-03 | 69969.9 | 1.00E-300 | 0.12 | -2.30E-03 | 7.95E-03 | 380301 | 7.75E-01 | 3250.36 |
| rs59632925 | 1 | 154406540 | T | G | 0.56 | -8.16E-01 | 3.90E-03 | 70934.9 | 1.00E-300 | 0.41 | 5.98E-03 | 5.17E-03 | 385056 | 2.23E-01 | 43788.25 |
| rs6427631 | 1 | 154370020 | T | C | 0.68 | -1.84E-01 | 6.20E-03 | 70934.9 | 2.06E-193 | 0.71 | -3.00E-03 | 5.64E-03 | 381767 | 5.67E-01 | 878.84 |
| rs6657938 | 1 | 154336126 | A | G | 0.67 | -1.30E-01 | 6.10E-03 | 70935 | 4.00E-100 | 0.70 | -5.98E-03 | 5.54E-03 | 386505 | 2.84E-01 | 455.58 |
| rs6664039 | 1 | 154337238 | A | G | 0.79 | -2.58E-01 | 7.80E-03 | 70935 | 8.02E-240 | 0.85 | -2.00E-03 | 7.15E-03 | 385646 | 7.38E-01 | 1096.63 |
| rs6664608 | 1 | 154479670 | T | C | 0.46 | 5.36E-01 | 4.30E-03 | 70934.9 | 1.00E-300 | 0.64 | -3.99E-03 | 5.33E-03 | 384341 | 4.39E-01 | 15549.51 |
| rs66654715 | 1 | 154376820 | C | G | 0.03 | -5.54E-01 | 1.76E-02 | 68557 | 9.70E-218 | 0.03 | 5.98E-03 | 1.54E-02 | 381033 | 7.19E-01 | 991.53 |
| rs6667434 | 1 | 154409100 | A | G | 0.56 | -8.17E-01 | 3.90E-03 | 70934.9 | 1.00E-300 | 0.41 | 5.98E-03 | 5.18E-03 | 384076 | 2.37E-01 | 43863.40 |
| rs6674171 | 1 | 154491683 | A | G | 0.77 | 5.88E-01 | 5.40E-03 | 69970 | 1.00E-300 | 0.81 | -3.99E-03 | 6.46E-03 | 384336 | 5.26E-01 | 11836.63 |
| rs6675472 | 1 | 154445503 | T | C | 0.58 | -5.88E-01 | 5.30E-03 | 70935 | 1.00E-300 | 0.19 | 4.99E-03 | 6.48E-03 | 383790 | 4.70E-01 | 12287.51 |
| rs6683206 | 1 | 154418088 | T | C | 0.56 | -8.25E-01 | 3.90E-03 | 70935 | 1.00E-300 | 0.41 | 4.99E-03 | 5.19E-03 | 383775 | 3.02E-01 | 44759.37 |
| rs6686467 | 1 | 154329095 | A | G | 0.14 | 2.52E-01 | 7.80E-03 | 70935 | 5.53E-228 | 0.15 | 3.00E-03 | 7.18E-03 | 385191 | 7.27E-01 | 1041.30 |
| rs6686750 | 1 | 154419843 | A | G | 0.55 | -8.42E-01 | 3.90E-03 | 70934.9 | 1.00E-300 | 0.42 | 3.99E-03 | 5.16E-03 | 385538 | 4.06E-01 | 46633.85 |
| rs6687597 | 1 | 154434936 | A | G | 0.39 | 6.20E-01 | 5.00E-03 | 70935 | 1.00E-300 | 0.77 | -2.00E-03 | 6.09E-03 | 386461 | 7.59E-01 | 15351.21 |
| rs6694817 | 1 | 154401972 | T | C | 0.56 | -7.97E-01 | 4.00E-03 | 70932.9 | 1.00E-300 | 0.42 | 4.99E-03 | 5.17E-03 | 384104 | 3.16E-01 | 39740.42 |
| rs6700296 | 1 | 154473660 | T | C | 0.53 | -5.36E-01 | 4.30E-03 | 70934.9 | 1.00E-300 | 0.36 | 4.99E-03 | 5.34E-03 | 383666 | 3.82E-01 | 15537.91 |
| rs72633646 | 1 | 154334683 | A | G | 0.14 | 2.53E-01 | 7.80E-03 | 70935 | 1.75E-230 | 0.15 | 2.00E-03 | 7.17E-03 | 386149 | 8.10E-01 | 1053.75 |
| rs72633650 | 1 | 154360838 | T | C | 0.79 | -3.54E-01 | 8.00E-03 | 70934 | 1.00E-300 | 0.86 | 1.80E-03 | 7.41E-03 | 384757 | 8.06E-01 | 1955.85 |
| rs72698115 | 1 | 154379369 | A | C | 0.82 | 1.79E-01 | 8.80E-03 | 70934 | 1.14E-92 | 0.90 | 3.41E-03 | 8.59E-03 | 380424 | 6.92E-01 | 412.37 |
| rs72698169 | 1 | 154486799 | A | C | 0.78 | -7.11E-01 | 5.50E-03 | 70934.9 | 1.00E-300 | 0.84 | 7.02E-03 | 6.94E-03 | 385788 | 3.13E-01 | 16697.34 |
| rs73018293 | 1 | 154465577 | T | C | 0.20 | -5.86E-01 | 5.30E-03 | 70934.9 | 1.00E-300 | 0.19 | 3.99E-03 | 6.46E-03 | 384222 | 5.13E-01 | 12233.19 |
| rs73020232 | 1 | 154482669 | T | C | 0.20 | -5.86E-01 | 5.30E-03 | 70934.9 | 1.00E-300 | 0.19 | 3.99E-03 | 6.46E-03 | 384264 | 5.52E-01 | 12237.37 |
| rs73020246 | 1 | 154485640 | A | G | 0.77 | 5.87E-01 | 5.30E-03 | 70934.9 | 1.00E-300 | 0.81 | -3.99E-03 | 6.46E-03 | 384272 | 5.24E-01 | 12258.25 |
| rs7513603 | 1 | 154481158 | T | C | 0.46 | -2.13E-01 | 6.90E-03 | 70934.9 | 5.71E-206 | 0.16 | 3.00E-03 | 6.93E-03 | 385766 | 6.73E-01 | 951.14 |
| rs7519499 | 1 | 154487926 | A | G | 0.46 | 5.36E-01 | 4.30E-03 | 70934.9 | 1.00E-300 | 0.65 | -3.99E-03 | 5.33E-03 | 384630 | 4.12E-01 | 15555.31 |
| rs7521458 | 1 | 154407713 | T | C | 0.57 | -9.71E-01 | 3.50E-03 | 70934.9 | 1.00E-300 | 0.58 | 8.03E-03 | 5.18E-03 | 382943 | 1.23E-01 | 76966.61 |
| rs7526131 | 1 | 154425135 | A | G | 0.44 | 8.44E-01 | 3.90E-03 | 70935 | 1.00E-300 | 0.58 | -4.99E-03 | 5.15E-03 | 386195 | 3.70E-01 | 46811.21 |
| rs7537291 | 1 | 154433407 | A | G | 0.39 | 6.21E-01 | 5.00E-03 | 70935 | 1.00E-300 | 0.77 | -2.00E-03 | 6.09E-03 | 386439 | 7.21E-01 | 15410.74 |
| rs7549250 | 1 | 154404336 | T | C | 0.44 | 8.26E-01 | 3.90E-03 | 70933 | 1.00E-300 | 0.58 | -6.98E-03 | 5.15E-03 | 386533 | 1.96E-01 | 44878.79 |
| rs7553271 | 1 | 154373231 | T | C | 0.49 | 1.26E-01 | 5.70E-03 | 70933.9 | 1.13E-108 | 0.40 | 2.00E-03 | 5.24E-03 | 379056 | 6.56E-01 | 487.87 |
| rs79794939 | 1 | 154390932 | T | C | 0.07 | -3.75E-01 | 1.06E-02 | 70930 | 5.13E-272 | 0.08 | -1.24E-02 | 9.58E-03 | 386533 | 1.97E-01 | 1248.89 |
| rs9651053 | 1 | 154359411 | A | G | 0.11 | -1.66E-01 | 8.70E-03 | 70934 | 4.13E-82 | 0.10 | -3.51E-03 | 8.40E-03 | 386307 | 6.78E-01 | 365.82 |
| rs9803896 | 1 | 154347450 | A | G | 0.14 | 2.74E-01 | 7.70E-03 | 70935 | 7.89E-275 | 0.15 | 1.00E-03 | 7.11E-03 | 385282 | 8.59E-01 | 1269.95 |

Instrumental cis SNPs (50K) select for CRP to Sleep classical Mendelian randomization analysis. CHR: chromosome; POS: hg19 genomic position; A1: effective allele; A2: the other allele; A1_exp_frq: A1 frequency in the exposure dataset; Beta_exp, effect of A1 for the exposure; SE_exp; standard error for Beta_exp; N_exp: sample size for exposure; P_exp, association p value for exposure; A1_out_frq: A1 frequency in the outcome dataset; Beta_out: effect size of A1 on outcome; SE_out: standard error of Beta_out; N_out: sample size for outcome; P_out: association p value for outcome; F-stats: F-statistics.

# **Table S19. Harmonized instrumental cisSNPs (p<5x10^-8^) for sIL6R to Sleepiness MR.**

| SNP | CHR | POS | A1 | A2 | A1_exp_frq | Beta_exp | SE_exp | N_exp | P_exp | A1_out_frq | Beta_out | SE_out | N_out | P_out | Fstats |
| --- | --- | --- | --- | --- | --- | --- | --- | --- | --- | --- | --- | --- | --- | --- | --- |
| rs10047079 | 1 | 154468135 | T | C | 0.77 | 5.88E-01 | 5.40E-03 | 69970 | 1.00E-300 | 0.81 | -9.46E-04 | 1.30E-03 | 452071 | 4.30E-01 | 11836.63 |
| rs1073907 | 1 | 154332520 | T | C | 0.30 | 1.53E-01 | 8.10E-03 | 35570.9 | 3.20E-79 | 0.30 | -2.16E-03 | 1.12E-03 | 452071 | 5.00E-02 | 357.26 |
| rs10752641 | 1 | 154432042 | C | G | 0.27 | 6.44E-01 | 5.80E-03 | 49179 | 1.00E-300 | 0.22 | 5.22E-04 | 1.23E-03 | 452071 | 6.30E-01 | 12317.17 |
| rs10908839 | 1 | 154430798 | C | G | 0.58 | -6.11E-01 | 5.20E-03 | 70935 | 1.00E-300 | 0.78 | -6.46E-04 | 1.25E-03 | 452071 | 5.70E-01 | 13801.73 |
| rs10908840 | 1 | 154459477 | T | C | 0.44 | 1.21E-01 | 5.70E-03 | 70929.9 | 2.23E-101 | 0.48 | -3.58E-05 | 1.03E-03 | 452071 | 9.40E-01 | 449.89 |
| rs111885536 | 1 | 154461260 | A | G | 0.19 | -2.11E-01 | 7.00E-03 | 70935 | 1.26E-202 | 0.16 | 1.58E-03 | 1.40E-03 | 452071 | 2.70E-01 | 910.32 |
| rs11265606 | 1 | 154356459 | T | C | 0.28 | 1.80E-01 | 6.20E-03 | 70934.9 | 4.84E-186 | 0.29 | -2.24E-03 | 1.13E-03 | 452071 | 4.60E-02 | 844.75 |
| rs11265607 | 1 | 154357678 | A | G | 0.68 | -1.83E-01 | 6.20E-03 | 70934.9 | 3.48E-191 | 0.71 | 2.31E-03 | 1.13E-03 | 452071 | 4.00E-02 | 869.30 |
| rs11265621 | 1 | 154442960 | A | G | 0.46 | 5.41E-01 | 4.30E-03 | 70934.9 | 1.00E-300 | 0.64 | -1.54E-03 | 1.07E-03 | 452071 | 1.40E-01 | 15811.60 |
| rs11265622 | 1 | 154451420 | A | G | 0.53 | -5.37E-01 | 4.30E-03 | 69970 | 1.00E-300 | 0.36 | 1.61E-03 | 1.07E-03 | 452071 | 1.30E-01 | 15578.52 |
| rs113624284 | 1 | 154339299 | A | G | 0.79 | -2.60E-01 | 7.80E-03 | 70935 | 1.49E-242 | 0.85 | 1.67E-03 | 1.44E-03 | 452071 | 2.30E-01 | 1108.55 |
| rs11576181 | 1 | 154330659 | T | G | 0.67 | -1.23E-01 | 6.10E-03 | 70934.9 | 1.70E-89 | 0.70 | 2.19E-03 | 1.12E-03 | 452071 | 4.70E-02 | 405.26 |
| rs11580178 | 1 | 154353262 | A | T | 0.29 | 1.21E-01 | 7.60E-03 | 49179 | 3.04E-57 | 0.31 | -2.16E-03 | 1.11E-03 | 452071 | 5.00E-02 | 253.48 |
| rs11580535 | 1 | 154364317 | T | G | 0.13 | 3.55E-01 | 8.00E-03 | 70935 | 1.00E-300 | 0.14 | -1.69E-03 | 1.49E-03 | 452071 | 2.50E-01 | 1965.81 |
| rs11582433 | 1 | 154349605 | T | C | 0.14 | 2.70E-01 | 7.70E-03 | 70935 | 4.44E-269 | 0.15 | -1.52E-03 | 1.43E-03 | 452071 | 2.80E-01 | 1233.20 |
| rs11590203 | 1 | 154333569 | T | G | 0.14 | 2.50E-01 | 7.90E-03 | 69970 | 2.44E-222 | 0.15 | -1.71E-03 | 1.44E-03 | 452071 | 2.30E-01 | 1002.24 |
| rs116088025 | 1 | 154354350 | T | C | 0.14 | 2.73E-01 | 7.70E-03 | 70935 | 3.37E-273 | 0.15 | -1.52E-03 | 1.43E-03 | 452071 | 2.80E-01 | 1259.79 |
| rs12023772 | 1 | 154483868 | A | G | 0.17 | 7.12E-01 | 5.50E-03 | 70934.9 | 1.00E-300 | 0.16 | -2.63E-03 | 1.40E-03 | 452071 | 6.20E-02 | 16749.07 |
| rs12025518 | 1 | 154340789 | A | C | 0.69 | 1.92E-01 | 6.30E-03 | 69969 | 1.00E-200 | 0.74 | 1.47E-03 | 1.17E-03 | 452071 | 2.00E-01 | 926.86 |
| rs12033701 | 1 | 154365886 | T | C | 0.11 | -1.66E-01 | 8.70E-03 | 70934 | 1.23E-81 | 0.10 | 1.72E-04 | 1.69E-03 | 452071 | 9.30E-01 | 364.06 |
| rs12044132 | 1 | 154462360 | T | C | 0.15 | 6.85E-01 | 9.60E-03 | 34606 | 1.00E-300 | 0.16 | -2.80E-03 | 1.40E-03 | 452071 | 4.70E-02 | 5086.96 |
| rs12061599 | 1 | 154344135 | T | G | 0.28 | 1.29E-01 | 6.20E-03 | 70935 | 5.38E-98 | 0.30 | -2.16E-03 | 1.12E-03 | 452071 | 4.90E-02 | 434.92 |
| rs12075836 | 1 | 154371487 | T | C | 0.14 | -2.62E-01 | 7.70E-03 | 70934.9 | 8.28E-252 | 0.15 | -1.75E-03 | 1.45E-03 | 452071 | 2.20E-01 | 1158.65 |
| rs12118018 | 1 | 154477440 | A | G | 0.46 | 5.36E-01 | 4.30E-03 | 70934.9 | 1.00E-300 | 0.64 | -1.62E-03 | 1.07E-03 | 452071 | 1.20E-01 | 15555.31 |
| rs12118721 | 1 | 154397416 | T | C | 0.55 | -8.09E-01 | 3.90E-03 | 70933.9 | 1.00E-300 | 0.43 | -6.99E-05 | 1.04E-03 | 452071 | 9.80E-01 | 42976.48 |
| rs12119111 | 1 | 154478600 | A | G | 0.46 | 5.37E-01 | 4.30E-03 | 70934.9 | 1.00E-300 | 0.64 | -1.57E-03 | 1.07E-03 | 452071 | 1.40E-01 | 15578.52 |
| rs12129500 | 1 | 154423764 | T | C | 0.56 | -8.43E-01 | 3.90E-03 | 70934.9 | 1.00E-300 | 0.42 | 5.61E-04 | 1.04E-03 | 452071 | 5.80E-01 | 46711.40 |
| rs12133641 | 1 | 154428283 | A | G | 0.57 | -1.01E+00 | 3.40E-03 | 70935 | 1.00E-300 | 0.59 | 1.50E-03 | 1.04E-03 | 452071 | 1.50E-01 | 88401.28 |
| rs12563459 | 1 | 154362686 | A | G | 0.15 | -1.85E-01 | 7.60E-03 | 70934.9 | 1.95E-130 | 0.15 | -2.01E-03 | 1.42E-03 | 452071 | 1.50E-01 | 594.46 |
| rs12568083 | 1 | 154455949 | T | C | 0.39 | -5.02E-01 | 7.20E-03 | 34606 | 1.00E-300 | 0.36 | 1.62E-03 | 1.07E-03 | 452071 | 1.20E-01 | 4853.44 |
| rs12753254 | 1 | 154416935 | A | G | 0.41 | 9.76E-01 | 3.50E-03 | 70934.9 | 1.00E-300 | 0.42 | -1.24E-03 | 1.04E-03 | 452071 | 2.30E-01 | 77745.37 |
| rs12753666 | 1 | 154474875 | A | G | 0.46 | 5.36E-01 | 4.30E-03 | 70934.9 | 1.00E-300 | 0.64 | -1.63E-03 | 1.07E-03 | 452071 | 1.20E-01 | 15537.91 |
| rs138765671 | 1 | 154345962 | A | C | 0.14 | 2.61E-01 | 7.80E-03 | 70935 | 2.75E-243 | 0.15 | -1.63E-03 | 1.45E-03 | 452071 | 2.50E-01 | 1123.11 |
| rs1889313 | 1 | 154351717 | A | C | 0.14 | 2.70E-01 | 7.70E-03 | 70935 | 6.23E-269 | 0.15 | -1.52E-03 | 1.43E-03 | 452071 | 2.80E-01 | 1233.20 |
| rs2229238 | 1 | 154437896 | T | C | 0.58 | -5.86E-01 | 5.50E-03 | 69970 | 1.00E-300 | 0.18 | 5.57E-04 | 1.32E-03 | 452071 | 6.30E-01 | 11336.44 |
| rs34094138 | 1 | 154366911 | T | G | 0.78 | 1.86E-01 | 7.60E-03 | 70935 | 1.20E-131 | 0.85 | 2.04E-03 | 1.42E-03 | 452071 | 1.50E-01 | 597.03 |
| rs35717427 | 1 | 154391882 | A | G | 0.12 | 4.99E-01 | 8.30E-03 | 69968.9 | 1.00E-300 | 0.12 | -2.40E-03 | 1.56E-03 | 452071 | 1.20E-01 | 3607.23 |
| rs4240872 | 1 | 154436195 | T | C | 0.39 | 6.19E-01 | 5.00E-03 | 70935 | 1.00E-300 | 0.77 | -6.42E-04 | 1.23E-03 | 452071 | 5.60E-01 | 15331.39 |
| rs4379670 | 1 | 154439865 | A | T | 0.24 | 6.12E-01 | 6.40E-03 | 49179 | 1.00E-300 | 0.18 | 5.31E-04 | 1.33E-03 | 452071 | 6.50E-01 | 9138.17 |
| rs4393147 | 1 | 154414037 | T | C | 0.41 | 9.75E-01 | 3.50E-03 | 70934.9 | 1.00E-300 | 0.42 | -1.21E-03 | 1.04E-03 | 452071 | 2.40E-01 | 77554.29 |
| rs4474240 | 1 | 154457855 | A | C | 0.47 | -2.14E-01 | 7.00E-03 | 69970 | 5.07E-206 | 0.16 | 1.58E-03 | 1.40E-03 | 452071 | 2.70E-01 | 933.74 |
| rs4478801 | 1 | 154464572 | A | G | 0.58 | 5.01E-01 | 7.20E-03 | 34597.9 | 1.00E-300 | 0.64 | -1.63E-03 | 1.07E-03 | 452071 | 1.20E-01 | 4843.77 |
| rs4521987 | 1 | 154388668 | T | C | 0.45 | -2.73E-01 | 7.90E-03 | 69968.9 | 8.69E-265 | 0.15 | -1.28E-03 | 1.47E-03 | 452071 | 3.80E-01 | 1196.81 |
| rs45478197 | 1 | 154422733 | T | C | 0.10 | -2.03E-01 | 9.00E-03 | 70935 | 2.60E-113 | 0.08 | -1.89E-03 | 1.89E-03 | 452071 | 3.10E-01 | 508.75 |
| rs4553185 | 1 | 154410955 | T | C | 0.44 | 8.30E-01 | 3.90E-03 | 69969.9 | 1.00E-300 | 0.58 | -1.75E-04 | 1.04E-03 | 452071 | 8.40E-01 | 45281.66 |
| rs4845372 | 1 | 154415396 | A | C | 0.42 | 9.40E-01 | 3.60E-03 | 70934.9 | 1.00E-300 | 0.43 | -1.26E-03 | 1.04E-03 | 452071 | 2.20E-01 | 68164.51 |
| rs4845374 | 1 | 154426947 | A | T | 0.20 | -2.57E-01 | 8.60E-03 | 49179 | 1.70E-196 | 0.16 | 1.81E-03 | 1.40E-03 | 452071 | 2.00E-01 | 893.73 |
| rs4845617 | 1 | 154377898 | A | G | 0.39 | 1.25E-01 | 5.70E-03 | 69965.8 | 1.71E-105 | 0.40 | -2.18E-03 | 1.05E-03 | 452071 | 3.50E-02 | 480.92 |
| rs4845618 | 1 | 154400015 | T | G | 0.44 | 8.09E-01 | 3.90E-03 | 70934 | 1.00E-300 | 0.57 | 7.59E-05 | 1.04E-03 | 452071 | 9.80E-01 | 42997.74 |
| rs4845637 | 1 | 154490178 | A | G | 0.53 | -5.36E-01 | 4.30E-03 | 70934.9 | 1.00E-300 | 0.36 | 1.63E-03 | 1.07E-03 | 452071 | 1.20E-01 | 15526.32 |
| rs57569414 | 1 | 154380419 | A | C | 0.12 | 4.79E-01 | 8.40E-03 | 69969.9 | 1.00E-300 | 0.12 | -1.61E-03 | 1.59E-03 | 452071 | 2.90E-01 | 3250.36 |
| rs59632925 | 1 | 154406540 | T | G | 0.56 | -8.16E-01 | 3.90E-03 | 70934.9 | 1.00E-300 | 0.41 | 2.30E-04 | 1.04E-03 | 452071 | 8.00E-01 | 43788.25 |
| rs6427631 | 1 | 154370020 | T | C | 0.68 | -1.84E-01 | 6.20E-03 | 70934.9 | 2.06E-193 | 0.71 | 2.34E-03 | 1.13E-03 | 452071 | 3.70E-02 | 878.84 |
| rs6657938 | 1 | 154336126 | A | G | 0.67 | -1.30E-01 | 6.10E-03 | 70935 | 4.00E-100 | 0.70 | 2.10E-03 | 1.12E-03 | 452071 | 5.60E-02 | 455.58 |
| rs6664039 | 1 | 154337238 | A | G | 0.79 | -2.58E-01 | 7.80E-03 | 70935 | 8.02E-240 | 0.85 | 1.67E-03 | 1.44E-03 | 452071 | 2.30E-01 | 1096.63 |
| rs6664608 | 1 | 154479670 | T | C | 0.46 | 5.36E-01 | 4.30E-03 | 70934.9 | 1.00E-300 | 0.64 | -1.63E-03 | 1.07E-03 | 452071 | 1.20E-01 | 15549.51 |
| rs6667434 | 1 | 154409100 | A | G | 0.56 | -8.17E-01 | 3.90E-03 | 70934.9 | 1.00E-300 | 0.41 | 2.39E-04 | 1.04E-03 | 452071 | 7.90E-01 | 43863.40 |
| rs6674171 | 1 | 154491683 | A | G | 0.77 | 5.88E-01 | 5.40E-03 | 69970 | 1.00E-300 | 0.81 | -9.94E-04 | 1.30E-03 | 452071 | 4.10E-01 | 11836.63 |
| rs6675472 | 1 | 154445503 | T | C | 0.58 | -5.88E-01 | 5.30E-03 | 70935 | 1.00E-300 | 0.19 | 6.42E-04 | 1.30E-03 | 452071 | 5.80E-01 | 12287.51 |
| rs6683206 | 1 | 154418088 | T | C | 0.56 | -8.25E-01 | 3.90E-03 | 70935 | 1.00E-300 | 0.41 | 4.11E-04 | 1.04E-03 | 452071 | 6.70E-01 | 44759.37 |
| rs6684439 | 1 | 154395839 | T | C | 0.40 | 9.64E-01 | 3.80E-03 | 62755.9 | 1.00E-300 | 0.41 | -9.73E-04 | 1.06E-03 | 452071 | 3.40E-01 | 64355.68 |
| rs6686467 | 1 | 154329095 | A | G | 0.14 | 2.52E-01 | 7.80E-03 | 70935 | 5.53E-228 | 0.15 | -1.78E-03 | 1.45E-03 | 452071 | 2.10E-01 | 1041.30 |
| rs6686750 | 1 | 154419843 | A | G | 0.55 | -8.42E-01 | 3.90E-03 | 70934.9 | 1.00E-300 | 0.42 | 5.68E-04 | 1.04E-03 | 452071 | 5.70E-01 | 46633.85 |
| rs6687597 | 1 | 154434936 | A | G | 0.39 | 6.20E-01 | 5.00E-03 | 70935 | 1.00E-300 | 0.77 | -6.36E-04 | 1.23E-03 | 452071 | 5.70E-01 | 15351.21 |
| rs6689965 | 1 | 154470606 | A | T | 0.42 | 5.53E-01 | 4.80E-03 | 49179 | 1.00E-300 | 0.36 | 1.62E-03 | 1.07E-03 | 452071 | 1.20E-01 | 13292.17 |
| rs6694817 | 1 | 154401972 | T | C | 0.56 | -7.97E-01 | 4.00E-03 | 70932.9 | 1.00E-300 | 0.42 | -3.95E-05 | 1.04E-03 | 452071 | 1.00E+00 | 39740.42 |
| rs6700296 | 1 | 154473660 | T | C | 0.53 | -5.36E-01 | 4.30E-03 | 70934.9 | 1.00E-300 | 0.36 | 1.61E-03 | 1.07E-03 | 452071 | 1.30E-01 | 15537.91 |
| rs72633646 | 1 | 154334683 | A | G | 0.14 | 2.53E-01 | 7.80E-03 | 70935 | 1.75E-230 | 0.15 | -1.69E-03 | 1.44E-03 | 452071 | 2.30E-01 | 1053.75 |
| rs72633650 | 1 | 154360838 | T | C | 0.79 | -3.54E-01 | 8.00E-03 | 70934 | 1.00E-300 | 0.86 | 1.81E-03 | 1.49E-03 | 452071 | 2.20E-01 | 1955.85 |
| rs72698115 | 1 | 154379369 | A | C | 0.82 | 1.79E-01 | 8.80E-03 | 70934 | 1.14E-92 | 0.90 | 1.38E-05 | 1.70E-03 | 452071 | 9.90E-01 | 412.37 |
| rs72698169 | 1 | 154486799 | A | C | 0.78 | -7.11E-01 | 5.50E-03 | 70934.9 | 1.00E-300 | 0.84 | 2.63E-03 | 1.40E-03 | 452071 | 6.20E-02 | 16697.34 |
| rs73018293 | 1 | 154465577 | T | C | 0.20 | -5.86E-01 | 5.30E-03 | 70934.9 | 1.00E-300 | 0.19 | 9.66E-04 | 1.30E-03 | 452071 | 4.20E-01 | 12233.19 |
| rs73020232 | 1 | 154482669 | T | C | 0.20 | -5.86E-01 | 5.30E-03 | 70934.9 | 1.00E-300 | 0.19 | 1.06E-03 | 1.30E-03 | 452071 | 3.80E-01 | 12237.37 |
| rs73020246 | 1 | 154485640 | A | G | 0.77 | 5.87E-01 | 5.30E-03 | 70934.9 | 1.00E-300 | 0.81 | -1.05E-03 | 1.30E-03 | 452071 | 3.80E-01 | 12258.25 |
| rs7513603 | 1 | 154481158 | T | C | 0.46 | -2.13E-01 | 6.90E-03 | 70934.9 | 5.71E-206 | 0.16 | 1.54E-03 | 1.39E-03 | 452071 | 2.80E-01 | 951.14 |
| rs7519499 | 1 | 154487926 | A | G | 0.46 | 5.36E-01 | 4.30E-03 | 70934.9 | 1.00E-300 | 0.64 | -1.54E-03 | 1.07E-03 | 452071 | 1.40E-01 | 15555.31 |
| rs7521458 | 1 | 154407713 | T | C | 0.57 | -9.71E-01 | 3.50E-03 | 70934.9 | 1.00E-300 | 0.58 | 1.21E-03 | 1.04E-03 | 452071 | 2.40E-01 | 76966.61 |
| rs7525477 | 1 | 154394297 | A | G | 0.42 | -3.30E-01 | 5.80E-03 | 69968.8 | 1.00E-300 | 0.44 | 2.29E-03 | 1.08E-03 | 452071 | 3.00E-02 | 3245.07 |
| rs7526131 | 1 | 154425135 | A | G | 0.44 | 8.44E-01 | 3.90E-03 | 70935 | 1.00E-300 | 0.58 | -5.74E-04 | 1.04E-03 | 452071 | 5.70E-01 | 46811.21 |
| rs7537291 | 1 | 154433407 | A | G | 0.39 | 6.21E-01 | 5.00E-03 | 70935 | 1.00E-300 | 0.77 | -6.06E-04 | 1.23E-03 | 452071 | 5.80E-01 | 15410.74 |
| rs7549250 | 1 | 154404336 | T | C | 0.44 | 8.26E-01 | 3.90E-03 | 70933 | 1.00E-300 | 0.58 | -2.10E-04 | 1.04E-03 | 452071 | 8.10E-01 | 44878.79 |
| rs7553271 | 1 | 154373231 | T | C | 0.49 | 1.26E-01 | 5.70E-03 | 70933.9 | 1.13E-108 | 0.40 | -2.05E-03 | 1.05E-03 | 452071 | 4.80E-02 | 487.87 |
| rs79438587 | 1 | 154342517 | T | C | 0.16 | 3.48E-01 | 7.70E-03 | 69969.9 | 1.00E-300 | 0.17 | 4.42E-03 | 1.40E-03 | 452071 | 1.60E-03 | 2036.71 |
| rs79794939 | 1 | 154390932 | T | C | 0.07 | -3.75E-01 | 1.06E-02 | 70930 | 5.13E-272 | 0.08 | -1.54E-04 | 1.92E-03 | 452071 | 9.30E-01 | 1248.89 |
| rs9651053 | 1 | 154359411 | A | G | 0.11 | -1.66E-01 | 8.70E-03 | 70934 | 4.13E-82 | 0.10 | 1.85E-04 | 1.69E-03 | 452071 | 9.20E-01 | 365.82 |
| rs9803896 | 1 | 154347450 | A | G | 0.14 | 2.74E-01 | 7.70E-03 | 70935 | 7.89E-275 | 0.15 | -1.67E-03 | 1.43E-03 | 452071 | 2.30E-01 | 1269.95 |

Instrumental cis SNPs (50K) select for CRP to Sleep classical Mendelian randomization analysis. CHR: chromosome; POS: hg19 genomic position; A1: effective allele; A2: the other allele; A1_exp_frq: A1 frequency in the exposure dataset; Beta_exp, effect of A1 for the exposure; SE_exp; standard error for Beta_exp; N_exp: sample size for exposure; P_exp, association p value for exposure; A1_out_frq: A1 frequency in the outcome dataset; Beta_out: effect size of A1 on outcome; SE_out: standard error of Beta_out; N_out: sample size for outcome; P_out: association p value for outcome; F-stats: F-statistics.

# **Table S20. Harmonized instrumental cis SNPs (p<5x10^-8^) for sgp130 to Insomnia MR.**

| SNP | CHR | POS | A1 | A2 | A1_exp_frq | Beta_exp | SE_exp | N_exp | P_exp | A1_out_frq | Beta_out | SE_out | N_out | P_out | Fstats |
| --- | --- | --- | --- | --- | --- | --- | --- | --- | --- | --- | --- | --- | --- | --- | --- |
| rs10043068 | 5 | 55201091 | A | G | 0.18 | 8.37E-02 | 9.20E-03 | 45876 | 9.03E-20 | 0.17 | 1.29E-02 | 6.78E-03 | 384389 | 6.56E-02 | 82.77 |
| rs10045084 | 5 | 55333925 | A | G | 0.42 | 7.71E-02 | 7.40E-03 | 45875 | 2.96E-25 | 0.66 | 1.10E-03 | 5.36E-03 | 386533 | 8.33E-01 | 108.55 |
| rs10056283 | 5 | 55330467 | T | C | 0.45 | 1.03E-01 | 7.20E-03 | 45875 | 2.16E-46 | 0.47 | -6.72E-03 | 5.16E-03 | 376495 | 1.94E-01 | 203.06 |
| rs10075152 | 5 | 55284949 | T | C | 0.28 | -7.86E-02 | 7.90E-03 | 45875 | 3.81E-23 | 0.28 | 4.99E-03 | 5.66E-03 | 385726 | 4.06E-01 | 98.99 |
| rs10214033 | 5 | 55327306 | A | G | 0.43 | 9.48E-02 | 7.20E-03 | 45875 | 1.68E-39 | 0.45 | -3.91E-03 | 5.18E-03 | 376079 | 4.52E-01 | 173.36 |
| rs10471419 | 5 | 55251223 | T | C | 0.81 | -3.95E-01 | 1.04E-02 | 45875 | 1.00E-300 | 0.86 | -3.00E-03 | 7.42E-03 | 386338 | 7.25E-01 | 1444.73 |
| rs10805486 | 5 | 55183662 | A | G | 0.47 | 5.00E-02 | 7.20E-03 | 45877 | 2.85E-12 | 0.50 | 4.71E-03 | 5.13E-03 | 381592 | 3.54E-01 | 48.23 |
| rs10940492 | 5 | 55235425 | T | C | 0.82 | -4.16E-01 | 1.12E-02 | 45876 | 1.00E-300 | 0.88 | -1.09E-02 | 7.92E-03 | 386229 | 1.65E-01 | 1381.58 |
| rs1144496 | 5 | 55210985 | T | C | 0.45 | -4.75E-02 | 7.20E-03 | 45876 | 5.04E-11 | 0.56 | 8.54E-03 | 5.13E-03 | 385133 | 9.64E-02 | 43.52 |
| rs11574762 | 5 | 55293271 | T | C | 0.85 | 7.83E-02 | 1.32E-02 | 45875 | 3.01E-09 | 0.93 | 6.82E-03 | 1.00E-02 | 382107 | 4.97E-01 | 35.19 |
| rs11574765 | 5 | 55278967 | A | G | 0.82 | -4.24E-01 | 1.12E-02 | 45875 | 1.00E-300 | 0.88 | -9.95E-03 | 7.93E-03 | 386366 | 2.22E-01 | 1431.81 |
| rs11574769 | 5 | 55258221 | T | C | 0.82 | -4.23E-01 | 1.12E-02 | 45875 | 1.00E-300 | 0.88 | -9.95E-03 | 7.93E-03 | 386404 | 2.09E-01 | 1426.41 |
| rs11574777 | 5 | 55248943 | A | C | 0.11 | 4.22E-01 | 1.12E-02 | 45875 | 1.00E-300 | 0.12 | 1.09E-02 | 7.93E-03 | 386354 | 1.86E-01 | 1419.67 |
| rs11739048 | 5 | 55263495 | T | C | 0.13 | 4.00E-01 | 1.04E-02 | 45875 | 1.00E-300 | 0.13 | 3.00E-03 | 7.46E-03 | 386433 | 7.31E-01 | 1476.33 |
| rs11740906 | 5 | 55319195 | A | G | 0.12 | 3.78E-01 | 1.06E-02 | 45875 | 3.21E-276 | 0.13 | 1.00E-03 | 7.51E-03 | 382487 | 8.89E-01 | 1271.66 |
| rs11741161 | 5 | 55314248 | C | G | 0.12 | 4.00E-01 | 1.10E-02 | 45875 | 1.50E-290 | 0.12 | 8.96E-03 | 7.82E-03 | 385332 | 2.70E-01 | 1322.98 |
| rs11741953 | 5 | 55267814 | T | C | 0.82 | -4.23E-01 | 1.08E-02 | 45875 | 1.00E-300 | 0.88 | -8.96E-03 | 7.84E-03 | 386406 | 2.37E-01 | 1534.75 |
| rs11742754 | 5 | 55298493 | T | C | 0.83 | -4.16E-01 | 1.15E-02 | 45875 | 1.29E-284 | 0.89 | -1.19E-02 | 8.20E-03 | 383664 | 1.39E-01 | 1309.81 |
| rs11747625 | 5 | 55276273 | T | G | 0.11 | 4.23E-01 | 1.12E-02 | 45875 | 1.00E-300 | 0.12 | 9.95E-03 | 7.93E-03 | 386326 | 2.15E-01 | 1429.11 |
| rs11953360 | 5 | 55324944 | A | C | 0.74 | -1.96E-01 | 8.70E-03 | 45875 | 1.04E-112 | 0.78 | -4.99E-03 | 6.14E-03 | 385569 | 4.19E-01 | 505.99 |
| rs12514537 | 5 | 55339462 | T | C | 0.44 | 8.58E-02 | 7.20E-03 | 45875 | 9.63E-33 | 0.45 | -4.81E-03 | 5.23E-03 | 369098 | 3.62E-01 | 142.01 |
| rs13170520 | 5 | 55273842 | T | C | 0.82 | -4.24E-01 | 1.12E-02 | 45875 | 1.00E-300 | 0.88 | -9.95E-03 | 7.93E-03 | 386423 | 2.20E-01 | 1430.46 |
| rs13179290 | 5 | 55302841 | T | C | 0.13 | 4.00E-01 | 1.05E-02 | 45875 | 1.00E-300 | 0.13 | 3.00E-03 | 7.47E-03 | 386003 | 7.10E-01 | 1449.07 |
| rs13182872 | 5 | 55228754 | T | C | 0.82 | -4.12E-01 | 1.11E-02 | 45875 | 1.00E-300 | 0.88 | -1.19E-02 | 7.91E-03 | 385637 | 1.27E-01 | 1379.02 |
| rs13183319 | 5 | 55208557 | A | G | 0.26 | -6.30E-02 | 8.20E-03 | 45876 | 1.16E-14 | 0.26 | 1.09E-02 | 5.80E-03 | 384947 | 6.77E-02 | 59.03 |
| rs13354596 | 5 | 55213858 | T | G | 0.74 | -8.23E-02 | 8.70E-03 | 45876 | 2.73E-21 | 0.79 | -3.00E-03 | 6.26E-03 | 380178 | 6.64E-01 | 89.49 |
| rs1373998 | 5 | 55255565 | A | G | 0.13 | 4.00E-01 | 1.04E-02 | 45875 | 1.00E-300 | 0.13 | 3.00E-03 | 7.46E-03 | 386419 | 7.26E-01 | 1476.33 |
| rs149027249 | 5 | 55227430 | A | G | 0.07 | -7.99E-02 | 1.38E-02 | 45875 | 6.56E-09 | 0.07 | -1.19E-02 | 1.02E-02 | 381960 | 2.43E-01 | 33.52 |
| rs161649 | 5 | 55190030 | T | C | 0.47 | 4.95E-02 | 7.20E-03 | 45877 | 4.83E-12 | 0.50 | 4.71E-03 | 5.13E-03 | 381606 | 3.58E-01 | 47.27 |
| rs166233 | 5 | 55186121 | T | C | 0.47 | 4.99E-02 | 7.20E-03 | 45877 | 3.31E-12 | 0.50 | 4.91E-03 | 5.13E-03 | 381578 | 3.42E-01 | 48.03 |
| rs2112979 | 5 | 55292036 | A | G | 0.68 | 7.81E-02 | 7.90E-03 | 45875 | 6.99E-23 | 0.72 | -4.99E-03 | 5.65E-03 | 386203 | 4.16E-01 | 97.73 |
| rs324995 | 5 | 55225328 | T | G | 0.49 | 4.36E-02 | 7.20E-03 | 45875 | 1.17E-09 | 0.44 | 2.00E-03 | 5.21E-03 | 375170 | 6.51E-01 | 36.67 |
| rs34954805 | 5 | 55212356 | A | G | 0.26 | -6.24E-02 | 8.20E-03 | 45876 | 2.19E-14 | 0.26 | 9.95E-03 | 5.84E-03 | 382723 | 9.30E-02 | 57.91 |
| rs62361958 | 5 | 55215989 | A | G | 0.14 | 1.59E-01 | 1.02E-02 | 45876 | 6.41E-55 | 0.13 | 1.29E-02 | 7.55E-03 | 376854 | 7.89E-02 | 241.77 |
| rs62363861 | 5 | 55223755 | A | G | 0.08 | 3.94E-01 | 1.31E-02 | 45875 | 5.96E-198 | 0.06 | 9.95E-03 | 1.05E-02 | 385397 | 3.58E-01 | 903.21 |
| rs62363863 | 5 | 55226939 | T | C | 0.10 | 3.94E-01 | 1.21E-02 | 45875 | 3.90E-233 | 0.07 | 8.96E-03 | 1.01E-02 | 385824 | 3.50E-01 | 1057.59 |
| rs62363895 | 5 | 55254536 | A | G | 0.81 | -3.95E-01 | 1.04E-02 | 45875 | 1.00E-300 | 0.86 | -3.00E-03 | 7.42E-03 | 386356 | 7.33E-01 | 1444.00 |
| rs6450357 | 5 | 55269996 | T | C | 0.82 | -4.23E-01 | 1.08E-02 | 45875 | 1.00E-300 | 0.88 | -8.96E-03 | 7.84E-03 | 386442 | 2.38E-01 | 1535.48 |
| rs6450361 | 5 | 55300526 | T | C | 0.13 | 4.00E-01 | 1.05E-02 | 45875 | 1.00E-300 | 0.13 | 3.00E-03 | 7.47E-03 | 386055 | 6.81E-01 | 1449.07 |
| rs6861772 | 5 | 55271621 | A | G | 0.81 | -3.99E-01 | 1.04E-02 | 45875 | 1.00E-300 | 0.87 | -3.00E-03 | 7.46E-03 | 386533 | 7.07E-01 | 1472.64 |
| rs6863337 | 5 | 55289146 | T | G | 0.81 | -4.00E-01 | 1.05E-02 | 45875 | 1.00E-300 | 0.87 | -3.00E-03 | 7.46E-03 | 386350 | 7.31E-01 | 1451.25 |
| rs6873542 | 5 | 55282618 | T | C | 0.13 | 4.00E-01 | 1.04E-02 | 45875 | 1.00E-300 | 0.13 | 3.00E-03 | 7.46E-03 | 386415 | 7.10E-01 | 1477.81 |
| rs6891628 | 5 | 55296538 | C | G | 0.13 | 4.00E-01 | 1.04E-02 | 45875 | 1.00E-300 | 0.13 | 3.00E-03 | 7.45E-03 | 386026 | 6.74E-01 | 1475.59 |
| rs7713750 | 5 | 55306073 | A | G | 0.13 | 3.99E-01 | 1.05E-02 | 45875 | 1.00E-300 | 0.13 | 3.00E-03 | 7.47E-03 | 385979 | 7.06E-01 | 1442.55 |
| rs7714146 | 5 | 55217758 | T | C | 0.08 | -7.60E-02 | 1.32E-02 | 45876 | 8.93E-09 | 0.08 | -2.64E-02 | 9.42E-03 | 383674 | 4.92E-03 | 33.15 |
| rs7726239 | 5 | 55295306 | A | C | 0.12 | 4.23E-01 | 1.08E-02 | 45875 | 1.00E-300 | 0.12 | 8.96E-03 | 7.84E-03 | 386032 | 2.32E-01 | 1533.30 |
| rs7730934 | 5 | 55266512 | A | G | 0.12 | 4.23E-01 | 1.07E-02 | 45875 | 1.00E-300 | 0.12 | 7.97E-03 | 7.83E-03 | 386423 | 2.85E-01 | 1559.88 |
| rs77847765 | 5 | 55220580 | A | G | 0.16 | 1.34E-01 | 9.60E-03 | 45876 | 4.44E-44 | 0.14 | -8.94E-03 | 7.56E-03 | 370970 | 2.38E-01 | 193.67 |
| rs78443884 | 5 | 55221681 | C | G | 0.07 | 3.93E-01 | 1.40E-02 | 45876 | 7.59E-174 | 0.06 | 9.95E-03 | 1.07E-02 | 386533 | 3.60E-01 | 786.00 |
| rs78842467 | 5 | 55323891 | A | G | 0.74 | -1.95E-01 | 8.70E-03 | 45875 | 6.22E-112 | 0.78 | -4.99E-03 | 6.13E-03 | 386036 | 4.15E-01 | 500.83 |
| rs9292108 | 5 | 55328578 | A | G | 0.55 | -1.03E-01 | 7.20E-03 | 45875 | 1.99E-46 | 0.53 | 6.62E-03 | 5.16E-03 | 376616 | 2.01E-01 | 203.46 |
| rs9632389 | 5 | 55204187 | T | G | 0.76 | -8.30E-02 | 9.20E-03 | 45877 | 1.56E-19 | 0.83 | -1.19E-02 | 6.76E-03 | 385178 | 7.28E-02 | 81.39 |

Instrumental cis SNPs (50K) select for CRP to Sleep classical Mendelian randomization analysis. CHR: chromosome; POS: hg19 genomic position; A1: effective allele; A2: the other allele; A1_exp_frq: A1 frequency in the exposure dataset; Beta_exp, effect of A1 for the exposure; SE_exp; standard error for Beta_exp; N_exp: sample size for exposure; P_exp, association p value for exposure; A1_out_frq: A1 frequency in the outcome dataset; Beta_out: effect size of A1 on outcome; SE_out: standard error of Beta_out; N_out: sample size for outcome; P_out: association p value for outcome; F-stats: F-statistics.

# **Table S21. Harmonized instrumental cisSNPs (p<5x10^-8^) for sgp130 to Sleepiness MR.**

| SNP | CHR | POS | A1 | A2 | A1_exp_frq | Beta_exp | SE_exp | N_exp | P_exp | A1_out_frq | Beta_out | SE_out | N_out | P_out | Fstats |
| --- | --- | --- | --- | --- | --- | --- | --- | --- | --- | --- | --- | --- | --- | --- | --- |
| rs10041573 | 5 | 55316640 | C | G | 0.81 | -3.78E-01 | 1.06E-02 | 45875 | 1.39E-276 | 0.86 | 8.62E-04 | 1.50E-03 | 452071 | 5.50E-01 | 1270.32 |
| rs10043068 | 5 | 55201091 | A | G | 0.18 | 8.37E-02 | 9.20E-03 | 45876 | 9.03E-20 | 0.17 | -1.65E-04 | 1.37E-03 | 452071 | 9.10E-01 | 82.77 |
| rs10045084 | 5 | 55333925 | A | G | 0.42 | 7.71E-02 | 7.40E-03 | 45875 | 2.96E-25 | 0.66 | -1.87E-03 | 1.08E-03 | 452071 | 7.80E-02 | 108.55 |
| rs10056283 | 5 | 55330467 | T | C | 0.45 | 1.03E-01 | 7.20E-03 | 45875 | 2.16E-46 | 0.47 | -2.59E-04 | 1.03E-03 | 452071 | 7.70E-01 | 203.06 |
| rs10075152 | 5 | 55284949 | T | C | 0.28 | -7.86E-02 | 7.90E-03 | 45875 | 3.81E-23 | 0.28 | -6.76E-04 | 1.14E-03 | 452071 | 5.60E-01 | 98.99 |
| rs10076283 | 5 | 55290446 | C | G | 0.81 | -4.00E-01 | 1.04E-02 | 45875 | 1.00E-300 | 0.87 | 6.27E-04 | 1.51E-03 | 452071 | 6.60E-01 | 1477.07 |
| rs10214033 | 5 | 55327306 | A | G | 0.43 | 9.48E-02 | 7.20E-03 | 45875 | 1.68E-39 | 0.45 | -3.94E-04 | 1.04E-03 | 452071 | 6.70E-01 | 173.36 |
| rs10471419 | 5 | 55251223 | T | C | 0.81 | -3.95E-01 | 1.04E-02 | 45875 | 1.00E-300 | 0.86 | 5.19E-04 | 1.50E-03 | 452071 | 7.10E-01 | 1444.73 |
| rs10805486 | 5 | 55183662 | A | G | 0.47 | 5.00E-02 | 7.20E-03 | 45877 | 2.85E-12 | 0.50 | 4.35E-04 | 1.03E-03 | 452071 | 7.00E-01 | 48.23 |
| rs10940492 | 5 | 55235425 | T | C | 0.82 | -4.16E-01 | 1.12E-02 | 45876 | 1.00E-300 | 0.88 | 1.32E-03 | 1.60E-03 | 452071 | 3.90E-01 | 1381.58 |
| rs1144496 | 5 | 55210985 | T | C | 0.45 | -4.75E-02 | 7.20E-03 | 45876 | 5.04E-11 | 0.56 | 1.51E-03 | 1.03E-03 | 452071 | 1.50E-01 | 43.52 |
| rs11574762 | 5 | 55293271 | T | C | 0.85 | 7.83E-02 | 1.32E-02 | 45875 | 3.01E-09 | 0.93 | 5.74E-04 | 2.00E-03 | 452071 | 7.90E-01 | 35.19 |
| rs11574765 | 5 | 55278967 | A | G | 0.82 | -4.24E-01 | 1.12E-02 | 45875 | 1.00E-300 | 0.88 | 1.15E-03 | 1.60E-03 | 452071 | 4.50E-01 | 1431.81 |
| rs11574769 | 5 | 55258221 | T | C | 0.82 | -4.23E-01 | 1.12E-02 | 45875 | 1.00E-300 | 0.88 | 1.15E-03 | 1.60E-03 | 452071 | 4.50E-01 | 1426.41 |
| rs11574770 | 5 | 55252616 | A | T | 0.82 | -4.22E-01 | 1.12E-02 | 45875 | 1.00E-300 | 0.88 | 1.15E-03 | 1.60E-03 | 452071 | 4.50E-01 | 1418.33 |
| rs11574777 | 5 | 55248943 | A | C | 0.11 | 4.22E-01 | 1.12E-02 | 45875 | 1.00E-300 | 0.12 | -1.18E-03 | 1.60E-03 | 452071 | 4.40E-01 | 1419.67 |
| rs11739048 | 5 | 55263495 | T | C | 0.13 | 4.00E-01 | 1.04E-02 | 45875 | 1.00E-300 | 0.13 | -6.17E-04 | 1.51E-03 | 452071 | 6.60E-01 | 1476.33 |
| rs11740906 | 5 | 55319195 | A | G | 0.12 | 3.78E-01 | 1.06E-02 | 45875 | 3.21E-276 | 0.14 | -8.51E-04 | 1.51E-03 | 452071 | 5.60E-01 | 1271.66 |
| rs11741161 | 5 | 55314248 | C | G | 0.12 | 4.00E-01 | 1.10E-02 | 45875 | 1.50E-290 | 0.12 | -1.29E-03 | 1.58E-03 | 452071 | 3.90E-01 | 1322.98 |
| rs11741953 | 5 | 55267814 | T | C | 0.82 | -4.23E-01 | 1.08E-02 | 45875 | 1.00E-300 | 0.88 | 1.08E-03 | 1.59E-03 | 452071 | 4.70E-01 | 1534.75 |
| rs11742754 | 5 | 55298493 | T | C | 0.83 | -4.16E-01 | 1.15E-02 | 45875 | 1.29E-284 | 0.89 | 5.33E-04 | 1.65E-03 | 452071 | 7.10E-01 | 1309.81 |
| rs11747625 | 5 | 55276273 | T | G | 0.11 | 4.23E-01 | 1.12E-02 | 45875 | 1.00E-300 | 0.12 | -1.16E-03 | 1.60E-03 | 452071 | 4.50E-01 | 1429.11 |
| rs11953360 | 5 | 55324944 | A | C | 0.74 | -1.96E-01 | 8.70E-03 | 45875 | 1.04E-112 | 0.78 | 5.17E-04 | 1.24E-03 | 452071 | 6.40E-01 | 505.99 |
| rs12514537 | 5 | 55339462 | T | C | 0.44 | 8.58E-02 | 7.20E-03 | 45875 | 9.63E-33 | 0.45 | -4.37E-04 | 1.04E-03 | 452071 | 6.30E-01 | 142.01 |
| rs13162054 | 5 | 55320369 | A | T | 0.81 | -3.31E-01 | 1.05E-02 | 45875 | 9.46E-219 | 0.87 | 2.04E-03 | 1.57E-03 | 452071 | 1.80E-01 | 994.35 |
| rs13166487 | 5 | 55303951 | A | T | 0.82 | -4.22E-01 | 1.12E-02 | 45875 | 1.00E-300 | 0.88 | 1.14E-03 | 1.61E-03 | 452071 | 4.50E-01 | 1418.33 |
| rs13170520 | 5 | 55273842 | T | C | 0.82 | -4.24E-01 | 1.12E-02 | 45875 | 1.00E-300 | 0.88 | 1.18E-03 | 1.60E-03 | 452071 | 4.40E-01 | 1430.46 |
| rs13179290 | 5 | 55302841 | T | C | 0.13 | 4.00E-01 | 1.05E-02 | 45875 | 1.00E-300 | 0.13 | -6.00E-04 | 1.51E-03 | 452071 | 6.70E-01 | 1449.07 |
| rs13182872 | 5 | 55228754 | T | C | 0.82 | -4.12E-01 | 1.11E-02 | 45875 | 1.00E-300 | 0.88 | 1.03E-03 | 1.60E-03 | 452071 | 4.90E-01 | 1379.02 |
| rs13183319 | 5 | 55208557 | A | G | 0.26 | -6.30E-02 | 8.20E-03 | 45876 | 1.16E-14 | 0.26 | -2.10E-04 | 1.17E-03 | 452071 | 8.60E-01 | 59.03 |
| rs13354596 | 5 | 55213858 | T | G | 0.74 | -8.23E-02 | 8.70E-03 | 45876 | 2.73E-21 | 0.79 | 1.15E-03 | 1.26E-03 | 452071 | 3.60E-01 | 89.49 |
| rs1373998 | 5 | 55255565 | A | G | 0.13 | 4.00E-01 | 1.04E-02 | 45875 | 1.00E-300 | 0.13 | -5.92E-04 | 1.51E-03 | 452071 | 6.70E-01 | 1476.33 |
| rs149027249 | 5 | 55227430 | A | G | 0.07 | -7.99E-02 | 1.38E-02 | 45875 | 6.56E-09 | 0.07 | -1.74E-03 | 2.04E-03 | 452071 | 4.00E-01 | 33.52 |
| rs161649 | 5 | 55190030 | T | C | 0.47 | 4.95E-02 | 7.20E-03 | 45877 | 4.83E-12 | 0.50 | 4.28E-04 | 1.03E-03 | 452071 | 7.10E-01 | 47.27 |
| rs166233 | 5 | 55186121 | T | C | 0.47 | 4.99E-02 | 7.20E-03 | 45877 | 3.31E-12 | 0.50 | 3.89E-04 | 1.03E-03 | 452071 | 7.40E-01 | 48.03 |
| rs1900173 | 5 | 55240006 | A | T | 0.85 | -3.95E-01 | 1.19E-02 | 45876 | 8.01E-240 | 0.93 | 2.41E-04 | 2.00E-03 | 452071 | 8.80E-01 | 1100.12 |
| rs2112979 | 5 | 55292036 | A | G | 0.68 | 7.81E-02 | 7.90E-03 | 45875 | 6.99E-23 | 0.72 | 7.45E-04 | 1.14E-03 | 452071 | 5.10E-01 | 97.73 |
| rs254987 | 5 | 55193329 | A | G | 0.54 | -6.34E-02 | 7.20E-03 | 45876 | 1.38E-18 | 0.52 | -1.93E-03 | 1.05E-03 | 452071 | 6.80E-02 | 77.54 |
| rs324995 | 5 | 55225328 | T | G | 0.49 | 4.36E-02 | 7.20E-03 | 45875 | 1.17E-09 | 0.44 | -1.34E-03 | 1.04E-03 | 452071 | 2.00E-01 | 36.67 |
| rs34954805 | 5 | 55212356 | A | G | 0.26 | -6.24E-02 | 8.20E-03 | 45876 | 2.19E-14 | 0.26 | -3.69E-04 | 1.18E-03 | 452071 | 7.60E-01 | 57.91 |
| rs62361958 | 5 | 55215989 | A | G | 0.14 | 1.59E-01 | 1.02E-02 | 45876 | 6.41E-55 | 0.14 | 3.58E-04 | 1.50E-03 | 452071 | 8.00E-01 | 241.77 |
| rs62363861 | 5 | 55223755 | A | G | 0.08 | 3.94E-01 | 1.31E-02 | 45875 | 5.96E-198 | 0.06 | -1.19E-03 | 2.13E-03 | 452071 | 5.60E-01 | 903.21 |
| rs62363863 | 5 | 55226939 | T | C | 0.10 | 3.94E-01 | 1.21E-02 | 45875 | 3.90E-233 | 0.07 | -5.24E-04 | 2.04E-03 | 452071 | 7.70E-01 | 1057.59 |
| rs62363895 | 5 | 55254536 | A | G | 0.81 | -3.95E-01 | 1.04E-02 | 45875 | 1.00E-300 | 0.86 | 4.89E-04 | 1.50E-03 | 452071 | 7.30E-01 | 1444.00 |
| rs6450357 | 5 | 55269996 | T | C | 0.82 | -4.23E-01 | 1.08E-02 | 45875 | 1.00E-300 | 0.88 | 1.08E-03 | 1.59E-03 | 452071 | 4.70E-01 | 1535.48 |
| rs6450361 | 5 | 55300526 | T | C | 0.13 | 4.00E-01 | 1.05E-02 | 45875 | 1.00E-300 | 0.13 | -6.45E-04 | 1.51E-03 | 452071 | 6.50E-01 | 1449.07 |
| rs6861772 | 5 | 55271621 | A | G | 0.81 | -3.99E-01 | 1.04E-02 | 45875 | 1.00E-300 | 0.87 | 5.59E-04 | 1.51E-03 | 452071 | 6.90E-01 | 1472.64 |
| rs6863337 | 5 | 55289146 | T | G | 0.81 | -4.00E-01 | 1.05E-02 | 45875 | 1.00E-300 | 0.87 | 6.24E-04 | 1.51E-03 | 452071 | 6.60E-01 | 1451.25 |
| rs6873542 | 5 | 55282618 | T | C | 0.13 | 4.00E-01 | 1.04E-02 | 45875 | 1.00E-300 | 0.13 | -5.97E-04 | 1.51E-03 | 452071 | 6.70E-01 | 1477.81 |
| rs6891628 | 5 | 55296538 | C | G | 0.13 | 4.00E-01 | 1.04E-02 | 45875 | 1.00E-300 | 0.13 | -5.69E-04 | 1.51E-03 | 452071 | 6.90E-01 | 1475.59 |
| rs7712207 | 5 | 55317646 | C | G | 0.80 | -3.82E-01 | 1.03E-02 | 45875 | 1.00E-300 | 0.86 | 6.88E-04 | 1.49E-03 | 452071 | 6.30E-01 | 1373.31 |
| rs7713750 | 5 | 55306073 | A | G | 0.13 | 3.99E-01 | 1.05E-02 | 45875 | 1.00E-300 | 0.13 | -6.01E-04 | 1.51E-03 | 452071 | 6.70E-01 | 1442.55 |
| rs7714146 | 5 | 55217758 | T | C | 0.08 | -7.60E-02 | 1.32E-02 | 45876 | 8.93E-09 | 0.08 | -3.26E-03 | 1.88E-03 | 452071 | 8.50E-02 | 33.15 |
| rs7719246 | 5 | 55244777 | A | T | 0.81 | -3.99E-01 | 1.04E-02 | 45875 | 1.00E-300 | 0.87 | 5.96E-04 | 1.51E-03 | 452071 | 6.70E-01 | 1470.43 |
| rs7720416 | 5 | 55322567 | A | T | 0.64 | -4.42E-01 | 2.01E-02 | 10514 | 5.96E-107 | 0.87 | 1.82E-03 | 1.54E-03 | 452071 | 2.20E-01 | 483.78 |
| rs7726239 | 5 | 55295306 | A | C | 0.12 | 4.23E-01 | 1.08E-02 | 45875 | 1.00E-300 | 0.12 | -1.01E-03 | 1.59E-03 | 452071 | 5.00E-01 | 1533.30 |
| rs7728232 | 5 | 55220344 | A | G | 0.35 | 8.63E-02 | 7.50E-03 | 45876 | 1.09E-30 | 0.34 | 4.98E-04 | 1.11E-03 | 452071 | 7.00E-01 | 132.40 |
| rs7730934 | 5 | 55266512 | A | G | 0.12 | 4.23E-01 | 1.07E-02 | 45875 | 1.00E-300 | 0.12 | -9.50E-04 | 1.58E-03 | 452071 | 5.20E-01 | 1559.88 |
| rs7736703 | 5 | 55312262 | A | T | 0.12 | 3.78E-01 | 1.06E-02 | 45875 | 1.02E-276 | 0.14 | -8.93E-04 | 1.50E-03 | 452071 | 5.40E-01 | 1274.36 |
| rs77847765 | 5 | 55220580 | A | G | 0.16 | 1.34E-01 | 9.60E-03 | 45876 | 4.44E-44 | 0.14 | -2.18E-03 | 1.49E-03 | 452071 | 1.40E-01 | 193.67 |
| rs78443884 | 5 | 55221681 | C | G | 0.07 | 3.93E-01 | 1.40E-02 | 45876 | 7.59E-174 | 0.06 | -2.02E-03 | 2.17E-03 | 452071 | 3.40E-01 | 786.00 |
| rs78842467 | 5 | 55323891 | A | G | 0.74 | -1.95E-01 | 8.70E-03 | 45875 | 6.22E-112 | 0.78 | 3.64E-04 | 1.24E-03 | 452071 | 7.30E-01 | 500.83 |
| rs9292108 | 5 | 55328578 | A | G | 0.55 | -1.03E-01 | 7.20E-03 | 45875 | 1.99E-46 | 0.53 | 2.43E-04 | 1.03E-03 | 452071 | 7.80E-01 | 203.46 |
| rs9632389 | 5 | 55204187 | T | G | 0.76 | -8.30E-02 | 9.20E-03 | 45877 | 1.56E-19 | 0.83 | 2.26E-04 | 1.36E-03 | 452071 | 8.80E-01 | 81.39 |

Instrumental cis SNPs (50K) select for CRP to Sleep classical Mendelian randomization analysis. CHR: chromosome; POS: hg19 genomic position; A1: effective allele; A2: the other allele; A1_exp_frq: A1 frequency in the exposure dataset; Beta_exp, effect of A1 for the exposure; SE_exp; standard error for Beta_exp; N_exp: sample size for exposure; P_exp, association p value for exposure; A1_out_frq: A1 frequency in the outcome dataset; Beta_out: effect size of A1 on outcome; SE_out: standard error of Beta_out; N_out: sample size for outcome; P_out: association p value for outcome; F-stats: F-statistics.

# **Table S22. Harmonized instrumental cisSNPs (p<5x10^-8^) for CRP in iPsych to Insomnia MR.**

| SNP | CHR | POS | A1 | A2 | A1_exp_frq | Beta_exp | SE_exp | N_exp | P_exp | A1_out_frq | Beta_out | SE_out | N_out | P_out | Fstats |
| --- | --- | --- | --- | --- | --- | --- | --- | --- | --- | --- | --- | --- | --- | --- | --- |
| rs11265257 | 1 | 159668984 | T | C | 0.39 | -2.16E-01 | 1.82E-02 | 8318 | 3.62E-32 | 0.39 | 2.00E-03 | 5.24E-03 | 384026 | 7.46E-01 | 141.37 |
| rs11265264 | 1 | 159712842 | A | G | 0.36 | 2.70E-01 | 1.85E-02 | 8318 | 1.35E-47 | 0.35 | -3.21E-03 | 5.35E-03 | 382611 | 5.47E-01 | 213.00 |
| rs1130864 | 1 | 159683091 | A | G | 0.32 | 3.33E-01 | 1.89E-02 | 8318 | 9.41E-68 | 0.31 | -4.81E-03 | 5.53E-03 | 385606 | 3.86E-01 | 309.50 |
| rs11588887 | 1 | 159717162 | A | G | 0.16 | -1.77E-01 | 2.50E-02 | 8318 | 1.78E-12 | 0.16 | -1.30E-03 | 7.05E-03 | 381493 | 8.52E-01 | 50.01 |
| rs12093699 | 1 | 159647988 | A | G | 0.32 | 3.03E-01 | 1.88E-02 | 8318 | 1.69E-57 | 0.31 | -7.02E-03 | 5.53E-03 | 386533 | 2.07E-01 | 259.93 |
| rs12094103 | 1 | 159723619 | A | G | 0.36 | 2.46E-01 | 1.85E-02 | 8318 | 7.20E-40 | 0.35 | -2.50E-03 | 5.36E-03 | 383822 | 6.37E-01 | 176.24 |
| rs12727021 | 1 | 159702487 | A | G | 0.32 | 3.31E-01 | 1.89E-02 | 8318 | 2.93E-67 | 0.31 | -4.11E-03 | 5.53E-03 | 386233 | 4.60E-01 | 306.16 |
| rs12739022 | 1 | 159659431 | T | C | 0.68 | -3.28E-01 | 1.90E-02 | 8318 | 7.59E-66 | 0.69 | 4.91E-03 | 5.57E-03 | 380707 | 3.82E-01 | 298.56 |
| rs12754745 | 1 | 159670483 | A | G | 0.32 | 3.30E-01 | 1.90E-02 | 8318 | 1.19E-66 | 0.31 | -5.11E-03 | 5.56E-03 | 381743 | 3.59E-01 | 302.03 |
| rs12754915 | 1 | 159660869 | T | C | 0.68 | -3.28E-01 | 1.90E-02 | 8318 | 7.18E-66 | 0.69 | 5.11E-03 | 5.57E-03 | 380709 | 3.60E-01 | 298.56 |
| rs12759988 | 1 | 159708751 | A | G | 0.35 | 2.72E-01 | 1.87E-02 | 8318 | 3.36E-47 | 0.33 | -2.00E-03 | 5.43E-03 | 384276 | 7.10E-01 | 210.79 |
| rs12760041 | 1 | 159714035 | T | C | 0.35 | 2.70E-01 | 1.87E-02 | 8318 | 1.03E-46 | 0.33 | -1.20E-03 | 5.42E-03 | 384393 | 8.31E-01 | 208.16 |
| rs137918962 | 1 | 159652311 | A | G | 0.05 | 2.92E-01 | 4.37E-02 | 8318 | 2.60E-11 | 0.05 | 4.99E-03 | 1.18E-02 | 383741 | 7.02E-01 | 44.56 |
| rs1470515 | 1 | 159653599 | T | C | 0.39 | -2.13E-01 | 1.83E-02 | 8318 | 4.19E-31 | 0.39 | 2.00E-03 | 5.25E-03 | 381417 | 6.93E-01 | 135.60 |
| rs16842484 | 1 | 159646924 | T | C | 0.72 | -2.94E-01 | 2.02E-02 | 8318 | 4.37E-47 | 0.75 | 9.14E-03 | 5.91E-03 | 378816 | 1.21E-01 | 211.26 |
| rs1811471 | 1 | 159642599 | A | G | 0.28 | 2.89E-01 | 2.03E-02 | 8318 | 1.60E-45 | 0.25 | -9.75E-03 | 5.94E-03 | 375626 | 1.02E-01 | 202.82 |
| rs1900394 | 1 | 159632498 | T | G | 0.64 | -2.14E-01 | 1.89E-02 | 8318 | 1.20E-29 | 0.65 | 5.52E-03 | 5.34E-03 | 385746 | 3.01E-01 | 128.68 |
| rs2211320 | 1 | 159693605 | A | G | 0.33 | -2.34E-01 | 1.88E-02 | 8318 | 4.97E-35 | 0.33 | 2.00E-03 | 5.40E-03 | 386389 | 6.59E-01 | 154.53 |
| rs2794498 | 1 | 159636116 | T | G | 0.27 | 2.26E-01 | 2.04E-02 | 8318 | 2.29E-28 | 0.25 | -6.82E-03 | 5.90E-03 | 385571 | 2.46E-01 | 122.73 |
| rs2794500 | 1 | 159635021 | T | C | 0.36 | 2.15E-01 | 1.89E-02 | 8318 | 1.25E-29 | 0.35 | -4.81E-03 | 5.34E-03 | 386358 | 3.68E-01 | 128.80 |
| rs2808629 | 1 | 159676796 | A | G | 0.34 | -2.36E-01 | 1.88E-02 | 8318 | 8.22E-36 | 0.33 | 3.00E-03 | 5.40E-03 | 386094 | 5.25E-01 | 157.58 |
| rs3093059 | 1 | 159685136 | A | G | 0.95 | -3.18E-01 | 4.15E-02 | 8318 | 2.26E-14 | 0.94 | -7.97E-03 | 1.10E-02 | 386533 | 4.53E-01 | 58.61 |
| rs3116636 | 1 | 159686483 | A | G | 0.32 | 3.33E-01 | 1.89E-02 | 8318 | 9.63E-68 | 0.31 | -4.11E-03 | 5.54E-03 | 385255 | 4.57E-01 | 309.50 |
| rs3116651 | 1 | 159698485 | T | C | 0.32 | 3.31E-01 | 1.89E-02 | 8318 | 2.54E-67 | 0.31 | -3.91E-03 | 5.52E-03 | 386481 | 4.76E-01 | 306.16 |
| rs3116653 | 1 | 159696910 | C | G | 0.32 | 3.33E-01 | 1.89E-02 | 8318 | 3.41E-68 | 0.31 | -4.01E-03 | 5.52E-03 | 386451 | 4.73E-01 | 309.87 |
| rs3116656 | 1 | 159692372 | A | G | 0.68 | -3.31E-01 | 1.89E-02 | 8318 | 2.56E-67 | 0.69 | 4.01E-03 | 5.52E-03 | 386533 | 4.65E-01 | 306.16 |
| rs3122012 | 1 | 159689323 | T | C | 0.68 | -3.30E-01 | 1.89E-02 | 8318 | 3.73E-67 | 0.69 | 4.01E-03 | 5.53E-03 | 385834 | 4.70E-01 | 305.42 |
| rs35198997 | 1 | 159710368 | T | C | 0.74 | -3.18E-01 | 2.06E-02 | 8318 | 4.85E-53 | 0.75 | 7.73E-03 | 5.97E-03 | 374058 | 1.95E-01 | 238.90 |
| rs4131568 | 1 | 159722056 | T | C | 0.36 | 2.54E-01 | 1.86E-02 | 8318 | 3.22E-42 | 0.34 | -1.00E-03 | 5.39E-03 | 386533 | 8.48E-01 | 187.07 |
| rs4255379 | 1 | 159718312 | A | G | 0.61 | 2.17E-01 | 1.84E-02 | 8318 | 1.00E-31 | 0.61 | -3.99E-03 | 5.24E-03 | 383610 | 4.09E-01 | 138.83 |
| rs4261114 | 1 | 159700989 | A | C | 0.68 | -3.31E-01 | 1.89E-02 | 8318 | 2.73E-67 | 0.69 | 3.81E-03 | 5.54E-03 | 385709 | 4.97E-01 | 306.34 |
| rs4420078 | 1 | 159720716 | A | G | 0.39 | -2.16E-01 | 1.84E-02 | 8318 | 1.94E-31 | 0.39 | 3.99E-03 | 5.23E-03 | 384549 | 4.36E-01 | 137.30 |
| rs4512645 | 1 | 159729047 | A | G | 0.36 | 2.52E-01 | 1.86E-02 | 8318 | 2.31E-41 | 0.34 | -1.60E-03 | 5.40E-03 | 385080 | 7.68E-01 | 183.41 |
| rs7551731 | 1 | 159694779 | T | C | 0.67 | 2.33E-01 | 1.88E-02 | 8318 | 8.61E-35 | 0.67 | -2.00E-03 | 5.40E-03 | 386389 | 6.60E-01 | 153.07 |
| rs77289344 | 1 | 159691075 | T | G | 0.95 | -3.23E-01 | 4.17E-02 | 8318 | 1.11E-14 | 0.94 | -6.98E-03 | 1.11E-02 | 386292 | 5.50E-01 | 59.92 |
| rs876537 | 1 | 159674933 | T | C | 0.39 | -2.18E-01 | 1.82E-02 | 8318 | 6.62E-33 | 0.39 | 1.00E-03 | 5.22E-03 | 386533 | 7.79E-01 | 143.87 |
| rs895581 | 1 | 159637987 | A | G | 0.73 | -2.25E-01 | 2.04E-02 | 8318 | 3.91E-28 | 0.75 | 7.02E-03 | 5.90E-03 | 385461 | 2.36E-01 | 121.76 |

Instrumental cis SNPs (50K) select for CRP to Sleep classical Mendelian randomization analysis. CHR: chromosome; POS: hg19 genomic position; A1: effective allele; A2: the other allele; A1_exp_frq: A1 frequency in the exposure dataset; Beta_exp, effect of A1 for the exposure; SE_exp; standard error for Beta_exp; N_exp: sample size for exposure; P_exp, association p value for exposure; A1_out_frq: A1 frequency in the outcome dataset; Beta_out: effect size of A1 on outcome; SE_out: standard error of Beta_out; N_out: sample size for outcome; P_out: association p value for outcome; F-stats: F-statistics.

# **Table S23. Harmonized instrumental cisSNPs (p<5x10^-8^) for CRP in iPsych to Sleepiness MR.**

| SNP | CHR | POS | A1 | A2 | A1_exp_frq | Beta_exp | SE_exp | N_exp | P_exp | A1_out_frq | Beta_out | SE_out | N_out | P_out | Fstats |
| --- | --- | --- | --- | --- | --- | --- | --- | --- | --- | --- | --- | --- | --- | --- | --- |
| rs11265257 | 1 | 159668984 | T | C | 0.39 | -2.16E-01 | 1.82E-02 | 8318 | 3.62E-32 | 0.39 | 1.72E-03 | 1.05E-03 | 452071 | 9.50E-02 | 141.37 |
| rs11265264 | 1 | 159712842 | A | G | 0.36 | 2.70E-01 | 1.85E-02 | 8318 | 1.35E-47 | 0.36 | 3.38E-04 | 1.07E-03 | 452071 | 7.70E-01 | 213.00 |
| rs1130864 | 1 | 159683091 | A | G | 0.32 | 3.33E-01 | 1.89E-02 | 8318 | 9.41E-68 | 0.31 | 3.44E-04 | 1.11E-03 | 452071 | 7.70E-01 | 309.50 |
| rs11588887 | 1 | 159717162 | A | G | 0.16 | -1.77E-01 | 2.50E-02 | 8318 | 1.78E-12 | 0.16 | 9.48E-04 | 1.41E-03 | 452071 | 4.60E-01 | 50.01 |
| rs12093699 | 1 | 159647988 | A | G | 0.32 | 3.03E-01 | 1.88E-02 | 8318 | 1.69E-57 | 0.31 | 6.34E-04 | 1.11E-03 | 452071 | 5.70E-01 | 259.93 |
| rs12094103 | 1 | 159723619 | A | G | 0.36 | 2.46E-01 | 1.85E-02 | 8318 | 7.20E-40 | 0.35 | 4.71E-04 | 1.08E-03 | 452071 | 6.90E-01 | 176.24 |
| rs12726900 | 1 | 159732523 | A | G | 0.37 | 2.48E-01 | 2.00E-02 | 8318 | 5.23E-35 | 0.35 | 5.25E-04 | 1.13E-03 | 452071 | 6.60E-01 | 153.64 |
| rs12727021 | 1 | 159702487 | A | G | 0.32 | 3.31E-01 | 1.89E-02 | 8318 | 2.93E-67 | 0.31 | 4.09E-04 | 1.11E-03 | 452071 | 7.20E-01 | 306.16 |
| rs12734907 | 1 | 159719594 | A | T | 0.64 | -2.56E-01 | 1.86E-02 | 8318 | 9.57E-43 | 0.66 | -4.11E-04 | 1.09E-03 | 452071 | 7.30E-01 | 189.73 |
| rs12739022 | 1 | 159659431 | T | C | 0.68 | -3.28E-01 | 1.90E-02 | 8318 | 7.59E-66 | 0.69 | -1.48E-04 | 1.12E-03 | 452071 | 9.10E-01 | 298.56 |
| rs12754745 | 1 | 159670483 | A | G | 0.32 | 3.30E-01 | 1.90E-02 | 8318 | 1.19E-66 | 0.31 | 2.14E-04 | 1.11E-03 | 452071 | 8.60E-01 | 302.03 |
| rs12754915 | 1 | 159660869 | T | C | 0.68 | -3.28E-01 | 1.90E-02 | 8318 | 7.18E-66 | 0.69 | -2.74E-04 | 1.12E-03 | 452071 | 8.20E-01 | 298.56 |
| rs12759988 | 1 | 159708751 | A | G | 0.35 | 2.72E-01 | 1.87E-02 | 8318 | 3.36E-47 | 0.33 | 2.18E-04 | 1.09E-03 | 452071 | 8.60E-01 | 210.79 |
| rs12760041 | 1 | 159714035 | T | C | 0.35 | 2.70E-01 | 1.87E-02 | 8318 | 1.03E-46 | 0.33 | 1.70E-04 | 1.09E-03 | 452071 | 9.00E-01 | 208.16 |
| rs1470515 | 1 | 159653599 | T | C | 0.39 | -2.13E-01 | 1.83E-02 | 8318 | 4.19E-31 | 0.39 | 1.63E-03 | 1.06E-03 | 452071 | 1.20E-01 | 135.60 |
| rs16842484 | 1 | 159646924 | T | C | 0.72 | -2.94E-01 | 2.02E-02 | 8318 | 4.37E-47 | 0.74 | -1.16E-03 | 1.18E-03 | 452071 | 3.50E-01 | 211.26 |
| rs1811471 | 1 | 159642599 | A | G | 0.28 | 2.89E-01 | 2.03E-02 | 8318 | 1.60E-45 | 0.26 | 1.02E-03 | 1.18E-03 | 452071 | 4.10E-01 | 202.82 |
| rs1900394 | 1 | 159632498 | T | G | 0.64 | -2.14E-01 | 1.89E-02 | 8318 | 1.20E-29 | 0.65 | 3.62E-04 | 1.08E-03 | 452071 | 7.30E-01 | 128.68 |
| rs1935193 | 1 | 159664090 | A | T | 0.71 | 1.10E-01 | 2.00E-02 | 8318 | 3.72E-08 | 0.70 | 2.38E-03 | 1.13E-03 | 452071 | 3.30E-02 | 30.36 |
| rs2211320 | 1 | 159693605 | A | G | 0.33 | -2.34E-01 | 1.88E-02 | 8318 | 4.97E-35 | 0.33 | 1.38E-03 | 1.09E-03 | 452071 | 2.00E-01 | 154.53 |
| rs2592892 | 1 | 159645586 | C | G | 0.26 | -1.60E-01 | 2.15E-02 | 8318 | 1.02E-13 | 0.26 | -3.48E-04 | 1.18E-03 | 452071 | 7.80E-01 | 55.59 |
| rs2592893 | 1 | 159644292 | T | C | 0.74 | 1.61E-01 | 2.15E-02 | 8318 | 8.38E-14 | 0.74 | 4.41E-04 | 1.18E-03 | 452071 | 7.20E-01 | 55.87 |
| rs2794498 | 1 | 159636116 | T | G | 0.27 | 2.26E-01 | 2.04E-02 | 8318 | 2.29E-28 | 0.25 | 3.16E-04 | 1.19E-03 | 452071 | 8.40E-01 | 122.73 |
| rs2794500 | 1 | 159635021 | T | C | 0.36 | 2.15E-01 | 1.89E-02 | 8318 | 1.25E-29 | 0.35 | -3.77E-04 | 1.08E-03 | 452071 | 7.20E-01 | 128.80 |
| rs2808624 | 1 | 159665921 | C | G | 0.61 | 2.14E-01 | 1.83E-02 | 8318 | 2.35E-31 | 0.61 | -1.76E-03 | 1.06E-03 | 452071 | 9.00E-02 | 136.62 |
| rs2808629 | 1 | 159676796 | A | G | 0.34 | -2.36E-01 | 1.88E-02 | 8318 | 8.22E-36 | 0.33 | 1.32E-03 | 1.09E-03 | 452071 | 2.10E-01 | 157.58 |
| rs3091244 | 1 | 159684665 | A | G | 0.32 | 3.33E-01 | 1.89E-02 | 8318 | 7.47E-68 | 0.31 | 5.37E-04 | 1.11E-03 | 452071 | 6.40E-01 | 310.24 |
| rs3093059 | 1 | 159685136 | A | G | 0.95 | -3.18E-01 | 4.15E-02 | 8318 | 2.26E-14 | 0.94 | 4.11E-03 | 2.23E-03 | 452071 | 6.90E-02 | 58.61 |
| rs3116636 | 1 | 159686483 | A | G | 0.32 | 3.33E-01 | 1.89E-02 | 8318 | 9.63E-68 | 0.31 | 3.63E-04 | 1.11E-03 | 452071 | 7.50E-01 | 309.50 |
| rs3116651 | 1 | 159698485 | T | C | 0.32 | 3.31E-01 | 1.89E-02 | 8318 | 2.54E-67 | 0.31 | 3.67E-04 | 1.11E-03 | 452071 | 7.50E-01 | 306.16 |
| rs3116653 | 1 | 159696910 | C | G | 0.32 | 3.33E-01 | 1.89E-02 | 8318 | 3.41E-68 | 0.31 | 3.66E-04 | 1.11E-03 | 452071 | 7.50E-01 | 309.87 |
| rs3116656 | 1 | 159692372 | A | G | 0.68 | -3.31E-01 | 1.89E-02 | 8318 | 2.56E-67 | 0.69 | -4.20E-04 | 1.11E-03 | 452071 | 7.10E-01 | 306.16 |
| rs3122012 | 1 | 159689323 | T | C | 0.68 | -3.30E-01 | 1.89E-02 | 8318 | 3.73E-67 | 0.69 | -3.78E-04 | 1.11E-03 | 452071 | 7.40E-01 | 305.42 |
| rs35198997 | 1 | 159710368 | T | C | 0.74 | -3.18E-01 | 2.06E-02 | 8318 | 4.85E-53 | 0.74 | -5.41E-04 | 1.19E-03 | 452071 | 6.60E-01 | 238.90 |
| rs4131568 | 1 | 159722056 | T | C | 0.36 | 2.54E-01 | 1.86E-02 | 8318 | 3.22E-42 | 0.34 | 4.32E-04 | 1.09E-03 | 452071 | 7.10E-01 | 187.07 |
| rs4255379 | 1 | 159718312 | A | G | 0.61 | 2.17E-01 | 1.84E-02 | 8318 | 1.00E-31 | 0.61 | 2.97E-04 | 1.05E-03 | 452071 | 7.60E-01 | 138.83 |
| rs4261114 | 1 | 159700989 | A | C | 0.68 | -3.31E-01 | 1.89E-02 | 8318 | 2.73E-67 | 0.69 | -5.48E-04 | 1.11E-03 | 452071 | 6.30E-01 | 306.34 |
| rs4420078 | 1 | 159720716 | A | G | 0.39 | -2.16E-01 | 1.84E-02 | 8318 | 1.94E-31 | 0.39 | -3.17E-04 | 1.05E-03 | 452071 | 7.40E-01 | 137.30 |
| rs4512645 | 1 | 159729047 | A | G | 0.36 | 2.52E-01 | 1.86E-02 | 8318 | 2.31E-41 | 0.34 | 4.80E-04 | 1.09E-03 | 452071 | 6.80E-01 | 183.41 |
| rs7551731 | 1 | 159694779 | T | C | 0.67 | 2.33E-01 | 1.88E-02 | 8318 | 8.61E-35 | 0.67 | -1.37E-03 | 1.09E-03 | 452071 | 2.00E-01 | 153.07 |
| rs77289344 | 1 | 159691075 | T | G | 0.95 | -3.23E-01 | 4.17E-02 | 8318 | 1.11E-14 | 0.95 | 4.31E-03 | 2.25E-03 | 452071 | 5.90E-02 | 59.92 |
| rs876537 | 1 | 159674933 | T | C | 0.39 | -2.18E-01 | 1.82E-02 | 8318 | 6.62E-33 | 0.39 | 1.71E-03 | 1.05E-03 | 452071 | 9.70E-02 | 143.87 |
| rs895581 | 1 | 159637987 | A | G | 0.73 | -2.25E-01 | 2.04E-02 | 8318 | 3.91E-28 | 0.75 | -2.75E-04 | 1.19E-03 | 452071 | 8.60E-01 | 121.76 |

Instrumental cis SNPs (50K) select for CRP to Sleep classical Mendelian randomization analysis. CHR: chromosome; POS: hg19 genomic position; A1: effective allele; A2: the other allele; A1_exp_frq: A1 frequency in the exposure dataset; Beta_exp, effect of A1 for the exposure; SE_exp; standard error for Beta_exp; N_exp: sample size for exposure; P_exp, association p value for exposure; A1_out_frq: A1 frequency in the outcome dataset; Beta_out: effect size of A1 on outcome; SE_out: standard error of Beta_out; N_out: sample size for outcome; P_out: association p value for outcome; F-stats: F-statistics.

# **Table S24. Causal effects of inflammatory markers on accelerometry measured sleep duration.**

| Marker | NSNP | Effect | SE | P | Egger_P | PRESSO_P |
| --- | --- | --- | --- | --- | --- | --- |
| CRP | 42 | -1.43e-2 | 5.28e-3 | 6.78e-3 | 0.81 | 1.0 |
| sIL6R | 88 | -6.04e-3 | 8.93e-4 | 0.0 | 0.37 | 0.34 |
| sgp130 | 64 | 1.14e-2 | 2.43e-3 | 2.8e-6 | 0.02 | 1.0 |

NSNP, the number of SNPs used as instrument; Effect, IVW estimates for causal effect; SE, standard errors for the estimated causal effects; P, IVW p values; Egger_P, p values for the test of horizontal pleiotropy; PRESSO_P, MRPRESSOR global test of horizontal pleiotropy.

# **Table S25. Harmonized instrumental cisSNPs (p<5x10^-8^) for CRP to accelerometry measure sleep duration MR.**

| SNP | CHR | POS | A1 | A2 | A1_exp_frq | Beta_exp | SE_exp | N_exp | P_exp | A1_out_frq | Beta_out | SE_out | N_out | P_out | Fstats |
| --- | --- | --- | --- | --- | --- | --- | --- | --- | --- | --- | --- | --- | --- | --- | --- |
| rs10908741 | 1 | 159726123 | T | G | 0.25 | -0.05 | 0.0041 | 204402 | 1.64e-33 | 0.24 | 2.29e-05 | 0.0048 | 85449 | 0.95 | 145.678 |
| rs10908742 | 1 | 159732811 | G | T | 0.24 | -0.06 | 0.00506 | 204402 | 3.50e-30 | 0.24 | -6.28e-4 | 0.0050 | 85449 | 0.86 | 130.47 |
| rs11265257 | 1 | 159668984 | T | C | 0.389 | -0.17 | 0.0036 | 204402 | 1,00E-300 | 0.39 | 3.04e-3 | 0.0042 | 85449 | 0.51 | 2150.79 |
| rs11265260 | 1 | 159700039 | G | A | 0.06 | 0.22 | 0.0073 | 204402 | 2.12e-202 | 0.06 | -2.36e-3 | 0.0087 | 85449 | 0.77 | 921.756 |
| rs11265263 | 1 | 159710517 | A | C | 0.08 | -0.27 | 0.0080 | 204402 | 7.52e-245 | 0.07 | 9.01e-3 | 0.0080 | 85449 | 0.27 | 1117.07 |
| rs11588887 | 1 | 159717162 | A | G | 0.14 | -0.20 | 0.0058 | 204402 | 5.79e-271 | 0.16 | 4.05e-3 | 0.0056 | 85449 | 0.5 | 1237.07 |
| rs11589667 | 1 | 159641029 | T | C | 0.33 | -0.05 | 0.0047 | 204402 | 5.18e-22 | 0.34 | 1.83e-3 | 0.0043 | 85449 | 0.64 | 93.123 |
| rs11811420 | 1 | 159719872 | C | G | 0.36 | 0.10 | 0.0037 | 204402 | 6.49e-162 | 0.35 | -1.79e-4 | 0.0043 | 85449 | 0.98 | 735.50 |
| rs12029953 | 1 | 159727739 | A | C | 0.25 | -0.05 | 0.0044 | 204402 | 5.18e-34 | 0.24 | -2.02e-4 | 0.0048 | 85449 | 0.91 | 147.98 |
| rs12049404 | 1 | 159713844 | T | C | 0.16 | -0.17 | 0.0050 | 204402 | 2.55e-270 | 0.16 | 4.18e-3 | 0.0056 | 85449 | 0.49 | 1234.47 |
| rs1205 | 1 | 159682233 | T | C | 0.33 | -0.18 | 0.0037 | 204402 | 1,00E-300 | 0.33 | 3.84e-3 | 0.0043 | 85449 | 0.41 | 2400.50 |
| rs12081252 | 1 | 159706513 | C | T | 0.06 | 0.22 | 0.0076 | 204402 | 5.40e-190 | 0.06 | -5.63e-4 | 0.0088 | 85449 | 0.93 | 864.74 |
| rs12093699 | 1 | 159647988 | A | G | 0.31 | 0.13 | 0.0038 | 204402 | 5.13e-243 | 0.31 | -8.31e-5 | 0.0044 | 85449 | 0.98 | 1108.82 |
| rs12094103 | 1 | 159723619 | A | G | 0.36 | 0.10 | 0.0037 | 204402 | 5.19e-159 | 0.35 | -6.34e-4 | 0.0043 | 85449 | 0.94 | 722.3 |
| rs12567054 | 1 | 159644968 | G | T | 0.07 | 0.15 | 0.0083 | 204402 | 1.08e-77 | 0.07 | 9.06e-3 | 0.0083 | 85449 | 0.26 | 348.39 |
| rs12727021 | 1 | 159702487 | A | G | 0.31 | 0.14 | 0.0038 | 204402 | 1.09e-294 | 0.31 | -2.01e-4 | 0.0044 | 85449 | 0.95 | 1346.52 |
| rs12739022 | 1 | 159659431 | C | T | 0.32 | 0.14 | 0.0040 | 204402 | 3.72e-278 | 0.31 | -2.29e-3 | 0.0044 | 85449 | 0.69 | 1270.61 |
| rs12754915 | 1 | 159660869 | C | T | 0.31 | 0.14 | 0.0039 | 204402 | 1.00e-300 | 0.31 | -2.52e-3 | 0.0044 | 85449 | 0.65 | 1377.5 |
| rs12755606 | 1 | 159670336 | G | C | 0.33 | 0.15 | 0.0040 | 204402 | 1.89e-296 | 0.31 | -2.19e-4 | 0.0044 | 85449 | 0.7 | 1354.33 |
| rs1341665 | 1 | 159691559 | A | G | 0.33 | -0.18 | 0.0038 | 204402 | 1.00e-300 | 0.34 | 2.03e-3 | 0.0043 | 85449 | 0.68 | 2221.42 |
| rs1417938 | 1 | 159684186 | A | T | 0.31 | 0.14 | 0.0038 | 204402 | 1.00e-300 | 0.31 | -3.97e-4 | 0.0044 | 85449 | 0.98 | 1374.35 |
| rs1470515 | 1 | 159653599 | T | C | 0.39 | -0.17 | 0.0037 | 204402 | 1.00e-300 | 0.39 | 2.43e-3 | 0.0042 | 85449 | 0.6 | 2033.302 |
| rs1572970 | 1 | 159673585 | A | G | 0.70 | -0.04 | 0.0040 | 204402 | 2.06e-28 | 0.70 | 4.39e-4 | 0.0044 | 85449 | 0.88 | 122.39 |
| rs16842484 | 1 | 159646924 | C | T | 0.26 | 0.12 | 0.0043 | 204402 | 6.84e-181 | 0.26 | -2.60e-3 | 0.0047 | 85449 | 0.63 | 822.83 |
| rs16842599 | 1 | 159697475 | C | T | 0.06 | 0.22 | 0.0075 | 204402 | 2.82e-192 | 0.06 | -6.55e-4 | 0.0088 | 85449 | 0.92 | 875.17 |
| rs1811472 | 1 | 159642349 | C | G | 0.41 | -0.12 | 0.0039 | 204402 | 2.11e-213 | 0.40 | 7.59e-4 | 0.0042 | 85449 | 0.87 | 972.20 |
| rs1935193 | 1 | 159664090 | A | T | 0.70 | -0.05 | 0.0039 | 204402 | 7.87e-32 | 0.70 | 3.84e-4 | 0.0045 | 85449 | 0.89 | 138.04 |
| rs1971863 | 1 | 159638931 | C | T | 0.26 | 0.09 | 0.0041 | 204402 | 7.94e-105 | 0.25 | -3.45e-3 | 0.0047 | 85449 | 0.5 | 473.01 |
| rs2027469 | 1 | 159667190 | A | G | 0.18 | -0.08 | 0.0049 | 204402 | 3.21e-66 | 0.19 | 5.48e-4 | 0.0052 | 85449 | 0.91 | 295.73 |
| rs2027471 | 1 | 159689388 | A | T | 0.34 | -0.18 | 0.0039 | 204402 | 1.00e-300 | 0.34 | 2.48e-3 | 0.0043 | 85449 | 0.61 | 2168.011 |
| rs2794498 | 1 | 159636116 | T | G | 0.26 | 0.09 | 0.0040 | 204402 | 8.34e-103 | 0.25 | -3.53e-3 | 0.0047 | 85449 | 0.49 | 463.87 |
| rs2794500 | 1 | 159635021 | T | C | 0.36 | 0.07 | 0.0037 | 204402 | 9.98e-81 | 0.35 | 5.09e-4 | 0.0043 | 85449 | 0.87 | 362.33 |
| rs2794520 | 1 | 159678816 | T | C | 0.33 | -0.18 | 0.0037 | 204402 | 1.00e-300 | 0.33 | 3.57e-3 | 0.0043 | 85449 | 0.44 | 2408.87 |
| rs2808624 | 1 | 159665921 | G | C | 0.39 | -0.17 | 0.0037 | 204402 | 1.00e-300 | 0.39 | 3.06e-3 | 0.0042 | 85449 | 0.5 | 2130.45 |
| rs2808628 | 1 | 159676011 | A | G | 0.33 | -0.18 | 0.0039 | 204402 | 1.00e-300 | 0.33 | 3.45e-3 | 0.0043 | 85449 | 0.46 | 2238.23 |
| rs3093075 | 1 | 159679913 | T | G | 0.06 | 0.23 | 0.0074 | 204402 | 2.02e-204 | 0.06 | 7.72e-3 | 0.0088 | 85449 | 0.95 | 931.16 |
| rs4131568 | 1 | 159722056 | T | C | 0.36 | 0.10 | 0.0038 | 204402 | 4.89e-142 | 0.34 | -7.89e-4 | 0.0043 | 85449 | 0.92 | 644.29 |
| rs4255379 | 1 | 159718312 | A | G | 0.63 | 0.06 | 0.0038 | 204402 | 1.35e-62 | 0.61 | -8.33e-4 | 0.0042 | 85449 | 0.84 | 279.045 |
| rs4512645 | 1 | 159729047 | A | G | 0.35 | 0.10 | 0.0039 | 204402 | 8.22e-142 | 0.34 | -1.53e-3 | 0.0043 | 85449 | 0.79 | 643.156 |
| rs7553007 | 1 | 159698549 | A | G | 0.32 | -0.18 | 0.0039 | 204402 | 1.00e-300 | 0.33 | 3.22e-3 | 0.0043 | 85449 | 0.49 | 2220.29 |
| rs876537 | 1 | 159674933 | T | C | 0.38 | -0.17 | 0.0037 | 204402 | 1.00e-300 | 0.39 | 2.97e-3 | 0.0042 | 85449 | 0.52 | 2137.45 |
| rs895582 | 1 | 159637919 | A | G | 0.26 | 0.09 | 0.0041 | 204402 | 3.79e-107 | 0.25 | -3.56e-3 | 0.0047 | 85449 | 0.48 | 483.62 |

Instrumental cis SNPs (50K) select for CRP to accelerometry measured sleep duration by classical Mendelian randomization analysis. CHR: chromosome; POS: hg19 genomic position; A1: effective allele; A2: the other allele; A1_exp_frq: A1 frequency in the exposure dataset; Beta_exp, effect of A1 for the exposure; SE_exp; standard error for Beta_exp; N_exp: sample size for exposure; P_exp, association p value for exposure; A1_out_frq: A1 frequency in the outcome dataset; Beta_out: effect size of A1 on outcome; SE_out: standard error of Beta_out; N_out: sample size for outcome; P_out: association p value for outcome; F-stats: F-statistics.

# **Table S26. Harmonized instrumental cisSNPs (p<5x10^-8^) for sgp130 to accelerometry measure sleep duration MR.**

| SNP | CHR | POS | A1 | A2 | A1_exp_frq | Beta_exp | SE_exp | N_exp | P_exp | Beta_out | A1_out_frq | SE_out | N_out | P_out | Fstats |
| --- | --- | --- | --- | --- | --- | --- | --- | --- | --- | --- | --- | --- | --- | --- | --- |
| rs10041573 | 5 | 55316640 | C | G | 0.8097 | -0.3778 | 0.0106 | 45875 | 1.39e-276 | -0.00382104 | 0.869 | 0.0059 | 85449 | 0.55 | 1270.32 |
| rs10043068 | 5 | 55201091 | A | G | 0.1841 | 0.0837 | 0.0092 | 45876 | 9.025e-20 | 0.0013006 | 0.17 | 0.0054 | 85449 | 0.83 | 82.77 |
| rs10045084 | 5 | 55333925 | A | G | 0.4244 | 0.0771 | 0.0074 | 45875 | 2.957e-25 | -0.00157557 | 0.66 | 0.0043 | 85449 | 0.74 | 108.55 |
| rs10056283 | 5 | 55330467 | T | C | 0.4496 | 0.1026 | 0.0072 | 45875 | 2.158e-46 | -0.00317072 | 0.47 | 0.0041 | 85449 | 0.46 | 203.06 |
| rs10075152 | 5 | 55284949 | T | C | 0.2824 | -0.0786 | 0.0079 | 45875 | 3.81e-23 | 0.000288879 | 0.28 | 0.0045 | 85449 | 0.93 | 98.99 |
| rs10076283 | 5 | 55290446 | C | G | 0.8093 | -0.3997 | 0.0104 | 45875 | 1,00E-300 | -0.00617476 | 0.87 | 0.0060 | 85449 | 0.31 | 1477.07 |
| rs10214033 | 5 | 55327306 | A | G | 0.4324 | 0.0948 | 0.0072 | 45875 | 1.675e-39 | -0.0048181 | 0.46 | 0.0041 | 85449 | 0.26 | 173.36 |
| rs10471419 | 5 | 55251223 | T | C | 0.8075 | -0.3953 | 0.0104 | 45875 | 1,00E-300 | -0.00567095 | 0.86 | 0.0059 | 85449 | 0.35 | 1444.739 |
| rs10805486 | 5 | 55183662 | A | G | 0.4704 | 0.05 | 0.0072 | 45877 | 2.849e-12 | -4.20647e-05 | 0.50 | 0.0041 | 85449 | 0.99 | 48.23 |
| rs10940492 | 5 | 55235425 | T | C | 0.8222 | -0.4163 | 0.0112 | 45876 | 1,00E-300 | -0.00472615 | 0.88 | 0.0063 | 85449 | 0.48 | 1381.585 |
| rs1144496 | 5 | 55210985 | T | C | 0.4462 | -0.0475 | 0.0072 | 45876 | 5.037e-11 | 0.000157313 | 0.56 | 0.0041 | 85449 | 0.96 | 43.52 |
| rs11574762 | 5 | 55293271 | T | C | 0.8504 | 0.0783 | 0.0132 | 45875 | 3.009e-09 | -0.0218267 | 0.93 | 0.0079 | 85449 | 0.006 | 35.19 |
| rs11574765 | 5 | 55278967 | A | G | 0.8223 | -0.4238 | 0.0112 | 45875 | 1,00E-300 | -0.00457025 | 0.88 | 0.0063 | 85449 | 0.49 | 1431.81 |
| rs11574769 | 5 | 55258221 | T | C | 0.8217 | -0.423 | 0.0112 | 45875 | 1,00E-300 | -0.00456317 | 0.88 | 0.0063 | 85449 | 0.49 | 1426.41 |
| rs11574770 | 5 | 55252616 | A | T | 0.8219 | -0.4218 | 0.0112 | 45875 | 1,00E-300 | -0.00459603 | 0.88 | 0.0063 | 85449 | 0.49 | 1418.33 |
| rs11574777 | 5 | 55248943 | A | C | 0.1104 | 0.422 | 0.0112 | 45875 | 1,00E-300 | 0.00443077 | 0.12 | 0.0063 | 85449 | 0.51 | 1419.67 |
| rs11739048 | 5 | 55263495 | T | C | 0.1314 | 0.3996 | 0.0104 | 45875 | 1,00E-300 | 0.00622965 | 0.13 | 0.0060 | 85449 | 0.31 | 1476.339 |
| rs11740906 | 5 | 55319195 | A | G | 0.1248 | 0.378 | 0.0106 | 45875 | 3.21e-276 | 0.00341295 | 0.14 | 0.0060 | 85449 | 0.59 | 1271.66 |
| rs11741161 | 5 | 55314248 | C | G | 0.1152 | 0.4001 | 0.011 | 45875 | 1.5e-290 | 0.00315738 | 0.12 | 0.0062 | 85449 | 0.65 | 1322.98 |
| rs11741953 | 5 | 55267814 | T | C | 0.8151 | -0.4231 | 0.0108 | 45875 | 1,00E-300 | -0.005917 | 0.88 | 0.0063 | 85449 | 0.36 | 1534.75 |
| rs11742754 | 5 | 55298493 | T | C | 0.829 | -0.4162 | 0.0115 | 45875 | 1.29e-284 | -0.00791356 | 0.89 | 0.0065 | 85449 | 0.24 | 1309.81 |
| rs11747625 | 5 | 55276273 | T | G | 0.1104 | 0.4234 | 0.0112 | 45875 | 1,00E-300 | 0.0046921 | 0.12 | 0.0063 | 85449 | 0.48 | 1429.11 |
| rs11953360 | 5 | 55324944 | A | C | 0.7425 | -0.1957 | 0.0087 | 45875 | 1.04e-112 | 0.000366325 | 0.78 | 0.0049 | 85449 | 0.96 | 505.99 |
| rs12514537 | 5 | 55339462 | T | C | 0.4357 | 0.0858 | 0.0072 | 45875 | 9.627e-33 | -0.00372343 | 0.45 | 0.0041 | 85449 | 0.39 | 142.01 |
| rs13162054 | 5 | 55320369 | A | T | 0.8099 | -0.3311 | 0.0105 | 45875 | 9.46e-219 | -0.00646938 | 0.87 | 0.0062 | 85449 | 0.3 | 994.35 |
| rs13166487 | 5 | 55303951 | A | T | 0.8236 | -0.4218 | 0.0112 | 45875 | 1,00E-300 | -0.00460873 | 0.88 | 0.0063 | 85449 | 0.49 | 1418.33 |
| rs13170520 | 5 | 55273842 | T | C | 0.8227 | -0.4236 | 0.0112 | 45875 | 1,00E-300 | -0.00463248 | 0.88 | 0.0063 | 85449 | 0.49 | 1430.46 |
| rs13179290 | 5 | 55302841 | T | C | 0.1312 | 0.3997 | 0.0105 | 45875 | 1,00E-300 | 0.0062583 | 0.13 | 0.0060 | 85449 | 0.31 | 1449.07 |
| rs13182872 | 5 | 55228754 | T | C | 0.823 | -0.4122 | 0.0111 | 45875 | 1,00E-300 | -0.00454959 | 0.88 | 0.0063 | 85449 | 0.49 | 1379.02 |
| rs13183319 | 5 | 55208557 | A | G | 0.2627 | -0.063 | 0.0082 | 45876 | 1.16e-14 | -0.000174086 | 0.26 | 0.0046 | 85449 | 0.96 | 59.03 |
| rs13354596 | 5 | 55213858 | T | G | 0.7406 | -0.0823 | 0.0087 | 45876 | 2.729e-21 | 0.00062757 | 0.79 | 0.0050 | 85449 | 0.9 | 89.49 |
| rs1373998 | 5 | 55255565 | A | G | 0.1315 | 0.3996 | 0.0104 | 45875 | 1,00E-300 | 0.00615749 | 0.13 | 0.0060 | 85449 | 0.31 | 1476.33 |
| rs149027249 | 5 | 55227430 | A | G | 0.0724 | -0.0799 | 0.0138 | 45875 | 6.564e-09 | 0.0203467 | 0.07 | 0.0081 | 85449 | 0.012 | 33.52 |
| rs161649 | 5 | 55190030 | T | C | 0.4705 | 0.0495 | 0.0072 | 45877 | 4.825e-12 | -0.000244879 | 0.50 | 0.0041 | 85449 | 0.97 | 47.27 |
| rs166233 | 5 | 55186121 | T | C | 0.4704 | 0.0499 | 0.0072 | 45877 | 3.309e-12 | -3.06154e-06 | 0.50 | 0.0041 | 85449 | 0.98 | 48.03 |
| rs1900173 | 5 | 55240006 | A | T | 0.8469 | -0.3947 | 0.0119 | 45876 | 8.01e-240 | -0.00419251 | 0.93 | 0.0079 | 85449 | 0.62 | 1100.12 |
| rs2112979 | 5 | 55292036 | A | G | 0.6824 | 0.0781 | 0.0079 | 45875 | 6.986e-23 | -0.00070136 | 0.72 | 0.0045 | 85449 | 0.86 | 97.734 |
| rs254987 | 5 | 55193329 | A | G | 0.5398 | -0.0634 | 0.0072 | 45876 | 1.383e-18 | 0.00194727 | 0.52 | 0.0041 | 85449 | 0.64 | 77.54 |
| rs324995 | 5 | 55225328 | T | G | 0.4937 | 0.0436 | 0.0072 | 45875 | 1.17e-09 | 0.00461412 | 0.44 | 0.0041 | 85449 | 0.26 | 36.67 |
| rs34954805 | 5 | 55212356 | A | G | 0.2607 | -0.0624 | 0.0082 | 45876 | 2.193e-14 | 0.00012715 | 0.26 | 0.0047 | 85449 | 0.99 | 57.91 |
| rs62361958 | 5 | 55215989 | A | G | 0.1436 | 0.1586 | 0.0102 | 45876 | 6.409e-55 | 0.000771861 | 0.14 | 0.0060 | 85449 | 0.93 | 241.77 |
| rs62363861 | 5 | 55223755 | A | G | 0.0805 | 0.3937 | 0.0131 | 45875 | 5.96e-198 | -0.00066003 | 0.06 | 0.0084 | 85449 | 0.9 | 903.21 |
| rs62363863 | 5 | 55226939 | T | C | 0.0964 | 0.3935 | 0.0121 | 45875 | 3.9e-233 | 0.00175674 | 0.07 | 0.0081 | 85449 | 0.86 | 1057.59 |
| rs62363895 | 5 | 55254536 | A | G | 0.8069 | -0.3952 | 0.0104 | 45875 | 1,00E-300 | -0.00556465 | 0.86 | 0.0059 | 85449 | 0.36 | 1444.00 |
| rs6450357 | 5 | 55269996 | T | C | 0.8157 | -0.4232 | 0.0108 | 45875 | 1,00E-300 | -0.00592058 | 0.88 | 0.0063 | 85449 | 0.36 | 1535.48 |
| rs6450361 | 5 | 55300526 | T | C | 0.1311 | 0.3997 | 0.0105 | 45875 | 1,00E-300 | 0.0057757 | 0.13 | 0.0060 | 85449 | 0.35 | 1449.07 |
| rs6861772 | 5 | 55271621 | A | G | 0.8084 | -0.3991 | 0.0104 | 45875 | 1,00E-300 | -0.00637442 | 0.87 | 0.0060 | 85449 | 0.3 | 1472.64 |
| rs6863337 | 5 | 55289146 | T | G | 0.8098 | -0.4 | 0.0105 | 45875 | 1,00E-300 | -0.00620163 | 0.87 | 0.0060 | 85449 | 0.31 | 1451.25 |
| rs6873542 | 5 | 55282618 | T | C | 0.1314 | 0.3998 | 0.0104 | 45875 | 1,00E-300 | 0.00620844 | 0.13 | 0.0060 | 85449 | 0.31 | 1477.81 |
| rs6891628 | 5 | 55296538 | C | G | 0.1317 | 0.3995 | 0.0104 | 45875 | 1,00E-300 | 0.00608704 | 0.13 | 0.0060 | 85449 | 0.32 | 1475.596 |
| rs7712207 | 5 | 55317646 | C | G | 0.8049 | -0.3817 | 0.0103 | 45875 | 1,00E-300 | -0.00481945 | 0.86 | 0.0059 | 85449 | 0.44 | 1373.312 |
| rs7713750 | 5 | 55306073 | A | G | 0.1312 | 0.3988 | 0.0105 | 45875 | 1,00E-300 | 0.00625107 | 0.13 | 0.0060 | 85449 | 0.31 | 1442.55 |
| rs7714146 | 5 | 55217758 | T | C | 0.0772 | -0.076 | 0.0132 | 45876 | 8.925e-09 | 0.00899909 | 0.08 | 0.0075 | 85449 | 0.22 | 33.15 |
| rs7719246 | 5 | 55244777 | A | T | 0.8087 | -0.3988 | 0.0104 | 45875 | 1,00E-300 | -0.00616721 | 0.87 | 0.0060 | 85449 | 0.31 | 1470.43 |
| rs7720416 | 5 | 55322567 | A | T | 0.6404 | -0.4421 | 0.0201 | 10514 | 5.96e-107 | -0.00771604 | 0.87 | 0.0061 | 85449 | 0.21 | 483.78 |
| rs7726239 | 5 | 55295306 | A | C | 0.1217 | 0.4229 | 0.0108 | 45875 | 1,00E-300 | 0.005805 | 0.12 | 0.0063 | 85449 | 0.37 | 1533.308 |
| rs7728232 | 5 | 55220344 | A | G | 0.3477 | 0.0863 | 0.0075 | 45876 | 1.09e-30 | 0.000185296 | 0.34 | 0.0044 | 85449 | 0.96 | 132.40 |
| rs7730934 | 5 | 55266512 | A | G | 0.122 | 0.4226 | 0.0107 | 45875 | 1,00E-300 | 0.00613385 | 0.12 | 0.0063 | 85449 | 0.34 | 1559.89 |
| rs7736703 | 5 | 55312262 | A | T | 0.1248 | 0.3784 | 0.0106 | 45875 | 1.02e-276 | 0.0040693 | 0.14 | 0.0060 | 85449 | 0.52 | 1274.36 |
| rs77847765 | 5 | 55220580 | A | G | 0.1637 | 0.1336 | 0.0096 | 45876 | 4.436e-44 | 0.00975671 | 0.15 | 0.0059 | 85449 | 0.1 | 193.671 |
| rs78443884 | 5 | 55221681 | C | G | 0.0686 | 0.3925 | 0.014 | 45876 | 7.59e-174 | -0.000821861 | 0.06 | 0.0086 | 85449 | 0.9 | 786.00 |
| rs78842467 | 5 | 55323891 | A | G | 0.7424 | -0.1947 | 0.0087 | 45875 | 6.22e-112 | 0.000768781 | 0.78 | 0.0049 | 85449 | 0.89 | 500.83 |
| rs9292108 | 5 | 55328578 | A | G | 0.5456 | -0.1027 | 0.0072 | 45875 | 1.987e-46 | 0.00322354 | 0.53 | 0.0041 | 85449 | 0.45 | 203.46 |
| rs9632389 | 5 | 55204187 | T | G | 0.7614 | -0.083 | 0.0092 | 45877 | 1.558e-19 | -0.0015414 | 0.83 | 0.0054 | 85449 | 0.8 | 81.39 |

Instrumental cis SNPs (50K) select for sgp130 to accelerometry measured sleep duration by classical Mendelian randomization analysis. CHR: chromosome; POS: hg19 genomic position; A1: effective allele; A2: the other allele; A1_exp_frq: A1 frequency in the exposure dataset; Beta_exp, effect of A1 for the exposure; SE_exp; standard error for Beta_exp; N_exp: sample size for exposure; P_exp, association p value for exposure; A1_out_frq: A1 frequency in the outcome dataset; Beta_out: effect size of A1 on outcome; SE_out: standard error of Beta_out; N_out: sample size for outcome; P_out: association p value for outcome; F-stats: F-statistics.

# **Table S27. Harmonized instrumental cisSNPs (p<5x10^-8^) for sIL6R to accelerometry measure sleep duration MR.**

| SNP | CHR | POS | A1 | A2 | A1_exp_frq | Beta_exp | SE_exp | N_exp | P_exp | A1_out_frq | Beta_out | Se_out | P_out | N_out |
| --- | --- | --- | --- | --- | --- | --- | --- | --- | --- | --- | --- | --- | --- | --- |
| rs10047079 | 1 | 154468135 | T | C | 0.7659 | 0.5875 | 0.0054 | 69970 | 1,00E-300 | 0.807069 | -0.0108499 | 0.00515232 | 0.039 | 85449 |
| rs1073907 | 1 | 154332520 | T | C | 0.2969 | 0.1531 | 0.0081 | 35570.9 | 3.201e-79 | 0.30135 | 9.88148e-05 | 0.00442614 | 0.99 | 85449 |
| rs10752641 | 1 | 154432042 | C | G | 0.2735 | 0.6437 | 0.0058 | 49179 | 1,00E-300 | 0.224371 | 0.0106534 | 0.00486732 | 0.031 | 85449 |
| rs10908839 | 1 | 154430798 | C | G | 0.5781 | -0.6109 | 0.0052 | 70935 | 1,00E-300 | 0.783901 | -0.0120313 | 0.00493102 | 0.017 | 85449 |
| rs10908840 | 1 | 154459477 | T | C | 0.4391 | 0.1209 | 0.0057 | 70929.9 | 2.23e-101 | 0.484379 | -0.00776645 | 0.00406235 | 0.053 | 85449 |
| rs111885536 | 1 | 154461260 | A | G | 0.1918 | -0.2112 | 0.007 | 70935 | 1.26e-202 | 0.164366 | -0.00080772 | 0.00548155 | 0.94 | 85449 |
| rs11265606 | 1 | 154356459 | T | C | 0.2791 | 0.1802 | 0.0062 | 70934.9 | 4.84e-186 | 0.291928 | 0.000595833 | 0.00448002 | 0.9 | 85449 |
| rs11265607 | 1 | 154357678 | A | G | 0.68 | -0.1828 | 0.0062 | 70934.9 | 3.48e-191 | 0.708947 | -0.000664123 | 0.00447944 | 0.89 | 85449 |
| rs11265621 | 1 | 154442960 | A | G | 0.4629 | 0.5407 | 0.0043 | 70934.9 | 1,00E-300 | 0.642778 | -0.00732331 | 0.00424457 | 0.08 | 85449 |
| rs11265622 | 1 | 154451420 | A | G | 0.5253 | -0.5367 | 0.0043 | 69970 | 1,00E-300 | 0.357891 | 0.00696528 | 0.00423983 | 0.096 | 85449 |
| rs113624284 | 1 | 154339299 | A | G | 0.7869 | -0.2597 | 0.0078 | 70935 | 1.49e-242 | 0.85222 | 0.00668919 | 0.00571218 | 0.22 | 85449 |
| rs11576181 | 1 | 154330659 | T | G | 0.6697 | -0.1228 | 0.0061 | 70934.9 | 1.7e-89 | 0.69847 | -0.000264182 | 0.00442625 | 0.96 | 85449 |
| rs11580178 | 1 | 154353262 | A | T | 0.2856 | 0.121 | 0.0076 | 49179 | 3.035e-57 | 0.304958 | 0.000431589 | 0.00441052 | 0.93 | 85449 |
| rs11580535 | 1 | 154364317 | T | G | 0.1325 | 0.3547 | 0.008 | 70935 | 1,00E-300 | 0.136746 | -0.00436188 | 0.00591665 | 0.43 | 85449 |
| rs11582433 | 1 | 154349605 | T | C | 0.1434 | 0.2704 | 0.0077 | 70935 | 4.44e-269 | 0.150795 | -0.00463081 | 0.00566112 | 0.38 | 85449 |
| rs11590203 | 1 | 154333569 | T | G | 0.1393 | 0.2501 | 0.0079 | 69970 | 2.44e-222 | 0.146425 | -0.00599293 | 0.00572972 | 0.27 | 85449 |
| rs116088025 | 1 | 154354350 | T | C | 0.1428 | 0.2733 | 0.0077 | 70935 | 3.37e-273 | 0.150804 | -0.0044964 | 0.00566408 | 0.39 | 85449 |
| rs12023772 | 1 | 154483868 | A | G | 0.1728 | 0.7118 | 0.0055 | 70934.9 | 1,00E-300 | 0.157712 | 0.00265655 | 0.00555953 | 0.63 | 85449 |
| rs12025518 | 1 | 154340789 | A | C | 0.6887 | 0.1918 | 0.0063 | 69969 | 1,00E-200 | 0.737454 | 0.00269912 | 0.00461136 | 0.54 | 85449 |
| rs12033701 | 1 | 154365886 | T | C | 0.1077 | -0.166 | 0.0087 | 70934 | 1.231e-81 | 0.105006 | -0.00100286 | 0.00661385 | 0.88 | 85449 |
| rs12044132 | 1 | 154462360 | T | C | 0.1548 | 0.6847 | 0.0096 | 34606 | 1,00E-300 | 0.157394 | 0.0027941 | 0.0055603 | 0.62 | 85449 |
| rs12061599 | 1 | 154344135 | T | G | 0.284 | 0.1293 | 0.0062 | 70935 | 5.38e-98 | 0.300356 | -0.000348261 | 0.00442868 | 0.93 | 85449 |
| rs12075836 | 1 | 154371487 | T | C | 0.1436 | -0.2621 | 0.0077 | 70934.9 | 8.28e-252 | 0.147622 | -0.00486747 | 0.00574748 | 0.37 | 85449 |
| rs12118018 | 1 | 154477440 | A | G | 0.463 | 0.5363 | 0.0043 | 70934.9 | 1,00E-300 | 0.641935 | -0.00680682 | 0.00423869 | 0.1 | 85449 |
| rs12118721 | 1 | 154397416 | T | C | 0.5499 | -0.8085 | 0.0039 | 70933.9 | 1,00E-300 | 0.427989 | 0.00292234 | 0.00414812 | 0.52 | 85449 |
| rs12119111 | 1 | 154478600 | A | G | 0.4634 | 0.5367 | 0.0043 | 70934.9 | 1,00E-300 | 0.642242 | -0.00671069 | 0.00423907 | 0.11 | 85449 |
| rs12129500 | 1 | 154423764 | T | C | 0.5552 | -0.8429 | 0.0039 | 70934.9 | 1,00E-300 | 0.420936 | 0.00522815 | 0.00411431 | 0.22 | 85449 |
| rs12133641 | 1 | 154428283 | A | G | 0.5717 | -1.0109 | 0.0034 | 70935 | 1,00E-300 | 0.591614 | 0.00373075 | 0.00412111 | 0.35 | 85449 |
| rs12563459 | 1 | 154362686 | A | G | 0.1518 | -0.1853 | 0.0076 | 70934.9 | 1.95e-130 | 0.153674 | -0.0022424 | 0.00563579 | 0.67 | 85449 |
| rs12568083 | 1 | 154455949 | T | C | 0.3873 | -0.5016 | 0.0072 | 34606 | 1,00E-300 | 0.357943 | 0.00692493 | 0.0042391 | 0.097 | 85449 |
| rs12739228 | 1 | 154426190 | A | G | 0.0366 | -0.6317 | 0.0159 | 68557 | 1,00E-300 | 0.0386030000000001 | -0.00097767 | 0.0106932 | 0.9 | 85449 |
| rs12753254 | 1 | 154416935 | A | G | 0.4076 | 0.9759 | 0.0035 | 70934.9 | 1,00E-300 | 0.416782 | -0.00283967 | 0.00412191 | 0.5 | 85449 |
| rs12753666 | 1 | 154474875 | A | G | 0.4629 | 0.536 | 0.0043 | 70934.9 | 1,00E-300 | 0.64195 | -0.00680197 | 0.00423884 | 0.1 | 85449 |
| rs138765671 | 1 | 154345962 | A | C | 0.1382 | 0.2614 | 0.0078 | 70935 | 2.75e-243 | 0.146015 | -0.00625276 | 0.00573428 | 0.25 | 85449 |
| rs147763778 | 1 | 154368223 | A | G | 0.0383 | 0.3994 | 0.016 | 68557 | 3.27e-138 | 0.041362 | -0.0074001 | 0.0105603 | 0.47 | 85449 |
| rs1889313 | 1 | 154351717 | A | C | 0.1435 | 0.2704 | 0.0077 | 70935 | 6.23e-269 | 0.150809 | -0.00449809 | 0.00565992 | 0.39 | 85449 |
| rs2229238 | 1 | 154437896 | T | C | 0.5805 | -0.5856 | 0.0055 | 69970 | 1,00E-300 | 0.183684 | 0.0134309 | 0.0052332 | 0.012 | 85449 |
| rs34094138 | 1 | 154366911 | T | G | 0.7767 | 0.1857 | 0.0076 | 70935 | 1.2e-131 | 0.846436 | 0.00218264 | 0.00563994 | 0.68 | 85449 |
| rs35717427 | 1 | 154391882 | A | G | 0.1208 | 0.4985 | 0.0083 | 69968.9 | 1,00E-300 | 0.122127 | 0.00188776 | 0.00619239 | 0.8 | 85449 |
| rs4240872 | 1 | 154436195 | T | C | 0.3921 | 0.6191 | 0.005 | 70935 | 1,00E-300 | 0.775322 | -0.0107057 | 0.00486307 | 0.03 | 85449 |
| rs4379670 | 1 | 154439865 | A | T | 0.2388 | 0.6118 | 0.0064 | 49179 | 1,00E-300 | 0.182235 | 0.0135544 | 0.00525996 | 0.012 | 85449 |
| rs4393147 | 1 | 154414037 | T | C | 0.4104 | 0.9747 | 0.0035 | 70934.9 | 1,00E-300 | 0.416838 | -0.0030352 | 0.00412469 | 0.46 | 85449 |
| rs4474240 | 1 | 154457855 | A | C | 0.4659 | -0.2139 | 0.007 | 69970 | 5.07e-206 | 0.164687 | -0.000925809 | 0.00547721 | 0.92 | 85449 |
| rs4478801 | 1 | 154464572 | A | G | 0.5777 | 0.5011 | 0.0072 | 34597.9 | 1,00E-300 | 0.642041 | -0.00684259 | 0.00423911 | 0.1 | 85449 |
| rs4521987 | 1 | 154388668 | T | C | 0.4451 | -0.2733 | 0.0079 | 69968.9 | 8.69e-265 | 0.147079 | -0.00558382 | 0.00581274 | 0.31 | 85449 |
| rs45478197 | 1 | 154422733 | T | C | 0.1008 | -0.203 | 0.009 | 70935 | 2.6e-113 | 0.08296 | -0.00764096 | 0.00738729 | 0.3 | 85449 |
| rs4553185 | 1 | 154410955 | T | C | 0.4419 | 0.8299 | 0.0039 | 69969.9 | 1,00E-300 | 0.578673 | -0.00335196 | 0.00412519 | 0.45 | 85449 |
| rs4845372 | 1 | 154415396 | A | C | 0.4245 | 0.9399 | 0.0036 | 70934.9 | 1,00E-300 | 0.425613 | -0.00390337 | 0.00410971 | 0.35 | 85449 |
| rs4845374 | 1 | 154426947 | A | T | 0.1978 | -0.2571 | 0.0086 | 49179 | 1.7e-196 | 0.162258 | -0.00131919 | 0.00550163 | 0.87 | 85449 |
| rs4845617 | 1 | 154377898 | A | G | 0.3921 | 0.125 | 0.0057 | 69965.8 | 1.71e-105 | 0.401143 | 0.000883765 | 0.00417328 | 0.82 | 85449 |
| rs4845618 | 1 | 154400015 | T | G | 0.443 | 0.8087 | 0.0039 | 70934 | 1,00E-300 | 0.572292 | -0.00299199 | 0.00413615 | 0.5 | 85449 |
| rs4845637 | 1 | 154490178 | A | G | 0.525 | -0.5358 | 0.0043 | 70934.9 | 1,00E-300 | 0.35798 | 0.00649567 | 0.00423801 | 0.12 | 85449 |
| rs57569414 | 1 | 154380419 | A | C | 0.117 | 0.4789 | 0.0084 | 69969.9 | 1,00E-300 | 0.120013 | 0.00179737 | 0.00629295 | 0.8 | 85449 |
| rs59632925 | 1 | 154406540 | T | G | 0.5566 | -0.8161 | 0.0039 | 70934.9 | 1,00E-300 | 0.412911 | 0.00408707 | 0.0041328 | 0.35 | 85449 |
| rs6427631 | 1 | 154370020 | T | C | 0.6795 | -0.1838 | 0.0062 | 70934.9 | 2.06e-193 | 0.708857 | -0.00079135 | 0.00448795 | 0.86 | 85449 |
| rs6657938 | 1 | 154336126 | A | G | 0.672 | -0.1302 | 0.0061 | 70935 | 4,00E-100 | 0.699378 | 0.000439259 | 0.00442582 | 0.92 | 85449 |
| rs6664039 | 1 | 154337238 | A | G | 0.7872 | -0.2583 | 0.0078 | 70935 | 8.02e-240 | 0.852288 | 0.00657567 | 0.00571243 | 0.23 | 85449 |
| rs6664608 | 1 | 154479670 | T | C | 0.463 | 0.5362 | 0.0043 | 70934.9 | 1,00E-300 | 0.641931 | -0.00682237 | 0.00423833 | 0.1 | 85449 |
| rs66654715 | 1 | 154376820 | C | G | 0.0336 | -0.5542 | 0.0176 | 68557 | 9.7e-218 | 0.032675 | -0.00850348 | 0.0117622 | 0.44 | 85449 |
| rs6667434 | 1 | 154409100 | A | G | 0.5565 | -0.8168 | 0.0039 | 70934.9 | 1,00E-300 | 0.412841 | 0.00411463 | 0.00413498 | 0.35 | 85449 |
| rs6674171 | 1 | 154491683 | A | G | 0.7662 | 0.5875 | 0.0054 | 69970 | 1,00E-300 | 0.807074 | -0.0106441 | 0.00515091 | 0.042 | 85449 |
| rs6675472 | 1 | 154445503 | T | C | 0.5818 | -0.5875 | 0.0053 | 70935 | 1,00E-300 | 0.19171 | 0.0112402 | 0.00516638 | 0.033 | 85449 |
| rs6683206 | 1 | 154418088 | T | C | 0.5584 | -0.8251 | 0.0039 | 70935 | 1,00E-300 | 0.411232 | 0.0042171 | 0.00413986 | 0.34 | 85449 |
| rs6684439 | 1 | 154395839 | T | C | 0.4037 | 0.964 | 0.0038 | 62755.9 | 1,00E-300 | 0.410558 | -0.0024149 | 0.00420162 | 0.57 | 85449 |
| rs6686467 | 1 | 154329095 | A | G | 0.1396 | 0.2517 | 0.0078 | 70935 | 5.53e-228 | 0.146618 | -0.00584233 | 0.00572949 | 0.28 | 85449 |
| rs6686750 | 1 | 154419843 | A | G | 0.5547 | -0.8422 | 0.0039 | 70934.9 | 1,00E-300 | 0.420898 | 0.00520248 | 0.00411552 | 0.22 | 85449 |
| rs6687597 | 1 | 154434936 | A | G | 0.3918 | 0.6195 | 0.005 | 70935 | 1,00E-300 | 0.775386 | -0.0105075 | 0.00486413 | 0.034 | 85449 |
| rs6689965 | 1 | 154470606 | A | T | 0.4214 | 0.5534 | 0.0048 | 49179 | 1,00E-300 | 0.357984 | 0.00681737 | 0.00423949 | 0.1 | 85449 |
| rs6694817 | 1 | 154401972 | T | C | 0.5569 | -0.7974 | 0.004 | 70932.9 | 1,00E-300 | 0.419088 | 0.00388988 | 0.00413654 | 0.38 | 85449 |
| rs66980031 | 1 | 154481153 | C | G | 0.8046 | 0.6472 | 0.02 | 49179 | 5.87e-230 | 0.963068 | 0.00395259 | 0.0109672 | 0.69 | 85449 |
| rs6700296 | 1 | 154473660 | T | C | 0.525 | -0.536 | 0.0043 | 70934.9 | 1,00E-300 | 0.357991 | 0.00682819 | 0.00423886 | 0.1 | 85449 |
| rs72633646 | 1 | 154334683 | A | G | 0.1397 | 0.2532 | 0.0078 | 70935 | 1.75e-230 | 0.146397 | -0.00604321 | 0.00572912 | 0.27 | 85449 |
| rs72633650 | 1 | 154360838 | T | C | 0.7932 | -0.3538 | 0.008 | 70934 | 1,00E-300 | 0.863011 | 0.0046114 | 0.00591062 | 0.4 | 85449 |
| rs72698115 | 1 | 154379369 | A | C | 0.8207 | 0.1787 | 0.0088 | 70934 | 1.14e-92 | 0.894949 | 0.00222127 | 0.00667202 | 0.74 | 85449 |
| rs72698169 | 1 | 154486799 | A | C | 0.7819 | -0.7107 | 0.0055 | 70934.9 | 1,00E-300 | 0.842053 | -0.00261374 | 0.00555689 | 0.64 | 85449 |
| rs73018293 | 1 | 154465577 | T | C | 0.197 | -0.5862 | 0.0053 | 70934.9 | 1,00E-300 | 0.192926 | 0.0108492 | 0.00515247 | 0.039 | 85449 |
| rs73020232 | 1 | 154482669 | T | C | 0.1969 | -0.5863 | 0.0053 | 70934.9 | 1,00E-300 | 0.192971 | 0.0105836 | 0.00515117 | 0.044 | 85449 |
| rs73020246 | 1 | 154485640 | A | G | 0.7657 | 0.5868 | 0.0053 | 70934.9 | 1,00E-300 | 0.807048 | -0.0106411 | 0.0051511 | 0.043 | 85449 |
| rs7513603 | 1 | 154481158 | T | C | 0.4632 | -0.2128 | 0.0069 | 70934.9 | 5.71e-206 | 0.164745 | -0.000973205 | 0.00547481 | 0.91 | 85449 |
| rs7519499 | 1 | 154487926 | A | G | 0.4631 | 0.5363 | 0.0043 | 70934.9 | 1,00E-300 | 0.642279 | -0.00644249 | 0.00423826 | 0.12 | 85449 |
| rs7521458 | 1 | 154407713 | T | C | 0.5675 | -0.971 | 0.0035 | 70934.9 | 1,00E-300 | 0.583308 | 0.00313445 | 0.00412773 | 0.45 | 85449 |
| rs7525477 | 1 | 154394297 | A | G | 0.4229 | -0.3304 | 0.0058 | 69968.8 | 1,00E-300 | 0.441637 | -0.00191704 | 0.00429146 | 0.6 | 85449 |
| rs7526131 | 1 | 154425135 | A | G | 0.4368 | 0.8438 | 0.0039 | 70935 | 1,00E-300 | 0.579015 | -0.00527707 | 0.00411487 | 0.21 | 85449 |
| rs7537291 | 1 | 154433407 | A | G | 0.3921 | 0.6207 | 0.005 | 70935 | 1,00E-300 | 0.775456 | -0.0105944 | 0.00486453 | 0.032 | 85449 |
| rs7549250 | 1 | 154404336 | T | C | 0.4425 | 0.8262 | 0.0039 | 70933 | 1,00E-300 | 0.578428 | -0.00343155 | 0.00411876 | 0.43 | 85449 |
| rs7553271 | 1 | 154373231 | T | C | 0.4938 | 0.1259 | 0.0057 | 70933.9 | 1.13e-108 | 0.401902 | 0.00163843 | 0.00416711 | 0.68 | 85449 |
| rs79438587 | 1 | 154342517 | T | C | 0.1639 | 0.3475 | 0.0077 | 69969.9 | 1,00E-300 | 0.171973 | -0.00583622 | 0.00555272 | 0.34 | 85449 |
| rs79794939 | 1 | 154390932 | T | C | 0.0682 | -0.3746 | 0.0106 | 70930 | 5.13e-272 | 0.076817 | 0.00203619 | 0.00761483 | 0.84 | 85449 |
| rs9651053 | 1 | 154359411 | A | G | 0.1076 | -0.1664 | 0.0087 | 70934 | 4.134e-82 | 0.10501 | -0.000920254 | 0.00661353 | 0.89 | 85449 |
| rs9803896 | 1 | 154347450 | A | G | 0.1433 | 0.2744 | 0.0077 | 70935 | 7.89e-275 | 0.149987 | -0.00556992 | 0.00567789 | 0.3 | 85449 |

Instrumental cis SNPs (50K) select for sIL6R to accelerometry measured sleep duration by classical Mendelian randomization analysis. CHR: chromosome; POS: hg19 genomic position; A1: effective allele; A2: the other allele; A1_exp_frq: A1 frequency in the exposure dataset; Beta_exp, effect of A1 for the exposure; SE_exp; standard error for Beta_exp; N_exp: sample size for exposure; P_exp, association p value for exposure; A1_out_frq: A1 frequency in the outcome dataset; Beta_out: effect size of A1 on outcome; SE_out: standard error of Beta_out; N_out: sample size for outcome; P_out: association p value for outcome.

**References**

Ahola-Olli, A.V., Wurtz, P., Havulinna, A.S., Aalto, K., Pitkanen, N., Lehtimaki, T., Kahonen, M., Lyytikainen, L.P., Raitoharju, E., Seppala, I., et al. (2017). Genome-wide Association Study Identifies 27 Loci Influencing Concentrations of Circulating Cytokines and Growth Factors. Am J Hum Genet *100*, 40-50. 10.1016/j.ajhg.2016.11.007.

Dashti, H.S., Jones, S.E., Wood, A.R., Lane, J.M., van Hees, V.T., Wang, H., Rhodes, J.A., Song, Y., Patel, K., Anderson, S.G., et al. (2019). Genome-wide association study identifies genetic loci for self-reported habitual sleep duration supported by accelerometer-derived estimates. Nat Commun *10*, 1100. 10.1038/s41467-019-08917-4.

Ferkingstad, E., Sulem, P., Atlason, B.A., Sveinbjornsson, G., Magnusson, M.I., Styrmisdottir, E.L., Gunnarsdottir, K., Helgason, A., Oddsson, A., Halldorsson, B.V., et al. (2021). Large-scale integration of the plasma proteome with genetics and disease. Nat Genet *53*, 1712-1721. 10.1038/s41588-021-00978-w.

Folkersen, L., Gustafsson, S., Wang, Q., Hansen, D.H., Hedman, A.K., Schork, A., Page, K., Zhernakova, D.V., Wu, Y., Peters, J., et al. (2020). Genomic and drug target evaluation of 90 cardiovascular proteins in 30,931 individuals. Nat Metab *2*, 1135-1148. 10.1038/s42255-020-00287-2.

Gudjonsson, A., Gudmundsdottir, V., Axelsson, G.T., Gudmundsson, E.F., Jonsson, B.G., Launer, L.J., Lamb, J.R., Jennings, L.L., Aspelund, T., Emilsson, V., and Gudnason, V. (2022). A genome-wide association study of serum proteins reveals shared loci with common diseases. Nat Commun *13*, 480. 10.1038/s41467-021-27850-z.

Lane, J.M., Jones, S.E., Dashti, H.S., Wood, A.R., Aragam, K.G., van Hees, V.T., Strand, L.B., Winsvold, B.S., Wang, H., Bowden, J., et al. (2019). Biological and clinical insights from genetics of insomnia symptoms. Nat Genet *51*, 387-393. 10.1038/s41588-019-0361-7.

Ligthart, S., Vaez, A., Vosa, U., Stathopoulou, M.G., de Vries, P.S., Prins, B.P., Van der Most, P.J., Tanaka, T., Naderi, E., Rose, L.M., et al. (2018). Genome Analyses of >200,000 Individuals Identify 58 Loci for Chronic Inflammation and Highlight Pathways that Link Inflammation and Complex Disorders. Am J Hum Genet *103*, 691-706. 10.1016/j.ajhg.2018.09.009.

Pedersen, C.B., Bybjerg-Grauholm, J., Pedersen, M.G., Grove, J., Agerbo, E., Baekvad-Hansen, M., Poulsen, J.B., Hansen, C.S., McGrath, J.J., Als, T.D., et al. (2018). The iPSYCH2012 case-cohort sample: new directions for unravelling genetic and environmental architectures of severe mental disorders. Mol Psychiatry *23*, 6-14. 10.1038/mp.2017.196.

Sun, B.B., Maranville, J.C., Peters, J.E., Stacey, D., Staley, J.R., Blackshaw, J., Burgess, S., Jiang, T., Paige, E., Surendran, P., et al. (2018). Genomic atlas of the human plasma proteome. Nature *558*, 73-79. 10.1038/s41586-018-0175-2.

Wang, H., Lane, J.M., Jones, S.E., Dashti, H.S., Ollila, H.M., Wood, A.R., van Hees, V.T., Brumpton, B., Winsvold, B.S., Kantojarvi, K., et al. (2019). Genome-wide association analysis of self-reported daytime sleepiness identifies 42 loci that suggest biological subtypes. Nat Commun *10*, 3503. 10.1038/s41467-019-11456-7.

Zhang, J., Dutta, D., Kottgen, A., Tin, A., Schlosser, P., Grams, M.E., Harvey, B., Consortium, C.K., Yu, B., Boerwinkle, E., et al. (2022). Plasma proteome analyses in individuals of European and African ancestry identify cis-pQTLs and models for proteome-wide association studies. Nat Genet *54*, 593-602. 10.1038/s41588-022-01051-w.
